# Coagulation factor II receptor-like 1 as a prognostic and immuno-modulatory factor in head and neck squamous cell carcinoma

**DOI:** 10.7717/peerj.20970

**Published:** 2026-03-18

**Authors:** Yong gang Dai, Shiliang Cheng, Wei Wang, Hongya Wang, Lu Zhang, Xuewei Zhuang

**Affiliations:** 1 Department of Clinical Laboratory, Shandong Provincial Third Hospital, Shandong University, Jinan City, Shandong Province, China; 2 Department of Clinical Microbiology Laboratory, Shandong Second Provincial General Hospital, Jinan City, Shandong Province, China

**Keywords:** Head and neck squamous cell carcinoma, F2RL1, Immune infiltration, N6-methyladenosine, Prognosis

## Abstract

**Background:**

Head and neck squamous cell carcinoma (HNSCC) remains a major cause of cancer mortality, and robust biomarkers are needed for prognosis stratification and immuno-oncology research. We investigated coagulation factor II receptor-like 1 (F2RL1) in HNSCC, focusing on expression, clinical outcomes and immune associations.

**Methods:**

RNA sequencing and clinical follow-up data from The Cancer Genome Atlas head and neck squamous cell carcinoma cohort (TCGA-HNSC) were analysed. Three independent Gene Expression Omnibus (GEO) cohorts (GSE9844, GSE55547 and GSE55548) were used for external confirmation, with preprocessing and differential-expression exports generated using Xiantao Academic. Immune infiltration, functional enrichment and multi-omics annotations were assessed. Experimental validation was performed using reverse transcription quantitative polymerase chain reaction (RT-qPCR), western blotting and immunohistochemistry (IHC).

**Results:**

F2RL1 was significantly upregulated in HNSCC and was associated with worse overall survival (OS). Expression differences were consistent across the three GEO cohorts. F2RL1 showed significant associations with immune cell infiltration and immune checkpoint genes, and correlated with DNA methylation features and N6-methyladenosine (m6A) regulators. A nomogram was constructed to estimate 1-, 3- and 5-year OS.

**Conclusion:**

F2RL1 is reproducibly overexpressed in HNSCC and is linked to adverse outcomes and immune-related features, supporting its potential as a prognostic biomarker and candidate therapeutic target.

## Introduction

Head and neck squamous cell carcinoma (HNSCC) refers to a range of tumors that originate from the mucosal layers of the upper respiratory and digestive tracts, encompassing tumors in the mouth, pharyngeum, and larynx. Worldwide, the incidence of HNSCC exceeds 650,000 annual instances, resulting in over 330,000 deaths, positioning it as the sixth most prevalent cancer and the seventh most cause of cancer-related fatalities (Johnson et al., 2020). Despite advancements in surgeries, radiotherapeutic methods, and cancer therapies, the five-year survival rate remains only about 50%, suggesting minimal advancements in recent years (Siegel et al., 2023). This highlights the critical need for innovative diagnostic markers and treatment options, especially for those struggling with persistent or metastatic diseases (Chung & Gillison, 2009).

The protein-activated receptor 2 (PAR2), known as coagulation factor II receptor-like 1 (F2RL1), is crucially involved in numerous biological and pathological processes, including inflammation, pain relief, and cancer development (Adams et al., 2011). Research has demonstrated F2RL1’s essential role in enhancing, extending, and modifying tumor microenvironments in cancers, including breast and gastric (Aman et al., 2017; Carvalho et al., 2018; Huo, Feng & Tang, 2024; Kawaguchi et al., 2020). However, the specific function of F2RL1 in relation to HNSCC remains largely unexplored (Leemans, Snijders & Brakenhoff, 2018). Considering its potential involvement in cancer initiation and immune regulation, F2RL1 presents an intriguing area for further investigation in the context of HNSCC (Zhang et al., 2023).

Based on our previous research, bioinformatics screening identified F2RL1 as closely associated with HNSCC, establishing it as a core differential gene. Experimental validation further revealed significant differences in F2RL1 expression between normal and cancerous cell lines in HNSCC.

The foremost aim of this research is to elucidate the expression patterns of F2RL1 in HNSCC and its engagement with diverse molecular formations, evaluate how F2RL1 impacts transcriptomic variances, identify genes associated with immunity impacted by it, and explore the role of F2RL1 in controlling the invasion of immune cells. Additionally, our studies delve into how changes in the methylation of F2RL1’s DNA and RNA influence the genesis of HNSCC. Furthermore, this study confirms the genetics and protein existence of F2RL1 in both cancer cells and regulated cellular settings. This research provides fresh perspectives on HNSCC development and its impact on the immune system, especially regarding alterations in methylation patterns, targeting the improvement of diagnostic and therapeutic methods (Shen et al., 2022).

We reframed the analysis to avoid any appearance of cherry-picking by implementing an objective, transcriptome-wide discovery → cross-cohort replication → immune cross-check → biological-plausibility pipeline. Under these criteria, F2RL1 was prioritized for in-depth evaluation. We explicitly state that findings are associative rather than causal and position F2RL1 within current HNSCC literature.

## Materials & Methods

### TCGA-HNSCC RNA-seq data collection and processing

#### Data sources and inclusion criteria

Gene-expression and clinical data for HNSCC were obtained from The Cancer Genome Atlas (TCGA-HNSCC) *via* the Genomic Data Commons (GDC, https://portal.gdc.cancer.gov). HTSeq-counts files generated by the STAR pipeline were downloaded and converted to transcripts per million (TPM). Only primary tumour samples with available overall survival (OS) information and follow-up ≥ 30 days were retained; samples lacking key clinicopathologic variables or with duplicated barcodes were excluded. For descriptive comparisons, normal tissue samples from TCGA and matched normal squamous tissues from GTEx were combined using the Toil pipeline where applicable.

Protein-coding genes with median TPM ≥ 0.1 and expression > 0 in at least 20% of tumours were kept for downstream analyses. Clinical variables extracted for multivariable modelling included age at diagnosis, sex, AJCC pathologic T and N stage, overall stage, histologic grade, HPV/p16 status, smoking history, alcohol consumption and receipt of radiotherapy, where available. All pre-processing steps were performed in R (version 4.2.1; R Core Team, 2022) using the TCGAbiolinks, edgeR and DESeq2 packages.

#### Study rationale, data sources and cross-cohort replication (TCGA and GEO)

Building on our prior work on this molecule in other tumour types (*e.g.*, cervical cancer), where it was linked to tumour progression and immune-microenvironment features, we observed a consistent differential-expression signal in head and neck squamous cell carcinoma (HNSCC). We therefore adopted an open-cohort validation strategy combining public datasets and an independent clinical cohort to evaluate its expression pattern, prognostic relevance and immune associations, while maintaining an auditable gene-selection pathway.

TCGA-HNSC served as the discovery/validation cohort. Transcriptomic profiles and clinical follow-up were obtained from the Genomic Data Commons. Samples were included if they had complete gene-expression data and usable survival endpoints (*e.g.*, overall survival), and were excluded if key clinical variables or follow-up were missing. In TCGA, we performed a transcriptome-wide univariable Cox screen to define candidate genes and carried forward F2RL1 for downstream analyses described in “Single-gene correlation screening–DNA methylation and RNA m6A analysis of the F2RL1 gene”.

For external confirmation at the expression level, we included three independent GEO HNSCC microarray cohorts (GSE9844, GSE55547 and GSE55548). These GEO series were processed and analysed in Xiantao Academic (https://www.xiantaozi.com/) using the platform’s standard preprocessing and probe-to-gene annotation workflow. Exported differential-expression tables and quality-control reports are provided for independent checking (Table S2; Data S1: GEO+ImmPort.zip).

To ensure cross-cohort comparability, GEO confirmation focused on effect-direction consistency and statistical significance in tumour-versus-normal differential expression (as exported by Xiantao Academic), with BH adjustment reported where available. Prognostic associations were evaluated in TCGA and further supported by the independent clinical cohort (Tables S5 and S6). A cohort and processing summary is provided in Table S4.

#### Cross-cohort replication in GEO microarray datasets

To independently confirm expression differences across GEO cohorts, we analysed GSE9844, GSE55547 and GSE55548 in Xiantao Academic (https://www.xiantaozi.com/). For each cohort, the platform was used to harmonise data formats (including log2 transformation when required), perform expression normalisation, map probes to gene symbols using the corresponding platform annotation, and summarise multiple probes per gene according to the platform rules. Tumour *versus* normal differential expression was then computed and exported in a limma-style table (logFC, t, *P* value and BH-adjusted *P* value). We compiled these exports into a unified gene-by-cohort replication matrix (Table S2), and provide the original exports and HTML reports for audit (Data S1: GEO+ImmPort.zip).

### Single-gene correlation screening

Correlation analysis was conducted between F2RL1 (ENSG00000164251.5) and all other molecules based on the aforementioned data. The Pearson correlation method with BH correction for *p*-values was employed. Target molecule and overall data were extracted, and pairwise correlation analysis was performed between F2RL1 and all other molecules (Robinson, McCarthy & Smyth, 2010). The results were ranked in descending order based on |correlation_pearson| > 0.3 and |pvalue_pearson| < 0.05.

### Differential expression analysis of mRNA (DEmRNA)

Samples were divided into two groups based on the level of F2RL1 expression for the purpose of conducting differential analysis: a low-expression group (0–50%) and a high-expression group (50–100%). The low-expression group served as the reference group. DESeq2 package was used to analyze differential expression on the raw Counts matrix of selected common data (Love, Huber & Anders, 2014). Threshold: |logFC| > 1. Volcano plots for mRNA and heatmaps showing target genes and co-expressed mRNAs were created using the “ggplot2” package. Immunohistochemistry information regarding F2RL1 was acquired from the Human Protein Atlas (HPA) database (http://www.proteinatlas.org/).

### Patient cohort, ethics approval and immunohistochemistry

Formalin-fixed, paraffin-embedded tumour specimens from 30 patients with primary HNSCC who underwent curative resection between 2019 and 2025 at Shandong Provincial Third Hospital, Shandong University (Jinan, China) were retrospectively collected. All cases had histologically confirmed squamous cell carcinoma and had not received preoperative chemotherapy or radiotherapy. The study was approved by the Ethics Committee of Shandong Provincial Third Hospital, Shandong University (approval No. KYLL-2022052), and written informed consent was obtained from all participants or their legal guardians.

Tissue microarrays were constructed with 1–2 representative cores (1.5–2.0 mm) per case. Sections (four μm) were deparaffinised, rehydrated and subjected to heat-induced antigen retrieval in 10 mM citrate buffer (pH 6.0) at 95 °C for 20 min. Endogenous peroxidase was blocked with 3% hydrogen peroxide for 10 min, followed by 5% normal goat serum for 30 min at room temperature. Sections were incubated overnight at 4 °C with a rabbit monoclonal anti-PAR2 (F2RL1) antibody (clone EPR13675, Abcam, Cambridge, UK; Cat. no. ab180953) diluted 1:200. Staining was performed on a Dako Autostainer Link 48 using a polymer-based horseradish peroxidase detection system and developed with 3,3′-diaminobenzidine (DAB) for 5 min, followed by haematoxylin counterstaining. Normal squamous epithelium and tonsil tissue served as positive controls, and omission of the primary antibody served as the negative control.

F2RL1 immunoreactivity was scored independently by two experienced pathologists blinded to clinical data using an H-score (0–300) combining staining intensity (0–3) and percentage of positive tumour cells. Interobserver agreement was excellent (Cohen’s *κ* = 0.82, 95% CI [0.74–0.90]), and discrepant cases were reviewed jointly to reach consensus. For survival analyses, H-scores were dichotomised at the optimal cut-off derived from time-dependent receiver operating characteristic (ROC) curves.

### Identification of immune-related target genes

Immune-related genes were obtained from the ImmPort database (https://www.immport.org/shared/home). After mapping to gene symbols in our analysis space, 414 ImmPort genes were retained. We intersected TCGA candidates (Table S1) with ImmPort membership to mark immune-gene status (Table S3), and intersected the ImmPort list with the F2RL1-correlated gene set to obtain immune-relevant, F2RL1-associated candidates. The set relationships and the full gene-by-gene membership table are provided, together with the original intersection lists (Data S1: GEO+ImmPort.zip). Protein–protein interaction (PPI) analysis and hub-gene prioritisation were performed using STRING (https://string-db.org/).

### Functional enrichment analysis (GO, KEGG, GSEA)

First, the molecular list was converted to Entrez ID using the org.Hs.eg.db package. Then, hypergeometric analysis of GOKEGG libraries (Homo sapiens) was performed employing the clusterProfiler package (Yu et al., 2012). The results were visually presented using the ggplot2 package. Gene sets for Gene Set Enrichment Analysis (GSEA) were obtained from the Molecular Signatures Database (MSigDB) (https://www.gsea-msigdb.org/gsea/) (Hänzelmann, Castelo & Guinney, 2013).

### Immune-related analysis of F2RL1

For evaluating immune cell infiltration, the single-sample Gene Set Enrichment Analysis (ssGSEA) method implemented in the GSVA package was used with established immune cell marker sets. Associations between gene expression and immune features were assessed using Spearman correlation, with multiple-testing control applied as specified for each analysis. We focused on the relationship between F2RL1 and key immune checkpoint genes (*e.g.*, PDCD1, CTLA4, CD274, LAG3, TIGIT) and representative immune-infiltration scores. The reported correlation statistics for F2RL1–checkpoint associations (effect size,*P* value and BH-FDR, with method labels) are provided in Table S3 (Section B).

### DNA methylation and RNA m6A analysis of the F2RL1 gene

Utilizing the MethSurv platform (biit.cs.ut.ee/methsurv/) researchers gathered information on the commonness of expression patterns and forecasted trends related to F2RL1 CpG methylation occurrences in HNSCC, as documented by Modhukur et al. (2018). Key results related to DNA methylation and the predictive survival of F2RL1 came from employing the MethSurv tool, as elaborated by Chen et al. (2024). The group of genes linked to the m6A gene includes enzymes involved in addition (Writer: WTAP, RBM15, RBM15B, ZC3H13, METTL14, METTL3, VIRMA), identification (Readers: YTHDF1-3, YTHDC1-2, HNRNPC, IGF2BP1-3, RBMX, HNRNPA2B1), and elimination (Erasers: FTO, ALKBH5), in line with the categorization set by Zhao et al. (2023).

### Cell culture

The human head and neck squamous cell carcinoma cell lines HN-6 (RRID:CVCL_8129; Cell Bank of Chinese Academy of Sciences, Cat# CBP60465) and FaDu (RRID:CVCL_1218; ATCC, Cat# HTB-43) were cultured in high-glucose DMEM (Gibco, Cat# 11965092) supplemented with 10% fetal bovine serum (FBS; Gibco, Cat# 10270-106), 100 IU/mL penicillin, and 100 μg/mL streptomycin (HyClone, Cat# SV30010). Cells were maintained at 37 °C in a humidified 5% CO_2_ atmosphere. All cell lines underwent authentication through short tandem repeat (STR) profiling (GenePrint^®^ 24 System; Promega) within six months prior to experimentation and were confirmed mycoplasma-free using the MycoAlert™ PLUS Kit (Lonza; Cat# LT07-705). Morphological consistency and absence of contamination were verified by daily phase-contrast microscopy (Nikon Eclipse TS100).

### RT-qPCR

Total RNA was extracted from HNSCC cell lines and matched tumour/adjacent tissue specimens using TRIeasy™ Total RNA Extraction Reagent (Yeasen, Cat. no. 19202ES60) according to the manufacturer’s protocol. RNA concentration and purity were assessed with a NanoDrop 2000 spectrophotometer (Thermo Fisher Scientific); samples with A260/280 ratios between 1.8 and 2.1 were used for downstream analyses.

For each sample, one µg of total RNA was reverse transcribed into cDNA in a 20 µL reaction using the Hifair^®^ II 1st Strand cDNA Synthesis Kit (Yeasen, Shanghai, China; Cat. no. 11119ES60), following the manufacturer’s instructions. Quantitative PCR was performed in triplicate on an ABI 7500 Real-Time PCR System (Applied Biosystems, USA) using Hifair^®^ II SYBR Green qPCR Master Mix (Low Rox Plus) (Yeasen; Cat. no. 11202ES08). The cycling conditions were: initial denaturation at 95 °C for 5 min, followed by 40 cycles of 95 °C for 10 s and 60 °C for 30 s. A melt curve analysis was performed at the end of each run to confirm amplification specificity. No-template controls and no-RT controls were included to exclude contamination and genomic DNA amplification.

The primer sequences were as follows: Homo-β-actin-F, 5′-CATGTACGTTGCTATCCA GGC-3′; Homo-β-actin-R, 5′-CTCCTTAATGTCACGCACGAT-3′; Homo-F2RL1-F, 5′-GTGTTTGGTGGTTTGCC-3′; Homo-F2RL1-R, 5′-CAGAGGAGGTCAGCCAAG-3′. Relative F2RL1 expression was calculated using the 2′-ΔΔCt method with β-actin as the endogenous control.

### Western blot

After cultivation, the cells were rinsed twice with PBS and then lysed on ice utilizing radioimmunoprecipitation assay (RIPA) buffer. Subsequently, 30 µg of the extracted proteins were subjected to SDS-PAGE on a 20% gel and transferred onto a polyvinylidene difluoride (PVDF) membrane. The membranes were then incubated with primary antibodies targeting F2RL1 (1:500; Abcam) and GAPDH (1:20,000; Abcam), followed by HRP-conjugated secondary antibodies (1:20,000; Thermo). Signal detection was carried out using enhanced chemiluminescence (ECL) in conjunction with chemiluminescent reagents, and the protein bands were subsequently analyzed utilizing Image J software.

### Statistical analysis

SPSS, GraphPad Prism, or R software were frequently used for statistical analysis in different studies. To assess the connection among gene expression, clinical pathology, and survival rates, tests such as Wilcoxon rank-sum, Kruskal–Wallis, and Cox regression analysis were employed, with a *P*-value below 0.05 considered statistically significant. Moreover, the data was visually represented through instruments such as volcanic charts, Venn diagrams, and Kaplan–Meier curves. Common signs of statistical significance included *P < 0.05, **P < 0.01, and ***P < 0.001.

## Results

### Differential expression of F2RL1 in HNSCC and normal tissues

**Figure 1:**
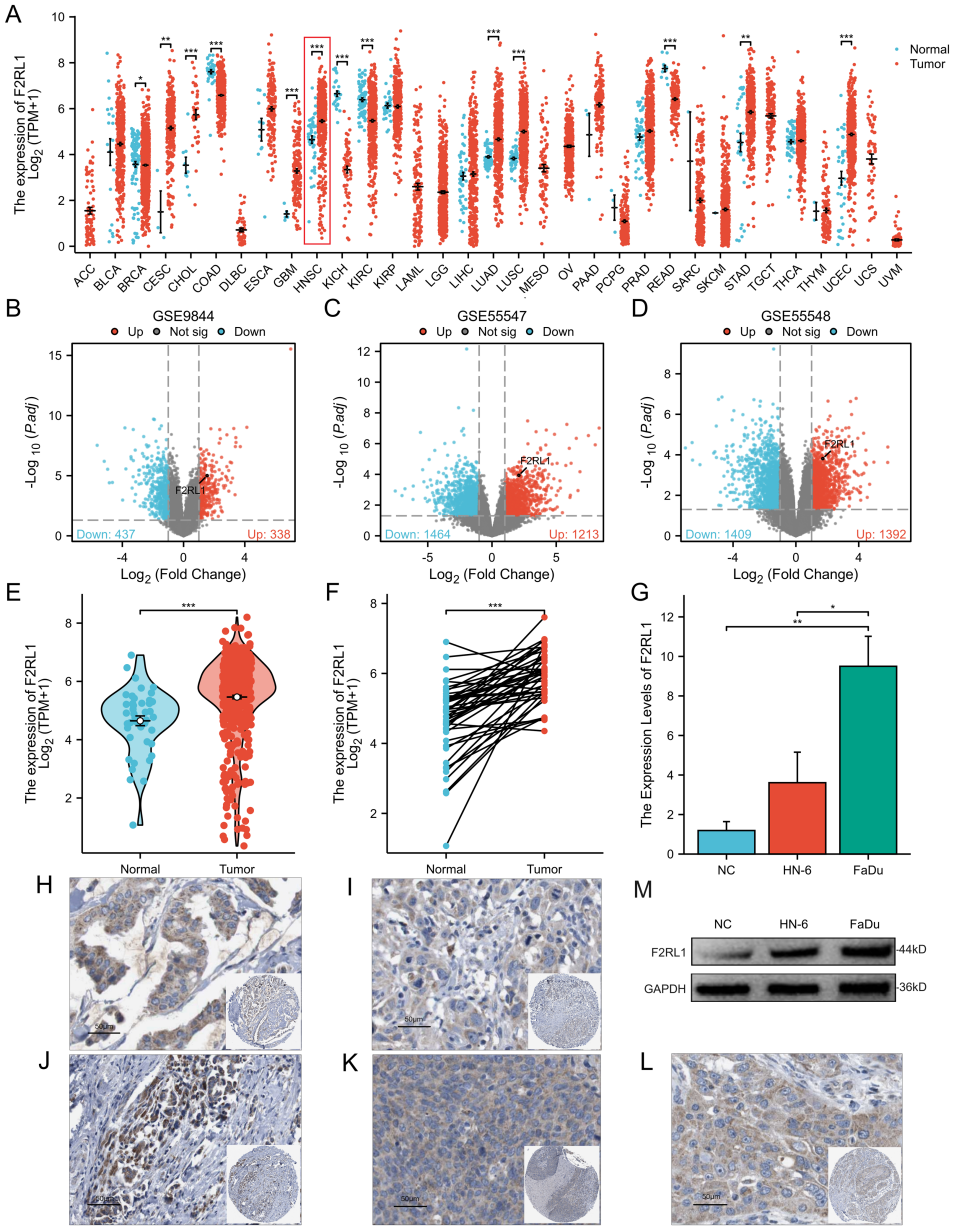
Differential expression of F2RL1 in HNSCC and normal tissues. (A)The expression pattern of F2RL1 across 33 cancer types in the TCGA database. (B–D) Volcano plots showing the differential expression of molecules in the GSE9844, GSE55548, and GSE55547 GEO datasets, with circled positions representing F2RL1. (E) Upregulated expression of F2RL1 in HNSCC compared to normal tissues. (F) Upregulated expression of F2RL1 in paired tissue samples of HNSCC. (G) Validation of F2RL1 expression by RT-qPCR, with experimental groups including HNSCC tumor tissues and related cell lines FaDu and HN-6, and a control group consisting of normal human oral epithelial cells. (M) Western blot results for each group. (H–L) Immunohistochemical staining of F2RL1 in HNSCC from various sources in the HPA database, including adenocarcinoma (H, J), squamous cell carcinoma of oral tissue (I), lymph node (K), skeletal muscle (L).

We profiled F2RL1 expression across 33 cancer types and observed elevated expression in HNSCC. In TCGA-HNSC, F2RL1 expression was significantly higher in tumour tissues than in normal controls, and this pattern remained consistent in paired tumour-normal samples (Figs. 1B–1F). The overexpression of F2RL1 was further confirmed in three independent GEO cohorts with survival follow-up (GSE9844, GSE55547 and GSE55548; Figs. 1B–1D; full cohort-wise statistics in Table S2). We also validated F2RL1 expression in HNSCC cell lines by RT-qPCR using a normal oral epithelial cell line as the control (Fig. 1G). In addition, immunohistochemistry images from the Human Protein Atlas supported cytoplasmic F2RL1 staining across HNSCC tissues from multiple anatomical sources (Figs. 1H–1L).

**Figure 2:**
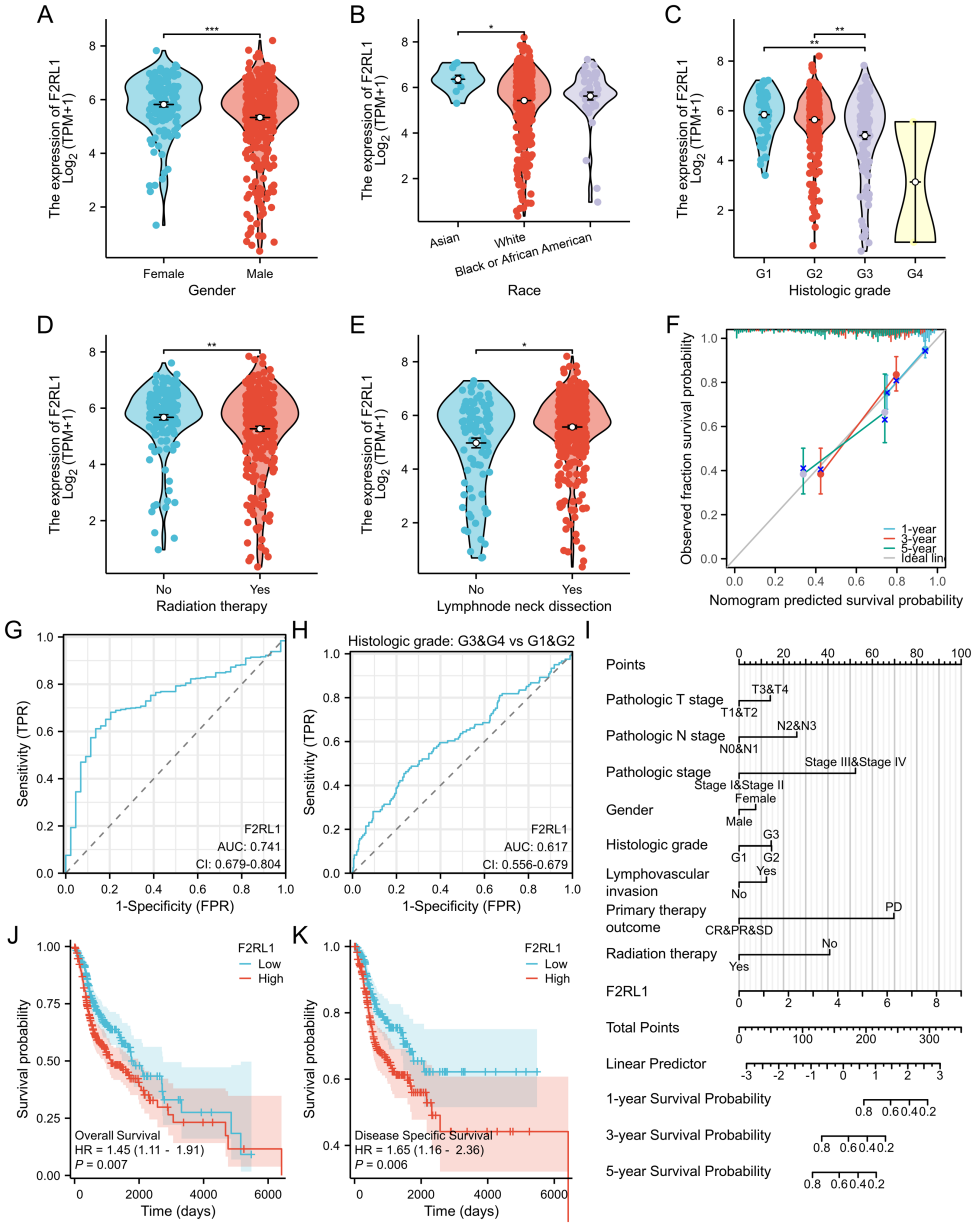
Examines the role of F2rl1 in the survival, prognosis, and diagnosis of HNSCC patients. (A–E) Comparison of F2RL1 levels in HNSCC patients with various clinical variables revealed significant differences in gender (A), race (B), histology grade (C), radiation therapy (D), and neck lymph node metastasis (E). (G) ROC analysis showed an AUC of 0.741. (H) ROC curve for the histology grade clinical variable (G1&G2 *vs* G3&G4) had an AUC of 0.617. (J, K) Kaplan–Meier curves for OS and DSS according to different F2RL1 expression levels. (I) Line plots illustrating the nanogram clinical prediction models for 1-year, 3-year, and 5-year survival of HNSCC patients. (F) Prognostic calibration correction curve.

### External patient validation and objective selection identifies F2RL1

IHC confirmed that higher F2RL1 (H-score) was associated with poorer outcomes and adverse clinicopathological features in the independent patient cohort. The raw clinical and IHC scoring data are provided in Table S5, and paired RT-qPCR validation data are provided in Table S6. Together with the GEO expression confirmation matrix (Table S2), and ImmPort intersection outputs (Table S3; original exports in Data S1), these materials enable step-by-step auditing of the selection pathway leading to F2RL1.

### Role of F2RL1 in survival, prognosis, and diagnosis of HNSCC patients

Next, we investigated whether the expression levels of F2RL1 could impact the survival, prognosis, and diagnostic value of HNSCC patients. Firstly, we compared different clinical significance groups based on F2RL1 expression levels utilizing the TCGA database. We analyzed the differences between selected clinical variable groups based on F2RL1 expression, including gender (Fig. 2A), race (Fig. 2B), histology grade (Fig. 2C), radiation therapy (Wang et al., 2015) (Fig. 2D), and neck lymph node metastasis (Fig. 2E). ROC analysis revealed an area under the curve (AUC) of 0.741 (Fig. 2G) when comparing HNSCC tissues with normal tissues. When incorporating the clinical variable histology grade (G1 *vs* G2), the AUC was 0.617 (Fig. 2H), indicating good diagnostic value of F2RL1 in HNSCC. Subsequently, we conducted survival analysis of HNSCC patients with different expression levels of F2RL1. The Kaplan–Meier curves demonstrated significantly higher overall survival (OS) and disease-specific survival (DSS) in low-expression patients compared to high-expression patients (Figs. 2J–2K), suggesting F2RL1 as an adverse factor for HNSCC patients. Finally, we utilized univariate and multivariate COX regression analysis on the aforementioned clinical variables to construct nanogram clinical prediction models for 1,3,5-year survival of HNSCC patients (Table 1), with corresponding forest plots (Fig. 2I) and calibration plots (Fig. 2F).

**Table 1:** Univariate and multivariate Cox regression analyses of F2RL1 expression in HNSCC.

**Characteristics**	**Total(N)**	**Univariate analysis**		**Multivariate analysis**	
		**Hazard ratio (95% CI)**	***P*** **value**	**Hazard ratio (95% CI)**	***P*** **value**
Pathologic T stage	445				
T1&T2	178	Reference		Reference	
T3&T4	267	1.913 (1.397–2.621)	<0.001	1.318 (0.662–2.625)	0.432
Pathologic N stage	408				
N0&N1	236	Reference		Reference	
N2&N3	172	2.288 (1.679–3.118)	<0.001	1.668 (1.000–2.780)	0.050
Pathologic stage	433				
Stage I&Stage II	94	Reference		Reference	
Stage III&Stage IV	339	1.834 (1.232–2.729)	0.003	2.809 (0.955–8.261)	0.061
Gender	501				
Female	133	Reference		Reference	
Male	368	0.750 (0.563–0.999)	0.049	0.864 (0.538–1.386)	0.544
Histologic grade	479				
G1	61	Reference		Reference	
G2	299	1.752 (1.104–2.779)	0.017	1.333 (0.520–3.420)	0.549
G3	119	1.509 (0.915–2.488)	0.107	1.329 (0.467–3.784)	0.594
Lymphovascular invasion	339				
No	219	Reference		Reference	
Yes	120	1.697 (1.207–2.384)	0.002	1.274 (0.771–2.104)	0.344
Primary therapy outcome	416				
PD	41	Reference		Reference	
CR&PR&SD	375	0.155 (0.103–0.232)	<0.001	0.254 (0.136–0.472)	<0.001
Radiation therapy	439				
No	153	Reference		Reference	
Yes	286	0.627 (0.462–0.851)	0.003	0.448 (0.264–0.761)	0.003
F2RL1	501	1.245 (1.105–1.403)	<0.001	1.244 (0.978–1.583)	0.075

### Relationship between F2RL1 and immune-related genes, and selection of HUB genes

**Figure 3:**
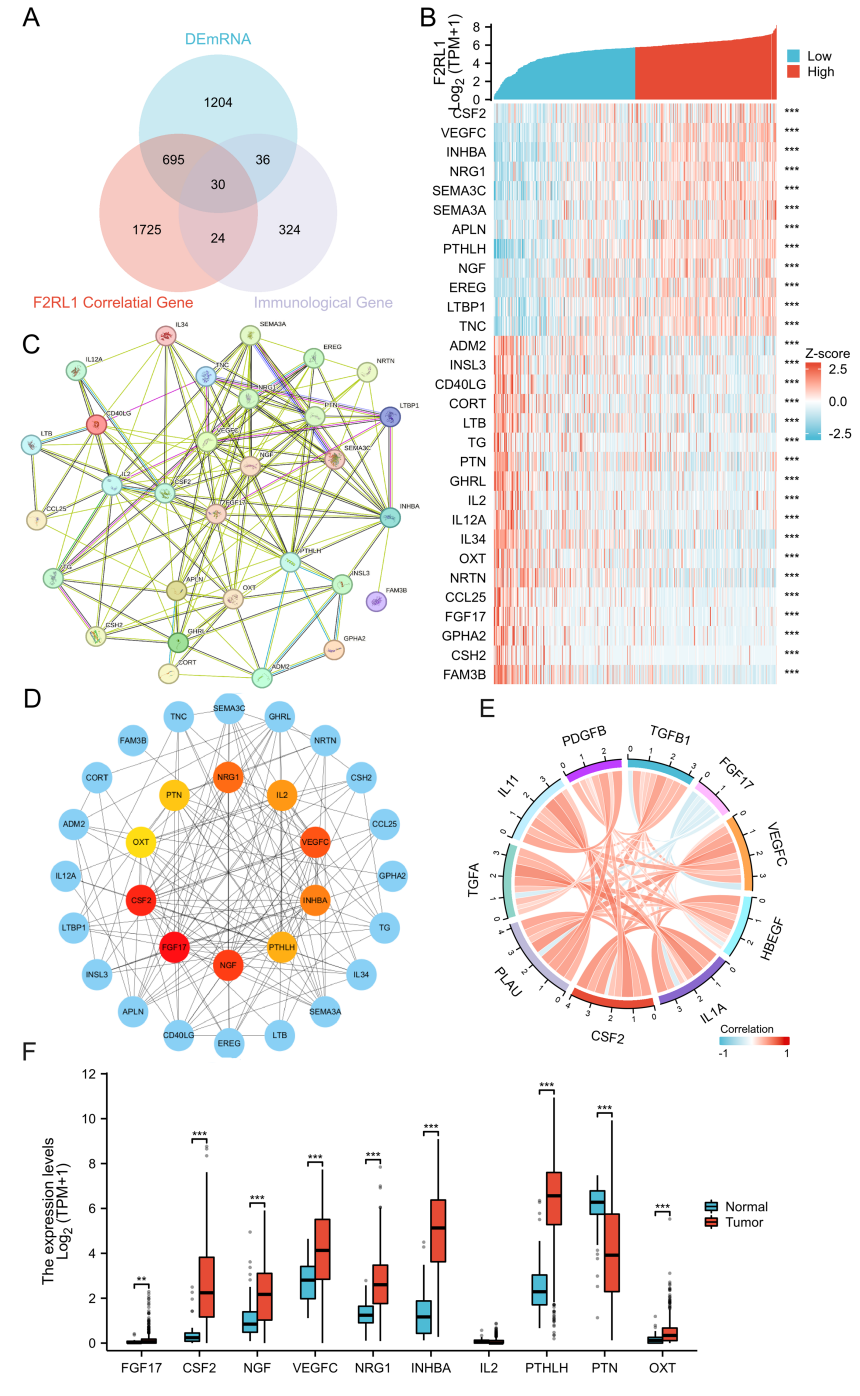
Relationship between F2RL1 and immune-related genes and selection of HUB genes. (A) Venn diagram illustrating immune-related genes. (B) Heatmap displaying the co-expression of immune-related genes and F2RL1. (C) Protein-protein interaction analysis of immune-related genes. (D) Selection of 10 HUB genes utilizing the Cytoscape plugin CytoHubba. (E) Chord diagram showing the correlation between HUB genes. (F) Comparison of expression levels of HUB genes in HNSCC tissues and normal tissues.

To examine whether F2RL1-linked transcriptional changes were enriched for immune biology, we first identified F2RL1-correlated genes in TCGA (|r| > 0.3, *P* < 0.05; Table S1) and mapped immune-related genes from ImmPort. After mapping, 414 ImmPort genes were retained, and their intersection with the F2RL1-correlated gene set yielded 54 immune-relevant, F2RL1-associated genes. Intersecting these with the DEmRNA list further produced a final set of 30 immune-related candidates (Table S3; Data S1). We then constructed a STRING PPI network for these candidates and prioritised hub genes for downstream analyses. The intersection outputs, PPI network and hub-gene prioritisation are shown in Fig. 3.

### GO/KEGG and GSEA of F2RL1 in HNSCC

**Figure 4:**
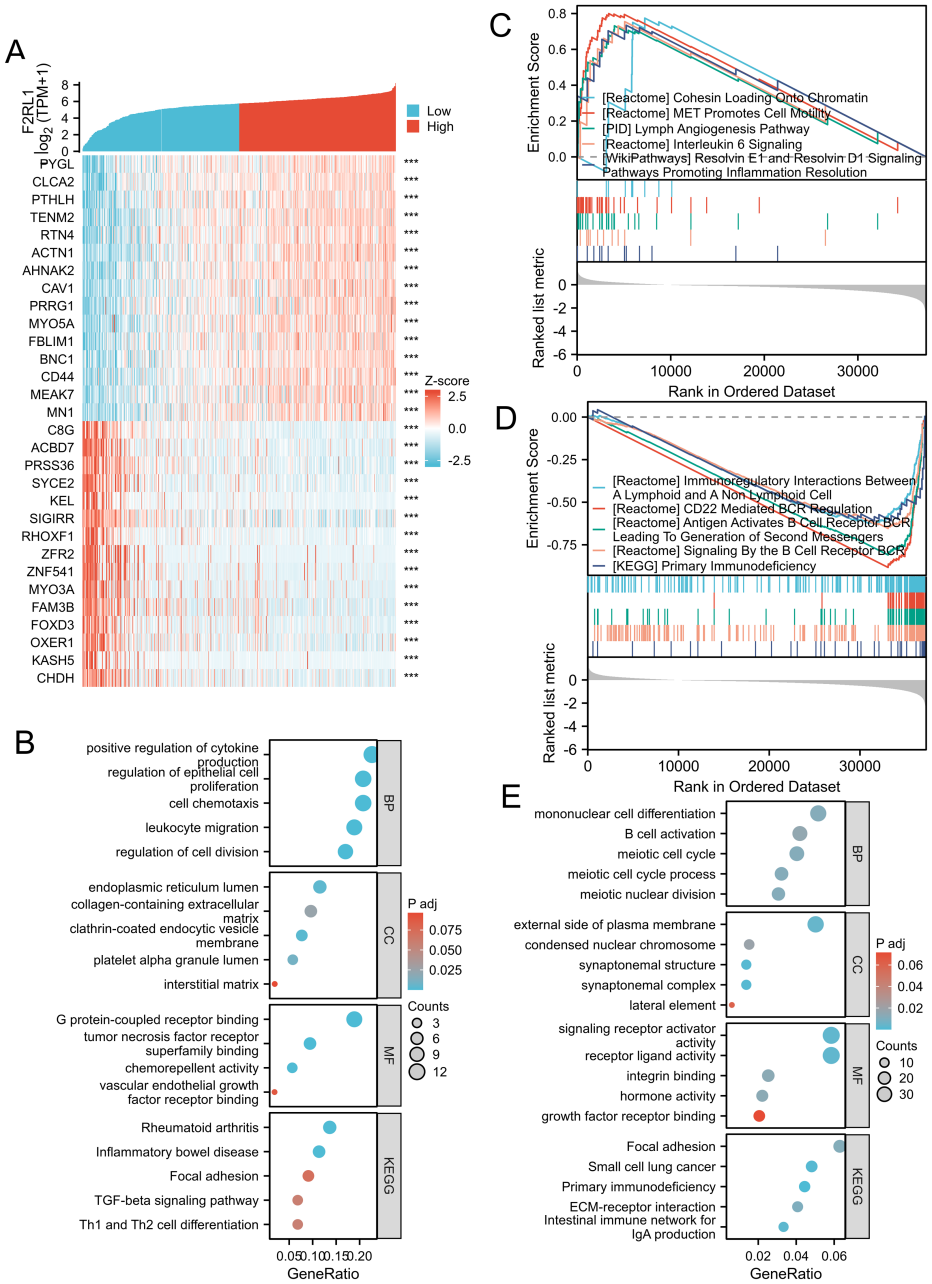
Functional clustering analysis of F2RL1-associated genes. (A) Heatmap displaying the top 15 genes positively and negatively correlated with F2RL1 expression in HNSCC. (B) GO and KEGG analysis performed on F2RL1-associated genes in HNSCC. (C, D) GSEA analysis conducted on F2RL1-associated genes in HNSCC. (C) Represents positive correlation, while (D) represents negative correlation. (E) GO and KEGG analysis conducted on F2RL1 with immune-related genes in HNSCC.

To understand the biological functions of F2RL1 in HNSCC, we conducted single-gene differential analysis. Using |logFC| > 1.5 and *P* < 0.05 as criteria, we identified F2RL1-differentially expressed mRNAs (DEmRNAs). The heatmap presented the top 15 genes that exhibited either positive or negative correlations with F2RL1. Notably, the top five genes showing positive correlation were PYGL, CLCA2, PTHLH, TENM2, and RTN4, whereas the top five genes displaying negative correlation were C8G, ACBD7, PRSS36, SYCE2, and KEL (Fig. 4A). Additionally, the intersection of DEmRNAs and immune-related genes were subjected to GO and KEGG pathway analysis, revealing associations with extracellular matrix and cell adhesion, immune cell migration and chemotaxis, autoimmune diseases, and immune cell differentiation (Figs. 4B, 4E). We ranked these selected functional molecules based on their corresponding |logFC| values and performed GSEA enrichment analysis using the MSigDB database, identifying 10 pathways that were positively or negatively correlated (Figs. 4C, 4D). These pathways primarily focused on immune cell activation and regulation, cell motility, chromatin replication, and other biological processes. Through these analyses, we found that the biological functions of F2RL1 in HNSCC primarily relate to immunity and cell proliferation. Given the close relationship between cell proliferation and DNA methylation, our next step will involve analyzing the immune infiltration and DNA/RNA methylation (m6A) of F2RL1 in HNSCC.

**Figure 5:**
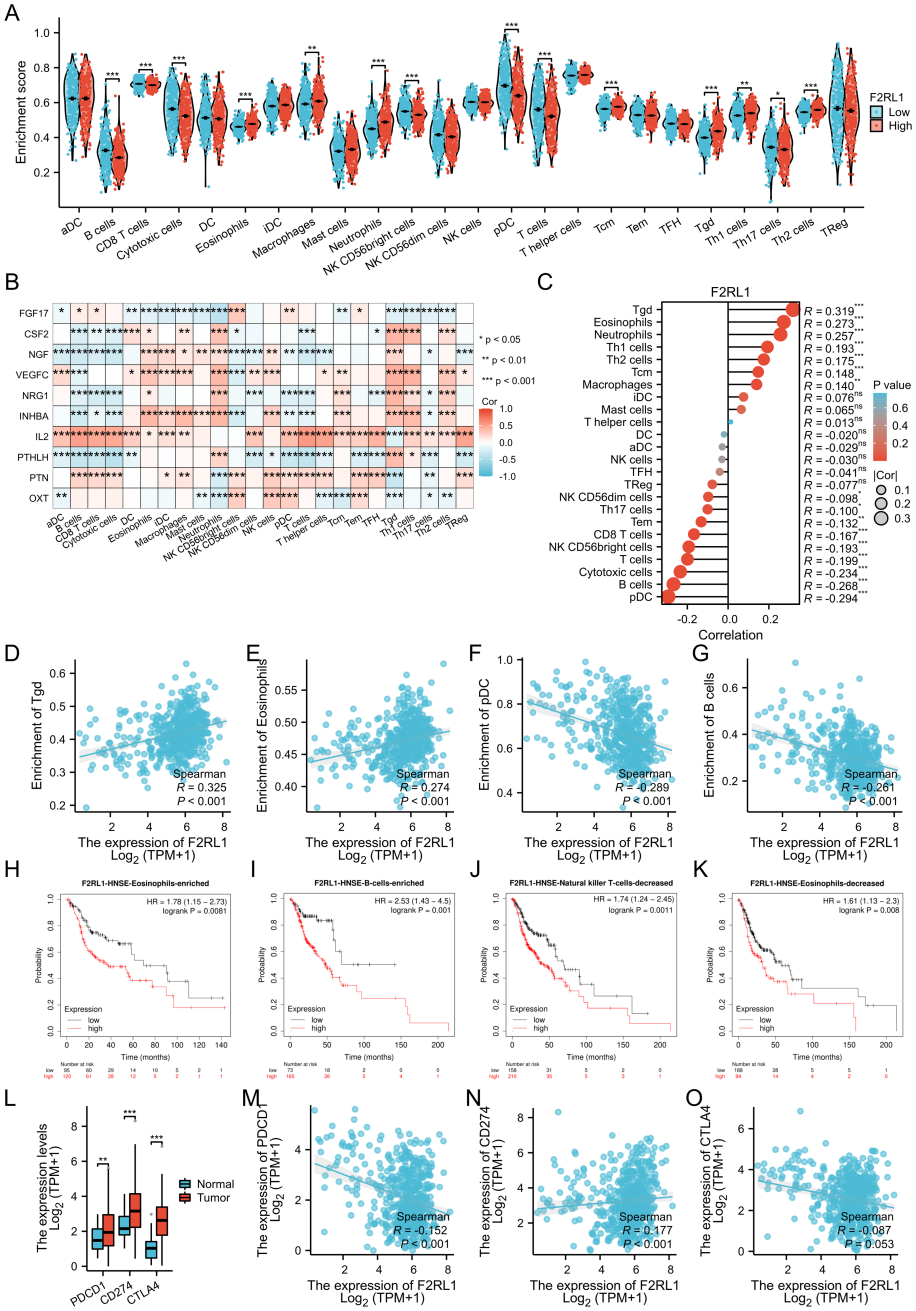
Immune infiltration analysis of F2RL1. (A) Comparison of F2RL1 expression levels and enrichment scores of 24 immune cell types. (B) Heatmap displaying the correlation between F2RL1-related 10 HUB genes and 24 immune cell types. (C) Lollipop chart exhibiting the correlation results between F2RL1 and immune infiltration. (D–G) Analysis of the correlation between F2RL1 and individual immune cells in HNSCC. (H–K) Impact of F2RL1 on survival curves with different immune cell infiltration patterns in HNSCC. (L–O) Immune checkpoints associated with F2RL1 in HNSCC.

### Immune infiltration analysis of F2RL1 in HNSCC

Based on the previous enrichment analysis and molecular screening results, we have discovered the significant role of F2RL1 in immune function.The presence of immune cells plays a significant role in the growth and advancement of tumors. Therefore, we proceeded to analyze the immune infiltration of F2RL1 in HNSCC. Initially, we compared the expression levels of F2RL1 with the enrichment scores of 24 immune cells (Fig. 5A) and observed differences in the Enrichment scores of F2RL1 with most immune cells in HNSCC. Following that, we conducted a correlation analysis between the 10 identified HUB genes associated with F2RL1 and the levels of infiltration of 24 immune cells. The results were visualized in a heatmap (Fig. 5B). We found correlations between HUB genes and most immune cells, with IL2 and INHBA showing predominantly positive correlations, while FGF17 and PTHLH showed predominantly negative correlations. Subsequently, we visualized the correlation results between F2RL1 and immune infiltrating cells in the form of lollipop plots (Fig. 5C), which revealed a relatively equal distribution of positive and negative correlations. We further displayed scatter plots of the two highest-ranking cell types correlated with F2RL1 (Figs. 5D–5G), where Tgd cells (D, *R* = 0.325) and Eosinophils (E, *R* = 0.274) showed positive correlations, while pDC (F, *R* = 0.289) and B cells (G, *R* = 0.261) showed negative correlations(Chi et al., 2022). We analyzed the impact of different patterns of immune cell infiltration in HNSCC with F2RL1 on survival outcomes by examining their effects on KM survival curves (Figs. 5H–5K). We selected enriched patterns of Eosinophils (H, HR = 1.78) and B cells (I, HR = 2.53), as well as decreased patterns of NK cells (J, HR = 1.74) and Eosinophils (K, HR = 1.61) for display (H-K). Lastly, we conducted an analysis of the relationship between immune checkpoint and F2RL1 in HNSCC. We selected PD1 (PDCD1), PDL1 (CD274), and CTLA4 related to immune therapy and compared their expression levels between HNSCC tissues and normal tissues (Bareche et al., 2022) (Fig. 5L). We discovered that the levels of these three immune checkpoints were elevated in HNSCC tissues compared to normal. Moreover, we observed a positive correlation between F2RL1 and PDL1 (CD274), while a negative correlation was found between F2RL1 and PD1 (PDCD1) in HNSCC (Figs. 5M–5O).

### DNA methylation analysis of F2RL1 gene in HNSCC

**Figure 6:**
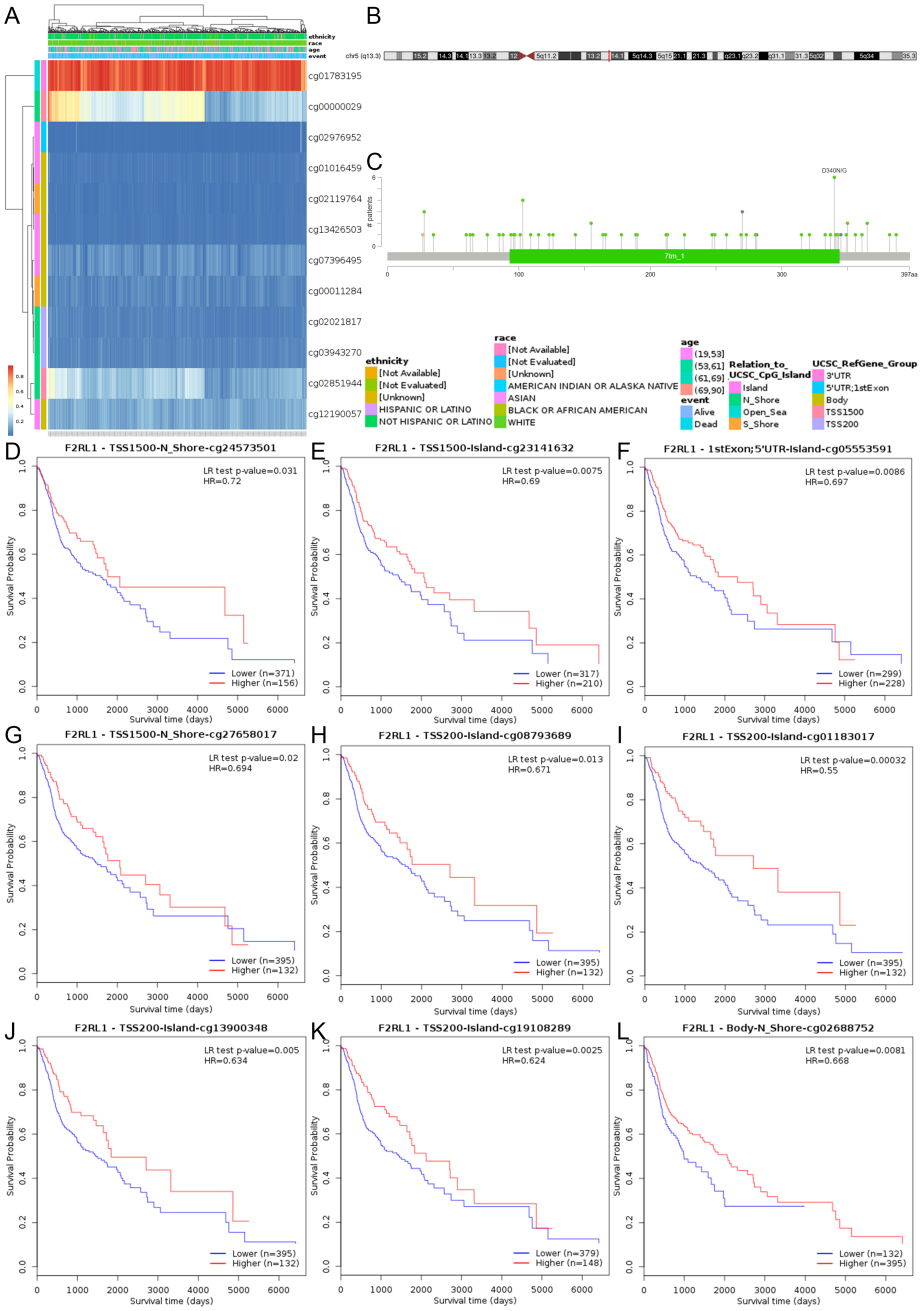
DNA methylation status of the F2RL1 gene in HNSCC from TCGA. (A) Heatmap depicting the methylation levels of the F2RL1 gene in HNSCC. (B) Genomic localization of the F2RL1 gene on the chromosome. (C) Common mutation sites and frequencies of the F2RL1 gene in HNSCC. (D–L) Kaplan–Meier curves illustrating the prognostic significance of individual CpG sites within the F2RL1 gene in HNSCC.

In our analysis, we discovered a link between F2RL1 and cell division in HNSCC, which plays a crucial role in tumor cells, and is closely related to DNA methylation (Huang et al., 2023; Wu, Xu & Cheng, 2021). CpG island methylation of DNA methyltransferases is a transcription factor that can inhibit or promote cell growth, and this process is reversible. Firstly, we displayed the methylation levels of the F2RL1 gene in HNSCC using a heatmap (Fig. 6A). Next, we reviewed the chromosomal location of F2RL1 (located in the q13.3 region of chromosome 5) and the common mutation sites of the F2RL1 gene in HNSCC (Fig. 6B), with D340N/G being the most common mutation site (Fig. 6C). Finally, we confirmed the significant prognostic value of the DNA methylation pattern of the F2RL1 gene utilizing the MethSurv database, such as cg02688752, cg24573501, cg27658017, cg01183017, cg08793689, cg13900348, cg19108289, cg05553591, and cg23141632 (Figs. 6D–6L, Table 2).

### Relationship between F2RL1 expression and m6A modification in HNSCC

**Figure 7:**
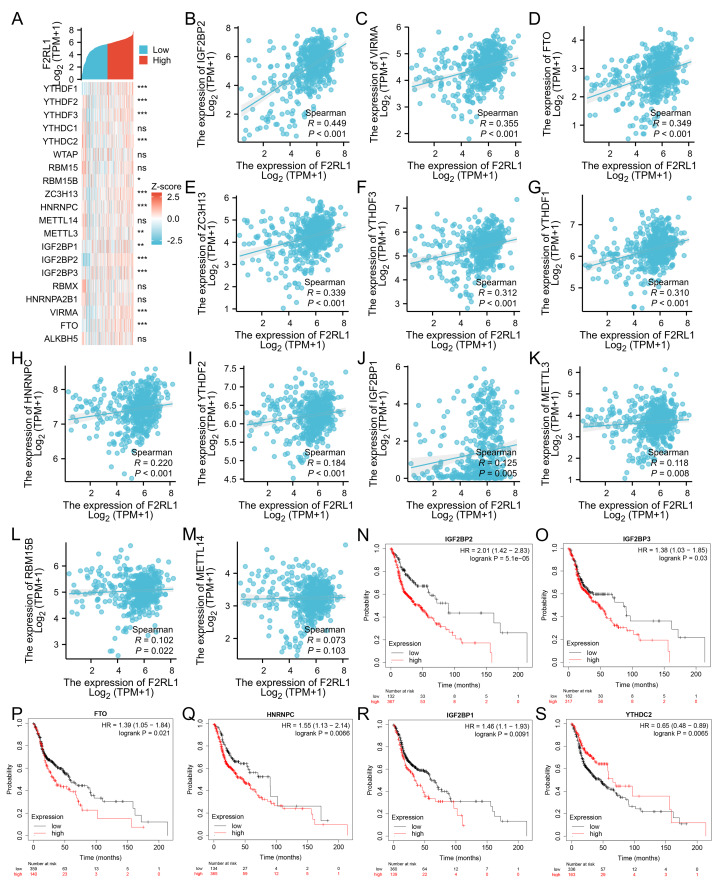
Relationship between F2RL1 and m6A-related genes in HNSCC. (A) Heatmap illustrating the co-expression of F2RL1 with 20 m6A-related genes in HNSCC. (B–M) Scatter plots showing the co-expression of F2RL1 with m6A-related genes in HNSCC. Significantly different expressions were observed for IGF2BP2, VIRMA, FTO, ZC3H13, YTHDF3, HNRNPC, YTHDF1, YTHDF2, IGF2BP1, METTL3, RBM15B, and METTL14. (N–S) Kaplan–Meier survival curves depicting the prognostic significance of F2RL1 with 20 m6A-related genes in HNSCC. Among them, IGF2BP2, IGF2BP3, FTO, HNRNPC, IGF2BP1, and YTHDC2 exhibited prognostic value in HNSCC.

The m6A RNA methylation process is crucial in HNSCC, suggesting a possible link between F2RL1 expression and its m6A methylation level. To explore this relationship, we investigated 20 m6A-related genes and found that F2RL1 expression was significantly positively correlated with IGF2BP2, VIRMA, FTO, ZC3H13, YTHDF3, HNRNPC, YTHDF1, YTHDF2, IGF2BP1, METTL3, RBM15B, and METTL14 (*p* < 0.001) (Fig. 7A). Figures 7B–7M display scatter plots of the co-expression of F2RL1 with 12 m6A-related genes (pearman *r* > 0.3, *p* < 0.001). As shown in the figures, in the m6A process, F2RL1 was significantly positively correlated with Writers (VIRMA, ZC3H13), Readers (YTHDF1, YTHDF3, IGF2BP2), and Eraser (FTO) (|R| > 0.3). We also observed that IGF2BP1-3, HNRNPC, and FTO had poorer prognosis in HNSCC (Figs. 7N–7S). This indicates that there could be a regulatory connection between Readers (IGF2BP1-3, HNRNPC) and Eraser (FTO) with F2RL1, thereby affecting patient prognosis.

## Discussion

HNSCC is a severe and prevalent cancer that originates in the mucosal layers of the oral cavity, pharynx, and larynx, accounts for approximately 90% of these cancers and poses significant health risks and complications (Bray et al., 2018). The primary hazards associated with the development of HNSCC include the intake of tobacco, alcohol, and infections caused by human papillomavirus (HPV) (Shield et al., 2017). HNSCC commonly emerges in advanced stages (Chen et al., 2024), highlighting the importance of developing pioneering biomarkers and therapeutic objectives to improve early detection, treatment, and patient outcomes. The complex traits of HNSCC, including histological variations and genetic heterogeneity, make treatment approaches complicated (Leemans, Snijders & Brakenhoff, 2018).

**Table 2:** Effect of high F2RL1 methylation levels at different CpG sites on the prognostic value of HNSCC.

**Name**	**CpG**	**HR**	**Wald** ***P*** **value**
cg02688752	Body	0.668	0.0065
cg24573501	TSS1500	0.72	0.036
cg27658017	TSS1500	0.694	0.024
cg01183017	TSS200	0.55	0.00072
cg08793689	TSS200	0.671	0.017
cg13900348	TSS200	0.634	0.0073
cg19108289	TSS200	0.624	0.0037
cg05553591	1stExon: 5‘UTR	0.697	0.0095
cg23141632	TSS1500	0.69	0.0085

Genetic factors, such as the F2RL1 element, play a role in various biological processes including inflammation, immune system regulation, and cancer evolution (Cwiklinski et al., 2024; Ramachandran et al., 2012). These factors are believed to influence tumor progression, cell development, and therapy response in HNSCC, where different genetic expressions may impact cancer progression and region-specific adaptation (Chen et al., 2023; Wang et al., 2022; Yang et al., 2017).

This study delves into the expression patterns of F2RL1 in HNSCC, exploring its interplay with various molecular markers and its effect on the tumor’s immunological milieu. Additionally, our study sought to elucidate F2RL1’s influence on diverse mRNA expression patterns and identify immune system-related genes, to augment our understanding of HNSCC, and to investigate potential treatment routes (Yang et al., 2023). Furthermore, our study explored the connection between F2RL1 and immune-related genes, shedding light on the tumor’s environment and tactics for dodging the immune response in HNSCC. Investigating the effects of F2RL1 DNA methylation and RNA m6A modifications on HNSCC progression was a key element of our dialogues.

Our research showed that F2RL1 levels were significantly elevated in HNSCC and correlated with poor patient survival outcomes, consistent with previous studies that identified F2RL1 as an indicator of cancer progression and treatment strategies. The heightened expression of F2RL1 was confirmed using three separate datasets from the GEO employing RT-qPCR and Western blotting methods across multiple cancerous and cellular lines (FaDu, HN-6). The findings demonstrated its proficiency in identifying HNSCC (AUC = 0.7) and differentiated it across different histopathological stages (G1 & G2 *vs.* G3 & G4). Functional advancements *via* GO, KEGG, and GSEA underscored the significant function of F2RL1 in activating immune cells, promoting lung cancer, and enhancing innate immune responses, highlighting its intricate role in the pathogenesis of HNSCC. Our enrichment research highlighted the critical role of F2RL1 in stimulating immune cells and meiosis, vital stages in tumor development, igniting discussions on the impacts of DNA and RNA methylation mechanisms (Diagouraga et al., 2018).

Our research revealed that fluctuations in F2RL1 expression were observed in relation to several genes linked to the immune state, particularly FGF17 and CSF2, highlighting the role of F2RL1 in modifying the immune response to HNSCC. The examination of the protein-protein interaction network highlighted the crucial function of these genes in the context of the immune function in HNSCC. The correlation between F2RL1 levels and immune cell infiltration trends in HNSCC emphasizes their critical role in shaping the immune landscape and affecting patient outcomes. The clear determination of the rates at which immune cells infiltrate indicates that F2RL1 functions to either amplify or debilitate immune responses in the tumor microenvironment (Ke et al., 2020), paving the way for novel immunological therapies.

Furthermore, the research highlights the significance of F2RL1 DNA methylation patterns in HNSCC, with specific methylation sites identified as crucial indicative factors (Li et al., 2019; Pawlowski et al., 2022). There is a significant link between F2RL1 expression and key m6A RNA methylation regulators like IGF2BP2 and VIRMA, indicating that post-transcription alterations might affect patient outcomes and be potential targets for treatment (Chen et al., 2021; Zhu et al., 2023).

Limitations: Despite pre-specified workflows and FDR control across discovery, replication, and external validation, heterogeneity (subsite, HPV/p16, treatment), residual confounding, and data-driven cut-points may persist. We mitigated these with subgroup analyses and multivariable models. As a correlational study, mechanistic causality requires prospective and functional validation. The F2RL1 association supports hypothesis generation for prognostic stratification and potential immunotherapy relevance. Next steps include prospective validation and mechanistic studies (*e.g.*, F2RL1–m6A/IGF2BP axis), avoiding overstatement until causal evidence emerges.

In summary, this research emphasizes the importance of F2RL1 in diagnosing, predicting, and treating HNSCC, illustrating its intricate ties with the immune system and the complexities of the tumor’s environment (Joyal et al., 2014). Considering constraints such as sample size and variability of the dataset, it is crucial to extend the scope of our study sample..

## Conclusions

The comprehensive research examined the impact of the F2RL1 gene on head and neck squamous cell carcinoma (HNSCC) by leveraging various accessible sources, including TCGA, GEO, Kaplan–Meier Plotter, TIMER, and STRING, supported by fundamental experimental investigations. Pioneering studies illuminated F2RL1’s function in predicting and diagnosing HNSCC, and its connection to genes involved in immune reactions and cell penetration. Altered methylation patterns of F2RL1 in DNA and m6A were also analysed in different HNSCC cases. Cumulative evidence suggests that F2RL1 may serve as a critical prognostic factor and a primary therapeutic target in HNSCC patients.

## Supplemental Information

10.7717/peerj.20970/supp-1 Supplemental Information 1 Supplementary Tables

10.7717/peerj.20970/supp-2 Supplemental Information 2 Supplementary Figures

10.7717/peerj.20970/supp-3 Supplemental Information 3 Code and parameters

10.7717/peerj.20970/supp-4 Supplemental Information 4 GEO+ImmPort

10.7717/peerj.20970/supp-5 Supplemental Information 5 Raw Data

10.7717/peerj.20970/supp-6 Supplemental Information 6 MIQE checklist

10.7717/peerj.20970/supp-7 Supplemental Information 7 WB Image LANE LABELS labels and MOLECULAR WEIGHTS labels

10.7717/peerj.20970/supp-8 Supplemental Information 8 Raw data of qPCR (Fig. 1G)

## Additional Information and Declarations

### Data Availability

The following information was supplied regarding data availability: The raw data is available in the Supplemental Files.
