# Supplementary material for: Coagulation factor II receptor-like 1 as a prognostic and immuno-modulatory factor in head and neck squamous cell carcinoma
Source: PeerJ. 2026 Mar 18;14:e20970. doi: 10.7717/peerj.20970 (PMC13005615; doi:10.7717/peerj.20970)
Supplement: Supplemental Information 2 [file peerj-14-20970-s002.docx]

**Supplementary_Figure**


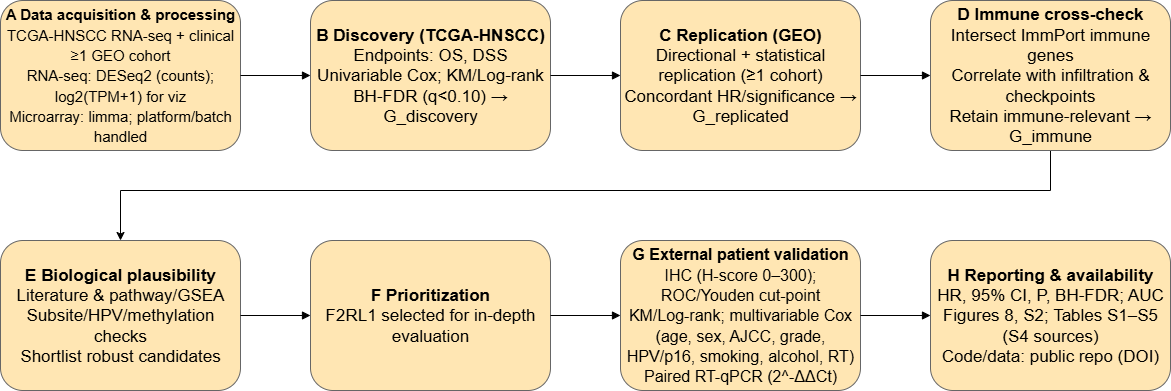


**Figure S1. Pre-specified pipeline for discovery, replication, immune cross-check, plausibility and external validation.**
**(A) Data acquisition & processing:** TCGA-HNSCC (RNA-seq + clinical) and ≥1 independent GEO cohorts; RNA-seq analyzed with DESeq2 on raw counts; microarrays with limma; log2(TPM+1) used for correlation/visualization; prespecified inclusion/exclusion (subsite, HPV/p16, follow-up).
**(B) Discovery (TCGA-HNSCC):** Transcriptome-wide screen for OS/DSS using univariable Cox and KM/Log-rank; multiple testing controlled by BH-FDR (q<0.10) to obtain the discovery set (G_discovery).
**(C) Replication (GEO):** Directional and statistical replication in ≥1 external cohort; genes with concordant HR and significance retained (G_replicated).
**(D) Immune cross-check:** Intersect with ImmPort immune genes; assess correlations with immune infiltration and immune-checkpoint genes; retain immune-relevant candidates (G_immune).
**(E) Biological plausibility:** Literature and pathway/GSEA support; checks across subsite, HPV/p16 and methylation; shortlist robust candidates.
**(F) Prioritization:** Candidates ranked by robustness and plausibility; **F2RL1** selected for in-depth analysis.
**(G) External patient validation:** IHC on FFPE/TMA (H-score 0–300) with ROC/Youden cut-point; survival by KM/Log-rank and multivariable Cox (prespecified covariates: age, sex, AJCC stage, grade, HPV/p16, smoking, alcohol, radiotherapy); complementary RT-qPCR in ≥30 paired tumor/adjacent tissues (2^-ΔΔCt).
**(H) Reporting & availability:** Report HR, 95% CI, P and BH-FDR; diagnostic AUC; figures/tables referenced; code, parameters and data pointers deposited in a public repository.

*Abbreviations:* TCGA-HNSCC, The Cancer Genome Atlas—Head and Neck Squamous Cell Carcinoma; GEO, Gene Expression Omnibus; OS/DSS, overall/disease-specific survival; KM, Kaplan–Meier; BH-FDR, Benjamini–Hochberg false discovery rate; ImmPort, Immunology Database and Analysis Portal; GSEA, gene-set enrichment analysis; FFPE, formalin-fixed paraffin-embedded; TMA, tissue microarray; ROC, receiver-operating characteristic; AUC, area under the curve; TPM, transcripts per million; H-score, semi-quantitative IHC score.


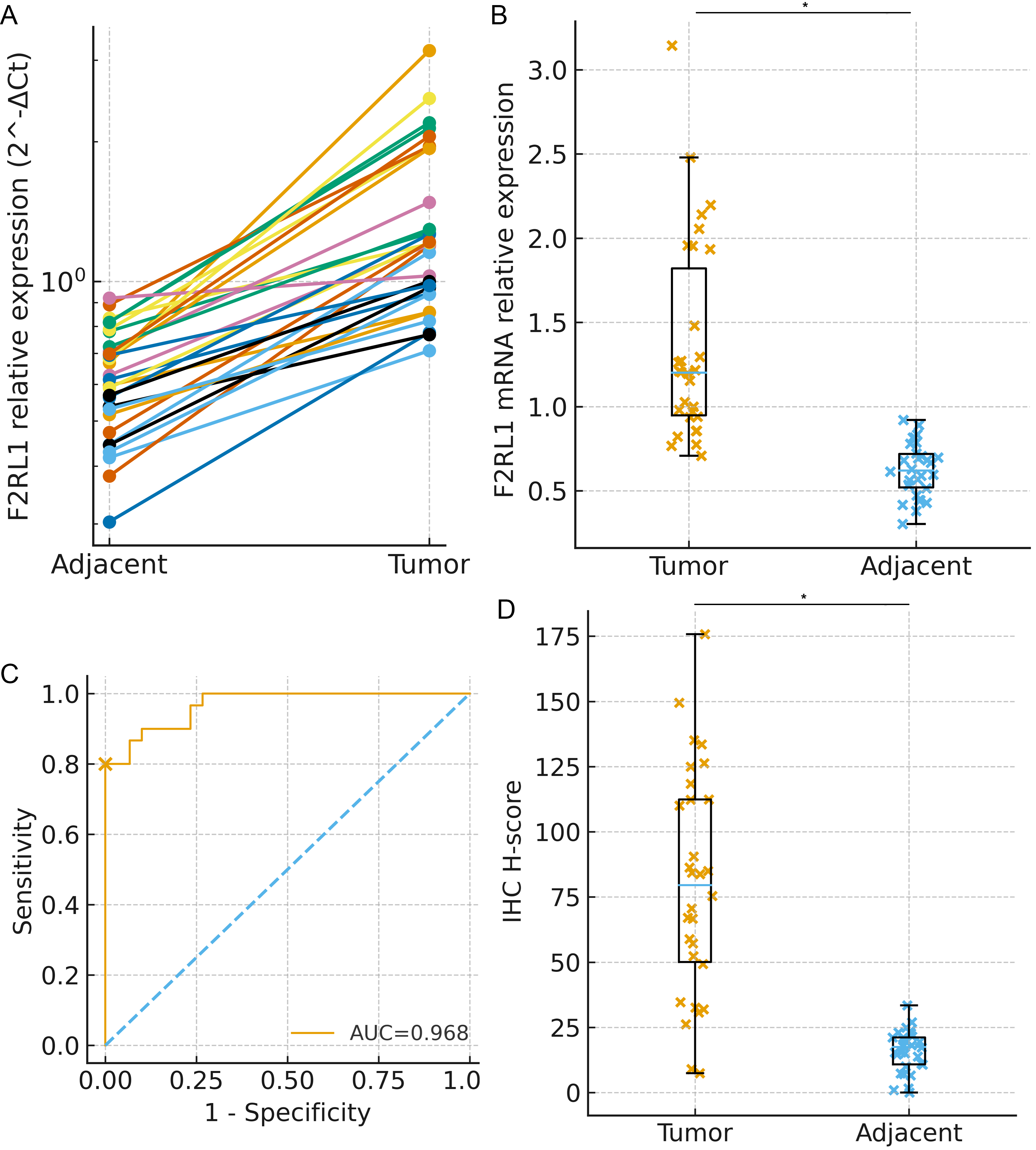


**Figure S2. External validation of F2RL1 in paired HNSCC tissues (n = 30 pairs).**
**(A)** Paired dot–line plot of F2RL1 mRNA relative expression measured by RT-qPCR (2^-ΔCt; y-axis on log scale). Each line links the adjacent non-tumor tissue to its matched tumor.
**(B)** Group summary (dot + box): center line = median, box = interquartile range (IQR), whiskers = 1.5×IQR, dots = individual samples. Significance assessed by two-sided Wilcoxon signed-rank test (* P < 0.05).
**(C)** ROC curve for discriminating tumor from adjacent tissue using 2^-ΔCt; the area under the curve (AUC) is shown in the panel.
**(D)** IHC H-score comparison between matched tumor and adjacent tissues (dot + box; annotations as in B); significance by Wilcoxon signed-rank test (* P < 0.05).


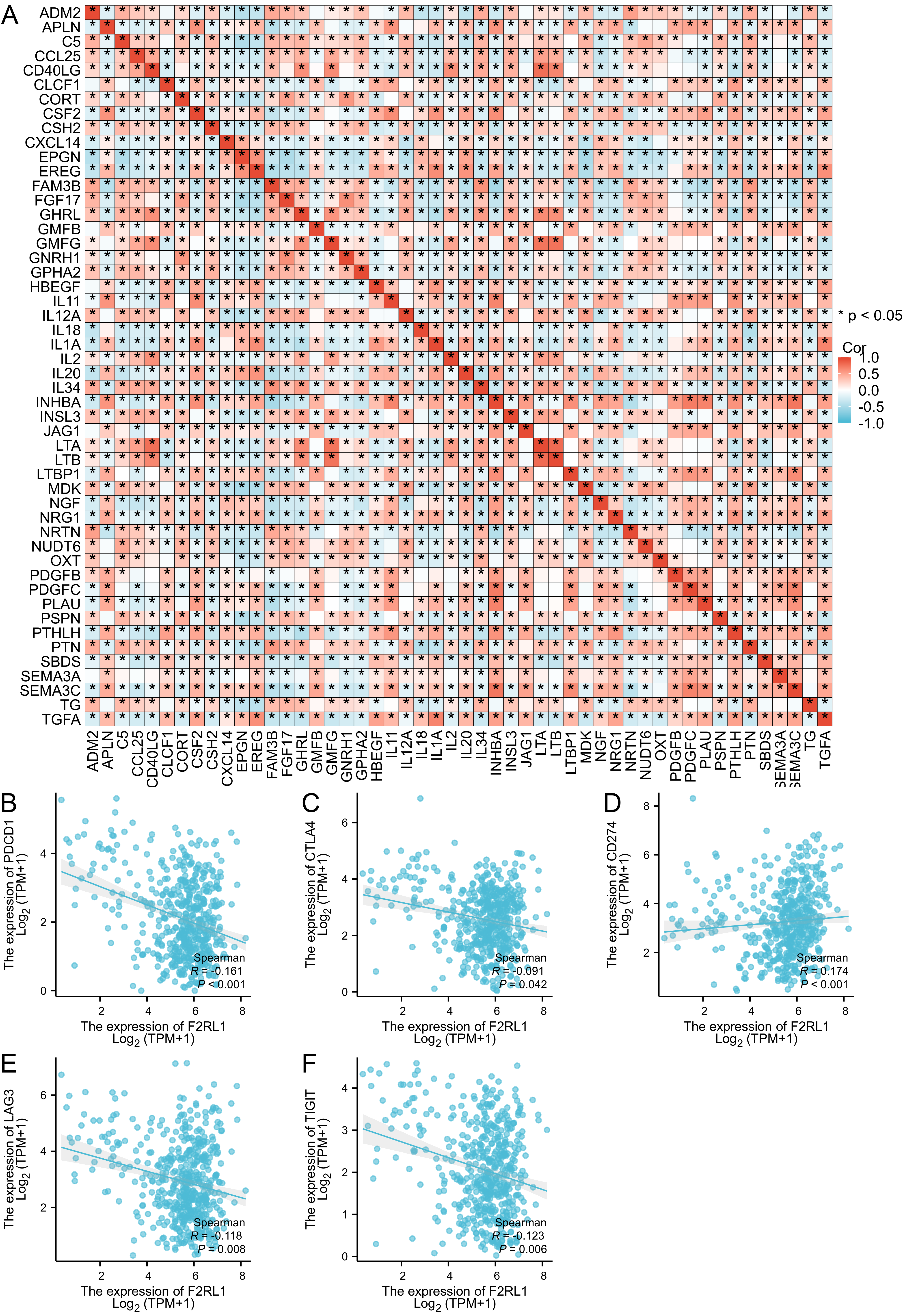


**Supplementary Figure S3. Auditable immune-correlation profiling of F2RL1 in HNSCC and benchmarking against canonical immune checkpoints.**
**(A)** Pairwise Spearman correlation heatmap among 50 immune-related genes that were identified as F2RL1-associated in TCGA-HNSC. Colors indicate correlation coefficients (ρ), and asterisks denote nominal significance (*P* < 0.05).
**(B–F)** Spearman correlation between F2RL1 expression and representative immune checkpoint genes in TCGA-HNSC tumour samples, including **PDCD1** (B), **CTLA4** (C), **CD274** (D), **LAG3** (E) and **TIGIT** (F). Gene expression was analysed as log2(TPM + 1). Each dot represents one tumour sample; the fitted trend line is shown with the shaded 95% confidence interval. Spearman ρ and *P* values are indicated in each panel.
