# Supplementary material for: Coagulation factor II receptor-like 1 as a prognostic and immuno-modulatory factor in head and neck squamous cell carcinoma
Source: PeerJ. 2026 Mar 18;14:e20970. doi: 10.7717/peerj.20970 (PMC13005615; doi:10.7717/peerj.20970)
Supplement: Supplemental Information 4 [file peerj-14-20970-s004.zip › GSE55547-reports.html]

仙桃-芯片-差异分析-在线分析报告


芯片-差异分析-在线分析报告

导出时间: 2025-12-14 01:06:59

目录

- 芯片-差异分析

- 样本信息

- 箱式图

- PCA图

- 差异统计

- 火山图

- 热图

- 方法学

芯片-差异分析

芯片-差异分析

**差异分析**: 基于表达谱数据数据进行两组差异分析

分析流程: limma包标准差异分析流程

页面中仅仅展示高表达(logFC为正)以及低表达(logFC为负)各30个的结果，更多的结果需要下载差异分析表格

| id | logFC | AveExpr | t | P.Value | adj.P.Val | B | anno |
| --- | --- | --- | --- | --- | --- | --- | --- |
| A\_23\_P1691 | 8.3426 | -1.0377 | 8.071 | 9.71e-08 | 0.0006670704 | 7.5769 | MMP1 |
| A\_23\_P161698 | 8.0402 | -1.218 | 6.8246 | 1.2e-06 | 0.0024108485 | 5.4095 | MMP3 |
| A\_33\_P3243887 | 7.5075 | -1.1872 | 7.4985 | 3e-07 | 0.0009511418 | 6.6132 | IL11 |
| A\_23\_P13094 | 6.8499 | 0.15221 | 5.0001 | 6.75e-05 | 0.0242919536 | 1.8002 | MMP10 |
| A\_33\_P3221203 | 6.6962 | -0.058469 | 3.7901 | 0.0011 | 0.0783111456 | -0.77373 | MMP13 |
| A\_23\_P431388 | 6.5652 | -1.171 | 7.2944 | 4.53e-07 | 0.0012773347 | 6.2565 | SPOCD1 |
| A\_23\_P2271 | 6.16 | -0.78285 | 5.5557 | 1.9e-05 | 0.0134484587 | 2.9475 | PTHLH |
| A\_33\_P3806965 | 6.1044 | -0.49268 | 3.4873 | 0.0023 | 0.1043590364 | -1.4154 | FLJ13744 |
| A\_23\_P92909 | 6.0713 | -0.63699 | 3.3489 | 0.0032 | 0.1166661863 | -1.7054 | SPINK6 |
| A\_23\_P7313 | 6.0192 | -0.86327 | 6.5754 | 2.03e-06 | 0.0034273690 | 4.9456 | SPP1 |
| A\_23\_P64808 | 5.8287 | -0.94451 | 8.3586 | 5.61e-08 | 0.0004740203 | 8.0407 | HOXC13 |
| A\_23\_P122924 | 5.7482 | -0.029299 | 5.4102 | 2.64e-05 | 0.0165410372 | 2.6504 | INHBA |
| A\_21\_P0013198 | 5.4841 | -1.9248 | 2.3151 | 0.0313 | 0.2697993346 | -3.74 | PSPHP1 |
| A\_23\_P157793 | 5.3282 | -1.0297 | 3.9456 | 0.0008 | 0.0673340863 | -0.44192 | CA9 |
| A\_32\_P189781 | 5.2934 | -0.42316 | 4.416 | 0.0003 | 0.0438539050 | 0.56382 | C14orf34 |
| A\_33\_P3232692 | 5.1407 | -0.18947 | 3.7779 | 0.0012 | 0.0795631133 | -0.79972 | IL24 |
| A\_23\_P43197 | 5.0847 | 0.71533 | 2.7109 | 0.0134 | 0.1914311492 | -2.9974 | CALB1 |
| A\_33\_P3304668 | 5.0705 | -0.45777 | 5.5537 | 1.91e-05 | 0.0134484587 | 2.9435 | COL1A1 |
| A\_23\_P150316 | 5.0083 | 0.012522 | 3.4834 | 0.0023 | 0.1048738854 | -1.4235 | MMP12 |
| A\_21\_P0002733 | 4.9171 | 0.68211 | 2.5795 | 0.0178 | 0.2159851074 | -3.2501 | XLOC\_002603 |
| A\_21\_P0011578 | 4.8674 | -0.64197 | 7.5937 | 2.48e-07 | 0.0008984868 | 6.7772 | XLOC\_l2\_006021 |
| A\_24\_P810290 | 4.8177 | -0.25124 | 4.5458 | 0.0002 | 0.0387272930 | 0.84029 | PPAPDC1A |
| A\_33\_P3342375 | 4.811 | 2.7021 | 1.9656 | 0.0633 | 0.3626331083 | -4.3404 | MAGEA6 |
| A\_33\_P3334423 | 4.7587 | 0.15873 | 2.5054 | 0.0209 | 0.2295572877 | -3.3902 | SPRR2G |
| A\_23\_P350396 | 4.7058 | -0.2215 | 2.5273 | 0.0200 | 0.2263407633 | -3.349 | CDSN |
| A\_21\_P0014892 | 4.6869 | -0.41516 | 5.5005 | 2.15e-05 | 0.0145370831 | 2.8352 | LOC100506027 |
| A\_33\_P3511265 | 4.6324 | 0.20157 | 3.9562 | 0.0008 | 0.0668550089 | -0.41922 | POSTN |
| A\_24\_P280274 | 4.5454 | 0.18552 | 3.1847 | 0.0046 | 0.1310040203 | -2.046 | S100A7A |
| A\_33\_P3379396 | 4.5321 | 0.18641 | 2.5983 | 0.0171 | 0.2123155485 | -3.2144 | KRT1 |
| A\_23\_P62081 | 4.5044 | -0.29498 | 6.1659 | 4.9e-06 | 0.0057821518 | 4.1618 | SCG5 |
| A\_33\_P3318097 | -7.5288 | 0.10839 | -3.7622 | 0.0012 | 0.0800264630 | -0.83313 | TMPRSS11B |
| A\_23\_P115202 | -7.0073 | 0.22154 | -3.4685 | 0.0024 | 0.1050618072 | -1.4549 | CRNN |
| A\_33\_P3275035 | -6.775 | 0.34682 | -3.9073 | 0.0009 | 0.0702484440 | -0.52384 | MUC21 |
| A\_23\_P2674 | -6.1484 | 0.48433 | -3.2179 | 0.0043 | 0.1267981884 | -1.9775 | KRT4 |
| A\_23\_P17134 | -6.1284 | 0.77378 | -4.0798 | 0.0006 | 0.0598255522 | -0.15498 | MAL |
| A\_23\_P23611 | -5.5672 | 0.91062 | -7.7374 | 1.86e-07 | 0.0007880126 | 7.0219 | AMY1C |
| A\_32\_P173662 | -5.3978 | 1.4508 | -5.1304 | 5e-05 | 0.0212153727 | 2.0723 | CRISP2 |
| A\_23\_P103617 | -5.3011 | 1.159 | -4.354 | 0.0003 | 0.0468221554 | 0.43134 | ANXA9 |
| A\_33\_P3410806 | -5.1938 | 1.2705 | -3.2902 | 0.0036 | 0.1200700672 | -1.8278 | CLDN10 |
| A\_23\_P166269 | -5.034 | 0.014334 | -4.0129 | 0.0007 | 0.0624007013 | -0.298 | FAM3B |
| A\_33\_P3335735 | -5.0336 | 0.96497 | -3.0753 | 0.0059 | 0.1424698009 | -2.2702 | LOC100128977 |
| A\_21\_P0008432 | -4.8878 | 1.1212 | -5.806 | 1.08e-05 | 0.0100033448 | 3.4519 | XLOC\_011012 |
| A\_33\_P3217845 | -4.8703 | -0.86088 | -3.176 | 0.0047 | 0.1313486154 | -2.064 | TMPRSS11A |
| A\_21\_P0008881 | -4.7942 | 1.5093 | -4.4726 | 0.0002 | 0.0421344619 | 0.68442 | LOC100507221 |
| A\_33\_P3315268 | -4.7035 | 0.64148 | -4.1299 | 0.0005 | 0.0576131258 | -0.047743 | KRT78 |
| A\_33\_P3358208 | -4.6232 | 0.024299 | -3.6491 | 0.0016 | 0.0893338365 | -1.0734 | PADI1 |
| A\_21\_P0006276 | -4.5369 | -0.14306 | -3.4682 | 0.0024 | 0.1050618072 | -1.4556 | XLOC\_007734 |
| A\_33\_P3356935 | -4.4757 | 1.6455 | -3.1902 | 0.0046 | 0.1301593629 | -2.0346 | C11orf92 |
| A\_23\_P166848 | -4.474 | 1.6768 | -2.4377 | 0.0242 | 0.2437252046 | -3.5163 | LTF |
| A\_21\_P0000806 | -4.4525 | 1.1369 | -6.4296 | 2.77e-06 | 0.0039567997 | 4.6695 | LOC100128590 |
| A\_23\_P45751 | -4.4469 | -0.56785 | -3.0541 | 0.0062 | 0.1437854190 | -2.3135 | CLCA4 |
| A\_23\_P41145 | -4.3797 | -0.24896 | -2.9734 | 0.0075 | 0.1536622246 | -2.477 | FAM3D |
| A\_24\_P228149 | -4.3623 | -0.45511 | -2.7023 | 0.0137 | 0.1928555290 | -3.0141 | KRT13 |
| A\_33\_P3285565 | -4.3544 | -0.32421 | -2.9123 | 0.0086 | 0.1618828142 | -2.5997 | CLDN3 |
| A\_23\_P66798 | -4.3365 | -2.2662 | -1.6904 | 0.1064 | 0.4465577079 | -4.7669 | KRT19 |
| A\_23\_P36531 | -4.3066 | 0.55216 | -3.7756 | 0.0012 | 0.0796655535 | -0.80454 | TSPAN8 |
| A\_21\_P0003132 | -4.2998 | 0.5577 | -4.2462 | 0.0004 | 0.0518448701 | 0.20099 | XLOC\_003167 |
| A\_33\_P3265783 | -4.2992 | 0.80668 | -2.515 | 0.0205 | 0.2283983870 | -3.3721 | STATH |
| A\_23\_P258887 | -4.2802 | 0.11554 | -3.6333 | 0.0016 | 0.0910217454 | -1.107 | ALDH1L1 |
| A\_23\_P500093 | -4.2643 | 0.75182 | -4.3933 | 0.0003 | 0.0452082155 | 0.51537 | RGS13 |

下载-差异分析.xlsx

样本信息

差异分析参考组: ref

| 组别 | 数量 |
| --- | --- |
| ref | 4 |
| test | 16 |

箱式图

**箱式图**: 用箱子绘制每个样本对应的数据情况，可用于查看样本校正情况

· 箱子中间的横线代表中位数，箱子的上边代表上四分位，箱子的下边代表下四分位

· 如果箱子上下存在有黑点，代表此样本存在有离群值

· 一般只要关注各个样本中位数的线 是否在同一个水平线上即可(如果是，则代表样本已经校正好)

PCA图

**PCA图**: 对高纬度数据进行降维后查看样本间差异情况

横坐标代表PCA降维后第1个主成分，纵坐标代表PCA降维后的第2个主成分，括号内代表主成分解释的比例

差异统计

差异分析后一些常见阈值(|logFC|大于2或者1或者是0.58(0.58换算过来就是1.5倍))下的差异分子数量, 也可以根据需要下载差异分析结果用excel表进行过滤

| 筛选条件 | 筛选后的数量 |
| --- | --- |
| |LogFC|>2 & p.adj<0.05 | 153 |
| |LogFC|>1 & p.adj<0.05 | 242 |
| |LogFC|>0.58 & p.adj<0.05 | 254 |

火山图

**火山图**: 可视化差异分析的结果

**阈值**: logFC(1) | p.adj(0.05)

图中横坐标代表logFC，纵坐标代表p值或者校正后p值

热图

**热图**: 热图主要由一个个不同颜色(深度)的方块组成，每个方块表示行列所对应的数值

**作用**: 主要用于可视化差异表达矩阵情况, 可以从差异分析中挑选差异表达的分子或者ID输入到第一个数据参数框中对数据进行可视化

**补充说明**

· 如果想要调整(列)样本的顺序，可以选择不同的聚类方法或者不对列进行聚类

方法学

**软件**: R (4.2.1)版本

**R包**: GEOquery[2.64.2], limma[3.52.2], ggplot2[3.4.4], ComplexHeatmap[2.13.1]

**补充说明:**

· 数据获取: 通过GEOquery包从GEO数据库中下载GSE55547

· 校正处理: 通过limma包的normalizeBetweenArrays函数再次标准化数据

· 注释处理: 去除掉一个探针对应多个分子的探针；当遇到对应同一个分子的探针时，仅保留信号值最大的探针

· 可视化: 通过箱式图查看查看样本情况, 通过PCA图查看样本分组间聚类情况, 利用limma包进行两组的差异分析, 差异分析结果用火山图进行可视化，同时对显著表达的分子用热图形式进行可视化

**参考文献:**

Davis, Sean, and Paul S. Meltzer. GEOquery: a bridge between the Gene Expression Omnibus (GEO) and BioConductor. Bioinformatics 23.14 (2007): 1846-1847.文献链接

Smyth, Gordon K. Limma: linear models for microarray data. Bioinformatics and computational biology solutions using R and Bioconductor. Springer, New York, NY, 2005. 397-420.文献链接

Gu, Zuguang, Roland Eils, and Matthias Schlesner. Complex heatmaps reveal patterns and correlations in multidimensional genomic data. Bioinformatics 32.18 (2016): 2847-2849.文献链接
