# Supplementary material for: Coagulation factor II receptor-like 1 as a prognostic and immuno-modulatory factor in head and neck squamous cell carcinoma
Source: PeerJ. 2026 Mar 18;14:e20970. doi: 10.7717/peerj.20970 (PMC13005615; doi:10.7717/peerj.20970)
Supplement: Supplemental Information 4 [file peerj-14-20970-s004.zip › GSE55548-reports.html]

仙桃-芯片-差异分析-在线分析报告


芯片-差异分析-在线分析报告

导出时间: 2025-12-14 01:07:06

目录

- 芯片-差异分析

- 样本信息

- 箱式图

- PCA图

- 差异统计

- 火山图

- 热图

- 方法学

芯片-差异分析

芯片-差异分析

**差异分析**: 基于表达谱数据数据进行两组差异分析

分析流程: limma包标准差异分析流程

页面中仅仅展示高表达(logFC为正)以及低表达(logFC为负)各30个的结果，更多的结果需要下载差异分析表格

| id | logFC | AveExpr | t | P.Value | adj.P.Val | B | anno |
| --- | --- | --- | --- | --- | --- | --- | --- |
| A\_23\_P1691 | 6.5486 | 1.7834 | 3.0363 | 0.0177 | 0.6812893 | -2.8477 | MMP1 |
| A\_23\_P161698 | 6.4049 | 0.72129 | 2.6146 | 0.0330 | 0.7126803 | -3.3901 | MMP3 |
| A\_23\_P92730 | 5.9615 | -0.29089 | 2.2379 | 0.0581 | 0.7489557 | -3.8827 | HSPB3 |
| A\_33\_P3379396 | 5.9313 | 1.6824 | 3.0007 | 0.0186 | 0.6812893 | -2.8928 | KRT1 |
| A\_33\_P3221203 | 5.7889 | 2.8293 | 2.5999 | 0.0337 | 0.7126803 | -3.4093 | MMP13 |
| A\_23\_P49657 | 5.2551 | 0.9537 | 2.1484 | 0.0666 | 0.7611076 | -3.9995 | MYH1 |
| A\_24\_P185945 | 4.9792 | 2.2386 | 2.5702 | 0.0352 | 0.7133789 | -3.448 | MAGEA4 |
| A\_24\_P191326 | 4.8331 | -0.74139 | 1.6798 | 0.1345 | 0.7949929 | -4.5934 | MYL1 |
| A\_33\_P3304668 | 4.7243 | 0.65708 | 6.2892 | 0.0003 | 0.2865316 | 0.34234 | COL1A1 |
| A\_23\_P24469 | 4.6111 | -0.062591 | 1.7425 | 0.1225 | 0.7932625 | -4.5165 | CSRP3 |
| A\_24\_P280274 | 4.5768 | 1.4688 | 2.4659 | 0.0412 | 0.7216947 | -3.5844 | S100A7A |
| A\_23\_P64808 | 4.5724 | 2.2157 | 3.2815 | 0.0124 | 0.6380512 | -2.5419 | HOXC13 |
| A\_23\_P86975 | 4.5714 | 1.2779 | 3.7128 | 0.0068 | 0.5503430 | -2.0268 | CARD18 |
| A\_23\_P432947 | 4.5426 | -0.43071 | 4.8865 | 0.0015 | 0.4063094 | -0.79891 | GREM1 |
| A\_23\_P50250 | 4.4882 | -0.42522 | 1.5266 | 0.1683 | 0.8085154 | -4.7766 | CKM |
| A\_23\_P144326 | 4.4858 | 0.31487 | 2.077 | 0.0742 | 0.7665113 | -4.0921 | ASB5 |
| A\_23\_P431388 | 4.3851 | 2.0919 | 2.9972 | 0.0187 | 0.6813974 | -2.8971 | SPOCD1 |
| A\_24\_P264943 | 4.3807 | 0.50643 | 2.663 | 0.0307 | 0.7072433 | -3.3271 | COMP |
| A\_33\_P3342375 | 4.3791 | 2.1644 | 1.8598 | 0.1029 | 0.7886851 | -4.37 | MAGEA6 |
| A\_23\_P7313 | 4.3732 | 0.39833 | 2.7627 | 0.0264 | 0.6928931 | -3.1977 | SPP1 |
| A\_23\_P17190 | 4.3732 | -0.12448 | 1.7014 | 0.1302 | 0.7945326 | -4.567 | KBTBD10 |
| A\_21\_P0002733 | 4.3481 | 1.9003 | 2.8819 | 0.0221 | 0.6813974 | -3.0442 | XLOC\_002603 |
| A\_23\_P253542 | 4.327 | -0.48938 | 1.7164 | 0.1274 | 0.7932625 | -4.5486 | SMPX |
| A\_24\_P349117 | 4.3029 | 2.0855 | 2.7852 | 0.0256 | 0.6887294 | -3.1687 | GPR158 |
| A\_33\_P3335177 | 4.2759 | 1.5815 | 3.3886 | 0.0106 | 0.6252127 | -2.411 | SFRP4 |
| A\_33\_P3511265 | 4.2689 | 0.13073 | 2.9319 | 0.0206 | 0.6813974 | -2.9803 | POSTN |
| A\_24\_P250922 | 4.2409 | 0.17993 | 10.062 | 1.38e-05 | 0.1562457 | 2.175 | PTGS2 |
| A\_23\_P76460 | 4.218 | 0.87859 | 1.8213 | 0.1090 | 0.7886851 | -4.4184 | MYF6 |
| A\_33\_P3252048 | 4.2132 | 1.2628 | 2.4078 | 0.0450 | 0.7244394 | -3.6604 | PPP1R27 |
| A\_23\_P148737 | 4.1954 | 1.046 | 2.4081 | 0.0450 | 0.7244394 | -3.6601 | MYBPH |
| A\_33\_P3275035 | -9.3111 | -0.67385 | -5.2648 | 0.0010 | 0.3918345 | -0.45809 | MUC21 |
| A\_33\_P3318097 | -8.0629 | -0.6167 | -8.8099 | 3.45e-05 | 0.1562457 | 1.7152 | TMPRSS11B |
| A\_23\_P2674 | -7.064 | -0.41066 | -9.4814 | 2.08e-05 | 0.1562457 | 1.9759 | KRT4 |
| A\_21\_P0006276 | -6.9896 | 0.19544 | -8.9885 | 3.01e-05 | 0.1562457 | 1.788 | XLOC\_007734 |
| A\_23\_P259314 | -6.956 | 3.427 | -3.1672 | 0.0146 | 0.6670601 | -2.6833 | RPS4Y1 |
| A\_23\_P324384 | -6.7802 | 3.3588 | -3.2737 | 0.0125 | 0.6412591 | -2.5514 | RPS4Y2 |
| A\_23\_P115202 | -6.7493 | -0.43967 | -6.4541 | 0.0003 | 0.2756167 | 0.45593 | CRNN |
| A\_33\_P3358208 | -6.5207 | -1.2201 | -4.1458 | 0.0038 | 0.4605943 | -1.5435 | PADI1 |
| A\_23\_P17134 | -6.1846 | -0.80221 | -4.9644 | 0.0014 | 0.4063094 | -0.72661 | MAL |
| A\_33\_P3217845 | -6.176 | -0.83383 | -5.1938 | 0.0011 | 0.3973824 | -0.52015 | TMPRSS11A |
| A\_23\_P57118 | -6.1391 | -1.8794 | -3.0408 | 0.0175 | 0.6812893 | -2.842 | TGM3 |
| A\_33\_P3315268 | -5.9606 | -0.6076 | -5.6048 | 0.0007 | 0.3252150 | -0.17337 | KRT78 |
| A\_33\_P3381378 | -5.8606 | 0.66391 | -2.7027 | 0.0289 | 0.6997576 | -3.2754 | PAX1 |
| A\_33\_P3224331 | -5.688 | 2.8282 | -3.1078 | 0.0159 | 0.6735995 | -2.7576 | DDX3Y |
| A\_33\_P3335735 | -5.6628 | -1.3932 | -3.4185 | 0.0102 | 0.6148207 | -2.3749 | LOC100128977 |
| A\_33\_P3403474 | -5.5539 | -0.48682 | -7.0095 | 0.0002 | 0.2545427 | 0.81081 | TMPRSS11BNL |
| A\_33\_P3365193 | -5.5102 | 0.059865 | -4.2822 | 0.0032 | 0.4493507 | -1.3985 | AMY1C |
| A\_21\_P0012777 | -5.5075 | 0.20981 | -6.0119 | 0.0004 | 0.3061371 | 0.14223 | XLOC\_l2\_011146 |
| A\_21\_P0008881 | -5.5037 | 0.38058 | -4.9623 | 0.0014 | 0.4063094 | -0.72854 | LOC100507221 |
| A\_23\_P94186 | -5.469 | -0.60496 | -2.9631 | 0.0197 | 0.6813974 | -2.9405 | LYPD2 |
| A\_24\_P228149 | -5.3859 | -0.5219 | -4.3274 | 0.0030 | 0.4487403 | -1.3513 | KRT13 |
| A\_21\_P0008432 | -5.3436 | 2.0855 | -3.158 | 0.0148 | 0.6670601 | -2.6948 | XLOC\_011012 |
| A\_23\_P45751 | -5.2901 | 0.4668 | -4.5 | 0.0024 | 0.4293928 | -1.1744 | CLCA4 |
| A\_33\_P3217700 | -5.2733 | 2.5928 | -3.0766 | 0.0167 | 0.6769307 | -2.7968 | USP9Y |
| A\_21\_P0000806 | -5.2456 | 0.0086532 | -6.8112 | 0.0002 | 0.2545427 | 0.68881 | LOC100128590 |
| A\_23\_P151975 | -5.2044 | -0.99048 | -4.5696 | 0.0022 | 0.4272155 | -1.1047 | RHCG |
| A\_21\_P0006594 | -5.1574 | 2.3648 | -2.9051 | 0.0214 | 0.6813974 | -3.0145 | TTTY15 |
| A\_33\_P3401295 | -5.1515 | -1.4271 | -2.2631 | 0.0560 | 0.7450842 | -3.8499 | CRCT1 |
| A\_23\_P77529 | -5.1197 | -0.68163 | -4.5175 | 0.0024 | 0.4293928 | -1.1568 | MSLN |
| A\_23\_P71379 | -5.1053 | -0.57629 | -3.4472 | 0.0098 | 0.6073288 | -2.3402 | PSCA |

下载-差异分析.xlsx

样本信息

差异分析参考组: ref

| 组别 | 数量 |
| --- | --- |
| ref | 4 |
| test | 4 |

箱式图

**箱式图**: 用箱子绘制每个样本对应的数据情况，可用于查看样本校正情况

· 箱子中间的横线代表中位数，箱子的上边代表上四分位，箱子的下边代表下四分位

· 如果箱子上下存在有黑点，代表此样本存在有离群值

· 一般只要关注各个样本中位数的线 是否在同一个水平线上即可(如果是，则代表样本已经校正好)

PCA图

**PCA图**: 对高纬度数据进行降维后查看样本间差异情况

横坐标代表PCA降维后第1个主成分，纵坐标代表PCA降维后的第2个主成分，括号内代表主成分解释的比例

差异统计

差异分析后一些常见阈值(|logFC|大于2或者1或者是0.58(0.58换算过来就是1.5倍))下的差异分子数量, 也可以根据需要下载差异分析结果用excel表进行过滤

当校正后p值均不满足&lt;0.05时会考虑使用p值来作为统计

| 筛选条件 | 筛选后的数量 |
| --- | --- |
| |LogFC|>2 & pvalue<0.05 | 533 |
| |LogFC|>1 & pvalue<0.05 | 1445 |
| |LogFC|>0.58 & pvalue<0.05 | 2047 |

火山图

**火山图**: 可视化差异分析的结果

**阈值**: logFC(1) | pvalue(0.05)

图中横坐标代表logFC，纵坐标代表p值或者校正后p值

热图

**热图**: 热图主要由一个个不同颜色(深度)的方块组成，每个方块表示行列所对应的数值

**作用**: 主要用于可视化差异表达矩阵情况, 可以从差异分析中挑选差异表达的分子或者ID输入到第一个数据参数框中对数据进行可视化

**补充说明**

· 如果想要调整(列)样本的顺序，可以选择不同的聚类方法或者不对列进行聚类

方法学

**软件**: R (4.2.1)版本

**R包**: GEOquery[2.64.2], limma[3.52.2], ggplot2[3.4.4], ComplexHeatmap[2.13.1]

**补充说明:**

· 数据获取: 通过GEOquery包从GEO数据库中下载GSE55548

· 校正处理: 通过limma包的normalizeBetweenArrays函数再次标准化数据

· 注释处理: 去除掉一个探针对应多个分子的探针；当遇到对应同一个分子的探针时，仅保留信号值最大的探针

· 可视化: 通过箱式图查看查看样本情况, 通过PCA图查看样本分组间聚类情况, 利用limma包进行两组的差异分析, 差异分析结果用火山图进行可视化，同时对显著表达的分子用热图形式进行可视化

**参考文献:**

Davis, Sean, and Paul S. Meltzer. GEOquery: a bridge between the Gene Expression Omnibus (GEO) and BioConductor. Bioinformatics 23.14 (2007): 1846-1847.文献链接

Smyth, Gordon K. Limma: linear models for microarray data. Bioinformatics and computational biology solutions using R and Bioconductor. Springer, New York, NY, 2005. 397-420.文献链接

Gu, Zuguang, Roland Eils, and Matthias Schlesner. Complex heatmaps reveal patterns and correlations in multidimensional genomic data. Bioinformatics 32.18 (2016): 2847-2849.文献链接
