# Supplementary material for: Coagulation factor II receptor-like 1 as a prognostic and immuno-modulatory factor in head and neck squamous cell carcinoma
Source: PeerJ. 2026 Mar 18;14:e20970. doi: 10.7717/peerj.20970 (PMC13005615; doi:10.7717/peerj.20970)
Supplement: Supplemental Information 4 [file peerj-14-20970-s004.zip › GSE9844-reports.html]

仙桃-芯片-差异分析-在线分析报告


芯片-差异分析-在线分析报告

导出时间: 2025-12-14 01:07:12

目录

- 芯片-差异分析

- 样本信息

- 箱式图

- PCA图

- 差异统计

- 火山图

- 热图

- 方法学

芯片-差异分析

芯片-差异分析

**差异分析**: 基于表达谱数据数据进行两组差异分析

分析流程: limma包标准差异分析流程

页面中仅仅展示高表达(logFC为正)以及低表达(logFC为负)各30个的结果，更多的结果需要下载差异分析表格

| id | logFC | AveExpr | t | P.Value | adj.P.Val | B | anno |
| --- | --- | --- | --- | --- | --- | --- | --- |
| 204475\_at | 7.0002 | 8.8043 | 13.656 | 2.93e-16 | 1.603116e-11 | 25.335 | MMP1 |
| 211756\_at | 4.1332 | 6.9997 | 8.0528 | 9.51e-10 | 5.047029e-06 | 12.035 | PTHLH |
| 202267\_at | 3.7074 | 7.2545 | 6.8526 | 3.84e-08 | 4.767094e-05 | 8.607 | LAMC2 |
| 204470\_at | 3.4237 | 7.6379 | 7.1094 | 1.72e-08 | 3.035892e-05 | 9.3529 | CXCL1 |
| 205828\_at | 3.4139 | 7.552 | 7.4089 | 6.8e-09 | 1.690483e-05 | 10.215 | MMP3 |
| 230193\_at | 3.3847 | 7.4665 | 6.8552 | 3.81e-08 | 4.767094e-05 | 8.6146 | WDR66 |
| 237732\_at | 3.3643 | 6.759 | 3.8936 | 0.0004 | 1.561082e-02 | 0.0063204 | PRR9 |
| 202859\_x\_at | 3.2805 | 8.6117 | 7.9777 | 1.19e-09 | 5.047029e-06 | 11.825 | CXCL8 |
| 210511\_s\_at | 3.1988 | 6.5287 | 4.9405 | 1.59e-05 | 2.077241e-03 | 2.9745 | INHBA |
| 204415\_at | 3.1368 | 7.924 | 5.8696 | 8.51e-07 | 3.152783e-04 | 5.7129 | IFI6 |
| 232170\_at | 3.0957 | 7.14 | 4.6679 | 3.7e-05 | 3.468292e-03 | 2.1834 | S100A7A |
| 232165\_at | 3.0494 | 9.6283 | 5.9094 | 7.5e-07 | 2.887965e-04 | 5.8308 | EPPK1 |
| 227174\_at | 2.9559 | 6.0193 | 4.1262 | 0.0002 | 1.007229e-02 | 0.64668 | WDR72 |
| 203413\_at | 2.948 | 7.1529 | 5.4026 | 3.72e-06 | 7.979073e-04 | 4.3319 | NELL2 |
| 220322\_at | 2.868 | 6.5635 | 4.8193 | 2.32e-05 | 2.622448e-03 | 2.6217 | IL36G |
| 213992\_at | 2.8306 | 6.3977 | 7.5822 | 3.99e-09 | 1.211909e-05 | 10.71 | COL4A6 |
| 204580\_at | 2.826 | 6.9522 | 5.6393 | 1.76e-06 | 5.257821e-04 | 5.0314 | MMP12 |
| 206569\_at | 2.8034 | 6.315 | 4.9778 | 1.41e-05 | 1.934944e-03 | 3.0834 | IL24 |
| 1555786\_s\_at | 2.7555 | 6.6647 | 5.0464 | 1.14e-05 | 1.694556e-03 | 3.284 | LINC00520 |
| 242625\_at | 2.7405 | 6.6268 | 4.4768 | 6.67e-05 | 5.003717e-03 | 1.6351 | RSAD2 |
| 205680\_at | 2.6451 | 5.0119 | 4.3207 | 0.0001 | 6.825042e-03 | 1.192 | MMP10 |
| 201839\_s\_at | 2.6055 | 6.6487 | 4.4684 | 6.84e-05 | 5.055768e-03 | 1.6112 | EPCAM |
| 205916\_at | 2.5295 | 10.756 | 2.7021 | 0.0102 | 1.131191e-01 | -2.9757 | S100A7 |
| 231867\_at | 2.5274 | 8.9929 | 5.2297 | 6.42e-06 | 1.109982e-03 | 3.8223 | TENM2 |
| 211980\_at | 2.4831 | 9.0501 | 6.2025 | 2.97e-07 | 1.803032e-04 | 6.6977 | COL4A1 |
| 205483\_s\_at | 2.4759 | 8.7116 | 4.8908 | 1.85e-05 | 2.288439e-03 | 2.8297 | ISG15 |
| 230835\_at | 2.4663 | 9.8233 | 2.7975 | 0.0080 | 9.832400e-02 | -2.7604 | KRTDAP |
| 214612\_x\_at | 2.4259 | 5.212 | 3.051 | 0.0041 | 6.726836e-02 | -2.1655 | MAGEA6 |
| 203256\_at | 2.4029 | 7.1925 | 7.3483 | 8.2e-09 | 1.868695e-05 | 10.041 | CDH3 |
| 212365\_at | 2.3642 | 7.2405 | 7.9074 | 1.48e-09 | 5.047029e-06 | 11.629 | MYO1B |
| 1560712\_at | -5.6438 | 8.6689 | -6.933 | 2.98e-08 | 4.183623e-05 | 8.8409 | TMPRSS11B |
| 213240\_s\_at | -5.1814 | 10.529 | -5.9588 | 6.42e-07 | 2.617747e-04 | 5.9769 | KRT4 |
| 204777\_s\_at | -4.9596 | 9.4867 | -5.48 | 2.92e-06 | 6.991957e-04 | 4.5602 | MAL |
| 220090\_at | -4.8476 | 9.3484 | -4.6515 | 3.9e-05 | 3.569145e-03 | 2.1361 | CRNN |
| 207935\_s\_at | -4.651 | 9.2814 | -4.6715 | 3.66e-05 | 3.465666e-03 | 2.1937 | KRT13 |
| 204631\_at | -3.9816 | 5.3874 | -3.543 | 0.0011 | 2.982237e-02 | -0.92902 | MYH2 |
| 209612\_s\_at | -3.8318 | 4.6592 | -8.0174 | 1.06e-09 | 5.047029e-06 | 11.936 | ADH1B |
| 220026\_at | -3.769 | 6.2201 | -5.4999 | 2.74e-06 | 6.712112e-04 | 4.6192 | CLCA4 |
| 222043\_at | -3.4348 | 6.7603 | -6.2387 | 2.65e-07 | 1.645046e-04 | 6.8044 | CLU |
| 230418\_s\_at | -3.4192 | 5.0853 | -6.1108 | 3.97e-07 | 2.008017e-04 | 6.4266 | GALNT16 |
| 213371\_at | -3.3608 | 5.9794 | -4.3199 | 0.0001 | 6.834410e-03 | 1.1896 | LDB3 |
| 206884\_s\_at | -3.294 | 7.4115 | -5.0241 | 1.22e-05 | 1.764914e-03 | 3.2189 | SCEL |
| 203914\_x\_at | -3.2868 | 6.641 | -7.9137 | 1.45e-09 | 5.047029e-06 | 11.646 | HPGD |
| 210096\_at | -3.28 | 5.7437 | -6.0043 | 5.55e-07 | 2.429653e-04 | 6.1117 | CYP4B1 |
| 207802\_at | -3.2007 | 5.5261 | -3.4674 | 0.0013 | 3.391239e-02 | -1.1251 | CRISP3 |
| 204719\_at | -3.156 | 5.0831 | -6.64 | 7.47e-08 | 7.365671e-05 | 7.9854 | ABCA8 |
| 227194\_at | -3.1315 | 7.3056 | -3.9136 | 0.0004 | 1.501550e-02 | 0.060945 | FAM3B |
| 205185\_at | -3.124 | 10.46 | -4.7114 | 3.24e-05 | 3.242233e-03 | 2.3091 | SPINK5 |
| 206605\_at | -2.9264 | 6.164 | -5.416 | 3.57e-06 | 7.841608e-04 | 4.3712 | ENDOU |
| 204284\_at | -2.9231 | 6.9168 | -5.1383 | 8.55e-06 | 1.362245e-03 | 3.5534 | PPP1R3C |
| 206199\_at | -2.9121 | 6.1597 | -4.5673 | 5.05e-05 | 4.128736e-03 | 1.894 | CEACAM7 |
| 209763\_at | -2.8715 | 5.1109 | -5.4304 | 3.41e-06 | 7.766454e-04 | 4.4139 | CHRDL1 |
| 219106\_s\_at | -2.8355 | 4.5023 | -3.1282 | 0.0034 | 5.934867e-02 | -1.9783 | KLHL41 |
| 1559607\_s\_at | -2.7664 | 9.5634 | -4.6101 | 4.43e-05 | 3.818739e-03 | 2.0169 | GBP6 |
| 1553212\_at | -2.6811 | 6.0616 | -6.4342 | 1.43e-07 | 1.148540e-04 | 7.3809 | KRT78 |
| 223395\_at | -2.6224 | 5.1704 | -5.6262 | 1.84e-06 | 5.314635e-04 | 4.9925 | ABI3BP |
| 223720\_at | -2.622 | 6.8767 | -3.3525 | 0.0018 | 4.115658e-02 | -1.4195 | SPINK7 |
| 205388\_at | -2.6112 | 4.9615 | -3.0885 | 0.0037 | 6.338805e-02 | -2.075 | TNNC2 |
| 228568\_at | -2.6041 | 5.9787 | -7.473 | 5.58e-09 | 1.453511e-05 | 10.398 | MYZAP |
| 205382\_s\_at | -2.591 | 7.7209 | -6.2576 | 2.49e-07 | 1.585590e-04 | 6.8603 | CFD |

下载-差异分析.xlsx

样本信息

差异分析参考组: ref

| 组别 | 数量 |
| --- | --- |
| ref | 12 |
| test | 26 |

箱式图

**箱式图**: 用箱子绘制每个样本对应的数据情况，可用于查看样本校正情况

· 箱子中间的横线代表中位数，箱子的上边代表上四分位，箱子的下边代表下四分位

· 如果箱子上下存在有黑点，代表此样本存在有离群值

· 一般只要关注各个样本中位数的线 是否在同一个水平线上即可(如果是，则代表样本已经校正好)

PCA图

**PCA图**: 对高纬度数据进行降维后查看样本间差异情况

横坐标代表PCA降维后第1个主成分，纵坐标代表PCA降维后的第2个主成分，括号内代表主成分解释的比例

差异统计

差异分析后一些常见阈值(|logFC|大于2或者1或者是0.58(0.58换算过来就是1.5倍))下的差异分子数量, 也可以根据需要下载差异分析结果用excel表进行过滤

| 筛选条件 | 筛选后的数量 |
| --- | --- |
| |LogFC|>2 & p.adj<0.05 | 120 |
| |LogFC|>1 & p.adj<0.05 | 572 |
| |LogFC|>0.58 & p.adj<0.05 | 1174 |

火山图

**火山图**: 可视化差异分析的结果

**阈值**: logFC(1) | p.adj(0.05)

图中横坐标代表logFC，纵坐标代表p值或者校正后p值

热图

**热图**: 热图主要由一个个不同颜色(深度)的方块组成，每个方块表示行列所对应的数值

**作用**: 主要用于可视化差异表达矩阵情况, 可以从差异分析中挑选差异表达的分子或者ID输入到第一个数据参数框中对数据进行可视化

**补充说明**

· 如果想要调整(列)样本的顺序，可以选择不同的聚类方法或者不对列进行聚类

方法学

**软件**: R (4.2.1)版本

**R包**: GEOquery[2.64.2], limma[3.52.2], ggplot2[3.4.4], ComplexHeatmap[2.13.1]

**补充说明:**

· 数据获取: 通过GEOquery包从GEO数据库中下载GSE9844

· 校正处理: 通过limma包的normalizeBetweenArrays函数再次标准化数据

· 注释处理: 去除掉一个探针对应多个分子的探针；当遇到对应同一个分子的探针时，仅保留信号值最大的探针

· 可视化: 通过箱式图查看查看样本情况, 通过PCA图查看样本分组间聚类情况, 利用limma包进行两组的差异分析, 差异分析结果用火山图进行可视化，同时对显著表达的分子用热图形式进行可视化

**参考文献:**

Davis, Sean, and Paul S. Meltzer. GEOquery: a bridge between the Gene Expression Omnibus (GEO) and BioConductor. Bioinformatics 23.14 (2007): 1846-1847.文献链接

Smyth, Gordon K. Limma: linear models for microarray data. Bioinformatics and computational biology solutions using R and Bioconductor. Springer, New York, NY, 2005. 397-420.文献链接

Gu, Zuguang, Roland Eils, and Matthias Schlesner. Complex heatmaps reveal patterns and correlations in multidimensional genomic data. Bioinformatics 32.18 (2016): 2847-2849.文献链接
