# Supplementary material for: Coagulation factor II receptor-like 1 as a prognostic and immuno-modulatory factor in head and neck squamous cell carcinoma
Source: PeerJ. 2026 Mar 18;14:e20970. doi: 10.7717/peerj.20970 (PMC13005615; doi:10.7717/peerj.20970)
Supplement: Supplemental Information 5 [file peerj-14-20970-s005.zip › Table1.docx]

**Table1** Univariate and multivariate COX regression analysis of F2RL1 in HNSCC

| **Characteristics** | **Total(N)** | **Univariate analysis** | |  | **Multivariate analysis** | |
| --- | --- | --- | --- | --- | --- | --- |
|  |  | **Hazard ratio (95% CI)** | ***P* value** |  | **Hazard ratio (95% CI)** | ***P* value** |
| Pathologic T stage | 445 |  |  |  |  |  |
| T1&T2 | 178 | Reference |  |  | Reference |  |
| T3&T4 | 267 | 1.913 (1.397 - 2.621) | **< 0.001** |  | 1.318 (0.662 - 2.625) | 0.432 |
| Pathologic N stage | 408 |  |  |  |  |  |
| N0&N1 | 236 | Reference |  |  | Reference |  |
| N2&N3 | 172 | 2.288 (1.679 - 3.118) | **< 0.001** |  | 1.668 (1.000 - 2.780) | 0.050 |
| Pathologic stage | 433 |  |  |  |  |  |
| Stage I&Stage II | 94 | Reference |  |  | Reference |  |
| Stage III&Stage IV | 339 | 1.834 (1.232 - 2.729) | **0.003** |  | 2.809 (0.955 - 8.261) | 0.061 |
| Gender | 501 |  |  |  |  |  |
| Female | 133 | Reference |  |  | Reference |  |
| Male | 368 | 0.750 (0.563 - 0.999) | **0.049** |  | 0.864 (0.538 - 1.386) | 0.544 |
| Histologic grade | 479 |  |  |  |  |  |
| G1 | 61 | Reference |  |  | Reference |  |
| G2 | 299 | 1.752 (1.104 - 2.779) | **0.017** |  | 1.333 (0.520 - 3.420) | 0.549 |
| G3 | 119 | 1.509 (0.915 - 2.488) | 0.107 |  | 1.329 (0.467 - 3.784) | 0.594 |
| Lymphovascular invasion | 339 |  |  |  |  |  |
| No | 219 | Reference |  |  | Reference |  |
| Yes | 120 | 1.697 (1.207 - 2.384) | **0.002** |  | 1.274 (0.771 - 2.104) | 0.344 |
| Primary therapy outcome | 416 |  |  |  |  |  |
| PD | 41 | Reference |  |  | Reference |  |
| CR&PR&SD | 375 | 0.155 (0.103 - 0.232) | **< 0.001** |  | 0.254 (0.136 - 0.472) | **< 0.001** |
| Radiation therapy | 439 |  |  |  |  |  |
| No | 153 | Reference |  |  | Reference |  |
| Yes | 286 | 0.627 (0.462 - 0.851) | **0.003** |  | 0.448 (0.264 - 0.761) | **0.003** |
| F2RL1 | 501 | 1.245 (1.105 - 1.403) | **< 0.001** |  | 1.244 (0.978 - 1.583) | 0.075 |
