# Supplementary material for: Coagulation factor II receptor-like 1 as a prognostic and immuno-modulatory factor in head and neck squamous cell carcinoma
Source: PeerJ. 2026 Mar 18;14:e20970. doi: 10.7717/peerj.20970 (PMC13005615; doi:10.7717/peerj.20970)
Supplement: Supplemental Information 5 [file peerj-14-20970-s005.zip › Table2.docx]

**Table 2** Effect of high F2RL1 methylation levels at different CpG sites on the prognostic value of HNSCC

| **Name** | **CpG** | **HR** | **Wald *P* value** |
| --- | --- | --- | --- |
| cg02688752 | Body | 0.668 | 0.0065 |
| cg24573501 | TSS1500 | 0.72 | 0.036 |
| cg27658017 | TSS1500 | 0.694 | 0.024 |
| cg01183017 | TSS200 | 0.55 | 0.00072 |
| cg08793689 | TSS200 | 0.671 | 0.017 |
| cg13900348 | TSS200 | 0.634 | 0.0073 |
| cg19108289 | TSS200 | 0.624 | 0.0037 |
| cg05553591 | 1stExon:5‘UTR | 0.697 | 0.0095 |
| cg23141632 | TSS1500 | 0.69 | 0.0085 |
