# Supplementary material for: Coagulation factor II receptor-like 1 as a prognostic and immuno-modulatory factor in head and neck squamous cell carcinoma
Source: PeerJ. 2026 Mar 18;14:e20970. doi: 10.7717/peerj.20970 (PMC13005615; doi:10.7717/peerj.20970)
Supplement: Supplemental Information 5 [file peerj-14-20970-s005.zip › Figure 1/A/F2RL1-Comparison of subgroups (pan-cancer)/reports.html]

仙桃-分组比较(泛)-在线分析报告


分组比较(泛)-在线分析报告

导出时间: 2024-05-09 11:14:46

目录

- 分组比较(泛)

- 统计描述

- 异常值分析

- 正态性检验

- 方差齐性检验

- Mann-Whitney U检验(Wilcoxon rank sum test)

- 方法学

分组比较(泛)

分组比较(泛)

**分组比较(泛)**: 基于公共数据直接分析分子在多组之间的差别

当前所选的统计方法: **Mann-Whitney U检验(Wilcoxon rank sum test)**

当前数据分组内存在有样本量少于3个或者是组内标准差(SD)为0的情况(SARC, SKCM, THYM, ACC, DLBC, LAML, LGG, MESO, O...)，这些组将不会纳入进行统计分析(仍会进行可视化)

**注意**: 统计要求每组样本都要满足3个样本以上，并且每组样本的方差不能为0，如果不满足条件，就不会进行统计分析

下载-分组比较.pdf

**补充说明**: 该模块会根据数据情况，自动选择合适的统计方法进行统计分析，其中统计方法涵盖:

- 两组: T test(满足正态+方差齐) | Welch t' test(满足正态+不满足方差齐性) | Wilcoxon rank sum test(不满足正态, 非参数检验)

统计描述

各个组常见「统计描述指标」

| 组别1 | 组别2 | 数目 | 最小值 | 最大值 | 中位数(Median) | 四分位距(IQR) | 下四分位 | 上四分位 | 均值(Mean) | 标准差(SD) | 标准误(SE) |
| --- | --- | --- | --- | --- | --- | --- | --- | --- | --- | --- | --- |
| ACC | Tumor | 79 | 0.049073 | 5.9568 | 1.1504 | 1.9398 | 0.56899 | 2.5088 | 1.5499 | 1.2936 | 0.14554 |
| BLCA | Normal | 19 | 0.19535 | 8.4157 | 4.617 | 4.1519 | 1.7056 | 5.8574 | 4.1045 | 2.5377 | 0.5822 |
| BLCA | Tumor | 412 | 0.17198 | 8.342 | 4.5817 | 1.9442 | 3.544 | 5.4882 | 4.456 | 1.4703 | 0.072437 |
| BRCA | Normal | 113 | 0.14639 | 5.1302 | 3.9768 | 1.2669 | 3.1862 | 4.4531 | 3.5718 | 1.3033 | 0.1226 |
| BRCA | Tumor | 1113 | 0 | 6.8271 | 3.6189 | 1.3305 | 2.9062 | 4.2367 | 3.5368 | 1.0589 | 0.031741 |
| CESC | Normal | 3 | 0.38073 | 3.3064 | 0.81065 | 1.4628 | 0.59569 | 2.0585 | 1.4993 | 1.5797 | 0.91205 |
| CESC | Tumor | 306 | 0.83285 | 8.5295 | 5.297 | 1.5564 | 4.4959 | 6.0523 | 5.152 | 1.3582 | 0.077642 |
| CHOL | Normal | 9 | 2.2306 | 5.7682 | 3.2424 | 1.0304 | 3.0585 | 4.0889 | 3.5341 | 1.047 | 0.34901 |
| CHOL | Tumor | 35 | 1.8094 | 8.0167 | 5.8081 | 1.775 | 4.8855 | 6.6605 | 5.7361 | 1.288 | 0.21772 |
| COAD | Normal | 41 | 4.8364 | 8.33 | 7.6861 | 0.40361 | 7.4746 | 7.8782 | 7.6069 | 0.57589 | 0.089939 |
| COAD | Tumor | 480 | 3.1389 | 8.3386 | 6.6122 | 0.83588 | 6.2069 | 7.0428 | 6.5818 | 0.69722 | 0.031823 |
| DLBC | Tumor | 48 | 0.057693 | 2.22 | 0.45867 | 0.72954 | 0.33041 | 1.0599 | 0.71314 | 0.55927 | 0.080723 |
| ESCA | Normal | 11 | 1.2784 | 6.8212 | 5.3688 | 2.1462 | 4.2771 | 6.4233 | 5.0814 | 1.6378 | 0.49382 |
| ESCA | Tumor | 163 | 2.8867 | 9.2118 | 6.0641 | 1.5186 | 5.2597 | 6.7783 | 5.9882 | 1.1633 | 0.091118 |
| GBM | Normal | 5 | 1.1336 | 1.8762 | 1.2705 | 0.24293 | 1.2606 | 1.5035 | 1.4089 | 0.29337 | 0.1312 |
| GBM | Tumor | 169 | 0.74123 | 6.3025 | 3.0828 | 1.6537 | 2.4249 | 4.0786 | 3.2846 | 1.2503 | 0.096174 |
| HNSC | Normal | 44 | 1.0703 | 6.9 | 4.8315 | 1.0313 | 4.2644 | 5.2957 | 4.6482 | 1.1055 | 0.16666 |
| HNSC | Tumor | 504 | 0.35558 | 8.2007 | 5.7588 | 1.2932 | 5.0475 | 6.3407 | 5.4569 | 1.3601 | 0.060583 |
| KICH | Normal | 25 | 5.3919 | 7.7648 | 6.5676 | 0.75321 | 6.2041 | 6.9573 | 6.6519 | 0.60452 | 0.1209 |
| KICH | Tumor | 65 | 0.28558 | 5.8809 | 3.5323 | 1.4358 | 2.6508 | 4.0866 | 3.3426 | 1.1938 | 0.14807 |
| KIRC | Normal | 72 | 4.5816 | 7.6797 | 6.3246 | 1.0728 | 5.8792 | 6.9519 | 6.3893 | 0.69466 | 0.081866 |
| KIRC | Tumor | 541 | 0.49958 | 8.4745 | 5.7187 | 1.4834 | 4.8588 | 6.3422 | 5.4719 | 1.2915 | 0.055525 |
| KIRP | Normal | 32 | 4.9889 | 8.1413 | 6.0643 | 0.5241 | 5.8267 | 6.3508 | 6.1325 | 0.62322 | 0.11017 |
| KIRP | Tumor | 291 | 2.2504 | 9.3858 | 6.1551 | 1.2852 | 5.4946 | 6.7798 | 6.0904 | 1.0891 | 0.063846 |
| LAML | Tumor | 150 | 0 | 6.9433 | 2.2439 | 2.8837 | 1.0327 | 3.9164 | 2.599 | 1.814 | 0.14811 |
| LGG | Tumor | 532 | 0.11849 | 7.1346 | 2.065 | 2.036 | 1.2544 | 3.2904 | 2.3566 | 1.3197 | 0.057218 |
| LIHC | Normal | 50 | 0.64367 | 5.7076 | 3.0622 | 1.6077 | 2.2422 | 3.8499 | 3.0572 | 1.1698 | 0.16544 |
| LIHC | Tumor | 374 | 0 | 7.591 | 3.3408 | 2.78 | 1.657 | 4.437 | 3.1496 | 1.7987 | 0.093009 |
| LUAD | Normal | 59 | 3.023 | 4.9992 | 3.838 | 0.54668 | 3.5977 | 4.1444 | 3.8981 | 0.38895 | 0.050637 |
| LUAD | Tumor | 539 | 0.88315 | 8.8588 | 4.7364 | 1.7029 | 3.9004 | 5.6033 | 4.6608 | 1.3521 | 0.05824 |
| LUSC | Normal | 49 | 2.8584 | 4.9393 | 3.8564 | 0.46929 | 3.5768 | 4.0461 | 3.8252 | 0.38736 | 0.055337 |
| LUSC | Tumor | 502 | 0.99863 | 7.9783 | 5.1194 | 1.8158 | 4.1485 | 5.9643 | 5.0009 | 1.2472 | 0.055667 |
| MESO | Tumor | 87 | 0.11197 | 7.6531 | 3.2655 | 2.0841 | 2.2623 | 4.3464 | 3.4042 | 1.489 | 0.15964 |
| OV | Tumor | 381 | 2.0187 | 7.4238 | 4.3752 | 1.1912 | 3.7901 | 4.9813 | 4.3575 | 0.91106 | 0.046675 |
| PAAD | Normal | 4 | 2.0392 | 5.9224 | 5.7382 | 0.97299 | 4.8124 | 5.7853 | 4.8595 | 1.8822 | 0.94109 |
| PAAD | Tumor | 179 | 1.1777 | 9.2364 | 6.3356 | 1.1211 | 5.6078 | 6.7289 | 6.166 | 1.1308 | 0.08452 |
| PCPG | Normal | 3 | 0.6141 | 2.4326 | 2.0078 | 0.90926 | 1.311 | 2.2202 | 1.6848 | 0.9513 | 0.54924 |
| PCPG | Tumor | 184 | 0.065848 | 4.1428 | 0.91391 | 0.8806 | 0.49157 | 1.3722 | 1.0912 | 0.7966 | 0.058726 |
| PRAD | Normal | 52 | 2.2183 | 6.4571 | 4.8528 | 1.1286 | 4.2804 | 5.409 | 4.762 | 0.96869 | 0.13433 |
| PRAD | Tumor | 501 | 1.5732 | 8.1854 | 4.9206 | 1.8149 | 4.0933 | 5.9082 | 5.0211 | 1.2614 | 0.056353 |
| READ | Normal | 10 | 7.0472 | 8.4608 | 7.7329 | 0.47703 | 7.4252 | 7.9022 | 7.7468 | 0.45479 | 0.14382 |
| READ | Tumor | 167 | 3.7702 | 7.9961 | 6.5099 | 0.84352 | 6.0605 | 6.904 | 6.4137 | 0.70929 | 0.054887 |
| SARC | Normal | 2 | 1.5593 | 5.8577 | 3.7085 | 2.1492 | 2.6339 | 4.7831 | 3.7085 | 3.0394 | 2.1492 |
| SARC | Tumor | 263 | 0.047957 | 7.7784 | 1.5966 | 2.0818 | 0.83806 | 2.9198 | 2.0036 | 1.4954 | 0.09221 |
| SKCM | Normal | 1 | 1.453 | 1.453 | 1.453 | 0 | 1.453 | 1.453 | 1.453 |  |  |
| SKCM | Tumor | 472 | 0 | 9.1769 | 1.246 | 1.3983 | 0.75146 | 2.1498 | 1.6049 | 1.2177 | 0.056051 |
| STAD | Normal | 32 | 0.24025 | 7.388 | 4.7674 | 2.6104 | 3.9316 | 6.5419 | 4.5239 | 2.263 | 0.40004 |
| STAD | Tumor | 375 | 0.86164 | 8.626 | 6.0305 | 1.4863 | 5.2393 | 6.7256 | 5.8457 | 1.2669 | 0.065422 |
| TGCT | Tumor | 156 | 1.7733 | 8.2248 | 5.8244 | 1.6054 | 4.9627 | 6.5681 | 5.684 | 1.1804 | 0.094504 |
| THCA | Normal | 59 | 2.1459 | 5.4813 | 4.5927 | 0.71239 | 4.2834 | 4.9958 | 4.555 | 0.6589 | 0.085781 |
| THCA | Tumor | 512 | 1.1798 | 8.4781 | 4.6537 | 0.97905 | 4.1421 | 5.1211 | 4.6002 | 0.9184 | 0.040588 |
| THYM | Normal | 2 | 1.1423 | 1.9145 | 1.5284 | 0.38607 | 1.3354 | 1.7215 | 1.5284 | 0.54599 | 0.38607 |
| THYM | Tumor | 120 | 0.17313 | 4.9925 | 1.2202 | 1.1849 | 0.79802 | 1.983 | 1.5637 | 1.0704 | 0.097712 |
| UCEC | Normal | 35 | 0.076969 | 6.5752 | 2.9315 | 2.7351 | 1.4995 | 4.2346 | 2.9643 | 1.7777 | 0.30048 |
| UCEC | Tumor | 554 | 0.05589 | 8.6459 | 5.0249 | 1.6104 | 4.109 | 5.7193 | 4.8758 | 1.242 | 0.052767 |
| UCS | Tumor | 57 | 0.26772 | 6.7628 | 4.1208 | 2.771 | 2.4272 | 5.1982 | 3.8042 | 1.6268 | 0.21547 |
| UVM | Tumor | 80 | 0 | 2.174 | 0.16459 | 0.23991 | 0.088752 | 0.32866 | 0.27692 | 0.34832 | 0.038943 |

异常值分析

离群值 = Q1(下四分位) - 1.5\*IQR(四分位间距) 或者 Q3(上四分位) + 1.5\*IQR(四分位间距)

异常值 = Q1(下四分位) - 3.0\*IQR(四分位间距) 或者 Q3(上四分位) + 3.0\*IQR(四分位间距)

| 组别1 | 组别2 | 离群值 | 异常值 |
| --- | --- | --- | --- |
| ACC | Tumor | 5.95678385883887 |  |
| BLCA | Tumor | 0.257493447876156... |  |
| BRCA | Normal | 1.16921951139959,... |  |
| BRCA | Tumor | 6.40404987916231,... |  |
| CESC | Tumor | 2.1516964881709, ... |  |
| CHOL | Normal | 5.76820285468597 |  |
| CHOL | Tumor | 1.80937328259191 |  |
| COAD | Normal | 6.3828055677867, ... | 4.83637863342661 |
| COAD | Tumor | 4.81168166506963,... | 3.13887998490061 |
| DLBC | Tumor | 2.21995839758492 |  |
| ESCA | Tumor | 2.88672571818729,... |  |
| GBM | Normal | 1.87617311443746 |  |
| HNSC | Normal | 2.57736839975281,... | 1.07032062649179 |
| HNSC | Tumor | 2.81485800155505,... | 0.916170875339791... |
| KICH | Tumor | 0.357495703225234... |  |
| KIRC | Tumor | 2.18573970940278,... |  |
| KIRP | Normal | 4.98885734545281,... | 8.14131476190795 |
| KIRP | Tumor | 2.88025451328193,... |  |
| LGG | Tumor | 7.1345710958752, ... |  |
| LUAD | Normal | 4.99923336460397 |  |
| LUAD | Tumor | 8.76657594863468,... |  |
| LUSC | Normal | 2.85837892508207,... |  |
| LUSC | Tumor | 0.998628788282414... |  |
| MESO | Tumor | 7.65309728815637 |  |
| OV | Tumor | 6.82740622270552,... |  |
| PAAD | Normal | 2.03924369627122 |  |
| PAAD | Tumor | 1.17766270753958,... | 1.17766270753958,... |
| PCPG | Tumor | 3.4655957889636, ... | 4.14279734300144 |
| PRAD | Normal | 2.21828520454911 |  |
| READ | Tumor | 4.65423502529769,... |  |
| SARC | Tumor | 6.12530394032789,... |  |
| SKCM | Tumor | 4.37005265571591,... | 9.17691737279612,... |
| STAD | Tumor | 1.89785997361841,... |  |
| TGCT | Tumor | 1.77327901770435,... |  |
| THCA | Normal | 2.14590566009033,... | 2.14590566009033 |
| THCA | Tumor | 2.52044739331893,... | 1.1798294908513, ... |
| THYM | Tumor | 3.77805083872558,... |  |
| UCEC | Tumor | 0.672742134333959... |  |
| UVM | Tumor | 0.892118976957511... | 1.05817793479155,... |

各组离群值和异常值如上所示，如数据确认非人为记录错误，可不进行处理

正态性检验

检验方法: Shapiro-Wilk normality test

| 组别1 | 组别2 | 自由度(df) | 统计量 | p值 |
| --- | --- | --- | --- | --- |
| GBM | Tumor | 168 | 0.97929 | 0.0125 |
| LUAD | Tumor | 538 | 0.99173 | 0.0042 |
| GBM | Normal | 4 | 0.88576 | 0.3363 |
| LUSC | Tumor | 501 | 0.98139 | 4.96e-06 |
| LUSC | Normal | 48 | 0.98084 | 0.6010 |
| PRAD | Tumor | 500 | 0.99485 | 0.0926 |
| UCEC | Tumor | 553 | 0.9702 | 3.52e-09 |
| BLCA | Tumor | 411 | 0.99049 | 0.0092 |
| ESCA | Tumor | 162 | 0.98956 | 0.2725 |
| PAAD | Tumor | 178 | 0.93664 | 4.35e-07 |
| KIRP | Tumor | 290 | 0.98615 | 0.0067 |
| LIHC | Tumor | 373 | 0.97359 | 2.52e-06 |
| CESC | Tumor | 305 | 0.96251 | 4.26e-07 |
| LUAD | Normal | 58 | 0.97538 | 0.2747 |
| BRCA | Tumor | 1112 | 0.99012 | 8.21e-07 |
| COAD | Tumor | 479 | 0.97901 | 2.09e-06 |
| STAD | Tumor | 374 | 0.95987 | 1.33e-08 |
| CHOL | Tumor | 34 | 0.96234 | 0.2685 |
| KIRC | Tumor | 540 | 0.94035 | 6.5e-14 |
| THCA | Tumor | 511 | 0.97036 | 1.16e-08 |
| HNSC | Tumor | 503 | 0.87714 | 1.53e-19 |
| KIRC | Normal | 71 | 0.98602 | 0.6091 |
| KIRP | Normal | 31 | 0.92024 | 0.0211 |
| COAD | Normal | 40 | 0.73142 | 2.6e-07 |
| BRCA | Normal | 112 | 0.84123 | 1.15e-09 |
| READ | Tumor | 166 | 0.97268 | 0.0022 |
| READ | Normal | 9 | 0.94948 | 0.6623 |
| UCEC | Normal | 34 | 0.94841 | 0.1012 |
| LIHC | Normal | 49 | 0.98856 | 0.9068 |
| THCA | Normal | 58 | 0.89241 | 8.02e-05 |
| BLCA | Normal | 18 | 0.91709 | 0.1000 |
| STAD | Normal | 31 | 0.8766 | 0.0017 |
| PRAD | Normal | 51 | 0.97513 | 0.3442 |
| HNSC | Normal | 43 | 0.94497 | 0.0358 |
| CESC | Normal | 2 | 0.85749 | 0.2607 |
| PAAD | Normal | 3 | 0.67039 | 0.0050 |
| ESCA | Normal | 10 | 0.8882 | 0.1320 |
| KICH | Tumor | 64 | 0.96802 | 0.0906 |
| KICH | Normal | 24 | 0.9609 | 0.4328 |
| PCPG | Tumor | 183 | 0.87106 | 1.94e-11 |
| PCPG | Normal | 2 | 0.91356 | 0.4301 |
| CHOL | Normal | 8 | 0.91828 | 0.3782 |

正态性检验结果显示，存在有不满足正态分布的分组(P < 0.05)，建议选择用 非参数检验的方法

方差齐性检验

检验方法: Levene's test

· Base on Mean

| 组别 | 自由度1(df1) | 自由度2(df2) | 统计量 | p值 |
| --- | --- | --- | --- | --- |
| BLCA | 1 | 429 | 19.68 | 1.16e-05 |
| BRCA | 1 | 1224 | 6.581 | 0.0104 |
| CESC | 1 | 307 | 0.11381 | 0.7361 |
| CHOL | 1 | 42 | 0.45921 | 0.5017 |
| COAD | 1 | 519 | 6.243 | 0.0128 |
| ESCA | 1 | 172 | 1.9415 | 0.1653 |
| GBM | 1 | 172 | 6.0108 | 0.0152 |
| HNSC | 1 | 546 | 1.296 | 0.2554 |
| KICH | 1 | 88 | 8.6937 | 0.0041 |
| KIRC | 1 | 611 | 16.102 | 6.75e-05 |
| KIRP | 1 | 321 | 10.329 | 0.0014 |
| LIHC | 1 | 422 | 16.968 | 4.58e-05 |
| LUAD | 1 | 596 | 44.97 | 4.65e-11 |
| LUSC | 1 | 549 | 46.794 | 2.11e-11 |
| PAAD | 1 | 181 | 2.2506 | 0.1353 |
| PCPG | 1 | 185 | 0.15532 | 0.6940 |
| PRAD | 1 | 551 | 6.3262 | 0.0122 |
| READ | 1 | 175 | 1.992 | 0.1599 |
| STAD | 1 | 405 | 25.539 | 6.57e-07 |
| THCA | 1 | 569 | 5.8595 | 0.0158 |
| UCEC | 1 | 587 | 17.063 | 4.14e-05 |

方差齐性检验显示，各组观测变量的方差不相等(P < 0.05)，建议选择用校正方法

Mann-Whitney U检验(Wilcoxon rank sum test)

| 组别 | 组别I | 组别J | 统计量 | 差值(J-I) | 置信区间(95%CI) | p值 |
| --- | --- | --- | --- | --- | --- | --- |
| BLCA | Normal | Tumor | 3868 | 0.049495 | -0.87838 - 1.1573 | 0.9317 |
| BRCA | Normal | Tumor | 7.096e+04 | -0.22952 | -0.42197 - -0.030326 | 0.0243 |
| CESC | Normal | Tumor | 31 | 3.9591 | 1.7649 - 5.4414 | 0.0055 |
| CHOL | Normal | Tumor | 29 | 2.4055 | 1.4565 - 3.1637 | 4.25e-05 |
| COAD | Normal | Tumor | 1.78e+04 | -1.0585 | -1.2243 - -0.88161 | 7.79e-18 |
| ESCA | Normal | Tumor | 603 | 0.76657 | -0.098725 - 1.641 | 0.0700 |
| GBM | Normal | Tumor | 51 | 1.7031 | 0.91659 - 2.7905 | 0.0008 |
| HNSC | Normal | Tumor | 5761 | 0.92758 | 0.62127 - 1.2219 | 1.24e-07 |
| KICH | Normal | Tumor | 1623 | -3.156 | -3.6266 - -2.7533 | 2.95e-13 |
| KIRC | Normal | Tumor | 2.838e+04 | -0.75981 | -0.99975 - -0.52802 | 2.8e-10 |
| KIRP | Normal | Tumor | 4530 | 0.037947 | -0.2757 - 0.33396 | 0.8024 |
| LIHC | Normal | Tumor | 8896 | 0.14723 | -0.37869 - 0.64656 | 0.5774 |
| LUAD | Normal | Tumor | 8548 | 0.83562 | 0.57594 - 1.085 | 5.37e-09 |
| LUSC | Normal | Tumor | 4537 | 1.2954 | 0.99662 - 1.5827 | 2.95e-13 |
| PAAD | Normal | Tumor | 167 | 0.78877 | -0.087288 - 3.2035 | 0.0690 |
| PCPG | Normal | Tumor | 386 | -0.80123 | -1.6835 - 0.43998 | 0.2390 |
| PRAD | Normal | Tumor | 1.17e+04 | 0.21257 | -0.12922 - 0.55107 | 0.2279 |
| READ | Normal | Tumor | 1607 | -1.2674 | -1.6632 - -0.90882 | 9.49e-07 |
| STAD | Normal | Tumor | 4050 | 1.0055 | 0.34749 - 1.6721 | 0.0023 |
| THCA | Normal | Tumor | 1.464e+04 | 0.037421 | -0.14668 - 0.22235 | 0.6975 |
| UCEC | Normal | Tumor | 3680 | 1.8423 | 1.2626 - 2.4792 | 7.28e-10 |

方法学

**软件**: R (4.2.1)版本

**R包**: ggplot2[3.3.6], stats[4.2.1], car[3.1-0]

**处理过程:**

· 根据数据格式特征情况选择合适的统计方法进行统计(stats包以及car包)(如果不满足统计要求将不会进行统计分析)，用ggplot2包对数据进行可视化

**补充说明:**

· 统计方法: Wilcoxon rank sum test

· 所选分子: F2RL1[ENSG00000164251.5]

**数据:**

· 表达数据获取: 从TCGA数据库 ( https://portal.gdc.cancer.gov ) 下载并整理33种肿瘤项目STAR流程的RNAseq数据并提取TPM格式的数据

· 数据过滤策略: 无

· 数据处理方法: log2(value+1)
