# Supplementary material for: Coagulation factor II receptor-like 1 as a prognostic and immuno-modulatory factor in head and neck squamous cell carcinoma
Source: PeerJ. 2026 Mar 18;14:e20970. doi: 10.7717/peerj.20970 (PMC13005615; doi:10.7717/peerj.20970)
Supplement: Supplemental Information 5 [file peerj-14-20970-s005.zip › Figure 1/F/Differences between paired samples/reports.html]

仙桃-配对样本-在线分析报告


配对样本-在线分析报告

导出时间: 2024-05-09 11:08:01

目录

- 配对样本

- 统计描述

- 异常值分析

- 正态性检验

- Wilcoxon signed rank test

- 方法学

配对样本

配对样本

**配对样本**: 基于公共数据直接分析分子在配对样本之间的差别

当前所选的统计方法: **Wilcoxon signed rank test**

**注意**: 统计要求每组样本都要满足3个样本以上，并且每组样本的方差不能为0，如果不满足条件，就不会进行统计分析

下载-配对样本.pdf

统计描述

各个组常见「统计描述指标」

| 组别 | 数目 | 最小值 | 最大值 | 中位数(Median) | 四分位距(IQR) | 下四分位 | 上四分位 | 均值(Mean) | 标准差(SD) | 标准误(SE) |
| --- | --- | --- | --- | --- | --- | --- | --- | --- | --- | --- |
| Normal | 43 | 1.0703 | 6.9 | 4.773 | 1.0697 | 4.2017 | 5.2714 | 4.6329 | 1.1139 | 0.16987 |
| Tumor | 43 | 4.3538 | 7.6058 | 6.026 | 1.0038 | 5.4747 | 6.4785 | 5.9424 | 0.72672 | 0.11082 |

异常值分析

离群值 = Q1(下四分位) - 1.5\*IQR(四分位间距) 或者 Q3(上四分位) + 1.5\*IQR(四分位间距)

异常值 = Q1(下四分位) - 3.0\*IQR(四分位间距) 或者 Q3(上四分位) + 3.0\*IQR(四分位间距)

| 组别 | 离群值 | 异常值 |
| --- | --- | --- |
| Normal | 2.57736839975281,... |  |

各组离群值和异常值如上所示，如数据确认非人为记录错误，可不进行处理

正态性检验

检验方法: Shapiro-Wilk normality test

| 自由度(df) | 统计量 | p值 |
| --- | --- | --- |
| 42 | 0.92349 | 0.0070 |

正态性检验结果显示，存在有不满足正态分布的情况(P < 0.05)，建议选择用 非参数检验的方法

Wilcoxon signed rank test

应用条件: 各组内两两配对样本差值满足不满足正态性时

| 组别I | 组别J | 统计量V | 差值(J-I) | 置信区间(95%CI) | p值 |
| --- | --- | --- | --- | --- | --- |
| Normal | Tumor | 30 | 1.2186 | 0.88012 - 1.5995 | 4.63e-10 |

方法学

**软件**: R (4.2.1)版本

**R包**: ggplot2[3.3.6], stats[4.2.1], car[3.1-0]

**处理过程:**

· 根据数据格式特征情况选择合适的统计方法进行统计(stats包以及car包)(如果不满足统计要求将不会进行统计分析)，用ggplot2包对数据进行可视化

**补充说明:**

· 统计方法: Wilcoxon signed rank test

**数据:**

· 表达数据获取: 从TCGA数据库 ( https://portal.gdc.cancer.gov ) 下载并整理TCGA-HNSC(头颈鳞状细胞癌)项目STAR流程的RNAseq数据并提取TPM格式的数据，提取对应编号配对的癌旁和癌样本

· 数据过滤策略: 去除无临床信息

· 数据处理方法: log2(value+1)
