# Supplementary material for: Coagulation factor II receptor-like 1 as a prognostic and immuno-modulatory factor in head and neck squamous cell carcinoma
Source: PeerJ. 2026 Mar 18;14:e20970. doi: 10.7717/peerj.20970 (PMC13005615; doi:10.7717/peerj.20970)
Supplement: Supplemental Information 5 [file peerj-14-20970-s005.zip › Figure 1/G/reports.html]

仙桃-qPCR实验分析与可视化-在线分析报告


qPCR实验分析与可视化-在线分析报告

导出时间: 2024-05-20 21:25:33

目录

- qPCR实验分析与可视化

- 统计描述

- 异常值分析

- 正态性检验

- 方差齐性检验

- One-way ANOVA

- 多重假设检验(Tukey HSD事后检验)

- 方法学

qPCR实验分析与可视化

qPCR实验分析与可视化

**qPCR实验分析与可视化**: 基于qPCR上机得到的Ct值进行数据整理、统计分析与结果可视化

当前所选的统计方法: **整体检验(One-way ANOVA) + 多重假设检验(Tukey HSD事后检验)**

**注意**: 统计要求每组(第1列分组)都要满足3个样本以上，并且每组样本的方差不能为0，如果不满足条件，就不会进行统计分析

**补充说明**:

· 该模块会根据数据情况，自动选择合适的统计方法进行统计分析，其中统计方法涵盖:

· 两组: T test(满足正态+方差齐) | Welch t' test(满足正态+不满足方差齐性) | Wilcoxon rank sum test(不满足正态, 非参数检验)

· qPRC数据整理和计算过程得到的各个环节的数据可以下载&lt;qPCR整理表&gt;

统计描述

各个组常见「统计描述指标」

| 组别 | 数目 | 最小值 | 最大值 | 中位数(Median) | 四分位距(IQR) | 下四分位 | 上四分位 | 均值(Mean) | 标准差(SD) | 标准误(SE) |
| --- | --- | --- | --- | --- | --- | --- | --- | --- | --- | --- |
| NC | 3 | 0.44237 | 2 | 1.1303 | 0.77881 | 0.78632 | 1.5651 | 1.1909 | 0.78058 | 0.45067 |
| HN-6 | 3 | 1.7804 | 6.6807 | 2.3684 | 2.4501 | 2.0744 | 4.5245 | 3.6098 | 2.6757 | 1.5448 |
| FaDu | 3 | 6.5432 | 11.569 | 10.387 | 2.513 | 8.465 | 10.978 | 9.4997 | 2.6278 | 1.5172 |

异常值分析

离群值 = Q1(下四分位) - 1.5\*IQR(四分位间距) 或者 Q3(上四分位) + 1.5\*IQR(四分位间距)

异常值 = Q1(下四分位) - 3.0\*IQR(四分位间距) 或者 Q3(上四分位) + 3.0\*IQR(四分位间距)

| 组别 | 离群值 | 异常值 |
| --- | --- | --- |

各组不存在 离群值和异常值

正态性检验

检验方法: Shapiro-Wilk normality test

| 组别 | 自由度(df) | 统计量 | p值 |
| --- | --- | --- | --- |
| NC | 2 | 0.99548 | 0.8715 |
| HN-6 | 2 | 0.83854 | 0.2103 |
| FaDu | 2 | 0.91455 | 0.4334 |

正态性检验结果显示，观测变量在各组内接近正态分布(P > 0.05)，建议选择用 参数检验的方法

方差齐性检验

检验方法: Levene's test

· Base on Mean

| 自由度1(df1) | 自由度2(df2) | 统计量 | p值 |
| --- | --- | --- | --- |
| 2 | 6 | 3.0583 | 0.1214 |

方差齐性检验显示，各组观测变量的方差相等(P > 0.05)

One-way ANOVA

| 比较的组 | 分子自由度(DFn) | 分母自由度(DFd) | 统计量 | p值 | η2 |
| --- | --- | --- | --- | --- | --- |
| 组内比较 | 2 | 6 | 11.202 | 0.0094 | 0.78876 |

多重假设检验(Tukey HSD事后检验)

| 分组I | 分组J | 估计值(J-I) | 置信区间(95%CI) | 校正后p值 |
| --- | --- | --- | --- | --- |
| NC | HN-6 | 2.4189 | -3.1217 - 7.9596 | 0.4264 |
| NC | FaDu | 8.3088 | 2.7682 - 13.849 | 0.0088 |
| HN-6 | FaDu | 5.8899 | 0.34928 - 11.431 | 0.0395 |

方法学

**软件**: R (4.2.1)版本

**R包**: ggplot2[3.3.6], stats[4.2.1], car[3.1-0]

**处理过程:**

· 根据公式整理和计算qPCR实验数据，根据数据格式特征情况选择合适的统计方法进行统计(stats包以及car包)(如果不满足统计要求将不会进行统计分析)，用ggplot2包对数据进行可视化

**补充说明:**

· 统计方法: One-way ANOVA
