# Supplementary material for: Coagulation factor II receptor-like 1 as a prognostic and immuno-modulatory factor in head and neck squamous cell carcinoma
Source: PeerJ. 2026 Mar 18;14:e20970. doi: 10.7717/peerj.20970 (PMC13005615; doi:10.7717/peerj.20970)
Supplement: Supplemental Information 5 [file peerj-14-20970-s005.zip › Figure 1/H-L/name.docx]

**CAB012989 CAB012989**
Male, age 51男性，51 岁
Skeletal muscle (T-13000)骨骼肌 (T-13000)
Head-Neck (T-Y0000)头颈 (T-Y0000)
Squamous cell carcinoma, NOS (M-80703)鳞状细胞癌，NOS (M-80703)
Normal tissue, NOS (M-00100)正常组织，NOS (M-00100)
Patient id: 2608患者 ID：2608
**CAB012989** Skeletal muscle Squamous cell carcinoma, NOS (M-80703)

Tumor cells肿瘤细胞

| Staining:染色： | **Low 低的** |
| --- | --- |
|  |  |
| Intensity:强度： | **Weak 虚弱的** |
|  |  |
| Quantity:数量： | **>75% >75%** |
|  |  |
| Location:地点： | **Cytoplasmic/ 细胞质/ membranous 膜质** |

**CAB012989 CAB012989**
Male, age 51男性，51 岁
Skeletal muscle (T-13000)骨骼肌 (T-13000)
Head-Neck (T-Y0000)头颈 (T-Y0000)
Squamous cell carcinoma, NOS (M-80703)鳞状细胞癌，NOS (M-80703)
Normal tissue, NOS (M-00100)正常组织，NOS (M-00100)
Patient id: 2608患者 ID：2608
**CAB012989** Skeletal muscle (T-13000) Squamous cell carcinoma, NOS (M-80703)

Tumor cells肿瘤细胞

| Staining:染色： | **Low 低的** |
| --- | --- |
|  |  |
| Intensity:强度： | **Weak 虚弱的** |
|  |  |
| Quantity:数量： | **>75% >75%** |
|  |  |
| Location:地点： | **Cytoplasmic/ 细胞质/ membranous 膜质** |

**CAB012989 CAB012989**
Female, age 59女性，59 岁
Head-Neck (T-Y0000)头颈 (T-Y0000)
Salivary gland (T-55100)唾液腺 (T-55100)
Adenocarcinoma, NOS (M-81403)腺癌，NOS (M-81403)
Patient id: 773患者 ID：773
**CAB012989** Head-Neck (T-Y0000) Salivary gland (T-55100) Adenocarcinoma, NOS (M-81403)

Tumor cells肿瘤细胞

| Staining:染色： | **Medium 中等的** |
| --- | --- |
|  |  |
| Intensity:强度： | **Moderate 缓和** |
|  |  |
| Quantity:数量： | **>75% >75%** |
|  |  |
| Location:地点： | **Cytoplasmic/ 细胞质/ membranous 膜质** |

**Head and neck cancer头颈癌**

**CAB012989 CAB012989**
Female, age 59女性，59 岁
Head-Neck (T-Y0000)头颈 (T-Y0000)
Salivary gland (T-55100)唾液腺 (T-55100)
Adenocarcinoma, NOS (M-81403)腺癌，NOS (M-81403)
Patient id: 773患者 ID：773
**CAB012989** Head-Neck (T-Y0000)头颈 (T-Y0000)Salivary gland (T-55100)唾液腺 (T-55100)Adenocarcinoma, NOS (M-81403)腺癌

Tumor cells肿瘤细胞

| Staining:染色： | **Medium 中等的** |
| --- | --- |
|  |  |
| Intensity:强度： | **Moderate 缓和** |
|  |  |
| Quantity:数量： | **>75% >75%** |
|  |  |
| Location:地点： | **Cytoplasmic/ 细胞质/ membranous 膜质** |

**CAB012989 CAB012989**
Male, age 62男性，62 岁
Head-Neck (T-Y0000)头颈 (T-Y0000)
Lymph node (T-08000)淋巴结 (T-08000)
Squamous cell carcinoma, metastatic, NOS (M-80706)鳞状细胞癌，转移性，NOS (M-80706)
Squamous cell carcinoma, NOS (M-80703)鳞状细胞癌，NOS (M-80703)
Patient id: 1743患者 ID：1743
**CAB012989** Head-Neck (T-Y0000) Lymph node (T-08000) Squamous cell carcinoma, metastatic, NOS (M-80706)1

Tumor cells肿瘤细胞

| Staining:染色： | **Low 低的** |
| --- | --- |
|  |  |
| Intensity:强度： | **Weak 虚弱的** |
|  |  |
| Quantity:数量： | **>75% >75%** |
|  |  |
| Location:地点： | **Cytoplasmic/ 细胞质/ membranous 膜质** |

**CAB012989 CAB012989**
Female, age 50女性，50 岁
Head-Neck (T-Y0000)头颈 (T-Y0000)
Oral tissue (T-51000)口腔组织 (T-51000)
Squamous cell carcinoma, NOS (M-80703)鳞状细胞癌，NOS (M-80703)
Patient id: 1176患者 ID：1176
**CAB012989** Head-Neck (T-Y0000) Oral tissue (T-51000) Squamous cell carcinoma, NOS (M-80703)

Tumor cells肿瘤细胞

| Staining:染色： | **Medium 中等的** |
| --- | --- |
|  |  |
| Intensity:强度： | **Moderate 缓和** |
|  |  |
| Quantity:数量： | **75%-25% 75%-25%** |
|  |  |
| Location:地点： | **Cytoplasmic/ 细胞质/ membranous 膜质** |
|  |  |

**CAB012989 CAB012989**
Female, age 50女性，50 岁
Head-Neck (T-Y0000)头颈 (T-Y0000)
Oral tissue (T-51000)口腔组织 (T-51000)
Squamous cell carcinoma, NOS (M-80703)鳞状细胞癌，NOS (M-80703)
Patient id: 1176患者 ID：1176
**CAB012989** Head-Neck (T-Y0000) Oral tissue (T-51000) Squamous cell carcinoma, NOS (M-80703)

Tumor cells肿瘤细胞

| Staining:染色： | **Medium 中等的** |
| --- | --- |
|  |  |
| Intensity:强度： | **Moderate 缓和** |
|  |  |
| Quantity:数量： | **75%-25% 75%-25%** |
|  |  |
| Location:地点： | **Cytoplasmic/ 细胞质/ membranous 膜质** |
|  |  |
