# Supplementary material for: Coagulation factor II receptor-like 1 as a prognostic and immuno-modulatory factor in head and neck squamous cell carcinoma
Source: PeerJ. 2026 Mar 18;14:e20970. doi: 10.7717/peerj.20970 (PMC13005615; doi:10.7717/peerj.20970)
Supplement: Supplemental Information 5 [file peerj-14-20970-s005.zip › Figure 2/B/Clinical significance(Race)/reports.html]

仙桃-临床意义(分组)-在线分析报告


临床意义(分组)-在线分析报告

导出时间: 2024-05-10 23:08:18

目录

- 临床意义(分组)

- 统计描述

- 异常值分析

- 正态性检验

- 方差齐性检验

- Kruskal-Wallis Test

- 多重假设检验(Dunn’s test)

- 方法学

临床意义(分组)

临床意义(分组)

**临床意义(分组)**: 基于公共数据直接分析分子在临床变量分组之间的差别

当前所选的统计方法: **整体检验(Kruskal-Wallis Test) + 多重假设检验(Dunn’s test)**

**注意**: 统计要求每组样本都要满足3个样本以上，并且每组样本的方差不能为0，如果不满足条件，就不会进行统计分析

下载-临床意义.pdf

**补充说明**: 该模块会根据数据情况，自动选择合适的统计方法进行统计分析，其中统计方法涵盖:

- 两组: T test(满足正态+方差齐) | Welch t' test(满足正态+不满足方差齐性) | Wilcoxon rank sum test(不满足正态, 非参数检验)

统计描述

各个组常见「统计描述指标」

| 组别 | 数目 | 最小值 | 最大值 | 中位数(Median) | 四分位距(IQR) | 下四分位 | 上四分位 | 均值(Mean) | 标准差(SD) | 标准误(SE) |
| --- | --- | --- | --- | --- | --- | --- | --- | --- | --- | --- |
| Asian | 10 | 5.3048 | 7.0858 | 6.3676 | 0.66715 | 6.0945 | 6.7616 | 6.3597 | 0.55209 | 0.17459 |
| White | 428 | 0.35558 | 8.2007 | 5.7147 | 1.4606 | 4.8688 | 6.3293 | 5.4248 | 1.3806 | 0.066736 |
| Black or African American | 47 | 0.96709 | 7.228 | 5.7529 | 0.82031 | 5.4416 | 6.2619 | 5.6222 | 1.187 | 0.17315 |

异常值分析

离群值 = Q1(下四分位) - 1.5\*IQR(四分位间距) 或者 Q3(上四分位) + 1.5\*IQR(四分位间距)

异常值 = Q1(下四分位) - 3.0\*IQR(四分位间距) 或者 Q3(上四分位) + 3.0\*IQR(四分位间距)

| 组别 | 离群值 | 异常值 |
| --- | --- | --- |
| White | 2.37203271359133,... | 0.355580147377359 |
| Black or African American | 1.57041435461765,... | 1.57041435461765,... |

各组离群值和异常值如上所示，如数据确认非人为记录错误，可不进行处理

正态性检验

检验方法: Shapiro-Wilk normality test

| 组别 | 自由度(df) | 统计量 | p值 |
| --- | --- | --- | --- |
| Asian | 9 | 0.95258 | 0.6990 |
| White | 427 | 0.89171 | 8.05e-17 |
| Black or African American | 46 | 0.75154 | 1.53e-07 |

正态性检验结果显示，存在有不满足正态分布的分组(P < 0.05)，建议选择用 非参数检验的方法

方差齐性检验

检验方法: Levene's test

· Base on Mean

| 自由度1(df1) | 自由度2(df2) | 统计量 | p值 |
| --- | --- | --- | --- |
| 2 | 482 | 4.1506 | 0.0163 |

方差齐性检验显示，各组观测变量的方差不相等(P < 0.05)，建议选择用校正方法

Kruskal-Wallis Test

| 比较的组 | 自由度(df) | 统计量 | p值 |
| --- | --- | --- | --- |
| 组内比较 | 2 | 7.285 | 0.0262 |

多重假设检验(Dunn’s test)

| 组别I | 组别J | 统计量 | 估计值 | 校正后p值 |
| --- | --- | --- | --- | --- |
| Asian | White | -2.6251 | -117.7 | 0.0260 |
| Asian | Black or African American | -2.0798 | -101.51 | 0.1126 |
| White | Black or African American | 0.7516 | 16.187 | 1.0000 |

方法学

**软件**: R (4.2.1)版本

**R包**: ggplot2[3.3.6], stats[4.2.1], car[3.1-0]

**处理过程:**

· 根据数据格式特征情况选择合适的统计方法进行统计(stats包以及car包)(如果不满足统计要求将不会进行统计分析)，用ggplot2包对数据进行可视化

**补充说明:**

· 统计方法: Kruskal-Wallis test

· 所选分子: F2RL1[ENSG00000164251.5]

**数据:**

· 数据获取: 从TCGA数据库 ( https://portal.gdc.cancer.gov ) 下载并整理TCGA-HNSC(头颈鳞状细胞癌)项目STAR流程的RNAseq数据并提取TPM格式的数据 以及 临床数据

· 数据过滤策略: 去除正常+去除无临床信息+去除重复

· 数据处理方法: log2(value+1)
