# Supplementary material for: Coagulation factor II receptor-like 1 as a prognostic and immuno-modulatory factor in head and neck squamous cell carcinoma
Source: PeerJ. 2026 Mar 18;14:e20970. doi: 10.7717/peerj.20970 (PMC13005615; doi:10.7717/peerj.20970)
Supplement: Supplemental Information 5 [file peerj-14-20970-s005.zip › Figure 2/C/Clinical significance(Histologic_grade)/reports.html]

仙桃-临床意义(分组)-在线分析报告


临床意义(分组)-在线分析报告

导出时间: 2024-05-10 23:11:44

目录

- 临床意义(分组)

- 统计描述

- 异常值分析

- 正态性检验

- 方差齐性检验

- Kruskal-Wallis Test

- 多重假设检验(Dunn’s test)

- 方法学

临床意义(分组)

临床意义(分组)

**临床意义(分组)**: 基于公共数据直接分析分子在临床变量分组之间的差别

当前所选的统计方法: **整体检验(Kruskal-Wallis Test) + 多重假设检验(Dunn’s test)**

当前数据分组内存在有样本量少于3个或者是组内标准差(SD)为0的情况(G4)，这些组将不会纳入进行统计分析(仍会进行可视化)

**注意**: 统计要求每组样本都要满足3个样本以上，并且每组样本的方差不能为0，如果不满足条件，就不会进行统计分析

下载-临床意义.pdf

**补充说明**: 该模块会根据数据情况，自动选择合适的统计方法进行统计分析，其中统计方法涵盖:

- 两组: T test(满足正态+方差齐) | Welch t' test(满足正态+不满足方差齐性) | Wilcoxon rank sum test(不满足正态, 非参数检验)

统计描述

各个组常见「统计描述指标」

| 组别 | 数目 | 最小值 | 最大值 | 中位数(Median) | 四分位距(IQR) | 下四分位 | 上四分位 | 均值(Mean) | 标准差(SD) | 标准误(SE) |
| --- | --- | --- | --- | --- | --- | --- | --- | --- | --- | --- |
| G1 | 61 | 3.4035 | 7.228 | 5.9713 | 1.1423 | 5.3673 | 6.5095 | 5.8449 | 0.90932 | 0.11643 |
| G2 | 300 | 0.57376 | 8.2007 | 5.7946 | 1.0519 | 5.3182 | 6.3701 | 5.6422 | 1.1256 | 0.064987 |
| G3 | 119 | 0.35558 | 7.8248 | 5.4742 | 1.9224 | 4.2573 | 6.1797 | 5.0053 | 1.6789 | 0.1539 |
| G4 | 2 | 0.71501 | 5.5586 | 3.1368 | 2.4218 | 1.9259 | 4.3477 | 3.1368 | 3.4249 | 2.4218 |

异常值分析

离群值 = Q1(下四分位) - 1.5\*IQR(四分位间距) 或者 Q3(上四分位) + 1.5\*IQR(四分位间距)

异常值 = Q1(下四分位) - 3.0\*IQR(四分位间距) 或者 Q3(上四分位) + 3.0\*IQR(四分位间距)

| 组别 | 离群值 | 异常值 |
| --- | --- | --- |
| G1 | 3.40345861453549 |  |
| G2 | 2.81485800155505,... | 0.573762295576323... |
| G3 | 1.20751807698255,... |  |

各组离群值和异常值如上所示，如数据确认非人为记录错误，可不进行处理

正态性检验

检验方法: Shapiro-Wilk normality test

| 组别 | 自由度(df) | 统计量 | p值 |
| --- | --- | --- | --- |
| G1 | 60 | 0.95876 | 0.0384 |
| G2 | 299 | 0.88468 | 2.85e-14 |
| G3 | 118 | 0.9009 | 2.4e-07 |

正态性检验结果显示，存在有不满足正态分布的分组(P < 0.05)，建议选择用 非参数检验的方法

方差齐性检验

检验方法: Levene's test

· Base on Mean

| 自由度1(df1) | 自由度2(df2) | 统计量 | p值 |
| --- | --- | --- | --- |
| 2 | 477 | 17.287 | 5.65e-08 |

方差齐性检验显示，各组观测变量的方差不相等(P < 0.05)，建议选择用校正方法

Kruskal-Wallis Test

| 比较的组 | 自由度(df) | 统计量 | p值 |
| --- | --- | --- | --- |
| 组内比较 | 2 | 15.319 | 0.0005 |

多重假设检验(Dunn’s test)

| 组别I | 组别J | 统计量 | 估计值 | 校正后p值 |
| --- | --- | --- | --- | --- |
| G1 | G2 | -1.1318 | -22.049 | 0.7732 |
| G1 | G3 | -3.3539 | -73.258 | 0.0024 |
| G2 | G3 | -3.4077 | -51.208 | 0.0020 |

方法学

**软件**: R (4.2.1)版本

**R包**: ggplot2[3.3.6], stats[4.2.1], car[3.1-0]

**处理过程:**

· 根据数据格式特征情况选择合适的统计方法进行统计(stats包以及car包)(如果不满足统计要求将不会进行统计分析)，用ggplot2包对数据进行可视化

**补充说明:**

· 统计方法: Kruskal-Wallis test

· 所选分子: F2RL1[ENSG00000164251.5]

**数据:**

· 数据获取: 从TCGA数据库 ( https://portal.gdc.cancer.gov ) 下载并整理TCGA-HNSC(头颈鳞状细胞癌)项目STAR流程的RNAseq数据并提取TPM格式的数据 以及 临床数据

· 数据过滤策略: 去除正常+去除无临床信息+去除重复

· 数据处理方法: log2(value+1)
