# Supplementary material for: Coagulation factor II receptor-like 1 as a prognostic and immuno-modulatory factor in head and neck squamous cell carcinoma
Source: PeerJ. 2026 Mar 18;14:e20970. doi: 10.7717/peerj.20970 (PMC13005615; doi:10.7717/peerj.20970)
Supplement: Supplemental Information 5 [file peerj-14-20970-s005.zip › Figure 2/D/Clinical significance(Radiation_therapy)/reports.html]

仙桃-临床意义(分组)-在线分析报告


临床意义(分组)-在线分析报告

导出时间: 2024-05-10 23:28:05

目录

- 临床意义(分组)

- 统计描述

- 异常值分析

- 正态性检验

- 方差齐性检验

- Mann-Whitney U检验(Wilcoxon rank sum test)

- 方法学

临床意义(分组)

临床意义(分组)

**临床意义(分组)**: 基于公共数据直接分析分子在临床变量分组之间的差别

当前所选的统计方法: **Mann-Whitney U检验(Wilcoxon rank sum test)**

**注意**: 统计要求每组样本都要满足3个样本以上，并且每组样本的方差不能为0，如果不满足条件，就不会进行统计分析

**补充说明**: 该模块会根据数据情况，自动选择合适的统计方法进行统计分析，其中统计方法涵盖:

- 两组: T test(满足正态+方差齐) | Welch t' test(满足正态+不满足方差齐性) | Wilcoxon rank sum test(不满足正态, 非参数检验)

统计描述

各个组常见「统计描述指标」

| 组别 | 数目 | 最小值 | 最大值 | 中位数(Median) | 四分位距(IQR) | 下四分位 | 上四分位 | 均值(Mean) | 标准差(SD) | 标准误(SE) |
| --- | --- | --- | --- | --- | --- | --- | --- | --- | --- | --- |
| No | 154 | 0.96709 | 7.6058 | 5.8438 | 1.015 | 5.4041 | 6.4191 | 5.6741 | 1.158 | 0.093317 |
| Yes | 286 | 0.35558 | 7.8422 | 5.6236 | 1.5794 | 4.6867 | 6.2662 | 5.2676 | 1.475 | 0.087217 |

异常值分析

离群值 = Q1(下四分位) - 1.5\*IQR(四分位间距) 或者 Q3(上四分位) + 1.5\*IQR(四分位间距)

异常值 = Q1(下四分位) - 3.0\*IQR(四分位间距) 或者 Q3(上四分位) + 3.0\*IQR(四分位间距)

| 组别 | 离群值 | 异常值 |
| --- | --- | --- |
| No | 2.64339445347989,... | 1.37706820638406,... |
| Yes | 1.20751807698255,... |  |

各组离群值和异常值如上所示，如数据确认非人为记录错误，可不进行处理

正态性检验

检验方法: Shapiro-Wilk normality test

| 组别 | 自由度(df) | 统计量 | p值 |
| --- | --- | --- | --- |
| No | 153 | 0.83302 | 5.75e-12 |
| Yes | 285 | 0.89854 | 6.29e-13 |

正态性检验结果显示，存在有不满足正态分布的分组(P < 0.05)，建议选择用 非参数检验的方法

方差齐性检验

检验方法: Levene's test

· Base on Mean

| 自由度1(df1) | 自由度2(df2) | 统计量 | p值 |
| --- | --- | --- | --- |
| 1 | 438 | 12.358 | 0.0005 |

方差齐性检验显示，各组观测变量的方差不相等(P < 0.05)，建议选择用校正方法

Mann-Whitney U检验(Wilcoxon rank sum test)

| 组别I | 组别J | 统计量 | 差值(J-I) | 置信区间(95%CI) | p值 |
| --- | --- | --- | --- | --- | --- |
| No | Yes | 2.573e+04 | -0.27724 | -0.47778 - -0.08928 | 0.0036 |

方法学

**软件**: R (4.2.1)版本

**R包**: ggplot2[3.3.6], stats[4.2.1], car[3.1-0]

**处理过程:**

· 根据数据格式特征情况选择合适的统计方法进行统计(stats包以及car包)(如果不满足统计要求将不会进行统计分析)，用ggplot2包对数据进行可视化

**补充说明:**

· 统计方法: Wilcoxon rank sum test

· 所选分子: F2RL1[ENSG00000164251.5]

**数据:**

· 数据获取: 从TCGA数据库 ( https://portal.gdc.cancer.gov ) 下载并整理TCGA-HNSC(头颈鳞状细胞癌)项目STAR流程的RNAseq数据并提取TPM格式的数据 以及 临床数据

· 数据过滤策略: 去除正常+去除无临床信息+去除重复

· 数据处理方法: log2(value+1)
