# Supplementary material for: Coagulation factor II receptor-like 1 as a prognostic and immuno-modulatory factor in head and neck squamous cell carcinoma
Source: PeerJ. 2026 Mar 18;14:e20970. doi: 10.7717/peerj.20970 (PMC13005615; doi:10.7717/peerj.20970)
Supplement: Supplemental Information 5 [file peerj-14-20970-s005.zip › Figure 2/G-H/ROC-HNSC-F2RL1-XIANTAO-Histologic-G3+G4vsG1+G2/reports.html]

仙桃-诊断ROC-云-在线分析报告


诊断ROC-云-在线分析报告

导出时间: 2024-05-09 10:44:39

目录

- 诊断ROC-云

- 统计描述

- AUC结果表

- ROC信息表

- 方法学

诊断ROC-云

诊断ROC-云

**诊断ROC-自选变量**: 用于预测所选结局的带分析的变量的准确率情况

· 预测结局(二分类): G3&amp;G4 vs. G1&amp;G2 (其中参考组: G1&amp;G2)[影响图中灵敏度和特异度以及真假阳性和阴性的计算][可以通过特殊参数中的分组顺序]

下载-诊断ROC.pdf

1. ROC曲线图是反映敏感性与特异性之间关系的曲线。横坐标X轴为1–特异性，也称为假阳性率，X轴越接近零准确率越高；纵坐标Y轴称为敏感度，也称为真阳性率(敏感度)，Y轴越大代表准确率越好。

2. ROC曲线下的面积（Area Under Curve，AUC）常用于诊断试验的评估, AUC取值范围一般在0.5和1之间, AUC越接近于1，说明该变量在预测结局上诊断效果越好。

3. ROC曲线的最佳截断值(cut-off)就是假阳性率与假阴性率之和最小时对应的点，常用约登指数来判断最佳截断值，约登指数 = （灵敏度+特异度）-1。

统计描述

各个组常见「统计描述指标」

| 结局 | 变量 | 数目 | 最小值 | 最大值 | 中位数(Median) | 四分位距(IQR) | 下四分位 | 上四分位 | 均值(Mean) | 标准差(SD) | 标准误(SE) |
| --- | --- | --- | --- | --- | --- | --- | --- | --- | --- | --- | --- |
| G1&G2 | F2RL1 | 363 | 0.57376 | 8.2007 | 5.8158 | 1.0666 | 5.3138 | 6.3803 | 5.6666 | 1.1223 | 0.058906 |
| G3&G4 | F2RL1 | 121 | 0.35558 | 7.8248 | 5.4742 | 1.929 | 4.2461 | 6.1751 | 4.9744 | 1.7107 | 0.15552 |

AUC结果表

| 预测变量 | 预测结局 | 曲线下面积(AUC) | 置信区间(CI) |
| --- | --- | --- | --- |
| F2RL1 | 反向 | 0.617 | 0.556 - 0.679 |

预测结局中, 正向或者反向会影响真/假阳性和真/假阴性的区分(如果统计-方向参数选择的是“自动”, 则会对结局的方向会进行调整保证曲线都是往上凸(pROC包提供))(如果选择“正向”或者“反向”，则图形有可能会向下凹)

在AUC＞0.5的情况下，AUC越接近于1，说明该变量在预测结局上诊断效果越好。

AUC在0.5～0.7时有较低准确性, AUC在0.7～0.9时有一定准确性, AUC在0.9以上时有较高准确性。

AUC＝0.5时，说明该变量不起作用，无诊断价值。

ROC信息表

| 预测变量 | cut-off值 | 灵敏度 | 特异度 | 准确率 | 真阳个数 | 真阴个数 | 假阳个数 | 假阴个性 | 阳性预测值 | 阴性预测值 | 约登指数 |
| --- | --- | --- | --- | --- | --- | --- | --- | --- | --- | --- | --- |
| F2RL1 | 5.3789 | 0.4876 | 0.73278 | 0.67149 | 59 | 266 | 97 | 62 | 0.37821 | 0.81098 | 0.22039 |
| F2RL1 | 5.3611 | 0.47934 | 0.74105 | 0.67562 | 58 | 269 | 94 | 63 | 0.38158 | 0.81024 | 0.22039 |
| F2RL1 | 5.291 | 0.46281 | 0.75758 | 0.68388 | 56 | 275 | 88 | 65 | 0.38889 | 0.80882 | 0.22039 |
| F2RL1 | 5.2378 | 0.45455 | 0.76584 | 0.68802 | 55 | 278 | 85 | 66 | 0.39286 | 0.80814 | 0.22039 |

各预测变量在各自最佳cut-off值下部分ROC相关信息和数据。

方法学

**软件**: R (4.2.1)版本

**R包**: pROC[1.18.0], ggplot2[3.3.6]

**处理过程:**

· 使用pROC包进行对数据进行ROC分析，结果用ggplot2进行可视化

· pROC包默认会对数据的结局顺序进行校正(保证结果是往上凸)

**补充说明:**

· 所选分子: F2RL1[ENSG00000164251.5]

· 所选预测结局: (临床)Histologic\_grade

**数据:**

· 数据获取: 从TCGA数据库 ( https://portal.gdc.cancer.gov ) 下载并整理TCGA-HNSC(头颈鳞状细胞癌)项目STAR流程的RNAseq数据并提取TPM格式的数据 以及 临床数据

· 数据过滤策略: 去除正常+去除无临床信息

· 数据处理方法: log2(value+1)
