# Supplementary material for: Coagulation factor II receptor-like 1 as a prognostic and immuno-modulatory factor in head and neck squamous cell carcinoma
Source: PeerJ. 2026 Mar 18;14:e20970. doi: 10.7717/peerj.20970 (PMC13005615; doi:10.7717/peerj.20970)
Supplement: Supplemental Information 5 [file peerj-14-20970-s005.zip › Figure 2/I,F/calibration curve/reports.html]

仙桃-预后Calibration-云-在线分析报告


预后Calibration-云-在线分析报告

导出时间: 2024-05-11 14:04:58

目录

- 预后Calibration-云

- 变量情况

- 中位生存时间

- 单因素Cox

- 多因素Cox

- 比例风险假设(PH)

- 方差膨胀因子(VIF)

- 方法学

预后Calibration-云

预后Calibration-云

**预后Calibration**: 描绘模型在不同时间点对应的预测概率与实际概率之间的差异情况

**预后类型**: OS[Overall Survival]

下载-预后校准曲线.pdf

· 横坐标为模型预测的生存概率；纵坐标为实际观测到的生存概率

· 每条线代表对应各时间点的生存情况与实际情况的对比、以及最理想的线（对角线：灰色）；越贴近对角线说明拟合情况越好

· 每条线的点代表模型预测的生存概率和实际观测到的生存概率情况（类似 nomogram中的最下部分中不同得分对应的概率）

· 每条线的点对应的竖线代表该位置的置信区间

· 每条线上蓝色的叉代表每个点经过分层 Kaplan-Meier 校正后的结果

· 顶部的竖线代表具体样本对应的生存概率（生存率的分布情况），越密集说明越多样本的生存概率在这个概率

变量情况

各个变量识别出来的类型 以及 是否纳入 进行分析

| 变量 | 类型 | 分类数量 | 缺失数量 | 是否纳入分析 | 补充说明 |
| --- | --- | --- | --- | --- | --- |
| event | 数值变量 | - | 0 | 纳入 |  |
| time | 数值变量 | - | 1 | 纳入 |  |
| Pathologic T stage | 分类变量 | 2 | 56 | 纳入 |  |
| Pathologic N stage | 分类变量 | 2 | 93 | 纳入 |  |
| Pathologic stage | 分类变量 | 2 | 68 | 纳入 |  |
| Gender | 分类变量 | 2 | 0 | 纳入 |  |
| Histologic grade | 分类变量 | 3 | 22 | 纳入 |  |
| Lymphovascular invasion | 分类变量 | 2 | 162 | 纳入 |  |
| Primary therapy outcome | 分类变量 | 2 | 85 | 纳入 |  |
| Radiation therapy | 分类变量 | 2 | 62 | 纳入 |  |
| F2RL1 | 数值变量 | - | 0 | 纳入 |  |

总样本数: 502

· 如果某个分类变量的分类&gt;10，将无法识别为分类变量/等级变量

· 如果变量的分组是以 0 1 2此类进行编码，如果分类数量&lt;5, 会被识别为分类变量；如果&gt;5, 会被识别为数值变量

· 如果数据中含有无穷值，无穷值会被当做缺失处理

补充说明: 单因素分析前，会先去掉 结局和时间列 中的缺失的样本(时间或者结局缺失的样本是无法纳入进行分析的)(当前存在有1个样本缺失时间或者结局)

缺失处理策略: 单因素后多因素前处理变量缺失

中位生存时间

中位生存时间只针对分类变量进行，数值变量无法统计中位生存时间

Pathologic T stage

| 分组 | 数目 | 总事件数 | 总删失数 | 总删失比例 | 中位生存时间 | 中位生存时间置信区间 |
| --- | --- | --- | --- | --- | --- | --- |
| T1&T2 | 178 | 58 | 120 | 67.4% | 2570 | 1732-? |
| T3&T4 | 267 | 136 | 131 | 49.1% | 998 | 853-1671 |

Pathologic N stage

| 分组 | 数目 | 总事件数 | 总删失数 | 总删失比例 | 中位生存时间 | 中位生存时间置信区间 |
| --- | --- | --- | --- | --- | --- | --- |
| N0&N1 | 236 | 72 | 164 | 69.5% | 2703 | 1748-? |
| N2&N3 | 172 | 95 | 77 | 44.8% | 789 | 577-1289 |

Pathologic stage

| 分组 | 数目 | 总事件数 | 总删失数 | 总删失比例 | 中位生存时间 | 中位生存时间置信区间 |
| --- | --- | --- | --- | --- | --- | --- |
| Stage I&Stage II | 94 | 29 | 65 | 69.1% | 3059 | 1748-? |
| Stage III&Stage IV | 339 | 157 | 182 | 53.7% | 1394 | 988-1972 |

Gender

| 分组 | 数目 | 总事件数 | 总删失数 | 总删失比例 | 中位生存时间 | 中位生存时间置信区间 |
| --- | --- | --- | --- | --- | --- | --- |
| Female | 133 | 69 | 64 | 48.1% | 1037 | 804-1732 |
| Male | 368 | 148 | 220 | 59.8% | 1762 | 1466-2319 |

Histologic grade

| 分组 | 数目 | 总事件数 | 总删失数 | 总删失比例 | 中位生存时间 | 中位生存时间置信区间 |
| --- | --- | --- | --- | --- | --- | --- |
| G1 | 61 | 22 | 39 | 63.9% | 2717 | 1718-? |
| G2 | 299 | 132 | 167 | 55.9% | 1394 | 985-2002 |
| G3 | 119 | 55 | 64 | 53.8% | 1732 | 993-? |

Lymphovascular invasion

| 分组 | 数目 | 总事件数 | 总删失数 | 总删失比例 | 中位生存时间 | 中位生存时间置信区间 |
| --- | --- | --- | --- | --- | --- | --- |
| No | 219 | 74 | 145 | 66.2% | 2319 | 1641-? |
| Yes | 120 | 62 | 58 | 48.3% | 980 | 680-1748 |

Primary therapy outcome

| 分组 | 数目 | 总事件数 | 总删失数 | 总删失比例 | 中位生存时间 | 中位生存时间置信区间 |
| --- | --- | --- | --- | --- | --- | --- |
| PD | 41 | 33 | 8 | 19.5% | 317 | 276-427 |
| CR&PR&SD | 375 | 121 | 254 | 67.7% | 2570 | 1838-4760 |

Radiation therapy

| 分组 | 数目 | 总事件数 | 总删失数 | 总删失比例 | 中位生存时间 | 中位生存时间置信区间 |
| --- | --- | --- | --- | --- | --- | --- |
| No | 153 | 71 | 82 | 53.6% | 1289 | 882-? |
| Yes | 286 | 99 | 187 | 65.4% | 2319 | 1762-3314 |

备注: 中位生存时间的置信区间如果有?，则代表 分组中样本较少 或者是 随访时间不足 或者是 预后相对较好无法计算出来对应的上限或者下限

单因素Cox

| 变量 | 类型 | 数量 | HR | 置信区间 | p值 |
| --- | --- | --- | --- | --- | --- |
| Pathologic T stage | 等级变量 | 445 |  |  |  |
| T1&T2 |  | 178 | Reference |  |  |
| T3&T4 |  | 267 | 1.913 | 1.397 - 2.621 | 5.32e-05 |
| Pathologic N stage | 等级变量 | 408 |  |  |  |
| N0&N1 |  | 236 | Reference |  |  |
| N2&N3 |  | 172 | 2.288 | 1.679 - 3.118 | 1.6e-07 |
| Pathologic stage | 等级变量 | 433 |  |  |  |
| Stage I&Stage II |  | 94 | Reference |  |  |
| Stage III&Stage IV |  | 339 | 1.834 | 1.232 - 2.729 | 0.0028 |
| Gender | 等级变量 | 501 |  |  |  |
| Female |  | 133 | Reference |  |  |
| Male |  | 368 | 0.750 | 0.563 - 0.999 | 0.0492 |
| Histologic grade | 等级变量 | 479 |  |  |  |
| G1 |  | 61 | Reference |  |  |
| G2 |  | 299 | 1.752 | 1.104 - 2.779 | 0.0172 |
| G3 |  | 119 | 1.509 | 0.915 - 2.488 | 0.1066 |
| Lymphovascular invasion | 等级变量 | 339 |  |  |  |
| No |  | 219 | Reference |  |  |
| Yes |  | 120 | 1.697 | 1.207 - 2.384 | 0.0023 |
| Primary therapy outcome | 等级变量 | 416 |  |  |  |
| PD |  | 41 | Reference |  |  |
| CR&PR&SD |  | 375 | 0.155 | 0.103 - 0.232 | 1.5e-19 |
| Radiation therapy | 等级变量 | 439 |  |  |  |
| No |  | 153 | Reference |  |  |
| Yes |  | 286 | 0.627 | 0.462 - 0.851 | 0.0028 |
| F2RL1 | 数值变量 | 501 | 1.245 | 1.105 - 1.403 | 0.0003 |

表中所有变量都会纳入到多因素中

多因素Cox

| 变量 | 系数β | HR | 置信区间 | p值 |
| --- | --- | --- | --- | --- |
| Pathologic T stage |  |  |  |  |
| T1&T2 |  | Reference |  |  |
| T3&T4 | 0.27621 | 1.318 | 0.662 - 2.625 | 0.4320 |
| Pathologic N stage |  |  |  |  |
| N0&N1 |  | Reference |  |  |
| N2&N3 | 0.5114 | 1.668 | 1.000 - 2.780 | 0.0498 |
| Pathologic stage |  |  |  |  |
| Stage I&Stage II |  | Reference |  |  |
| Stage III&Stage IV | 1.0328 | 2.809 | 0.955 - 8.261 | 0.0606 |
| Gender |  |  |  |  |
| Female |  | Reference |  |  |
| Male | -0.14656 | 0.864 | 0.538 - 1.386 | 0.5439 |
| Histologic grade |  |  |  |  |
| G1 |  | Reference |  |  |
| G2 | 0.28772 | 1.333 | 0.520 - 3.420 | 0.5494 |
| G3 | 0.28432 | 1.329 | 0.467 - 3.784 | 0.5944 |
| Lymphovascular invasion |  |  |  |  |
| No |  | Reference |  |  |
| Yes | 0.24224 | 1.274 | 0.771 - 2.104 | 0.3440 |
| Primary therapy outcome |  |  |  |  |
| PD |  | Reference |  |  |
| CR&PR&SD | -1.3707 | 0.254 | 0.136 - 0.472 | 1.51e-05 |
| Radiation therapy |  |  |  |  |
| No |  | Reference |  |  |
| Yes | -0.80217 | 0.448 | 0.264 - 0.761 | 0.0029 |
| F2RL1 | 0.21854 | 1.244 | 0.978 - 1.583 | 0.0749 |

模型常数/截距(Intercept): -1.2222

原始数据一共有501个, 变量信息缺失的样本有254个, 最终纳入的样本数: 247

备注: 如果出现纳入了多因素但是对应的统计量为空的情况，说明(1)这个变量在去除变量信息缺失后某个分类数目过少(只有1个或者0个)或者是(2)存在严重共线性导致这个变量导致没办法计算。

备注: 当如果多因素中出现HR异常大或者异常小时，说明这个变量的这个分类数量过少或者是存在共线性问题导致

·(分类/等级)变量(非分组)对应的单因素p值为对应变量单因素模型全局性检验的p值，该变量是否纳入取决于此p值

△ 模型全局性统计检验情况：

·· 一致性(Concordance, C-index): 0.773(0.744-0.801)

·· Likelihood ratio test= 74.2 on 10 df, p=6.81e-12

·· Wald test = 88.19 on 10 df, p=1.22e-14

·· Score (logrank) test = 118.04 on 10 df, p=<2e-16

比例风险假设(PH)

Cox回归应用的前提是要求自变量满足等比例风险假设(P > 0.05)，即自变量的风险不会随着时间改变而改变，若不满足，则不适合用Cox回归进行检验。

这里只对多因素模型以及纳入的变量进行ph假设检验

备注: (1)单个变量直接PH假设和在模型里面这个变量的PH假设的结果是不一样的; (2)同一份数据不同Cox模型中同一个变量的PH假设的结果也是不一样的

| 变量 | 统计量(卡方值) | 自由度(df) | p值 |
| --- | --- | --- | --- |
| Pathologic T stage | 4.0561 | 1 | 0.0440 |
| Pathologic N stage | 2.074 | 1 | 0.1498 |
| Pathologic stage | 1.3351 | 1 | 0.2479 |
| Gender | 0.040705 | 1 | 0.8401 |
| Histologic grade | 3.6655 | 2 | 0.1600 |
| Lymphovascular invasion | 0.27029 | 1 | 0.6031 |
| Primary therapy outcome | 1.4657 | 1 | 0.2260 |
| Radiation therapy | 9.1991 | 1 | 0.0024 |
| F2RL1 | 1.2188 | 1 | 0.2696 |
| GLOBAL | 23.224 | 10 | 0.0100 |

如果全局(GLOBAL)满足p > 0.05，可以认为多因素模型满足比例风险假设

方差膨胀因子(VIF)

方差膨胀因子可用于分析模型中的变量是否存在多重共线性问题

| 变量 | 类型 | VIF |
| --- | --- | --- |
| Pathologic T stage | 等级变量 |  |
| T1&T2 |  | Reference |
| T3&T4 |  | 1.6012 |
| Pathologic N stage | 等级变量 |  |
| N0&N1 |  | Reference |
| N2&N3 |  | 1.4126 |
| Pathologic stage | 等级变量 |  |
| Stage I&Stage II |  | Reference |
| Stage III&Stage IV |  | 1.6825 |
| Gender | 等级变量 |  |
| Female |  | Reference |
| Male |  | 1.0772 |
| Histologic grade | 等级变量 |  |
| G1 |  | Reference |
| G2 |  | 4.3514 |
| G3 |  | 4.7875 |
| Lymphovascular invasion | 等级变量 |  |
| No |  | Reference |
| Yes |  | 1.3894 |
| Primary therapy outcome | 等级变量 |  |
| PD |  | Reference |
| CR&PR&SD |  | 1.465 |
| Radiation therapy | 等级变量 |  |
| No |  | Reference |
| Yes |  | 1.4634 |
| F2RL1 | 数值变量 | 1.0933 |

一般认为，当0 < VIF < 10，不存在多重共线性(补充: 也有认为VIF > 4就存在多重共线性); 当10 ≤ VIF < 100，存在较强的多重共线性; 当VIF >= 100或者是出现NaN，多重共线性非常严重

方法学

**软件**: R (4.2.1)版本

**R包**: survival[3.3.1], rms[6.3-0]

**处理过程:**

· 使用survival包进行比例风险假设检验 并 进行Cox回归分析, 使用rms包进行Calibration分析与可视化

**补充说明:**

· 预后类型: OS[Overall Survival]

· 每次重复抽样的样本量: 123

· 抽样次数:800

**数据:**

· 数据获取: 从TCGA数据库 ( https://portal.gdc.cancer.gov ) 下载并整理TCGA-HNSC(头颈鳞状细胞癌)项目STAR流程的RNAseq数据并提取TPM格式的数据 以及 临床数据

· 补充数据: 预后数据来自一篇Cell的文章(LIU, Jianfang, et al., 2018)

· 数据过滤策略: 去除正常+去除无临床信息+去除重复

· 数据处理方法: log2(value+1)

**参考文献:**

LIU, Jianfang, et al. An integrated TCGA pan-cancer clinical data resource to drive high-quality survival outcome analytics. Cell, 2018, 173.2: 400-416. e11.文献链接
