# Supplementary material for: Coagulation factor II receptor-like 1 as a prognostic and immuno-modulatory factor in head and neck squamous cell carcinoma
Source: PeerJ. 2026 Mar 18;14:e20970. doi: 10.7717/peerj.20970 (PMC13005615; doi:10.7717/peerj.20970)
Supplement: Supplemental Information 5 [file peerj-14-20970-s005.zip › Figure 2/J-K/KM-NHSC-DSS-F2RL1-XIANTAO/reports.html]

仙桃-生存曲线-分子-在线分析报告


生存曲线-分子-在线分析报告

导出时间: 2024-05-09 10:31:30

目录

- 生存曲线-分子

- 统计描述

- 比例风险假设(PH)

- 统计分析

- 方法学

生存曲线-分子

生存曲线-分子

**生存曲线图**: 比较F2RL1高低组之间的预后情况

**统计方法**: Cox回归

下载-生存曲线图.pdf

**补充说明**: Cox回归需要满足比例风险假设(PH假设)，默认选择logrank检验

统计描述

| 分组 | 数目 | 总事件数 | 总删失数 | 总删失比例 | 中位生存时间 | 中位生存时间置信区间 |
| --- | --- | --- | --- | --- | --- | --- |
| Low | 239 | 49 | 190 | 79.5% | 无法计算 | ?-? |
| High | 237 | 80 | 157 | 66.2% | 2319 | 1718-? |

备注: 中位生存时间的置信区间如果有?，则代表 分组中样本较少 或者是 随访时间不足 或者是 预后相对较好无法计算出来对应的上限或者下限

· 数据中时间列或者结局列中含有缺失的数量: 26

比例风险假设(PH)

Cox回归应用的前提是要求自变量满足等比例风险假设(P > 0.05)，即自变量的风险不会随着时间改变而改变，若不满足，则不适合用Cox回归进行检验

Logrank检验没有要求满足比例风险假设, 当不满足比例风险假设时可以临时选用Logrank检验, 但是最严谨的是采用RMST(Restricted mean survival time)方法, 当前模块无法兼容采用RMST方法

| 统计量(卡方值) | 自由度 | p值 |
| --- | --- | --- |
| 0.94401 | 1 | 0.3312 |

如果p值小于0.05，则说明变量不满足比例风险假设，此时可以选用Logrank检验

统计分析

同时提供Log-rank和Cox回归的检验结果，Cox回归应用需要满足风险比例假设(PH假设)

| 方法 | 统计量 | HR | 置信区间 | p值 |
| --- | --- | --- | --- | --- |
| Log-rank | 7.7719 | 1.646 | 1.165 - 2.324 | 0.0053 |
| Cox回归 | 7.8312 | 1.652 | 1.156 - 2.360 | 0.0058 |

参考组(Reference): Low

方法学

**软件**: R (4.2.1)版本

**R包**: survival[3.3.1], survminer[0.4.9], ggplot2[3.3.6]

**处理过程:**

· 使用survival包进行比例风险假设检验 并 进行拟合生存回归，结果用survminer包以及ggplot2包进行可视化。

· 如果选用了最佳分组方法(best)，则对应使用survminer包中surv\_cutpoint函数进行最佳分组cut-off筛选。

**补充说明:**

· 统计方法: Cox回归

· 所选分子: F2RL1[ENSG00000164251.5]

· 预后类型: DSS[Disease Specific Survival]

**数据:**

· 数据获取: 从TCGA数据库 ( https://portal.gdc.cancer.gov ) 下载并整理TCGA-HNSC(头颈鳞状细胞癌)项目STAR流程的RNAseq数据并提取TPM格式的数据 以及 临床数据

· 补充数据: 预后数据来自一篇Cell的文章(LIU, Jianfang, et al., 2018)

· 数据过滤策略: 去除正常+去除无临床信息+去除重复

· 数据处理方法: log2(value+1)

**参考文献:**

LIU, Jianfang, et al. An integrated TCGA pan-cancer clinical data resource to drive high-quality survival outcome analytics. Cell, 2018, 173.2: 400-416. e11.文献链接
