# Supplementary material for: Coagulation factor II receptor-like 1 as a prognostic and immuno-modulatory factor in head and neck squamous cell carcinoma
Source: PeerJ. 2026 Mar 18;14:e20970. doi: 10.7717/peerj.20970 (PMC13005615; doi:10.7717/peerj.20970)
Supplement: Supplemental Information 5 [file peerj-14-20970-s005.zip › Figure 3/E/F2RL1-HUB-Chord diagram/reports.html]

仙桃-相关性和弦图-云-在线分析报告


相关性和弦图-云-在线分析报告

导出时间: 2024-05-11 19:54:02

目录

- 相关性和弦图-云

- 相关性分析

- 方法学

相关性和弦图-云

相关性和弦图-云

**相关性和弦图**: 展示数据间的相互关系，用弦的宽度展示相关性强弱

**统计方法**: pearson

下载-相关性和弦图.pdf

和弦图最外层的文字标签显示的是数据中的变量

每个节点代表各个变量区域, 每个变量区域内都展示了该变量与其他变量之间的关系强度，节点上的刻度线显示该变量与其他变量间关系强度的值

连接弦的宽度显示的是两个变量间的相关关系强度大小，每个节点的宽度为连接弦的宽度之和

相关性分析

统计方法: pearson

表1: 相关系数表格

|  | TGFB1 | FGF17 | VEGFC | HBEGF | IL1A | CSF2 | PLAU | TGFA | IL11 | PDGFB |
| --- | --- | --- | --- | --- | --- | --- | --- | --- | --- | --- |
| TGFB1 |  | -0.256 | 0.443 | 0.218 | 0.287 | 0.336 | 0.541 | 0.364 | 0.407 | 0.417 |
| FGF17 | -0.256 |  | -0.367 | -0.227 | -0.305 | -0.174 | -0.283 | -0.297 | -0.0342 | -0.0498 |
| VEGFC | 0.443 | -0.367 |  | 0.177 | 0.414 | 0.524 | 0.59 | 0.347 | 0.502 | 0.311 |
| HBEGF | 0.218 | -0.227 | 0.177 |  | 0.552 | 0.432 | 0.336 | 0.509 | 0.341 | 0.2 |
| IL1A | 0.287 | -0.305 | 0.414 | 0.552 |  | 0.594 | 0.429 | 0.651 | 0.378 | 0.127 |
| CSF2 | 0.336 | -0.174 | 0.524 | 0.432 | 0.594 |  | 0.518 | 0.441 | 0.618 | 0.388 |
| PLAU | 0.541 | -0.283 | 0.59 | 0.336 | 0.429 | 0.518 |  | 0.433 | 0.543 | 0.515 |
| TGFA | 0.364 | -0.297 | 0.347 | 0.509 | 0.651 | 0.441 | 0.433 |  | 0.298 | 0.222 |
| IL11 | 0.407 | -0.0342 | 0.502 | 0.341 | 0.378 | 0.618 | 0.543 | 0.298 |  | 0.517 |
| PDGFB | 0.417 | -0.0498 | 0.311 | 0.2 | 0.127 | 0.388 | 0.515 | 0.222 | 0.517 |  |

表2: 相关性检验表格

|  | TGFB1 | FGF17 | VEGFC | HBEGF | IL1A | CSF2 | PLAU | TGFA | IL11 | PDGFB |
| --- | --- | --- | --- | --- | --- | --- | --- | --- | --- | --- |
| TGFB1 |  | 5.6e-09 | 0 | 8.4e-07 | 5.57e-11 | 1.02e-14 | 0 | 0 | 0 | 0 |
| FGF17 | 5.6e-09 |  | 0 | 2.79e-07 | 3.09e-12 | 9.16e-05 | 1.14e-10 | 1.15e-11 | 0.445 | 0.265 |
| VEGFC | 0 | 0 |  | 6.54e-05 | 0 | 0 | 0 | 1.33e-15 | 0 | 1.07e-12 |
| HBEGF | 8.4e-07 | 2.79e-07 | 6.54e-05 |  | 0 | 0 | 1.13e-14 | 0 | 3.55e-15 | 6.44e-06 |
| IL1A | 5.57e-11 | 3.09e-12 | 0 | 0 |  | 0 | 0 | 0 | 0 | 0.00433 |
| CSF2 | 1.02e-14 | 9.16e-05 | 0 | 0 | 0 |  | 0 | 0 | 0 | 0 |
| PLAU | 0 | 1.14e-10 | 0 | 1.13e-14 | 0 | 0 |  | 0 | 0 | 0 |
| TGFA | 0 | 1.15e-11 | 1.33e-15 | 0 | 0 | 0 | 0 |  | 8.97e-12 | 5.07e-07 |
| IL11 | 0 | 0.445 | 0 | 3.55e-15 | 0 | 0 | 0 | 8.97e-12 |  | 0 |
| PDGFB | 0 | 0.265 | 1.07e-12 | 6.44e-06 | 0.00433 | 0 | 0 | 5.07e-07 | 0 |  |

相关性系数表格：

1. 表中包含了各个变量间的相关系数(r)值，相关系数一般是 -1到1 之间，正负号表示正相关和负相关，系数绝对值大小表示相关性大小

2. 一般关系强度是: |r|>0.95：显著性相关；|r|≥0.8：高度相关；0.5≤|r|<0.8：中度相关；0.3≤|r|<0.5：低度相关；|r|<0.3：弱相关

相关性p值表格：

1. 表中包含了各个变量间的相关性的检验p值

方法学

**软件**: R (4.2.1)版本

**R包**: circlize[v0.4.1]

**处理过程:**

· 分析数据中两两变量之间的相关性，并使用circlize包对相关性结果进行可视化

**补充说明:**

· 统计方法: Pearson

**参考文献:**

GU, Zuguang, et al. circlize implements and enhances circular visualization in R. Bioinformatics, 2014, 30.19: 2811-2812.文献链接

**数据:**

· 数据获取: 从TCGA数据库 ( https://portal.gdc.cancer.gov ) 下载并整理TCGA-HNSC(头颈鳞状细胞癌)项目STAR流程的RNAseq数据并提取TPM格式的数据 以及 临床数据

· 数据过滤策略: 去除正常+去除无临床信息+去除重复

· 数据处理方法: log2(value+1)
