# Supplementary material for: Coagulation factor II receptor-like 1 as a prognostic and immuno-modulatory factor in head and neck squamous cell carcinoma
Source: PeerJ. 2026 Mar 18;14:e20970. doi: 10.7717/peerj.20970 (PMC13005615; doi:10.7717/peerj.20970)
Supplement: Supplemental Information 5 [file peerj-14-20970-s005.zip › Figure 5/A/Immune Infiltration - Subgroup Comparison/reports.html]

仙桃-免疫浸润-分组比较-在线分析报告


免疫浸润-分组比较-在线分析报告

导出时间: 2024-05-10 08:14:39

目录

- 免疫浸润-分组比较

- 统计描述

- 异常值分析

- 正态性检验

- 方差齐性检验

- Mann-Whitney U检验(Wilcoxon rank sum test)

- 方法学

免疫浸润-分组比较

免疫浸润-分组比较

**分组比较**: 基于公共数据分析分子(中位数)高低表达组之间免疫浸润结果的差别

当前所选的统计方法: **Mann-Whitney U检验(Wilcoxon rank sum test)**

**注意**: 统计要求每组样本都要满足3个样本以上，并且每组样本的方差不能为0，如果不满足条件，就不会进行统计分析

**补充说明**: 该模块会根据数据情况，自动选择合适的统计方法进行统计分析，其中统计方法涵盖:

- 两组: T test(满足正态+方差齐) | Welch t' test(满足正态+不满足方差齐性) | Wilcoxon rank sum test(不满足正态, 非参数检验)

统计描述

各个组常见「统计描述指标」

| 组别1 | 组别2 | 数目 | 最小值 | 最大值 | 中位数(Median) | 四分位距(IQR) | 下四分位 | 上四分位 | 均值(Mean) | 标准差(SD) | 标准误(SE) |
| --- | --- | --- | --- | --- | --- | --- | --- | --- | --- | --- | --- |
| aDC | Low | 251 | 0.26812 | 0.8785 | 0.63225 | 0.1719 | 0.54265 | 0.71455 | 0.62335 | 0.11299 | 0.0071319 |
| aDC | High | 251 | 0.28836 | 0.87614 | 0.63429 | 0.18286 | 0.53941 | 0.72227 | 0.62355 | 0.11959 | 0.0075487 |
| B cells | Low | 251 | 0.085785 | 0.70678 | 0.31838 | 0.13477 | 0.25791 | 0.39268 | 0.32627 | 0.10153 | 0.0064084 |
| B cells | High | 251 | 0.10304 | 0.63862 | 0.28481 | 0.1035 | 0.22866 | 0.33216 | 0.28486 | 0.077542 | 0.0048944 |
| CD8 T cells | Low | 251 | 0.63453 | 0.75875 | 0.70588 | 0.033577 | 0.69208 | 0.72565 | 0.70742 | 0.024819 | 0.0015666 |
| CD8 T cells | High | 251 | 0.64687 | 0.75972 | 0.70065 | 0.02832 | 0.68685 | 0.71517 | 0.7002 | 0.020936 | 0.0013215 |
| Cytotoxic cells | Low | 251 | 0.29761 | 0.79835 | 0.56793 | 0.17062 | 0.48203 | 0.65265 | 0.56336 | 0.11081 | 0.0069944 |
| Cytotoxic cells | High | 251 | 0.26275 | 0.76666 | 0.52366 | 0.15866 | 0.44417 | 0.60283 | 0.52291 | 0.10209 | 0.0064442 |
| DC | Low | 251 | 0.11821 | 0.76339 | 0.50132 | 0.12266 | 0.44808 | 0.57073 | 0.51263 | 0.096251 | 0.0060753 |
| DC | High | 251 | 0.24252 | 0.77543 | 0.51154 | 0.12834 | 0.44101 | 0.56936 | 0.50707 | 0.096419 | 0.0060859 |
| Eosinophils | Low | 251 | 0.36716 | 0.55494 | 0.46161 | 0.040715 | 0.44054 | 0.48126 | 0.46146 | 0.032354 | 0.0020422 |
| Eosinophils | High | 251 | 0.38701 | 0.59099 | 0.47667 | 0.038875 | 0.45751 | 0.49638 | 0.47763 | 0.031757 | 0.0020045 |
| iDC | Low | 251 | 0.41775 | 0.73604 | 0.57922 | 0.076201 | 0.54207 | 0.61827 | 0.57983 | 0.060741 | 0.0038339 |
| iDC | High | 251 | 0.43989 | 0.70388 | 0.58612 | 0.077999 | 0.5504 | 0.6284 | 0.58647 | 0.053641 | 0.0033858 |
| Macrophages | Low | 251 | 0.38876 | 0.82455 | 0.58863 | 0.10566 | 0.53576 | 0.64142 | 0.59188 | 0.076573 | 0.0048332 |
| Macrophages | High | 251 | 0.41896 | 0.85889 | 0.60687 | 0.089055 | 0.55755 | 0.6466 | 0.60795 | 0.070376 | 0.0044421 |
| Mast cells | Low | 251 | 0.11087 | 0.54529 | 0.32402 | 0.10336 | 0.26719 | 0.37054 | 0.32122 | 0.078445 | 0.0049514 |
| Mast cells | High | 251 | 0.13274 | 0.54994 | 0.32668 | 0.11018 | 0.27837 | 0.38855 | 0.3318 | 0.079721 | 0.0050319 |
| Neutrophils | Low | 251 | 0.28043 | 0.7206 | 0.44736 | 0.1004 | 0.40056 | 0.50096 | 0.44966 | 0.070184 | 0.00443 |
| Neutrophils | High | 251 | 0.28927 | 0.78324 | 0.48616 | 0.095933 | 0.43526 | 0.53119 | 0.48869 | 0.078456 | 0.0049521 |
| NK CD56bright cells | Low | 251 | 0.39319 | 0.69762 | 0.54232 | 0.081313 | 0.50639 | 0.58771 | 0.54967 | 0.056372 | 0.0035582 |
| NK CD56bright cells | High | 251 | 0.40541 | 0.67966 | 0.53085 | 0.057698 | 0.49817 | 0.55587 | 0.53013 | 0.04973 | 0.003139 |
| NK CD56dim cells | Low | 251 | 0.17314 | 0.72793 | 0.41095 | 0.1169 | 0.35543 | 0.47233 | 0.41585 | 0.08993 | 0.0056763 |
| NK CD56dim cells | High | 251 | 0.19637 | 0.61925 | 0.40824 | 0.11968 | 0.34325 | 0.46293 | 0.40433 | 0.079847 | 0.0050399 |
| NK cells | Low | 251 | 0.49965 | 0.70871 | 0.60134 | 0.049696 | 0.57806 | 0.62776 | 0.60384 | 0.036454 | 0.002301 |
| NK cells | High | 251 | 0.50213 | 0.67625 | 0.60113 | 0.044352 | 0.58087 | 0.62523 | 0.60235 | 0.03131 | 0.0019763 |
| pDC | Low | 251 | 0.33313 | 0.99018 | 0.68662 | 0.17469 | 0.6088 | 0.78349 | 0.69685 | 0.12007 | 0.007579 |
| pDC | High | 251 | 0.37043 | 0.91811 | 0.62793 | 0.14715 | 0.55677 | 0.70392 | 0.63896 | 0.10505 | 0.0066305 |
| T cells | Low | 251 | 0.23925 | 0.8227 | 0.55599 | 0.18434 | 0.4715 | 0.65584 | 0.56078 | 0.1277 | 0.0080603 |
| T cells | High | 251 | 0.23287 | 0.81916 | 0.52732 | 0.16443 | 0.42963 | 0.59406 | 0.5208 | 0.11414 | 0.0072042 |
| T helper cells | Low | 251 | 0.64879 | 0.84284 | 0.75631 | 0.038666 | 0.73714 | 0.77581 | 0.75471 | 0.033204 | 0.0020958 |
| T helper cells | High | 251 | 0.70566 | 0.828 | 0.75799 | 0.02776 | 0.74485 | 0.77261 | 0.75875 | 0.022101 | 0.001395 |
| Tcm | Low | 251 | 0.43953 | 0.64672 | 0.56411 | 0.045263 | 0.54116 | 0.58642 | 0.56371 | 0.035684 | 0.0022523 |
| Tcm | High | 251 | 0.50474 | 0.65781 | 0.57642 | 0.03596 | 0.55845 | 0.59441 | 0.57615 | 0.026468 | 0.0016706 |
| Tem | Low | 251 | 0.4082 | 0.63948 | 0.52988 | 0.065776 | 0.49916 | 0.56494 | 0.5286 | 0.04587 | 0.0028953 |
| Tem | High | 251 | 0.37346 | 0.65831 | 0.52371 | 0.060823 | 0.49396 | 0.55478 | 0.52582 | 0.044337 | 0.0027985 |
| TFH | Low | 251 | 0.35975 | 0.58927 | 0.47834 | 0.055834 | 0.4519 | 0.50774 | 0.47945 | 0.042745 | 0.002698 |
| TFH | High | 251 | 0.3769 | 0.55619 | 0.47753 | 0.043221 | 0.45643 | 0.49965 | 0.47694 | 0.032778 | 0.0020689 |
| Tgd | Low | 251 | 0.19295 | 0.58766 | 0.39908 | 0.079254 | 0.35992 | 0.43917 | 0.39923 | 0.056683 | 0.0035778 |
| Tgd | High | 251 | 0.27554 | 0.62903 | 0.43787 | 0.072525 | 0.39754 | 0.47006 | 0.43609 | 0.057876 | 0.0036531 |
| Th1 cells | Low | 251 | 0.3796 | 0.65542 | 0.52571 | 0.068511 | 0.49392 | 0.56243 | 0.52585 | 0.051084 | 0.0032244 |
| Th1 cells | High | 251 | 0.36622 | 0.64986 | 0.5455 | 0.070128 | 0.50374 | 0.57387 | 0.53955 | 0.049457 | 0.0031217 |
| Th17 cells | Low | 251 | 0.029963 | 0.57514 | 0.35087 | 0.1112 | 0.29318 | 0.40438 | 0.34456 | 0.08681 | 0.0054794 |
| Th17 cells | High | 251 | 0.12259 | 0.59574 | 0.33165 | 0.10948 | 0.27935 | 0.38883 | 0.33095 | 0.084567 | 0.0053378 |
| Th2 cells | Low | 251 | 0.4238 | 0.62444 | 0.54676 | 0.05056 | 0.5197 | 0.57026 | 0.54525 | 0.03435 | 0.0021681 |
| Th2 cells | High | 251 | 0.46012 | 0.64273 | 0.55842 | 0.041763 | 0.53729 | 0.57906 | 0.55829 | 0.031047 | 0.0019597 |
| TReg | Low | 251 | 0.12895 | 0.93927 | 0.56366 | 0.21984 | 0.459 | 0.67884 | 0.56724 | 0.1596 | 0.010074 |
| TReg | High | 251 | 0.13014 | 0.91038 | 0.55073 | 0.18434 | 0.46634 | 0.65068 | 0.5531 | 0.14181 | 0.0089507 |

异常值分析

离群值 = Q1(下四分位) - 1.5\*IQR(四分位间距) 或者 Q3(上四分位) + 1.5\*IQR(四分位间距)

异常值 = Q1(下四分位) - 3.0\*IQR(四分位间距) 或者 Q3(上四分位) + 3.0\*IQR(四分位间距)

| 组别1 | 组别2 | 离群值 | 异常值 |
| --- | --- | --- | --- |
| aDC | Low | 0.26812334163151 |  |
| B cells | Low | 0.608682732824903... |  |
| B cells | High | 0.638619188530835 |  |
| CD8 T cells | Low | 0.634531888923104 |  |
| CD8 T cells | High | 0.759717571120247 |  |
| DC | Low | 0.118208893356081... |  |
| DC | High | 0.77542516122592,... |  |
| Eosinophils | Low | 0.367978670451763... |  |
| Eosinophils | High | 0.38701416508561,... |  |
| iDC | Low | 0.736035000344204... |  |
| Macrophages | Low | 0.82455137468625,... |  |
| Macrophages | High | 0.834598319417593... |  |
| Mast cells | Low | 0.110867902499128... |  |
| Neutrophils | Low | 0.720597703506433 |  |
| Neutrophils | High | 0.685791242222557... |  |
| NK CD56bright cells | High | 0.409552899322142... |  |
| NK CD56dim cells | Low | 0.173138356845271... |  |
| NK cells | Low | 0.499653518249493... |  |
| NK cells | High | 0.502132583903561 |  |
| pDC | Low | 0.333125429062017 |  |
| T helper cells | Low | 0.655188903619651... |  |
| T helper cells | High | 0.824827199669671... |  |
| Tcm | Low | 0.471300840997033... |  |
| Tcm | High | 0.657809017632657... |  |
| Tem | High | 0.373464097557412... |  |
| TFH | Low | 0.359748964147839 |  |
| TFH | High | 0.376902347442787 |  |
| Tgd | Low | 0.561320634310984... |  |
| Tgd | High | 0.595620573992758... |  |
| Th1 cells | Low | 0.379601563675231 |  |
| Th1 cells | High | 0.392789867652092... |  |
| Th17 cells | Low | 0.036845544625172... |  |
| Th17 cells | High | 0.565252422154354... |  |
| Th2 cells | Low | 0.423801325170845 |  |
| Th2 cells | High | 0.642734222172606... |  |
| TReg | Low | 0.128949958564579 |  |
| TReg | High | 0.130135056652043... |  |

各组离群值和异常值如上所示，如数据确认非人为记录错误，可不进行处理

正态性检验

检验方法: Shapiro-Wilk normality test

| 组别1 | 组别2 | 自由度(df) | 统计量 | p值 |
| --- | --- | --- | --- | --- |
| aDC | High | 250 | 0.98256 | 0.0036 |
| aDC | Low | 250 | 0.98306 | 0.0044 |
| B cells | High | 250 | 0.98037 | 0.0015 |
| B cells | Low | 250 | 0.98771 | 0.0305 |
| CD8 T cells | High | 250 | 0.99533 | 0.6482 |
| CD8 T cells | Low | 250 | 0.99052 | 0.1021 |
| Cytotoxic cells | High | 250 | 0.99174 | 0.1714 |
| Cytotoxic cells | Low | 250 | 0.98626 | 0.0165 |
| DC | High | 250 | 0.99737 | 0.9570 |
| DC | Low | 250 | 0.97948 | 0.0011 |
| Eosinophils | High | 250 | 0.99128 | 0.1410 |
| Eosinophils | Low | 250 | 0.99521 | 0.6259 |
| iDC | High | 250 | 0.9922 | 0.2078 |
| iDC | Low | 250 | 0.99379 | 0.3873 |
| Macrophages | High | 250 | 0.99254 | 0.2384 |
| Macrophages | Low | 250 | 0.99085 | 0.1178 |
| Mast cells | High | 250 | 0.99521 | 0.6246 |
| Mast cells | Low | 250 | 0.99651 | 0.8543 |
| Neutrophils | High | 250 | 0.98273 | 0.0039 |
| Neutrophils | Low | 250 | 0.99293 | 0.2790 |
| NK CD56bright cells | High | 250 | 0.9899 | 0.0784 |
| NK CD56bright cells | Low | 250 | 0.9863 | 0.0168 |
| NK CD56dim cells | High | 250 | 0.99379 | 0.3881 |
| NK CD56dim cells | Low | 250 | 0.99649 | 0.8517 |
| NK cells | High | 250 | 0.99266 | 0.2507 |
| NK cells | Low | 250 | 0.99201 | 0.1918 |
| pDC | High | 250 | 0.98983 | 0.0761 |
| pDC | Low | 250 | 0.99192 | 0.1846 |
| T cells | High | 250 | 0.98984 | 0.0762 |
| T cells | Low | 250 | 0.98861 | 0.0450 |
| T helper cells | High | 250 | 0.99468 | 0.5296 |
| T helper cells | Low | 250 | 0.97789 | 0.0006 |
| Tcm | High | 250 | 0.99808 | 0.9928 |
| Tcm | Low | 250 | 0.98586 | 0.0139 |
| Tem | High | 250 | 0.99577 | 0.7280 |
| Tem | Low | 250 | 0.98938 | 0.0625 |
| TFH | High | 250 | 0.9957 | 0.7153 |
| TFH | Low | 250 | 0.99553 | 0.6849 |
| Tgd | High | 250 | 0.99676 | 0.8903 |
| Tgd | Low | 250 | 0.99513 | 0.6099 |
| Th1 cells | High | 250 | 0.98389 | 0.0061 |
| Th1 cells | Low | 250 | 0.99318 | 0.3077 |
| Th17 cells | High | 250 | 0.99318 | 0.3072 |
| Th17 cells | Low | 250 | 0.98447 | 0.0078 |
| Th2 cells | High | 250 | 0.99555 | 0.6874 |
| Th2 cells | Low | 250 | 0.99016 | 0.0875 |
| TReg | High | 250 | 0.99687 | 0.9052 |
| TReg | Low | 250 | 0.99027 | 0.0919 |

正态性检验结果显示，存在有不满足正态分布的分组(P < 0.05)，建议选择用 非参数检验的方法

方差齐性检验

检验方法: Levene's test

· Base on Mean

| 组别 | 自由度1(df1) | 自由度2(df2) | 统计量 | p值 |
| --- | --- | --- | --- | --- |
| aDC | 1 | 500 | 0.57296 | 0.4494 |
| B cells | 1 | 500 | 11.599 | 0.0007 |
| CD8 T cells | 1 | 500 | 5.538 | 0.0190 |
| Cytotoxic cells | 1 | 500 | 2.3169 | 0.1286 |
| DC | 1 | 500 | 0.34343 | 0.5581 |
| Eosinophils | 1 | 500 | 0.19404 | 0.6598 |
| iDC | 1 | 500 | 2.0477 | 0.1531 |
| Macrophages | 1 | 500 | 1.9624 | 0.1619 |
| Mast cells | 1 | 500 | 0.18571 | 0.6667 |
| Neutrophils | 1 | 500 | 1.6267 | 0.2028 |
| NK CD56bright cells | 1 | 500 | 6.7665 | 0.0096 |
| NK CD56dim cells | 1 | 500 | 2.3706 | 0.1243 |
| NK cells | 1 | 500 | 6.6609 | 0.0101 |
| pDC | 1 | 500 | 6.0743 | 0.0141 |
| T cells | 1 | 500 | 2.9552 | 0.0862 |
| T helper cells | 1 | 500 | 23.575 | 1.61e-06 |
| Tcm | 1 | 500 | 13.75 | 0.0002 |
| Tem | 1 | 500 | 1.3421 | 0.2472 |
| TFH | 1 | 500 | 15.101 | 0.0001 |
| Tgd | 1 | 500 | 0.06008 | 0.8065 |
| Th1 cells | 1 | 500 | 0.017927 | 0.8935 |
| Th17 cells | 1 | 500 | 0.084497 | 0.7714 |
| Th2 cells | 1 | 500 | 3.6669 | 0.0561 |
| TReg | 1 | 500 | 5.3349 | 0.0213 |

方差齐性检验显示，各组观测变量的方差不相等(P < 0.05)，建议选择用校正方法

Mann-Whitney U检验(Wilcoxon rank sum test)

| 组别 | 组别I | 组别J | 统计量 | 差值(J-I) | 置信区间(95%CI) | p值 |
| --- | --- | --- | --- | --- | --- | --- |
| aDC | Low | High | 3.134e+04 | 0.00096445 | -0.019983 - 0.022186 | 0.9225 |
| B cells | Low | High | 3.959e+04 | -0.040066 | -0.056533 - -0.024907 | 6.52e-07 |
| CD8 T cells | Low | High | 3.687e+04 | -0.0071136 | -0.01131 - -0.0028848 | 0.0010 |
| Cytotoxic cells | Low | High | 3.81e+04 | -0.041551 | -0.061754 - -0.021605 | 4.85e-05 |
| DC | Low | High | 3.175e+04 | -0.0014456 | -0.018918 - 0.015981 | 0.8772 |
| Eosinophils | Low | High | 2.24e+04 | 0.015976 | 0.010556 - 0.021411 | 2.13e-08 |
| iDC | Low | High | 2.951e+04 | 0.006642 | -0.0038009 - 0.016964 | 0.2203 |
| Macrophages | Low | High | 2.715e+04 | 0.017233 | 0.0045987 - 0.030223 | 0.0074 |
| Mast cells | Low | High | 2.952e+04 | 0.009242 | -0.0055042 - 0.023345 | 0.2242 |
| Neutrophils | Low | High | 2.271e+04 | 0.036795 | 0.023573 - 0.049882 | 6.31e-08 |
| NK CD56bright cells | Low | High | 3.754e+04 | -0.0183 | -0.028034 - -0.0086302 | 0.0002 |
| NK CD56dim cells | Low | High | 3.352e+04 | -0.009773 | -0.025282 - 0.0055072 | 0.2141 |
| NK cells | Low | High | 3.171e+04 | -0.00037099 | -0.0067059 - 0.0055889 | 0.8962 |
| pDC | Low | High | 4.036e+04 | -0.057741 | -0.078924 - -0.037446 | 5.01e-08 |
| T cells | Low | High | 3.734e+04 | -0.042553 | -0.065421 - -0.019418 | 0.0003 |
| T helper cells | Low | High | 2.996e+04 | 0.0022226 | -0.0023717 - 0.0069865 | 0.3436 |
| Tcm | Low | High | 2.472e+04 | 0.011643 | 0.0062929 - 0.017121 | 2.98e-05 |
| Tem | Low | High | 3.305e+04 | -0.0040511 | -0.012534 - 0.0042827 | 0.3405 |
| TFH | Low | High | 3.258e+04 | -0.0024396 | -0.009311 - 0.0045767 | 0.5059 |
| Tgd | Low | High | 2.04e+04 | 0.036929 | 0.026875 - 0.047028 | 8.35e-12 |
| Th1 cells | Low | High | 2.624e+04 | 0.014635 | 0.0057161 - 0.023563 | 0.0012 |
| Th17 cells | Low | High | 3.479e+04 | -0.016102 | -0.031341 - -0.00056255 | 0.0430 |
| Th2 cells | Low | High | 2.49e+04 | 0.012637 | 0.0066312 - 0.018571 | 4.93e-05 |
| TReg | Low | High | 3.298e+04 | -0.012925 | -0.040019 - 0.01499 | 0.3641 |

方法学

**软件**: R (4.2.1)版本

**R包**: ggplot2[3.3.6], stats[4.2.1], car[3.1-0]

**处理过程:**

· 对主变量进行分组后，根据数据格式特征情况选择合适的统计方法进行统计(stats包以及car包)(如果不满足统计要求将不会进行统计分析)，用ggplot2包对数据进行可视化

**补充说明:**

· 统计方法: Wilcoxon rank sum test

· 主分子: F2RL1[ENSG00000164251.5]

· 免疫浸润算法: 基于R包-GSVA[1.46.0](Hänzelmann et al., 2013)中提供的ssGSEA算法, 利用Immunity文章(Bindea, Gabriela, et al., 2013)提供的24种免疫细胞的markers来计算对应云端数据的免疫浸润情况，具体24种免疫细胞可查看对应参考文献

· 免疫细胞: aDC[activated DC]; B cells; CD8 T cells; Cytotoxic cells; DC; Eosinophils; iDC[immature DC]; Macrophages; Mast cells; Neutrophils; NK CD56bright cells; NK CD56dim cells; NK cells; pDC[Plasmacytoid DC]; T cells; T helper cells; Tcm[T central memory]; Tem[T effector memory]; TFH[T follicular helper]; Tgd[T gamma delta]; Th1 cells; Th17 cells; Th2 cells; TReg

**参考文献:**

Hänzelmann, Sonja, Robert Castelo, and Justin Guinney. GSVA: gene set variation analysis for microarray and RNA-seq data. BMC bioinformatics 14.1 (2013): 1-15.文献链接

Bindea, Gabriela, et al. Spatiotemporal dynamics of intratumoral immune cells reveal the immune landscape in human cancer. Immunity 39.4 (2013): 782-795.文献链接

**数据:**

· 数据获取: 从TCGA数据库 ( https://portal.gdc.cancer.gov ) 下载并整理TCGA-HNSC(头颈鳞状细胞癌)项目STAR流程的RNAseq数据并提取TPM格式的数据 以及 临床数据

· 数据过滤策略: 去除正常+去除无临床信息+去除重复

· 数据处理方法: log2(value+1)
