# Supplementary material for: Coagulation factor II receptor-like 1 as a prognostic and immuno-modulatory factor in head and neck squamous cell carcinoma
Source: PeerJ. 2026 Mar 18;14:e20970. doi: 10.7717/peerj.20970 (PMC13005615; doi:10.7717/peerj.20970)
Supplement: Supplemental Information 5 [file peerj-14-20970-s005.zip › Figure 5/C/F2RL1-HNSE-Immune Infiltration - Lollipop Chart/reports.html]

仙桃-免疫浸润-棒棒糖图-在线分析报告


免疫浸润-棒棒糖图-在线分析报告

导出时间: 2024-05-09 13:49:03

目录

- 免疫浸润-棒棒糖图

- 相关性分析

- 方法学

免疫浸润-棒棒糖图

免疫浸润-棒棒糖图

**相关性棒棒糖图**: 分析F2RL1和免疫浸润之间的相关性结果并用棒棒糖图形式展示结果

**统计方法**: spearman

下载-相关性棒棒糖图.pdf

具体相关性情况可查看<补充结果>

相关性分析

提供Pearson和Spearman统计方法的结果

| 主变量 | 次变量 | 自由度(df) | 统计量-Pearson | 相关系数-Pearson | p值-Pearson | 统计量-Spearman | 相关系数-Spearman | p值-Spearman |
| --- | --- | --- | --- | --- | --- | --- | --- | --- |
| F2RL1 | aDC | 502 | -2.19169 | -0.0973551 | 0.0289 | 2.195e+07 | -0.0286868 | 0.5204 |
| F2RL1 | B cells | 502 | -7.74972 | -0.326885 | 5.15e-14 | 2.705e+07 | -0.267591 | 1.2e-09 |
| F2RL1 | CD8 T cells | 502 | -5.70802 | -0.246876 | 1.96e-08 | 2.49e+07 | -0.167127 | 0.0002 |
| F2RL1 | Cytotoxic cells | 502 | -7.36027 | -0.312096 | 7.56e-13 | 2.634e+07 | -0.234456 | 1.1e-07 |
| F2RL1 | DC | 502 | -0.88159 | -0.0393169 | 0.3784 | 2.176e+07 | -0.019983 | 0.6544 |
| F2RL1 | Eosinophils | 502 | 5.85964 | 0.253019 | 8.4e-09 | 1.551e+07 | 0.27309 | 5.35e-10 |
| F2RL1 | iDC | 502 | 2.64427 | 0.117206 | 0.0084 | 1.972e+07 | 0.0757646 | 0.0893 |
| F2RL1 | Macrophages | 502 | 3.77704 | 0.166232 | 0.0002 | 1.836e+07 | 0.139697 | 0.0017 |
| F2RL1 | Mast cells | 502 | 3.30279 | 0.145835 | 0.0010 | 1.996e+07 | 0.0646579 | 0.1472 |
| F2RL1 | Neutrophils | 502 | 7.57088 | 0.320123 | 1.79e-13 | 1.585e+07 | 0.257137 | 5.36e-09 |
| F2RL1 | NK CD56bright cells | 502 | -4.34449 | -0.190359 | 1.69e-05 | 2.545e+07 | -0.192784 | 1.36e-05 |
| F2RL1 | NK CD56dim cells | 502 | -4.50704 | -0.197209 | 8.19e-06 | 2.342e+07 | -0.0976383 | 0.0284 |
| F2RL1 | NK cells | 502 | -1.45489 | -0.0647985 | 0.1463 | 2.198e+07 | -0.0302552 | 0.4978 |
| F2RL1 | pDC | 502 | -7.74658 | -0.326767 | 5.26e-14 | 2.761e+07 | -0.294102 | 1.63e-11 |
| F2RL1 | T cells | 502 | -6.62954 | -0.283731 | 8.71e-11 | 2.559e+07 | -0.199409 | 6.74e-06 |
| F2RL1 | T helper cells | 502 | -1.35234 | -0.0602482 | 0.1769 | 2.105e+07 | 0.0132607 | 0.7664 |
| F2RL1 | Tcm | 502 | 2.77405 | 0.122874 | 0.0057 | 1.819e+07 | 0.147703 | 0.0009 |
| F2RL1 | Tem | 502 | -3.49347 | -0.15406 | 0.0005 | 2.415e+07 | -0.13187 | 0.0030 |
| F2RL1 | TFH | 502 | -2.97369 | -0.131568 | 0.0031 | 2.222e+07 | -0.0413421 | 0.3542 |
| F2RL1 | Tgd | 502 | 7.18912 | 0.305524 | 2.38e-12 | 1.453e+07 | 0.319254 | 2.8e-13 |
| F2RL1 | Th1 cells | 502 | 3.06361 | 0.135475 | 0.0023 | 1.722e+07 | 0.193047 | 1.32e-05 |
| F2RL1 | Th17 cells | 502 | -2.11849 | -0.0941329 | 0.0346 | 2.348e+07 | -0.100368 | 0.0243 |
| F2RL1 | Th2 cells | 502 | 2.0436 | 0.0908334 | 0.0415 | 1.76e+07 | 0.175121 | 7.94e-05 |
| F2RL1 | TReg | 502 | -4.19117 | -0.183872 | 3.28e-05 | 2.299e+07 | -0.0774824 | 0.0823 |

相关系数为正，说明两个变量之间存在正相关关系; 相关系数为负，说明两个变量之间存在负相关关系;

相关系数绝对值代表相关程度，0-0.3代表弱或者不相关; 0.3-0.5代表弱相关; 0.5-0.8代表中等程度相关; 0.8-1代表强相关

相关是否有统计学意义还需要结合p值来查看

方法学

**软件**: R (4.2.1)版本

**R包**: ggplot2[3.3.6]

**处理过程:**

· 对数据中主变量和免疫浸润矩阵数据之间进行相关性分析，分析结果用ggplot2包进行棒棒糖图可视化

**补充说明:**

· 统计方法: Spearman

· 主分子: F2RL1[ENSG00000164251.5]

· 免疫浸润算法: 基于R包-GSVA[1.46.0](Hänzelmann et al., 2013)中提供的ssGSEA算法, 利用Immunity文章(Bindea, Gabriela, et al., 2013)提供的24种免疫细胞的markers来计算对应云端数据的免疫浸润情况，具体24种免疫细胞可查看对应参考文献

· 免疫细胞: aDC[activated DC]; B cells; CD8 T cells; Cytotoxic cells; DC; Eosinophils; iDC[immature DC]; Macrophages; Mast cells; Neutrophils; NK CD56bright cells; NK CD56dim cells; NK cells; pDC[Plasmacytoid DC]; T cells; T helper cells; Tcm[T central memory]; Tem[T effector memory]; TFH[T follicular helper]; Tgd[T gamma delta]; Th1 cells; Th17 cells; Th2 cells; TReg

**参考文献:**

Hänzelmann, Sonja, Robert Castelo, and Justin Guinney. GSVA: gene set variation analysis for microarray and RNA-seq data. BMC bioinformatics 14.1 (2013): 1-15.文献链接

Bindea, Gabriela, et al. Spatiotemporal dynamics of intratumoral immune cells reveal the immune landscape in human cancer. Immunity 39.4 (2013): 782-795.文献链接

**数据:**

· 数据获取: 从TCGA数据库 ( https://portal.gdc.cancer.gov ) 下载并整理TCGA-HNSC(头颈鳞状细胞癌)项目STAR流程的RNAseq数据并提取TPM格式的数据 以及 临床数据

· 数据过滤策略: 去除正常+去除无临床信息

· 数据处理方法: log2(value+1)
