# Supplementary material for: Coagulation factor II receptor-like 1 as a prognostic and immuno-modulatory factor in head and neck squamous cell carcinoma
Source: PeerJ. 2026 Mar 18;14:e20970. doi: 10.7717/peerj.20970 (PMC13005615; doi:10.7717/peerj.20970)
Supplement: Supplemental Information 5 [file peerj-14-20970-s005.zip › Figure 5/L/reports.html]

仙桃-非配对样本(分组)-在线分析报告


非配对样本(分组)-在线分析报告

导出时间: 2024-05-20 21:09:10

目录

- 非配对样本(分组)

- 统计描述

- 异常值分析

- 正态性检验

- 方差齐性检验

- Mann-Whitney U检验(Wilcoxon rank sum test)

- 方法学

非配对样本(分组)

非配对样本(分组)

**非配对样本图(分组)**: 基于公共数据直接分析分子在两组之间的差别

当前所选的统计方法: **Mann-Whitney U检验(Wilcoxon rank sum test)**

**注意**: 统计要求每组样本都要满足3个样本以上，并且每组样本的方差不能为0，如果不满足条件，就不会进行统计分析

**补充说明**: 该模块会根据数据情况，自动选择合适的统计方法进行统计分析，其中统计方法涵盖:

- 两组: T test(满足正态+方差齐) | Welch t' test(满足正态+不满足方差齐性) | Wilcoxon rank sum test(不满足正态, 非参数检验)

统计描述

各个组常见「统计描述指标」

| 组别1 | 组别2 | 数目 | 最小值 | 最大值 | 中位数(Median) | 四分位距(IQR) | 下四分位 | 上四分位 | 均值(Mean) | 标准差(SD) | 标准误(SE) |
| --- | --- | --- | --- | --- | --- | --- | --- | --- | --- | --- | --- |
| PDCD1 | Normal | 44 | 0.45807 | 2.9776 | 1.4801 | 1.1498 | 0.98791 | 2.1377 | 1.5298 | 0.67462 | 0.1017 |
| PDCD1 | Tumor | 502 | 0 | 5.6036 | 1.9413 | 1.7686 | 1.1766 | 2.9452 | 2.1151 | 1.1642 | 0.051962 |
| CD274 | Normal | 44 | 1.0205 | 4.1372 | 2.153 | 1.0549 | 1.7951 | 2.8501 | 2.2736 | 0.82822 | 0.12486 |
| CD274 | Tumor | 502 | 0.43531 | 8.311 | 3.1545 | 1.9133 | 2.2307 | 4.1439 | 3.2545 | 1.3924 | 0.062146 |
| CTLA4 | Normal | 44 | 0.062951 | 2.9755 | 1.0305 | 0.93702 | 0.47602 | 1.413 | 1.0827 | 0.74219 | 0.11189 |
| CTLA4 | Tumor | 502 | 0.042644 | 6.8565 | 2.6238 | 1.6079 | 1.779 | 3.3869 | 2.5906 | 1.0983 | 0.049018 |

异常值分析

离群值 = Q1(下四分位) - 1.5\*IQR(四分位间距) 或者 Q3(上四分位) + 1.5\*IQR(四分位间距)

异常值 = Q1(下四分位) - 3.0\*IQR(四分位间距) 或者 Q3(上四分位) + 3.0\*IQR(四分位间距)

| 组别1 | 组别2 | 离群值 | 异常值 |
| --- | --- | --- | --- |
| PDCD1 | Tumor | 5.60355810278417 |  |
| CD274 | Tumor | 8.31098760647849 |  |
| CTLA4 | Normal | 2.97553847887585 |  |
| CTLA4 | Tumor | 6.85650271930484 |  |

各组离群值和异常值如上所示，如数据确认非人为记录错误，可不进行处理

正态性检验

检验方法: Shapiro-Wilk normality test

| 组别1 | 组别2 | 自由度(df) | 统计量 | p值 |
| --- | --- | --- | --- | --- |
| PDCD1 | Tumor | 501 | 0.97217 | 3.59e-08 |
| PDCD1 | Normal | 43 | 0.95484 | 0.0834 |
| CD274 | Tumor | 501 | 0.98507 | 5.04e-05 |
| CD274 | Normal | 43 | 0.94505 | 0.0361 |
| CTLA4 | Tumor | 501 | 0.99248 | 0.0126 |
| CTLA4 | Normal | 43 | 0.93746 | 0.0192 |

正态性检验结果显示，存在有不满足正态分布的分组(P < 0.05)，建议选择用 非参数检验的方法

方差齐性检验

检验方法: Levene's test

· Base on Mean

| 组别 | 自由度1(df1) | 自由度2(df2) | 统计量 | p值 |
| --- | --- | --- | --- | --- |
| PDCD1 | 1 | 544 | 16.79 | 4.81e-05 |
| CD274 | 1 | 544 | 13.398 | 0.0003 |
| CTLA4 | 1 | 544 | 11.437 | 0.0008 |

方差齐性检验显示，各组观测变量的方差不相等(P < 0.05)，建议选择用校正方法

Mann-Whitney U检验(Wilcoxon rank sum test)

| 组别 | 组别I | 组别J | 统计量 | 差值(J-I) | 置信区间(95%CI) | p值 |
| --- | --- | --- | --- | --- | --- | --- |
| PDCD1 | Normal | Tumor | 7871 | 0.49136 | 0.18898 - 0.8126 | 0.0016 |
| CD274 | Normal | Tumor | 6230.5 | 0.93319 | 0.56902 - 1.2995 | 1.61e-06 |
| CTLA4 | Normal | Tumor | 2924 | 1.5428 | 1.2192 - 1.852 | 5.88e-16 |

方法学

**软件**: R (4.2.1)版本

**R包**: ggplot2[3.3.6], stats[4.2.1], car[3.1-0]

**处理过程:**

· 根据数据格式特征情况选择合适的统计方法进行统计(stats包以及car包)(如果不满足统计要求将不会进行统计分析)，用ggplot2包对数据进行可视化

**补充说明:**

· 统计方法: Wilcoxon rank sum test

· 所选分子: PDCD1[ENSG00000188389.11], CD274[ENSG00000120217.14], CTLA4[ENSG00000163599.17]

**数据:**

· 表达数据获取: 从TCGA数据库 ( https://portal.gdc.cancer.gov ) 下载并整理TCGA-HNSC(头颈鳞状细胞癌)项目STAR流程的RNAseq数据并提取TPM格式的数据

· 数据过滤策略: 去除无临床信息+去除重复

· 数据处理方法: log2(value+1)
