# Supplementary figures and images for: Coagulation factor II receptor-like 1 as a prognostic and immuno-modulatory factor in head and neck squamous cell carcinoma (part 2 of 2)
Source: PeerJ. 2026 Mar 18;14:e20970. doi: 10.7717/peerj.20970 (PMC13005615; doi:10.7717/peerj.20970)

# IGF2BP2

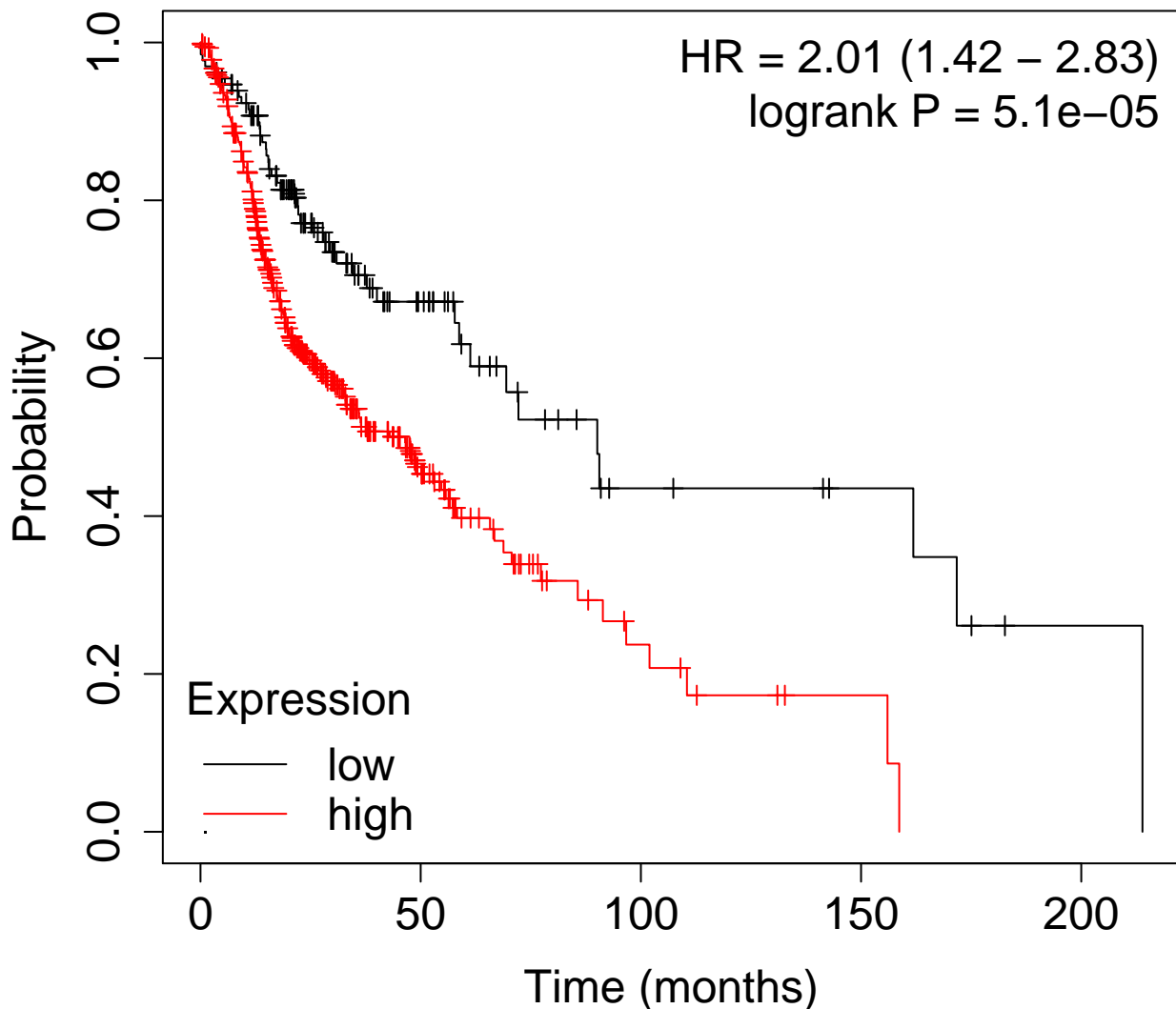

Number at risk

|      |     |    |   |   |   |
|------|-----|----|---|---|---|
| low  | 132 | 33 | 8 | 5 | 1 |
| high | 367 | 53 | 8 | 2 | 0 |

Supplement: Supplemental Information 5 [file peerj-14-20970-s005.zip › Figure 7/N-S/032223_250200_6641876f3d150_IGF2BP2.pdf]

# IGF2BP3

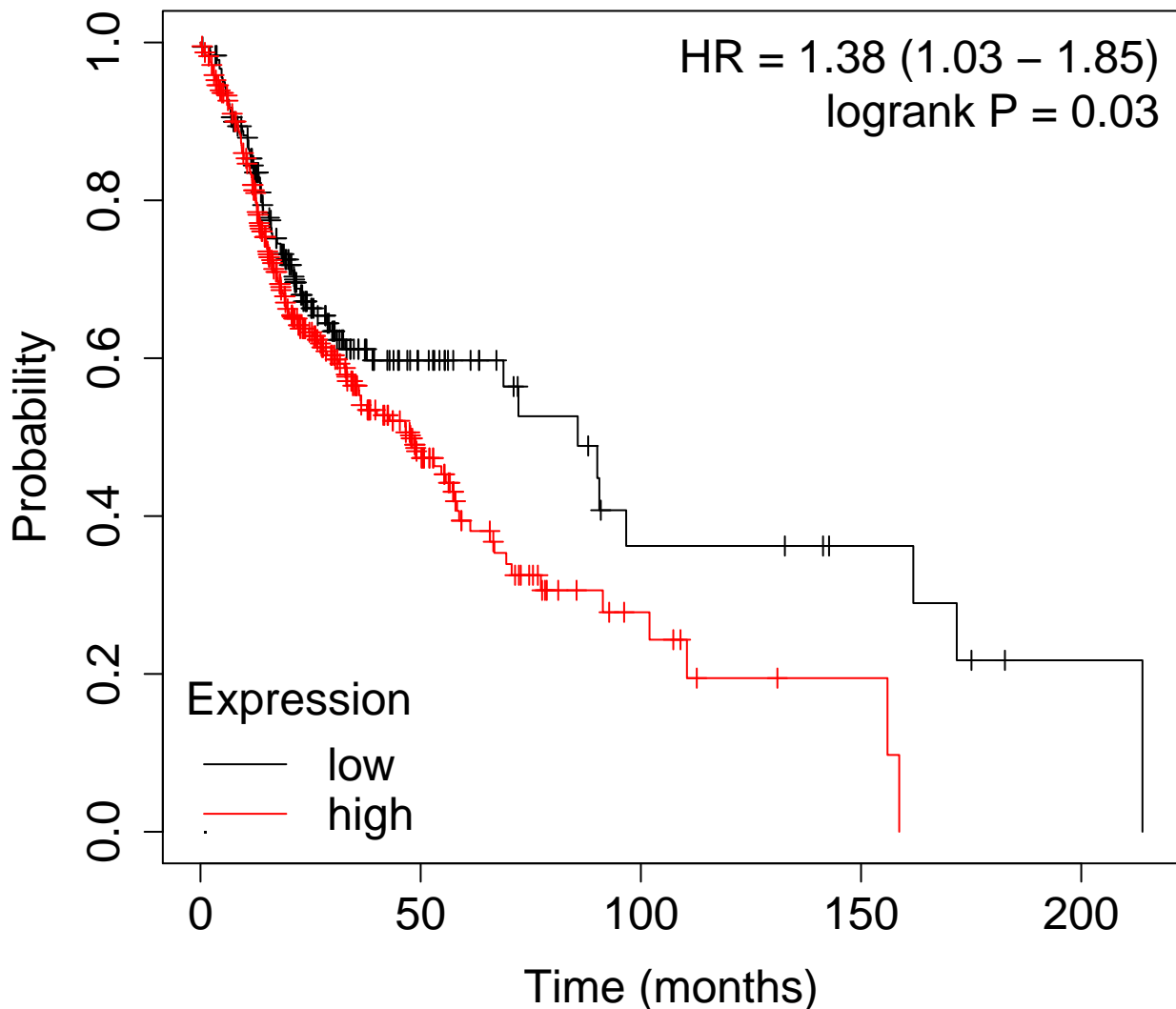

Number at risk

|      |     |    |   |   |   |
|------|-----|----|---|---|---|
| low  | 182 | 30 | 8 | 5 | 1 |
| high | 317 | 56 | 8 | 2 | 0 |

Supplement: Supplemental Information 5 [file peerj-14-20970-s005.zip › Figure 7/N-S/032319_693600_664187a7a9565_IGF2BP3.pdf]

# YTHDC2

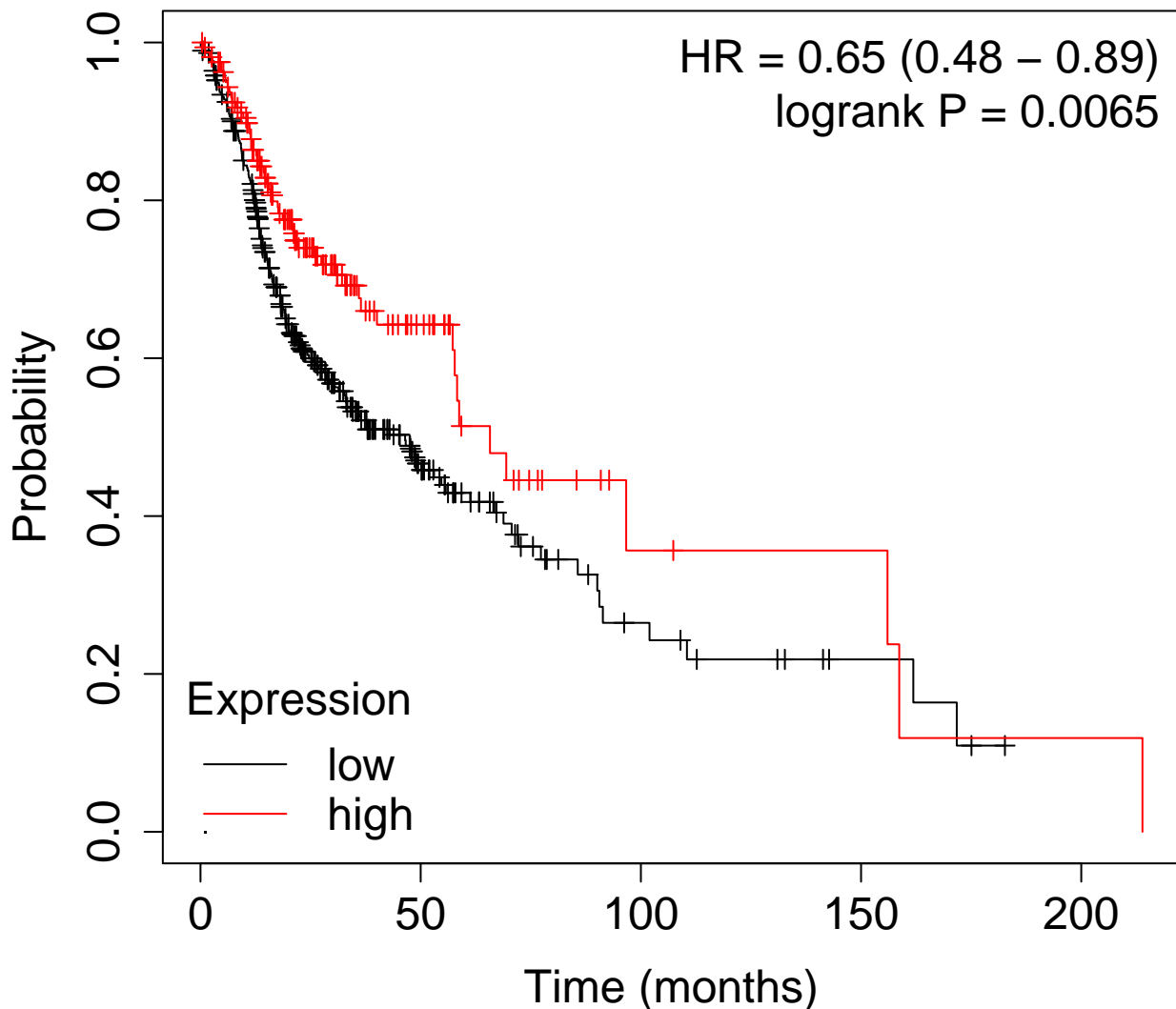

Number at risk

low  
high

336  
163

57  
29

12  
4

4  
3

0  
1

Supplement: Supplemental Information 5 [file peerj-14-20970-s005.zip › Figure 7/N-S/032533_078800_6641882d13404_YTHDC2.pdf]

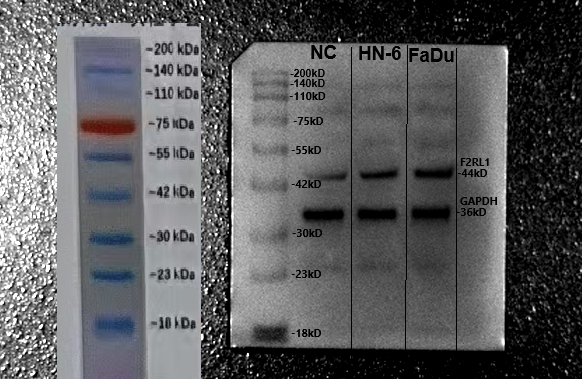

Supplement: Supplemental Information 7 [file peerj-14-20970-s007.tif]
