# Supplementary figures and images for: Coagulation factor II receptor-like 1 as a prognostic and immuno-modulatory factor in head and neck squamous cell carcinoma (part 1 of 2)
Source: PeerJ. 2026 Mar 18;14:e20970. doi: 10.7717/peerj.20970 (PMC13005615; doi:10.7717/peerj.20970)

The expression of F2RL1  
Log<sub>2</sub> (TPM+1)

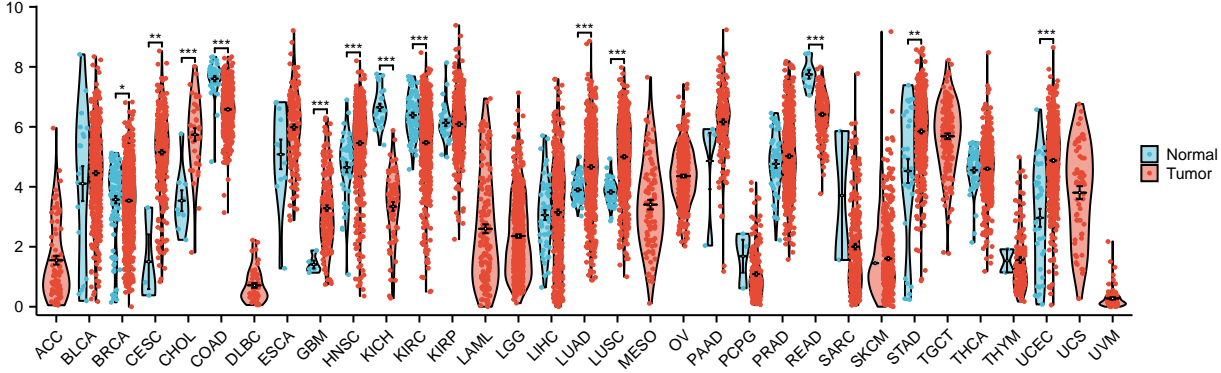

Supplement: Supplemental Information 5 [file peerj-14-20970-s005.zip › Figure 1/A/F2RL1-Comparison of subgroups (pan-cancer)/output/Group comparison.pdf]

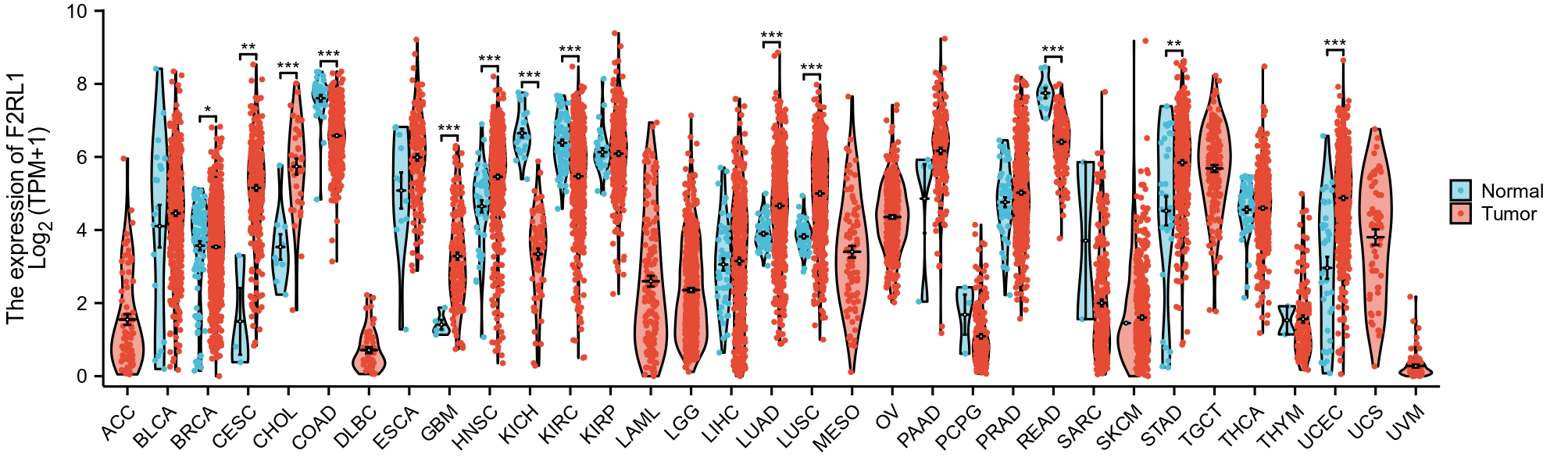

Supplement: Supplemental Information 5 [file peerj-14-20970-s005.zip › Figure 1/A/F2RL1-Comparison of subgroups (pan-cancer)/output/Group comparison.png]

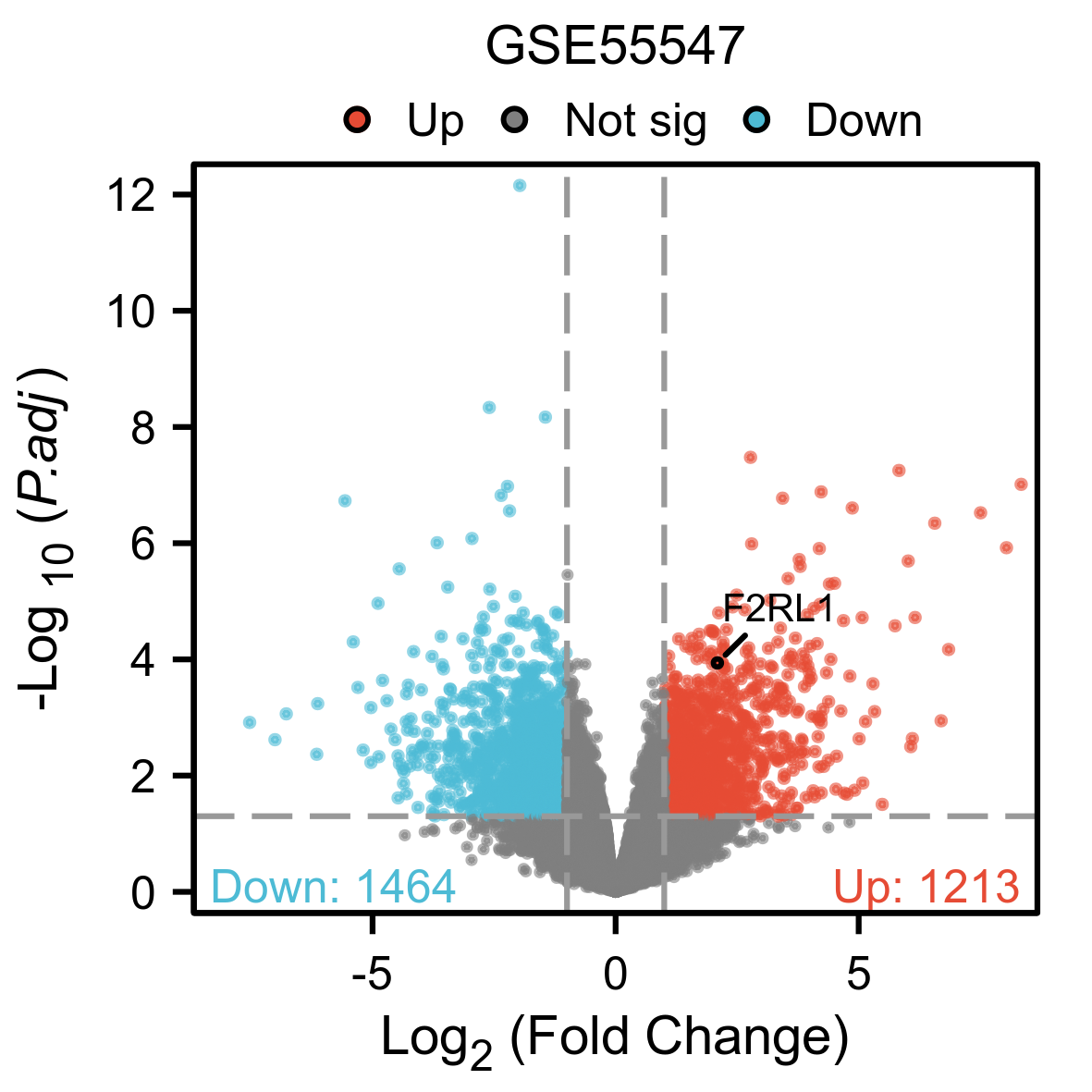

Supplement: Supplemental Information 5 [file peerj-14-20970-s005.zip › Figure 1/B-D/GSE55547.tiff]

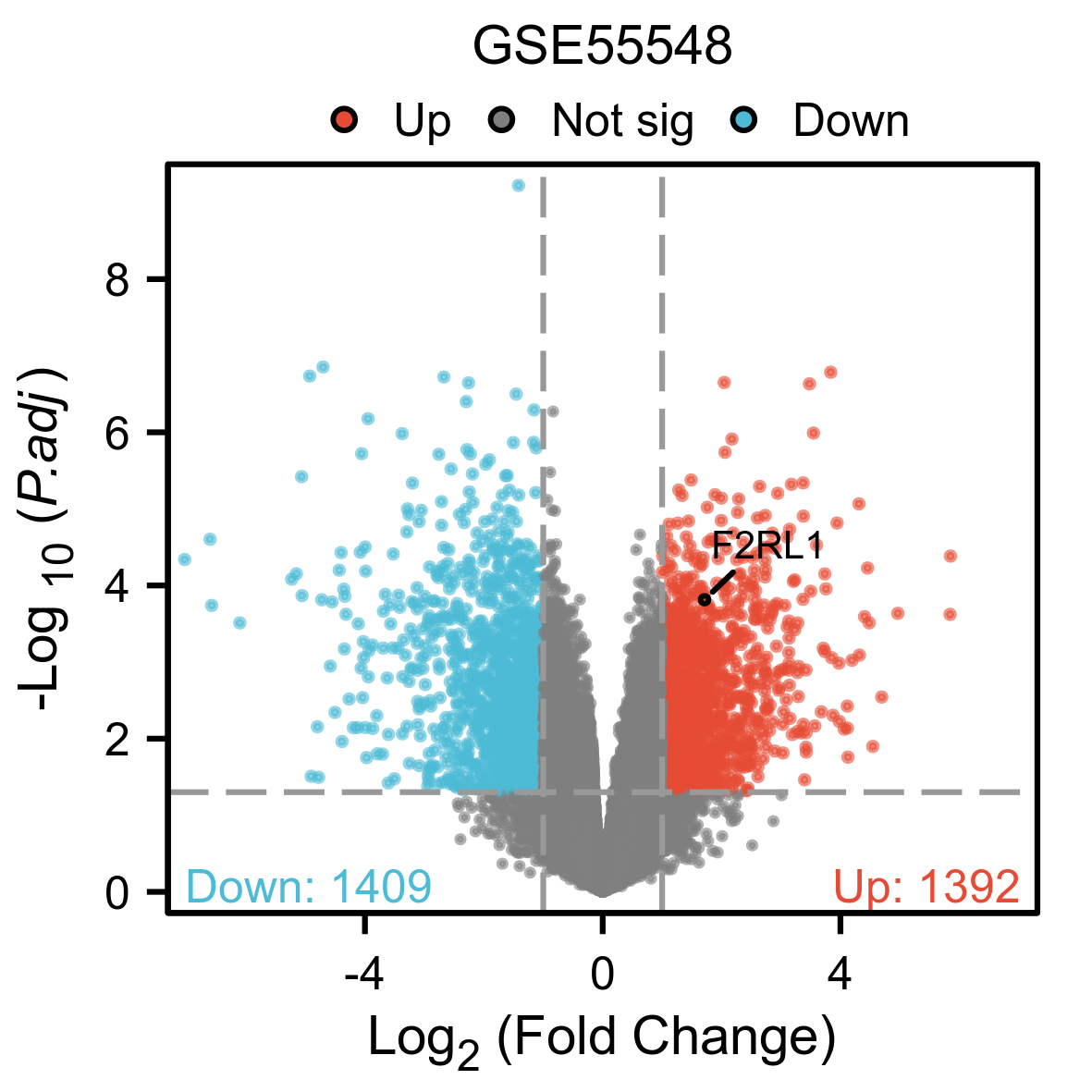

Supplement: Supplemental Information 5 [file peerj-14-20970-s005.zip › Figure 1/B-D/GSE55548.tiff]

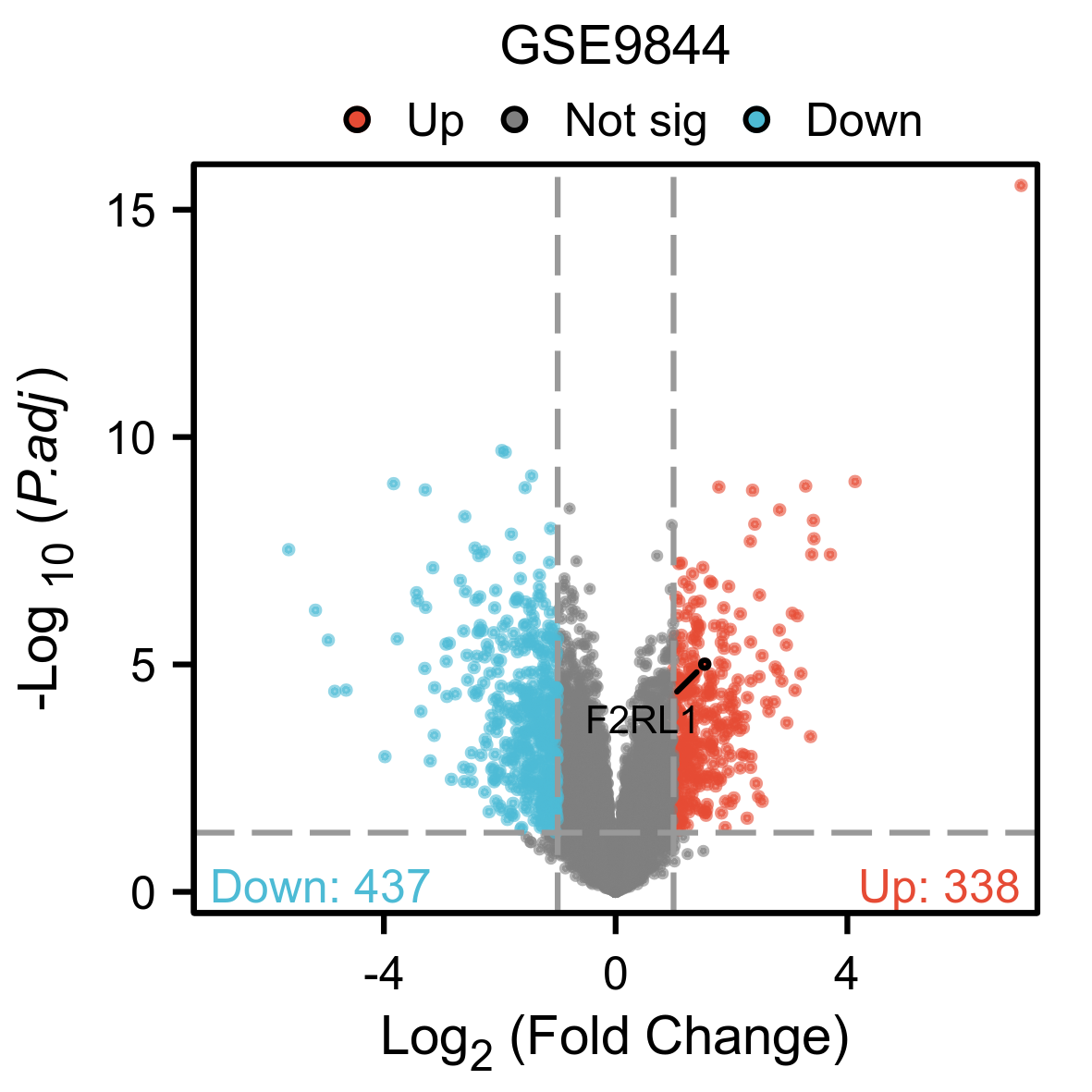

Supplement: Supplemental Information 5 [file peerj-14-20970-s005.zip › Figure 1/B-D/GSE9844.tiff]

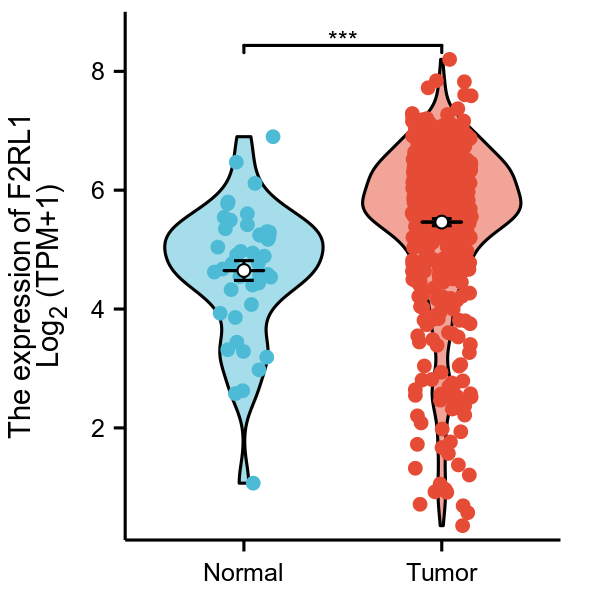

Supplement: Supplemental Information 5 [file peerj-14-20970-s005.zip › Figure 1/E/Unpaired samples plot expression differences/output/Unpaired sample.png]

The expression of F2RL1  
 $\text{Log}_2(\text{TPM}+1)$

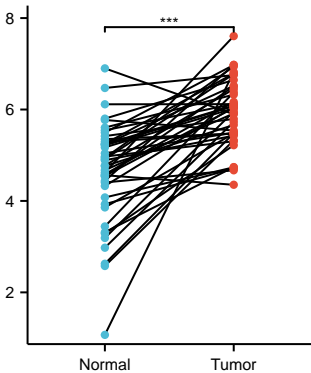

Supplement: Supplemental Information 5 [file peerj-14-20970-s005.zip › Figure 1/F/Differences between paired samples/output/paired sample.pdf]

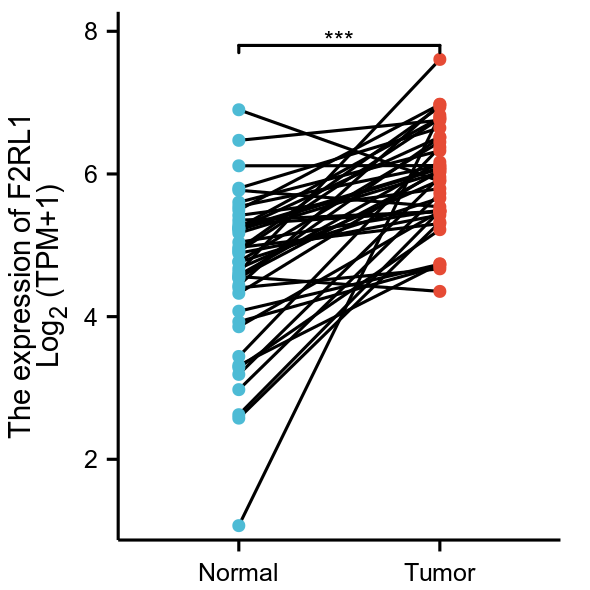

Supplement: Supplemental Information 5 [file peerj-14-20970-s005.zip › Figure 1/F/Differences between paired samples/output/paired sample.png]

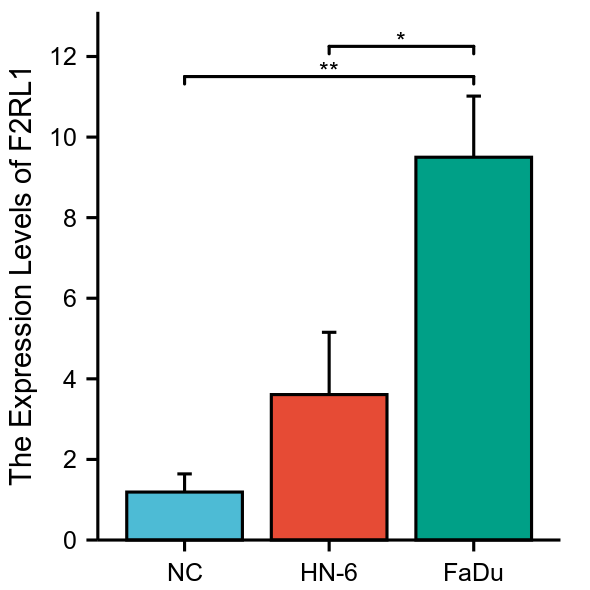

Supplement: Supplemental Information 5 [file peerj-14-20970-s005.zip › Figure 1/G/output/qPCR.png]

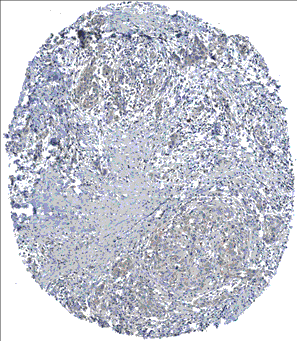

Supplement: Supplemental Information 5 [file peerj-14-20970-s005.zip › Figure 1/H-L/pic/(T-51000) Squamous cell carcinoma, NOS (M-80703)0_已压缩.png]

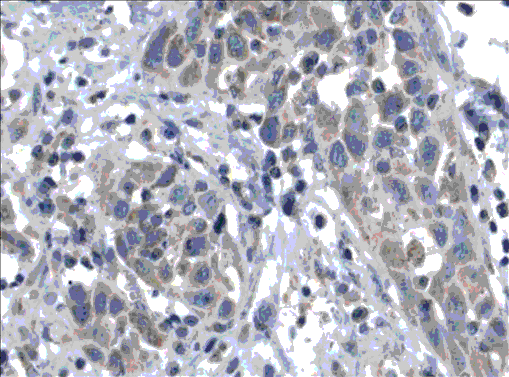

Supplement: Supplemental Information 5 [file peerj-14-20970-s005.zip › Figure 1/H-L/pic/(T-51000) Squamous cell carcinoma, NOS (M-80703)1_已压缩.png]

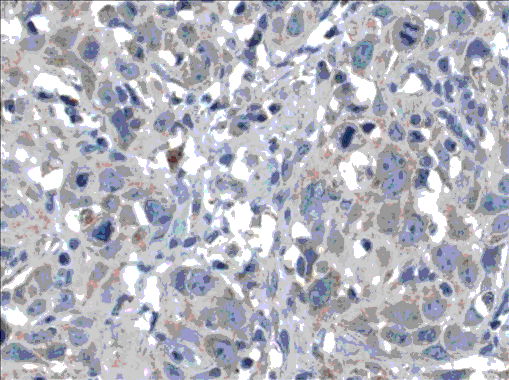

Supplement: Supplemental Information 5 [file peerj-14-20970-s005.zip › Figure 1/H-L/pic/(T-51000) Squamous cell carcinoma, NOS (M-80703)2_已压缩.png]

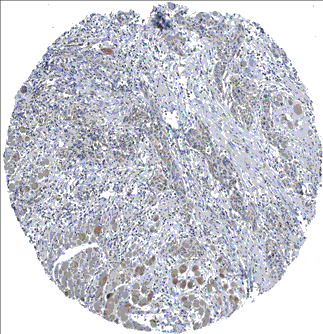

Supplement: Supplemental Information 5 [file peerj-14-20970-s005.zip › Figure 1/H-L/pic/(T-51000) Squamous cell carcinoma, NOS (M-80703)_已压缩.png]

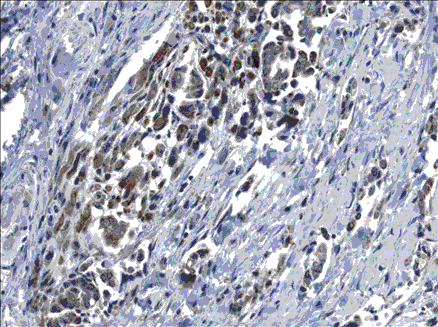

Supplement: Supplemental Information 5 [file peerj-14-20970-s005.zip › Figure 1/H-L/pic/(T-55100) Adenocarcinoma, NOS (M-81403)1_已压缩.png]

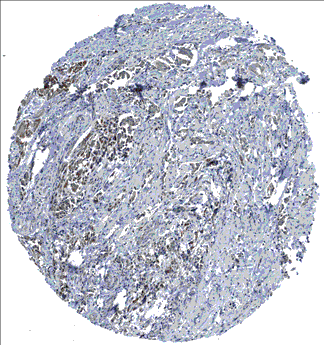

Supplement: Supplemental Information 5 [file peerj-14-20970-s005.zip › Figure 1/H-L/pic/(T-55100) Adenocarcinoma, NOS (M-81403)_已压缩.png]

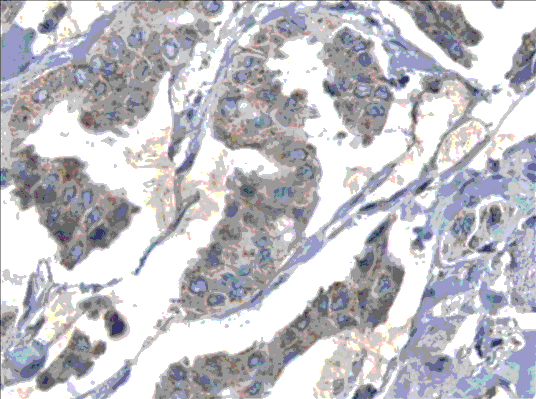

Supplement: Supplemental Information 5 [file peerj-14-20970-s005.zip › Figure 1/H-L/pic/(T-55100)Adenocarcinoma, NOS (M-81403)1_已压缩.png]

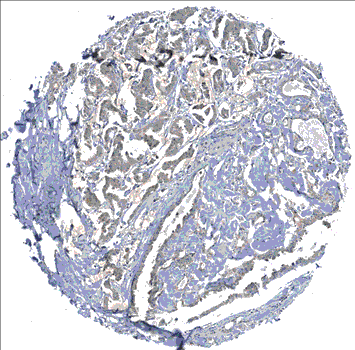

Supplement: Supplemental Information 5 [file peerj-14-20970-s005.zip › Figure 1/H-L/pic/Head-Neck (T-Y0000) (T-Y0000)Salivary gland (T-55100)_已压缩.png]

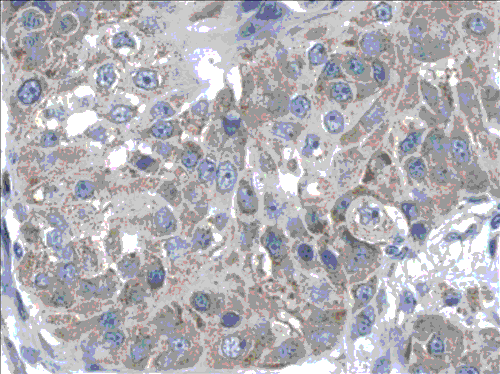

Supplement: Supplemental Information 5 [file peerj-14-20970-s005.zip › Figure 1/H-L/pic/NOS (M-80703)1_已压缩.png]

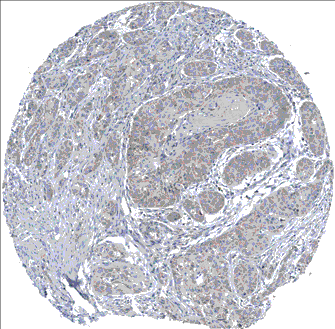

Supplement: Supplemental Information 5 [file peerj-14-20970-s005.zip › Figure 1/H-L/pic/Skeletal muscle Squamous cell carcinoma, NOSM-80703_已压缩.png]

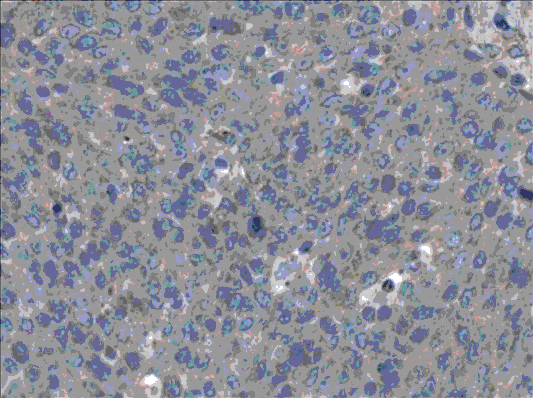

Supplement: Supplemental Information 5 [file peerj-14-20970-s005.zip › Figure 1/H-L/pic/Squamous cell carcinoma, metastatic, NOS (M-80706)1_已压缩.png]

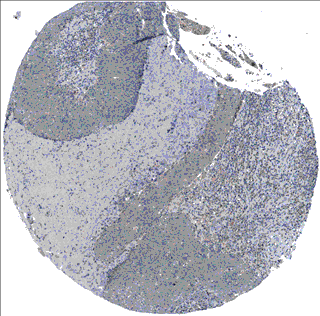

Supplement: Supplemental Information 5 [file peerj-14-20970-s005.zip › Figure 1/H-L/pic/Squamous cell carcinoma, metastatic, NOS (M-80706)_已压缩.png]

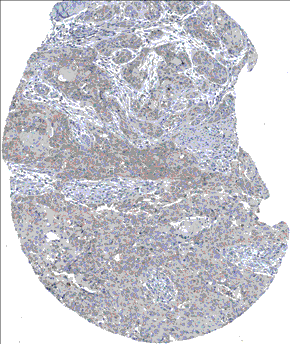

Supplement: Supplemental Information 5 [file peerj-14-20970-s005.zip › Figure 1/H-L/pic/Squamous cell carcinoma, NOS (M-80703)_已压缩.png]

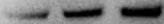

Supplement: Supplemental Information 5 [file peerj-14-20970-s005.zip › Figure 1/M/F2RL1.png]

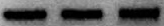

Supplement: Supplemental Information 5 [file peerj-14-20970-s005.zip › Figure 1/M/GAPDH.png]

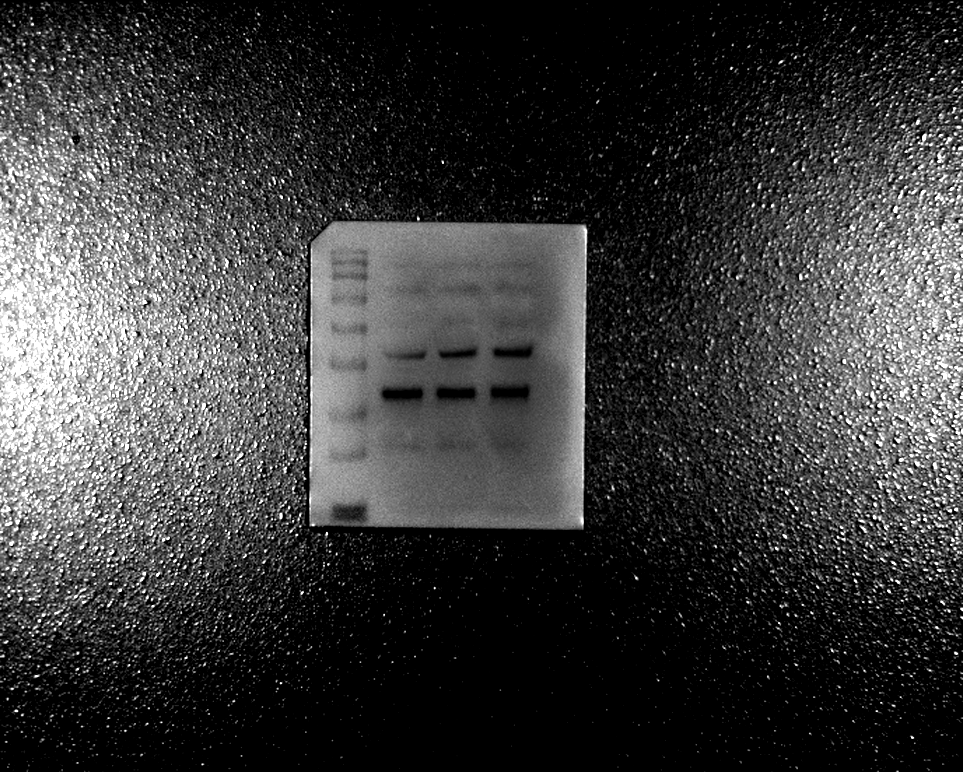

Supplement: Supplemental Information 5 [file peerj-14-20970-s005.zip › Figure 1/M/WB/1.png]

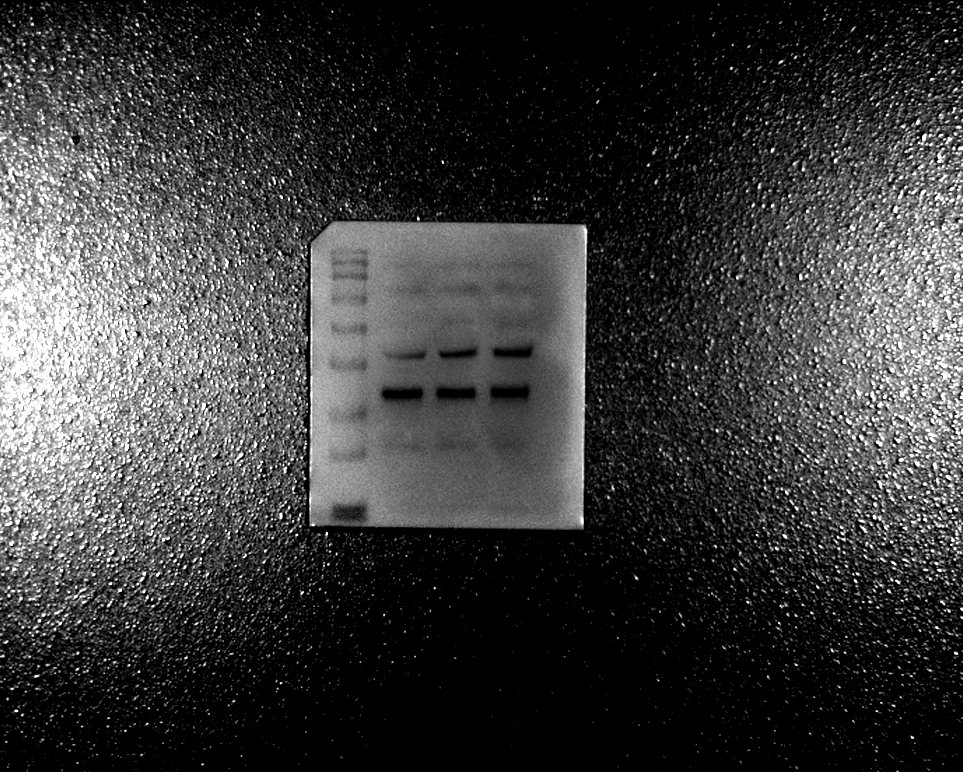

Supplement: Supplemental Information 5 [file peerj-14-20970-s005.zip › Figure 1/M/WB/1.tif]

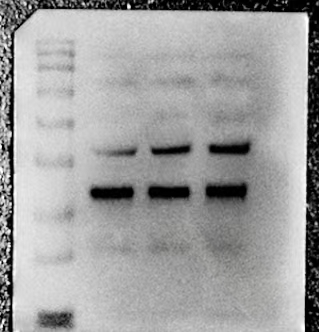

Supplement: Supplemental Information 5 [file peerj-14-20970-s005.zip › Figure 1/M/wb.jpg]

The expression of F2RL1  
 $\text{Log}_2(\text{TPM}+1)$

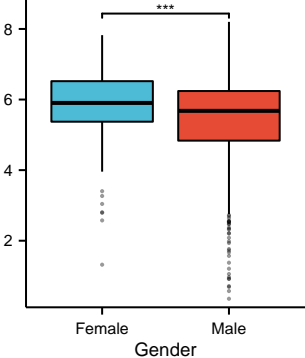

Supplement: Supplemental Information 5 [file peerj-14-20970-s005.zip › Figure 2/A/Clinical significance (Gender)/output/Gender.pdf]

The expression of F2RL1  
 $\text{Log}_2(\text{TPM}+1)$

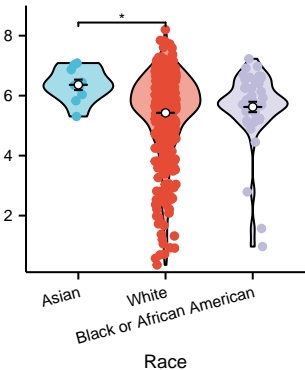

Supplement: Supplemental Information 5 [file peerj-14-20970-s005.zip › Figure 2/B/Clinical significance(Race)/output/Race.pdf]

The expression of F2RL1  
 $\text{Log}_2(\text{TPM}+1)$

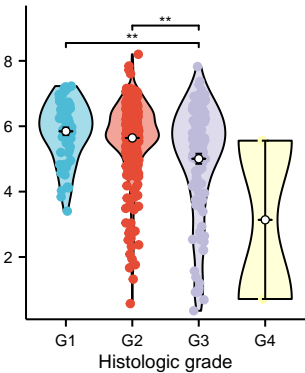

Supplement: Supplemental Information 5 [file peerj-14-20970-s005.zip › Figure 2/C/Clinical significance(Histologic_grade)/output/Histologic grade.pdf]

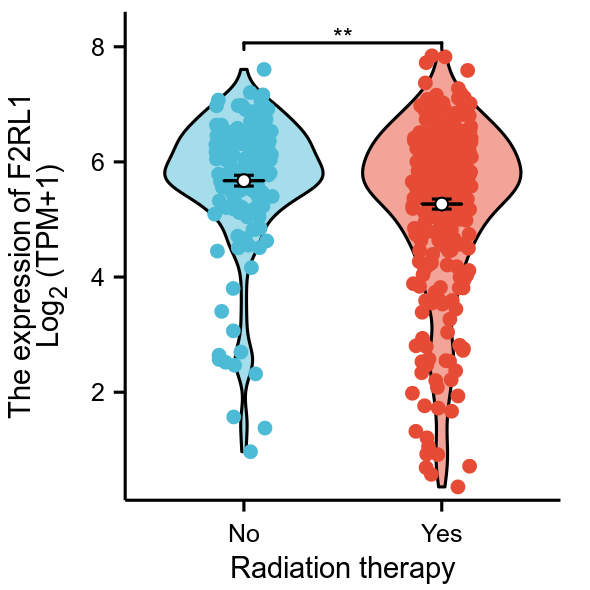

Supplement: Supplemental Information 5 [file peerj-14-20970-s005.zip › Figure 2/D/Clinical significance(Radiation_therapy)/output/Radiation_therapy.png]

The expression of F2RL1  
 $\text{Log}_2(\text{TPM}+1)$

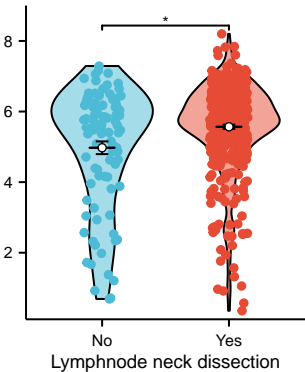

Supplement: Supplemental Information 5 [file peerj-14-20970-s005.zip › Figure 2/E/Clinical significance(Lymphnode_neck_dissection)/output/Lymphnode_neck_dissection.pdf]

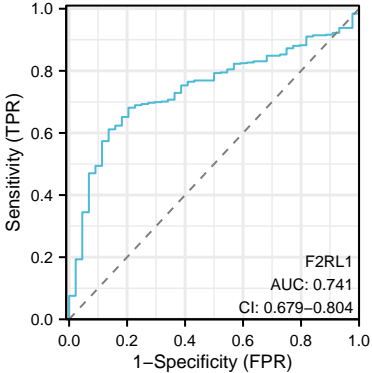

Supplement: Supplemental Information 5 [file peerj-14-20970-s005.zip › Figure 2/G-H/ROC-HNSC-F2RL1-XIANTAO/output/ROC-HNSC-F2RL1.pdf]

Histologic grade: G3&G4 vs G1&G2

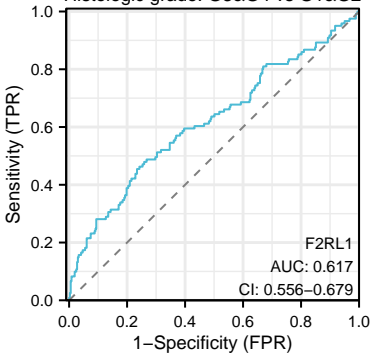

Supplement: Supplemental Information 5 [file peerj-14-20970-s005.zip › Figure 2/G-H/ROC-HNSC-F2RL1-XIANTAO-Histologic-G3+G4vsG1+G2/output/ROC-HNSC-F2RL1-XIANTAO-Histologic-G3+G4vsG1+G2.pdf]

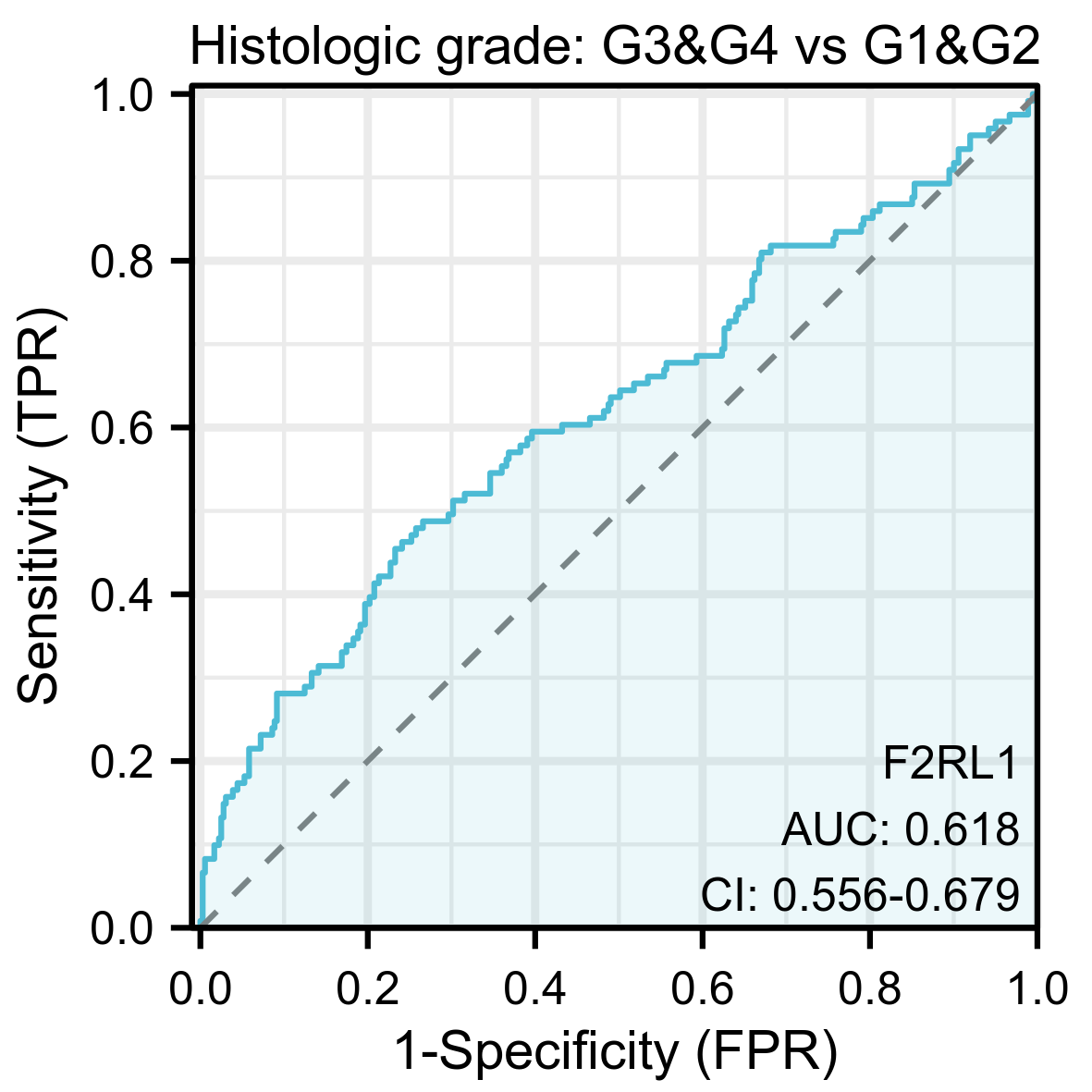

Supplement: Supplemental Information 5 [file peerj-14-20970-s005.zip › Figure 2/G-H/ROC-HNSC-F2RL1-XIANTAO-Histologic-G3+G4vsG1+G2.tiff]

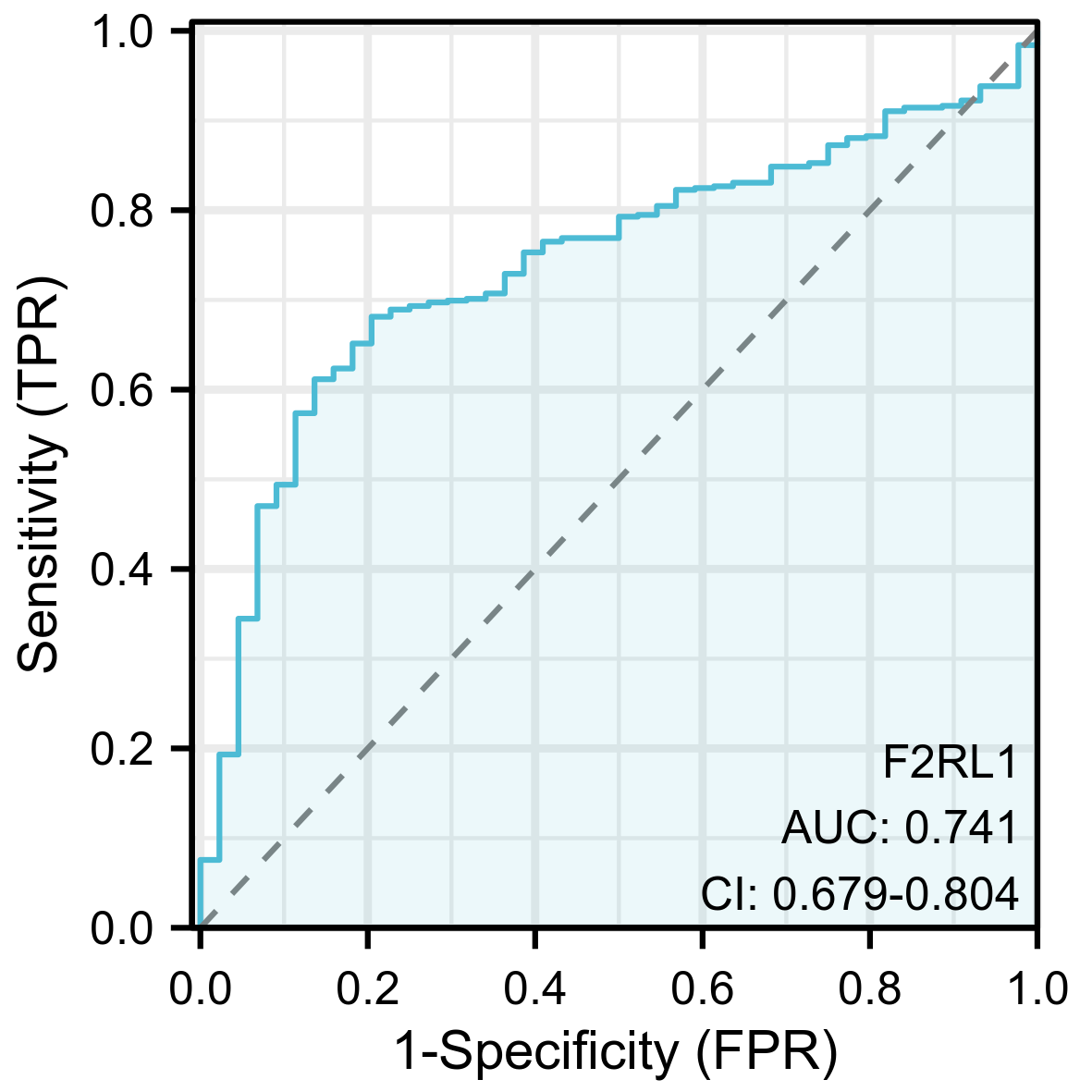

Supplement: Supplemental Information 5 [file peerj-14-20970-s005.zip › Figure 2/G-H/ROC-HNSC-F2RL1-XIANTAO.tiff]

Observed fraction survival probability

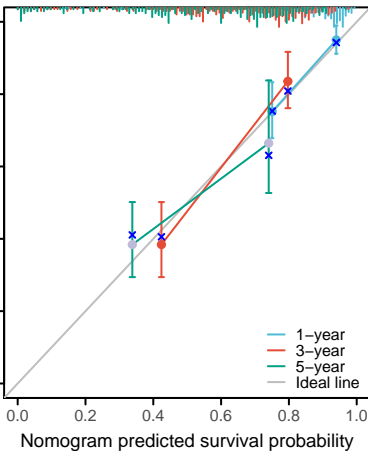

Supplement: Supplemental Information 5 [file peerj-14-20970-s005.zip › Figure 2/I,F/calibration curve/output/calibration curve.pdf]

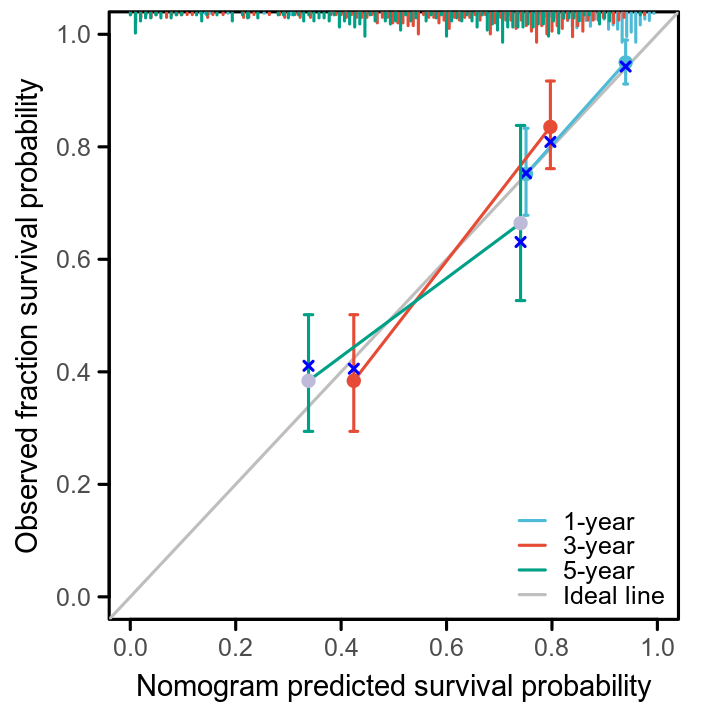

Supplement: Supplemental Information 5 [file peerj-14-20970-s005.zip › Figure 2/I,F/calibration curve.png]

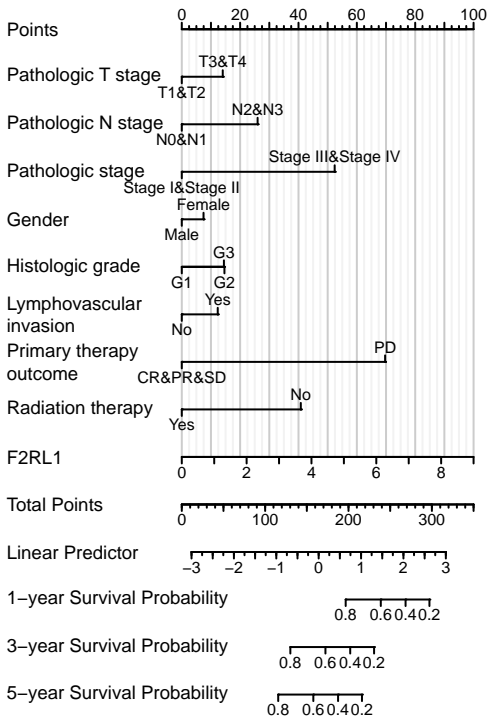

Supplement: Supplemental Information 5 [file peerj-14-20970-s005.zip › Figure 2/I,F/F2RL1-HNSC-Nomograms/output/F2RL1-HNSC-Nomograms.pdf]

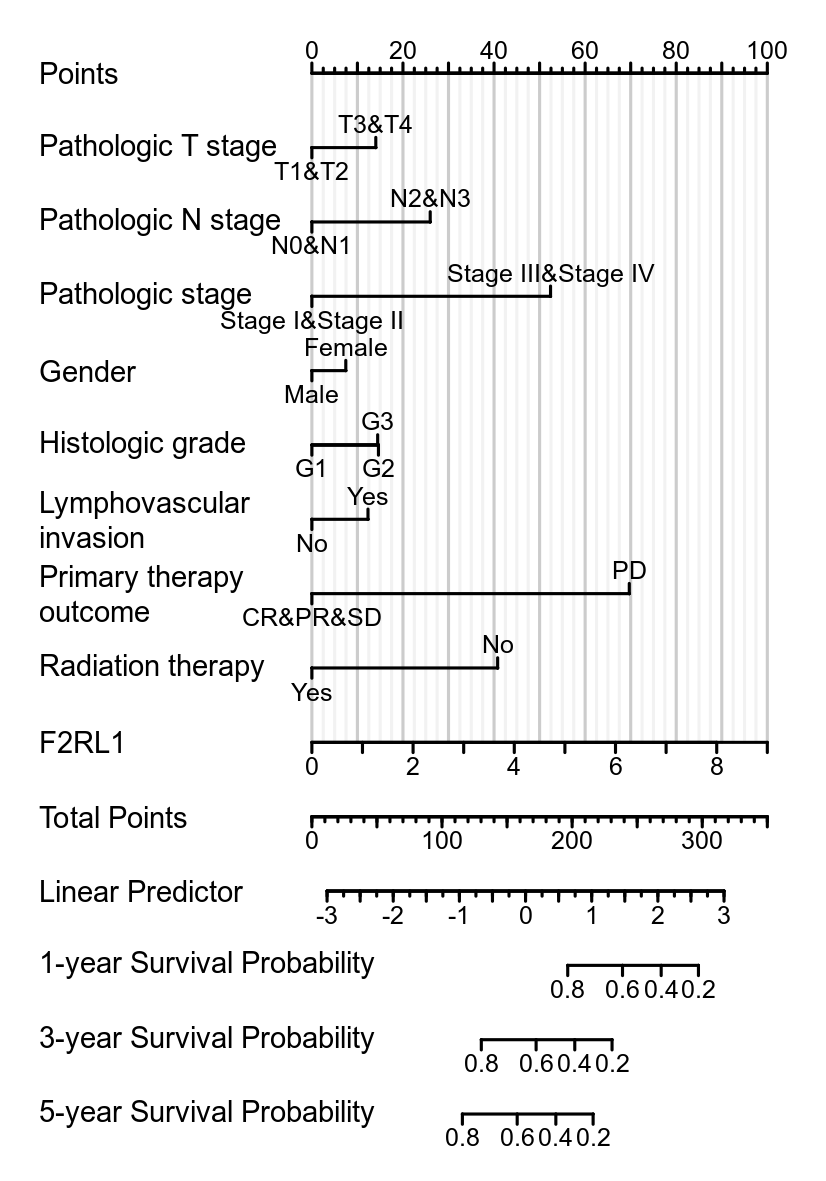

Supplement: Supplemental Information 5 [file peerj-14-20970-s005.zip › Figure 2/I,F/F2RL1-HNSC-Nomograms.png]

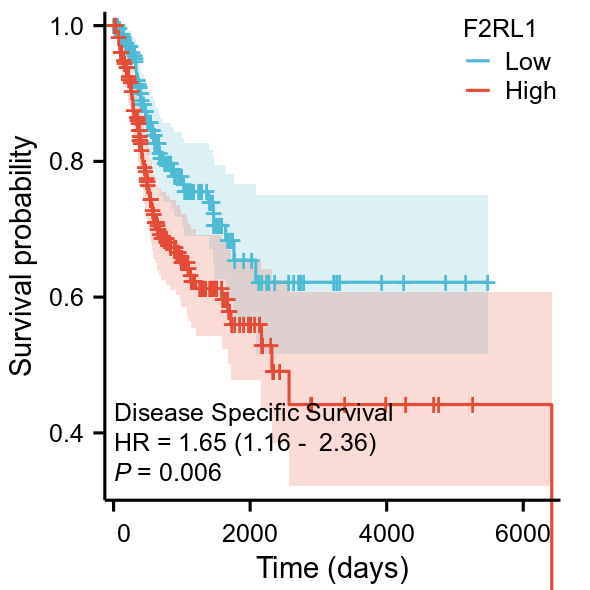

Supplement: Supplemental Information 5 [file peerj-14-20970-s005.zip › Figure 2/J-K/KM-NHSC-DSS-F2RL1-XIANTAO/output/KM-NHSC-DSS-F2RL1-XIANTAO.png]

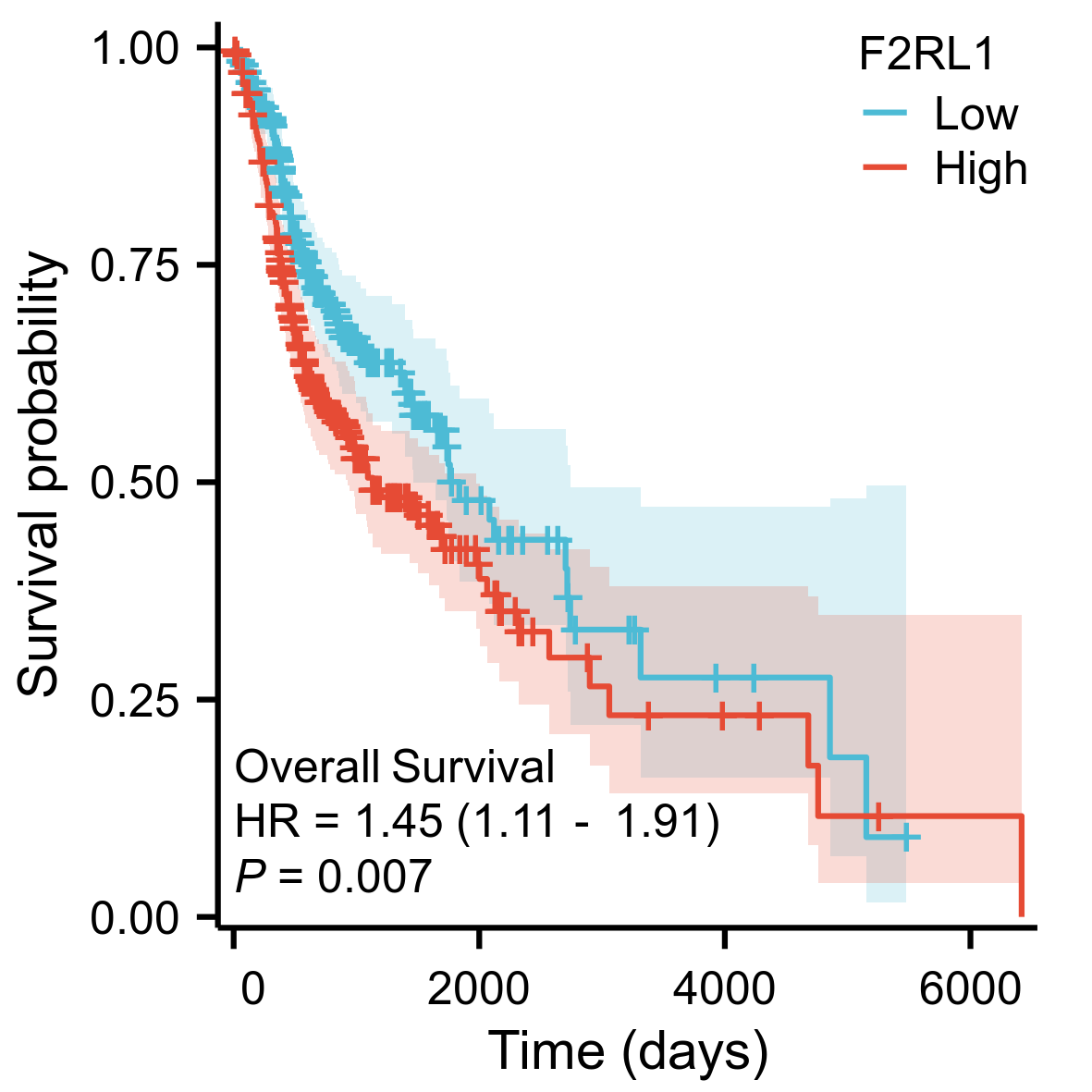

Supplement: Supplemental Information 5 [file peerj-14-20970-s005.zip › Figure 2/J-K/KM-NHSC-OS-F2RL1-XIANTAO.tiff]

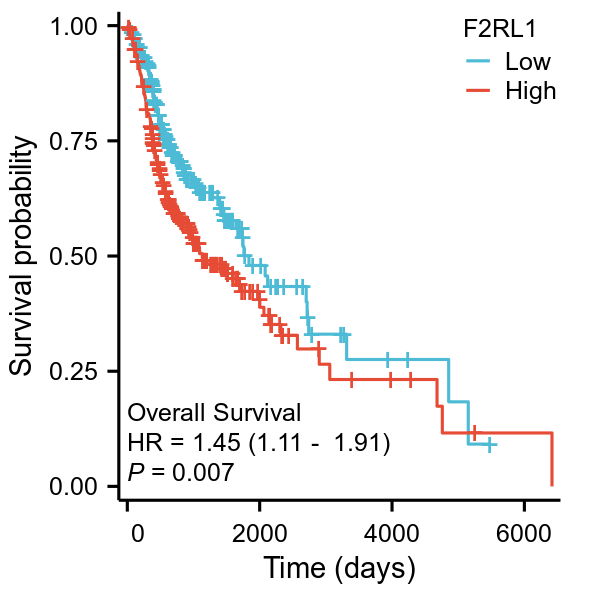

Supplement: Supplemental Information 5 [file peerj-14-20970-s005.zip › Figure 2/J-K/NHSC-OS-F2RL1-XIANTAO/output/KM-NHSC-OS-F2RL1-XIANTAO.png]

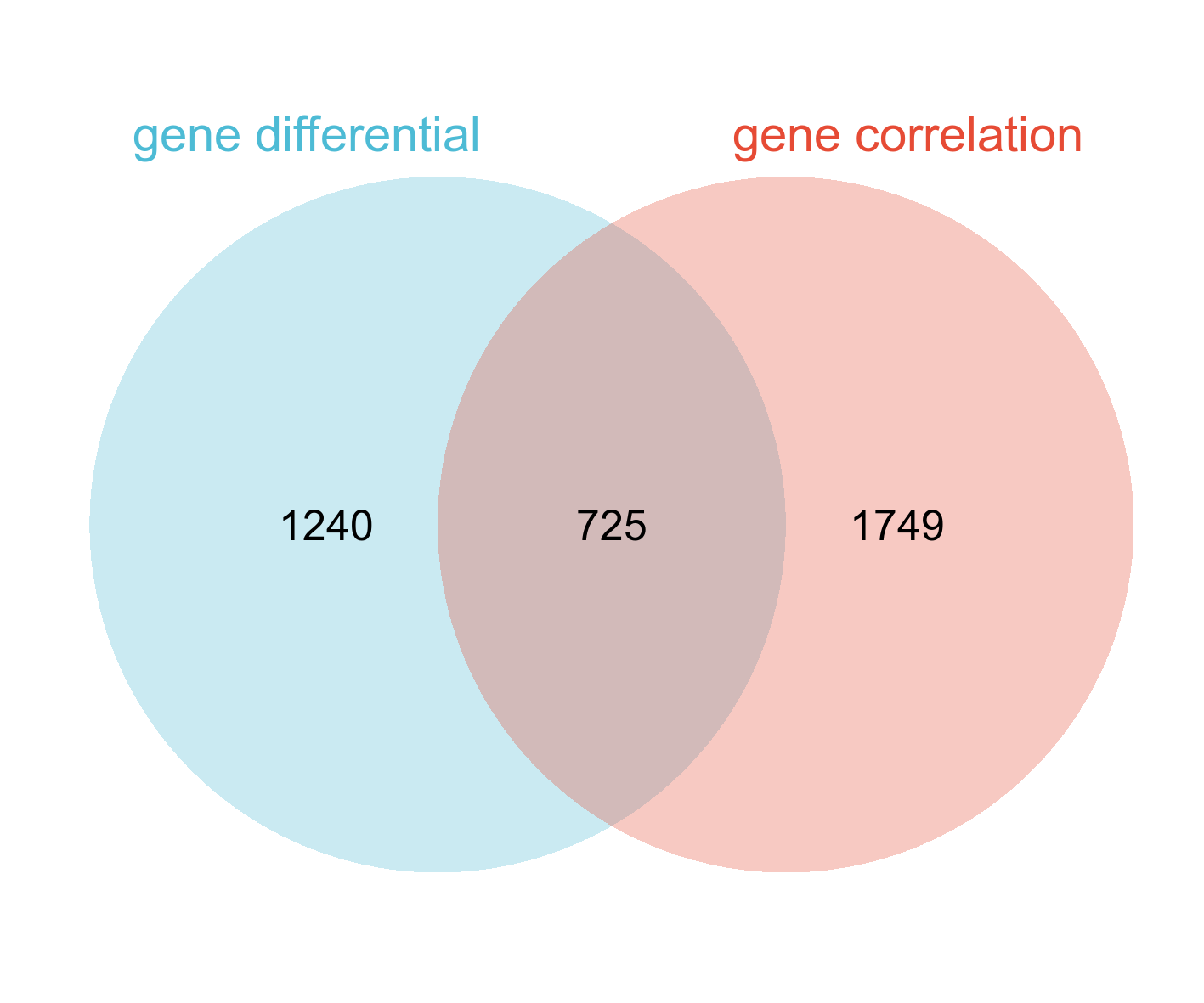

Supplement: Supplemental Information 5 [file peerj-14-20970-s005.zip › Figure 3/A/Single gene correlation+differential.tiff]

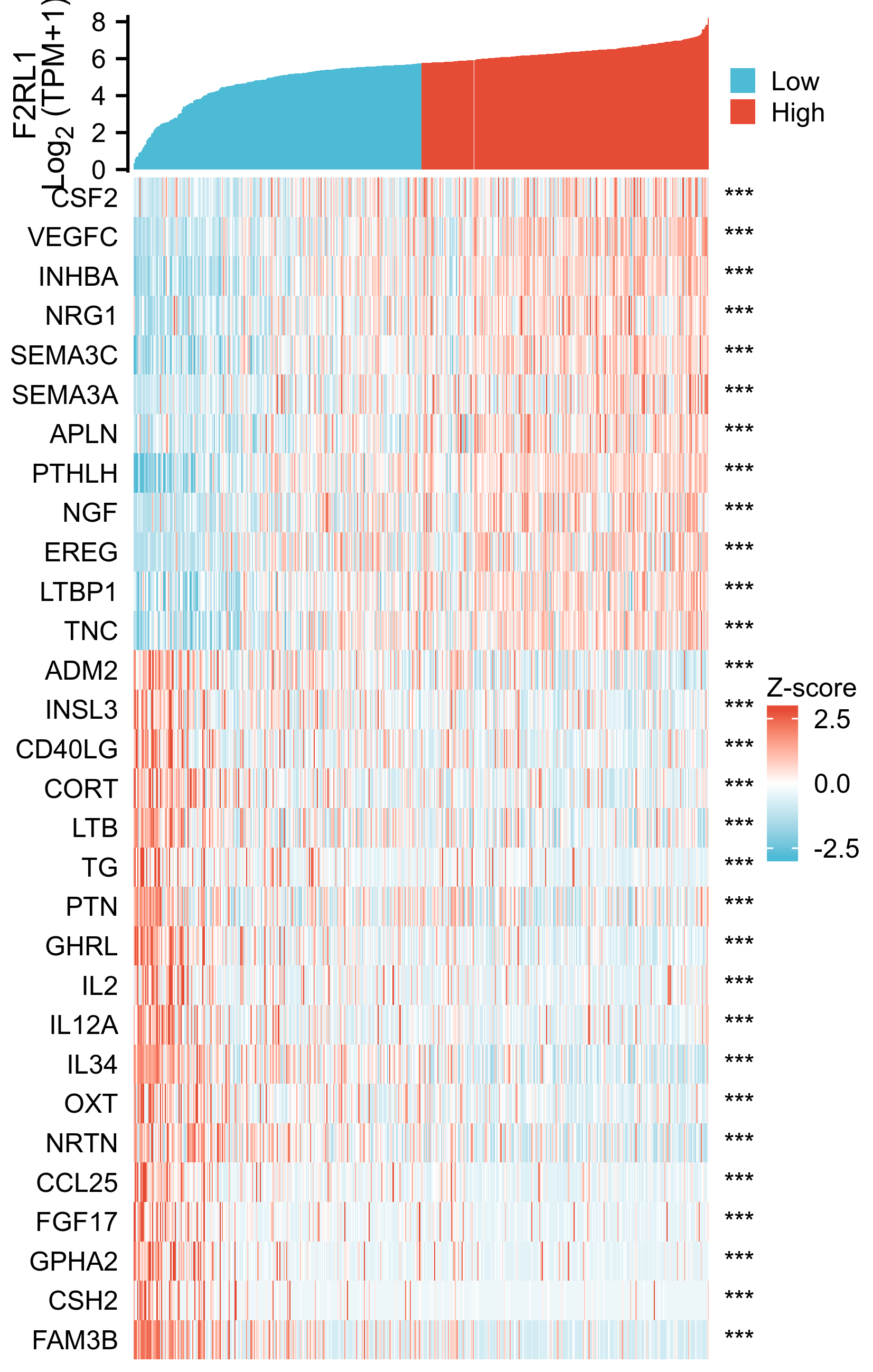

Supplement: Supplemental Information 5 [file peerj-14-20970-s005.zip › Figure 3/B/DEmRNA+F2RL1 Correlated Gene+Imm Gene(U).tiff]

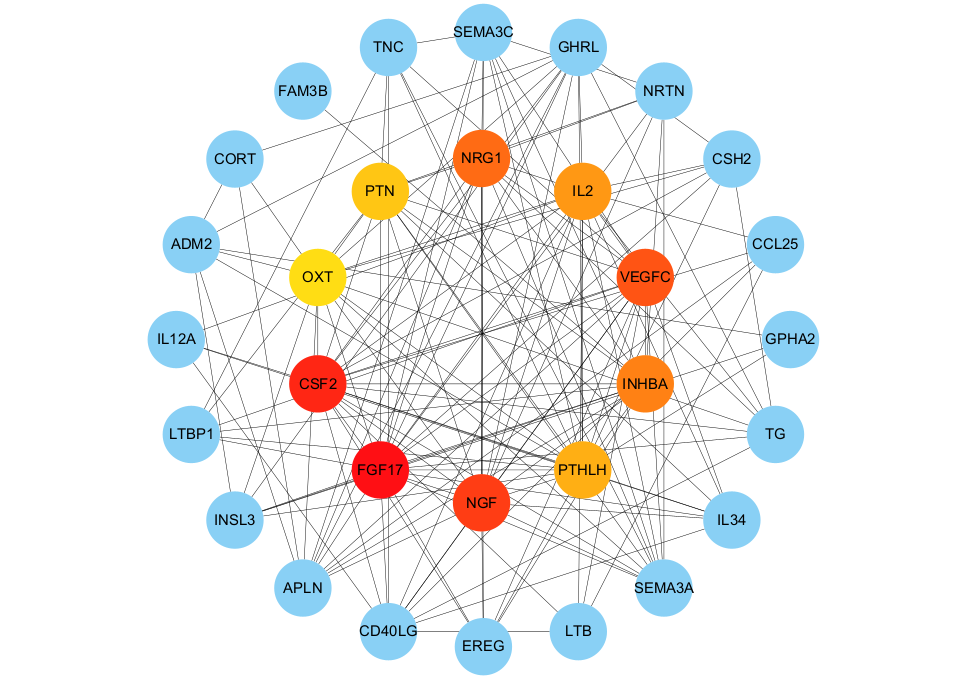

Supplement: Supplemental Information 5 [file peerj-14-20970-s005.zip › Figure 3/D/MCC_top10_with_neighbors_and_expanded.png]

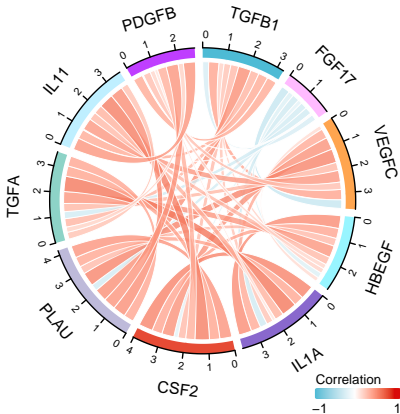

Supplement: Supplemental Information 5 [file peerj-14-20970-s005.zip › Figure 3/E/F2RL1-HUB-Chord diagram/output/F2RL1-HUB-Chord diagram.pdf]

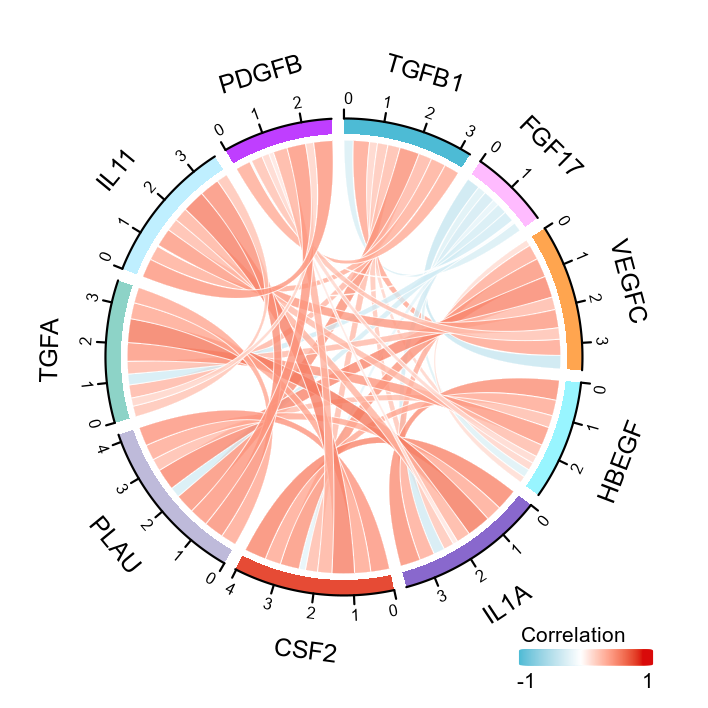

Supplement: Supplemental Information 5 [file peerj-14-20970-s005.zip › Figure 3/E/F2RL1-HUB-Chord diagram/output/F2RL1-HUB-Chord diagram.png]

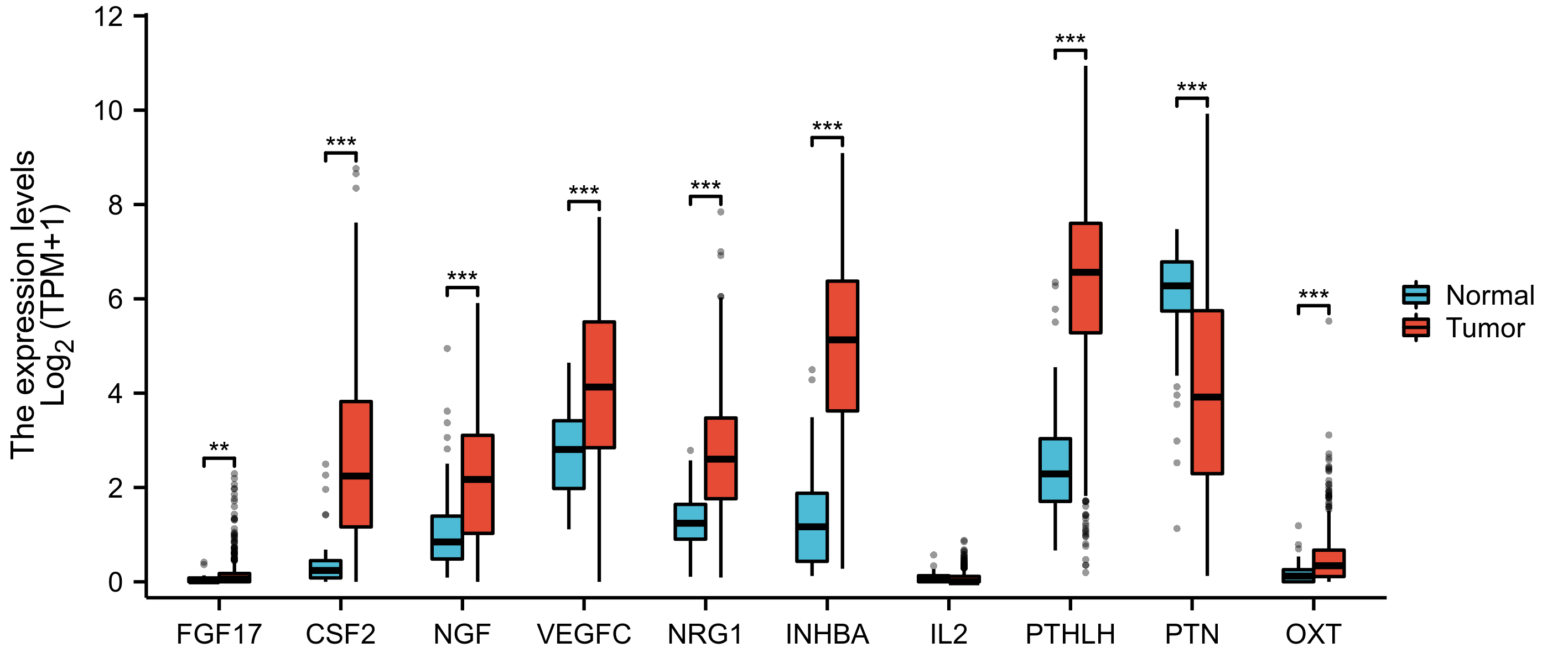

Supplement: Supplemental Information 5 [file peerj-14-20970-s005.zip › Figure 3/F/HUB Unpaired Sample.tiff]

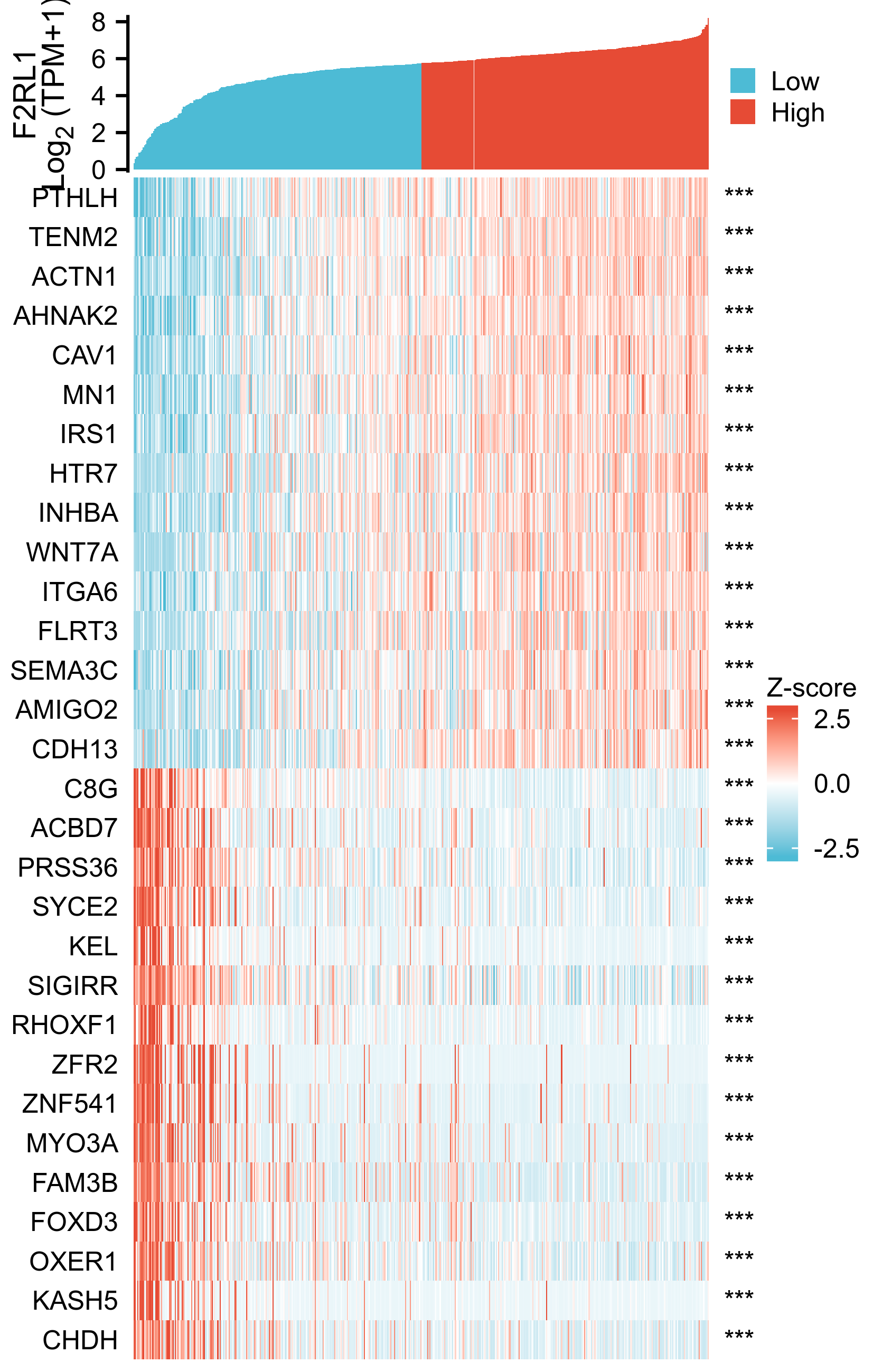

Supplement: Supplemental Information 5 [file peerj-14-20970-s005.zip › Figure 4/A/DEmRNA+F2RL1 Correlated Gene.tiff]

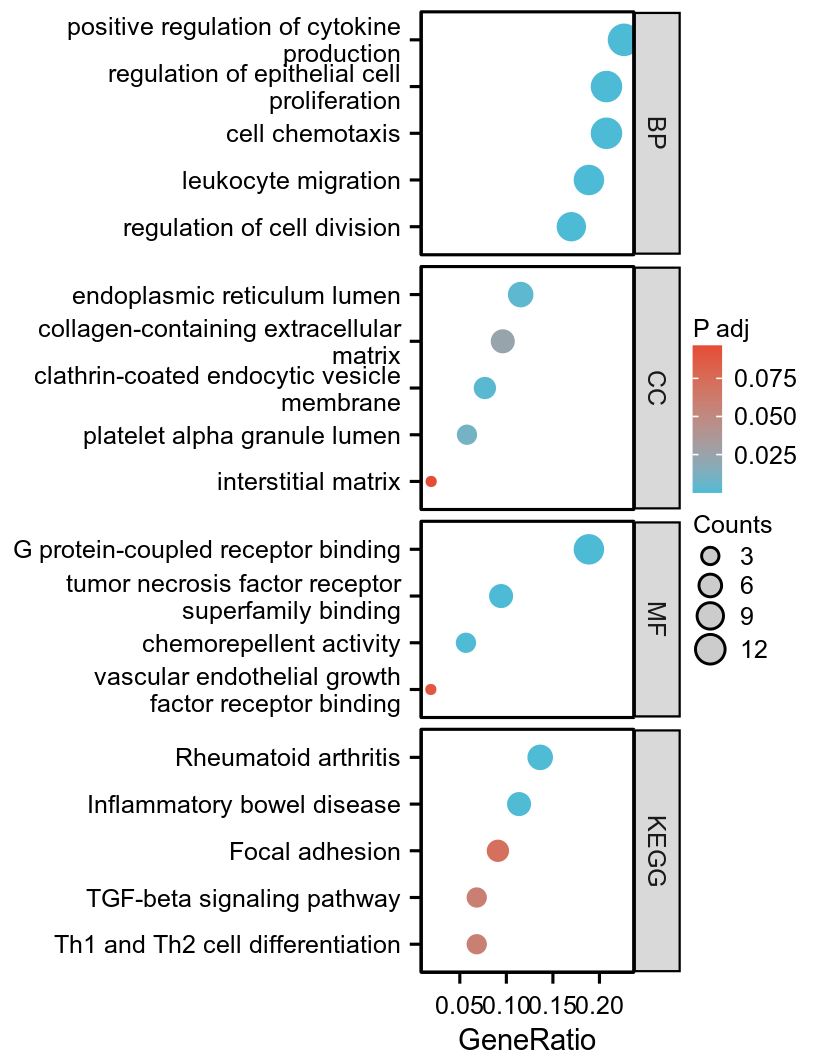

Supplement: Supplemental Information 5 [file peerj-14-20970-s005.zip › Figure 4/B/GOKEGG.png]

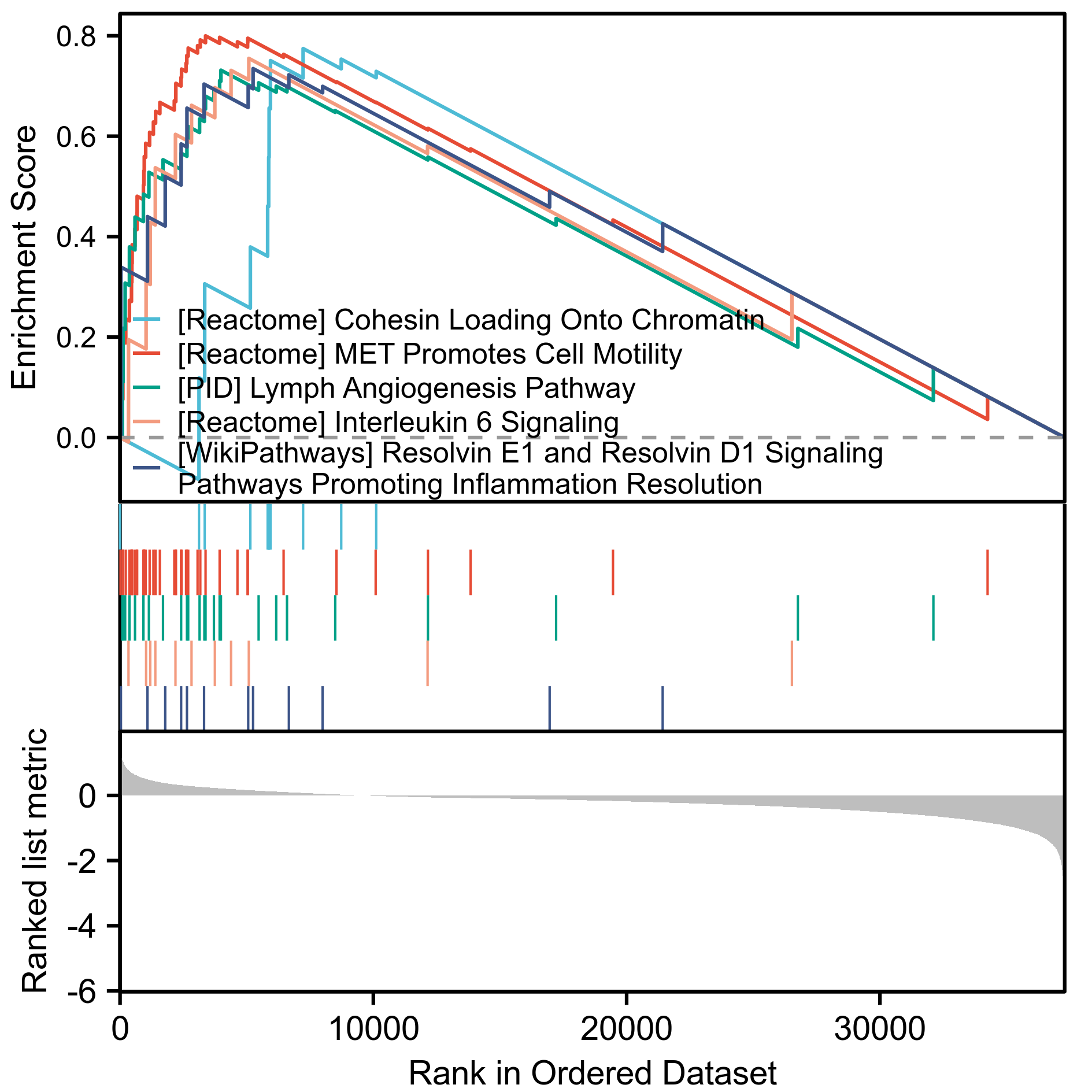

Supplement: Supplemental Information 5 [file peerj-14-20970-s005.zip › Figure 4/C,D/GSEA+5.tiff]

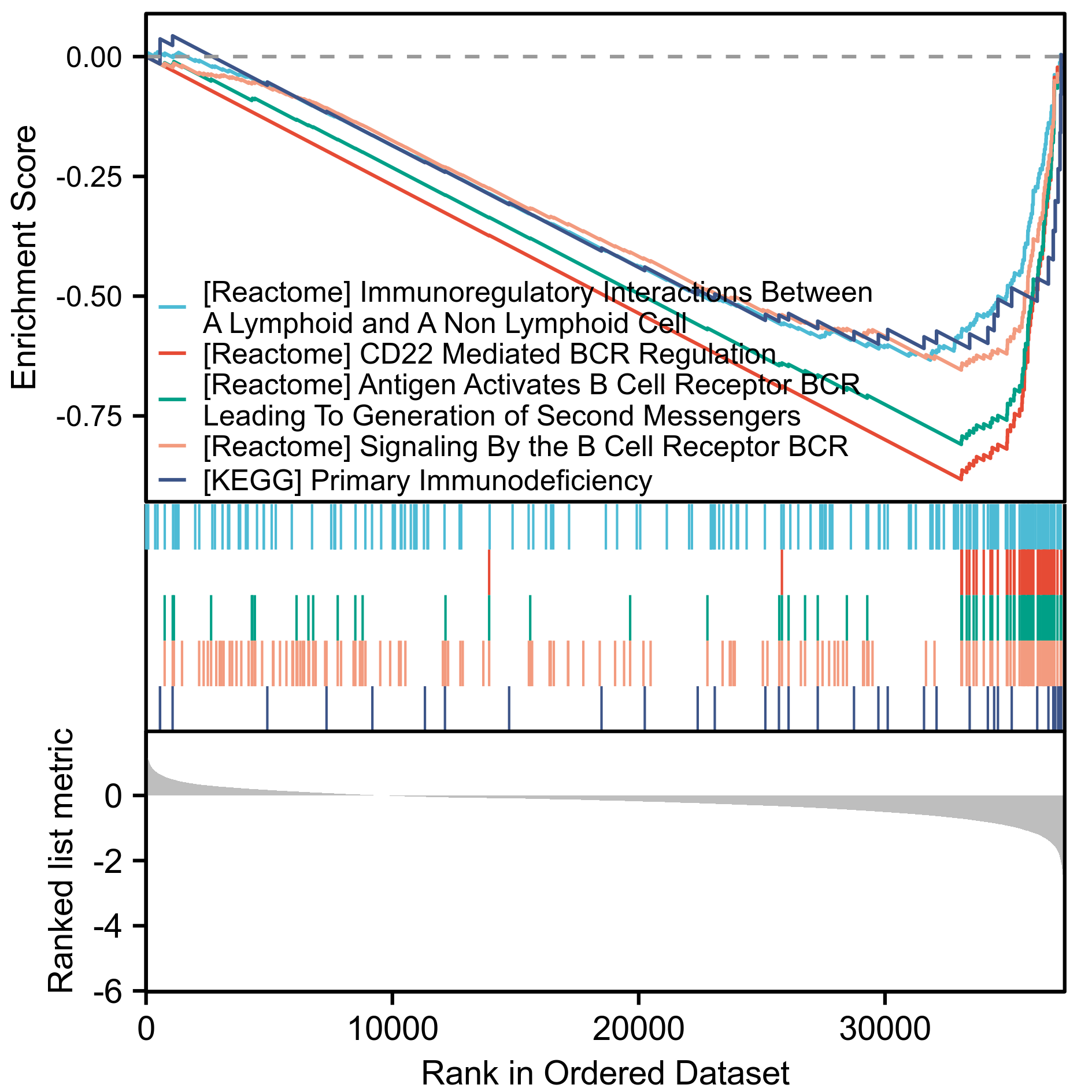

Supplement: Supplemental Information 5 [file peerj-14-20970-s005.zip › Figure 4/C,D/GSEA-5.tiff]

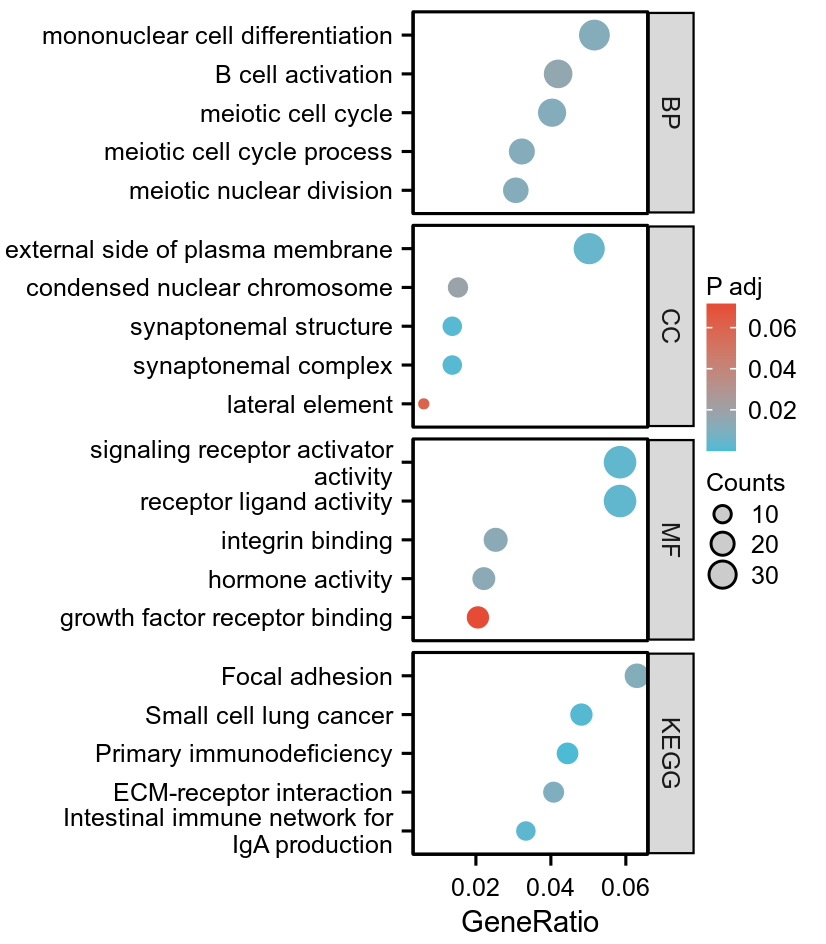

Supplement: Supplemental Information 5 [file peerj-14-20970-s005.zip › Figure 4/E/GOKEGG Bubble Chart.png]

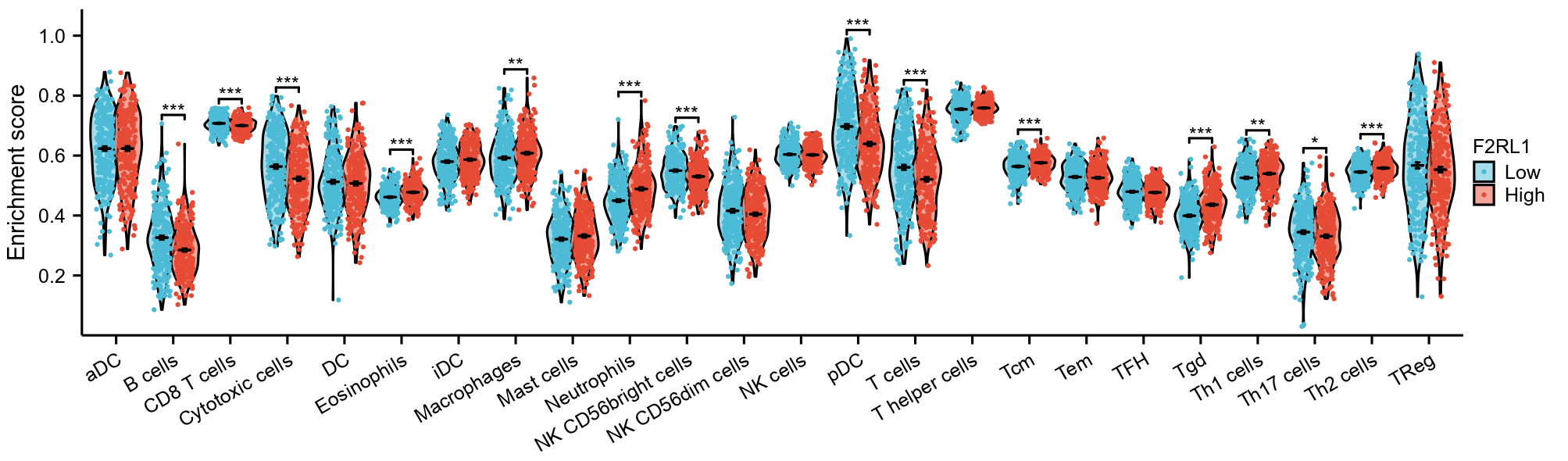

Supplement: Supplemental Information 5 [file peerj-14-20970-s005.zip › Figure 5/A/Immune Infiltration - Subgroup Comparison/output/Group comparison.png]

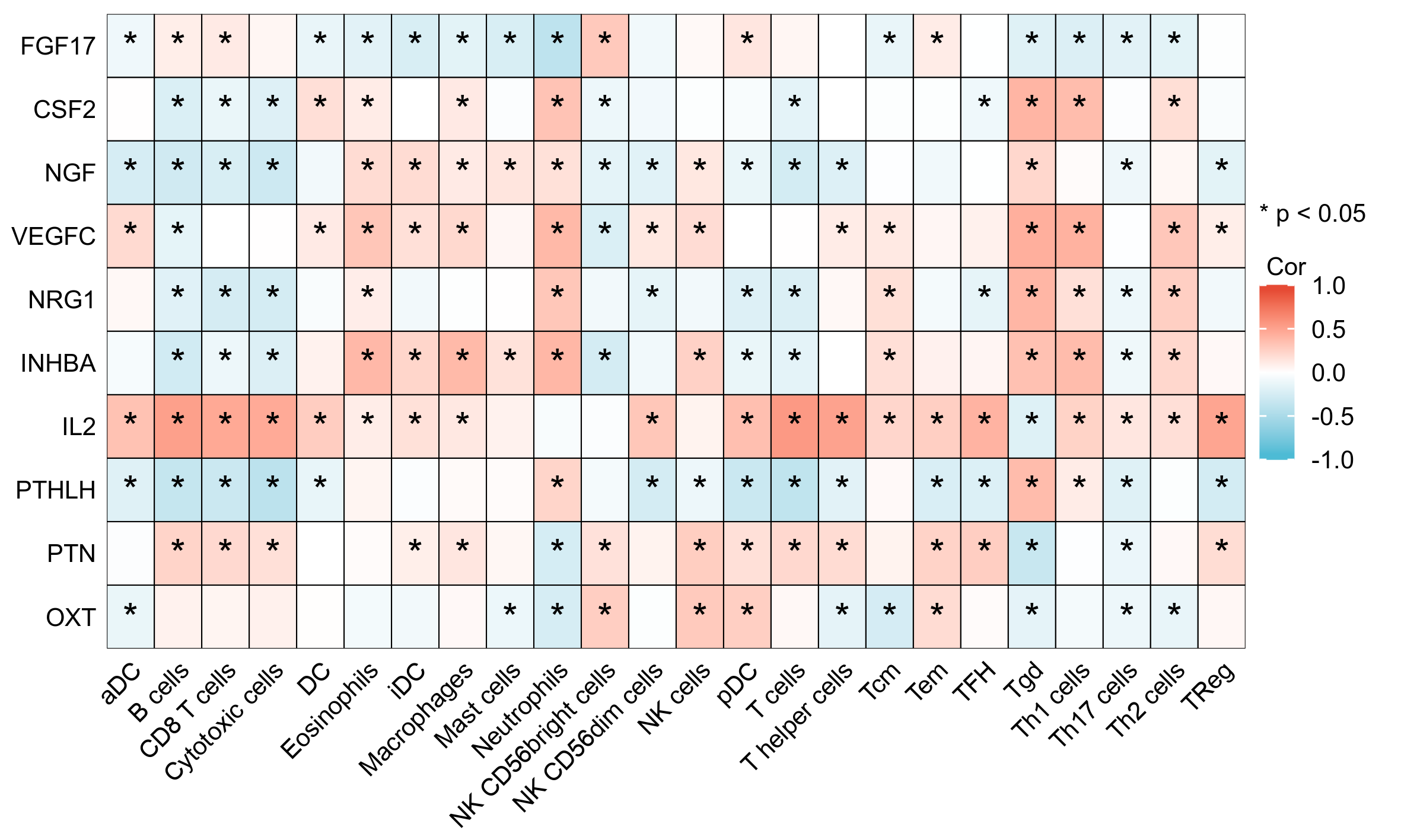

Supplement: Supplemental Information 5 [file peerj-14-20970-s005.zip › Figure 5/B/Immune cell and gene correlation.tiff]

## F2RL1

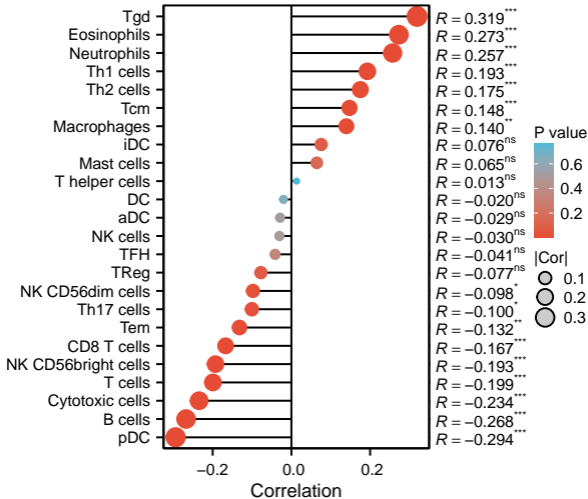

Supplement: Supplemental Information 5 [file peerj-14-20970-s005.zip › Figure 5/C/F2RL1-HNSE-Immune Infiltration - Lollipop Chart/output/pic.pdf]

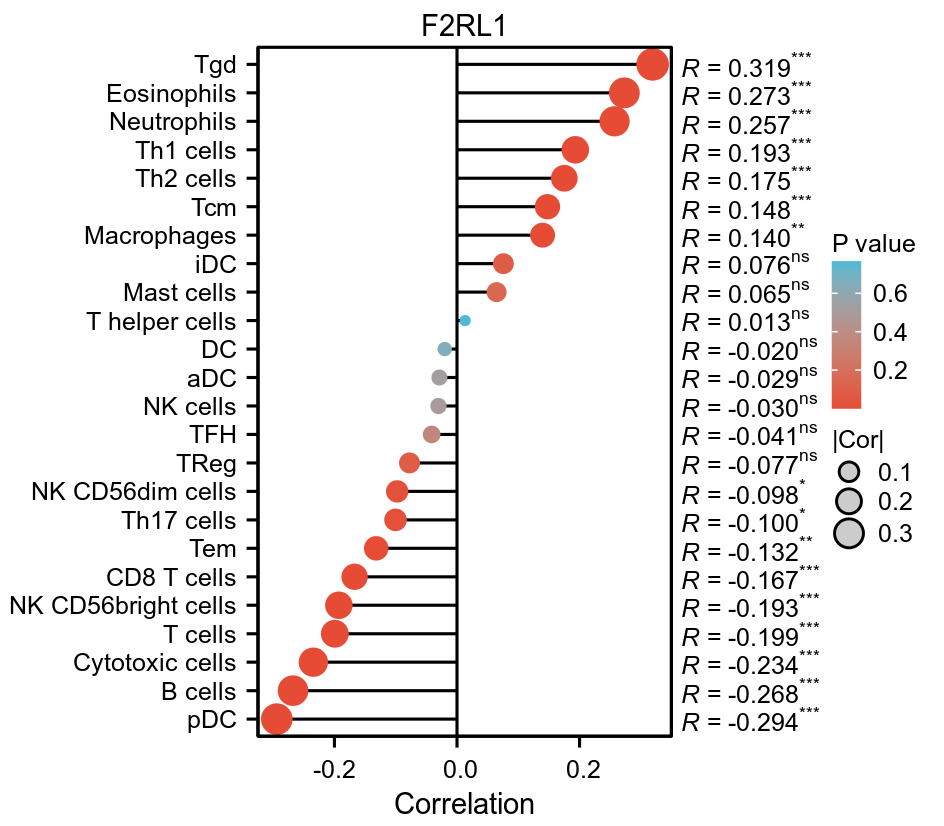

Supplement: Supplemental Information 5 [file peerj-14-20970-s005.zip › Figure 5/C/F2RL1-HNSE-Immune Infiltration - Lollipop Chart/output/pic.png]

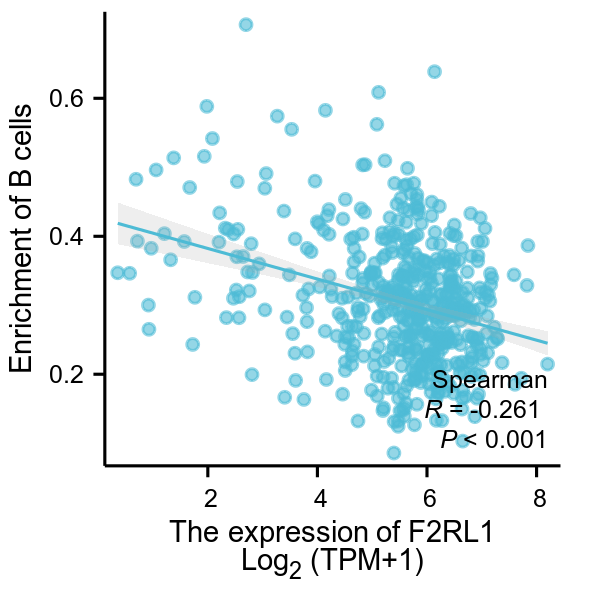

Supplement: Supplemental Information 5 [file peerj-14-20970-s005.zip › Figure 5/D-G/pic.png]

## F2RL1-HNSE-B-cells-enriched

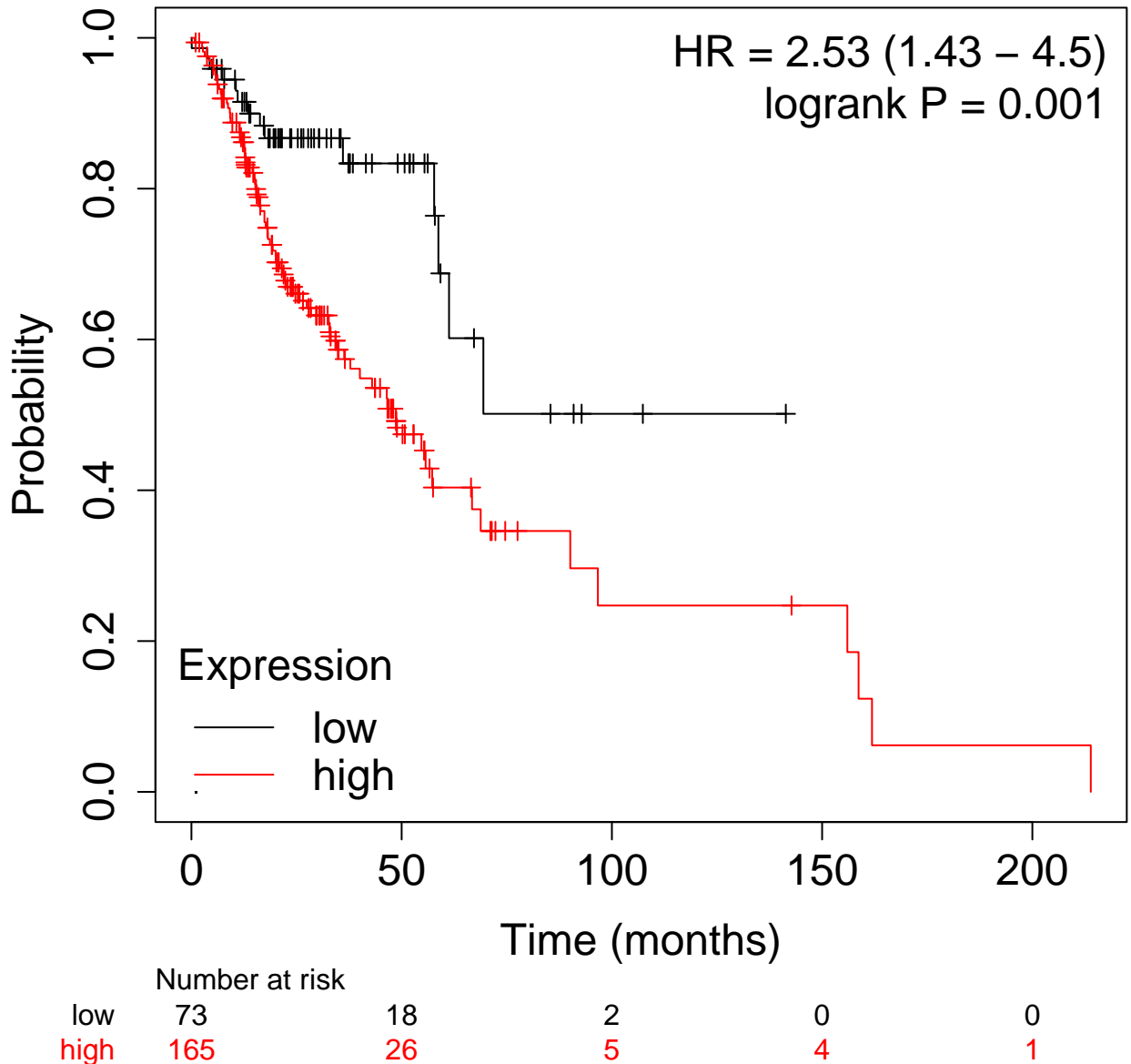

Supplement: Supplemental Information 5 [file peerj-14-20970-s005.zip › Figure 5/H-K/F2RL1-HNSE-B-cells-enriched.pdf]

# F2RL1-HNSE-Eosinophils-decreased

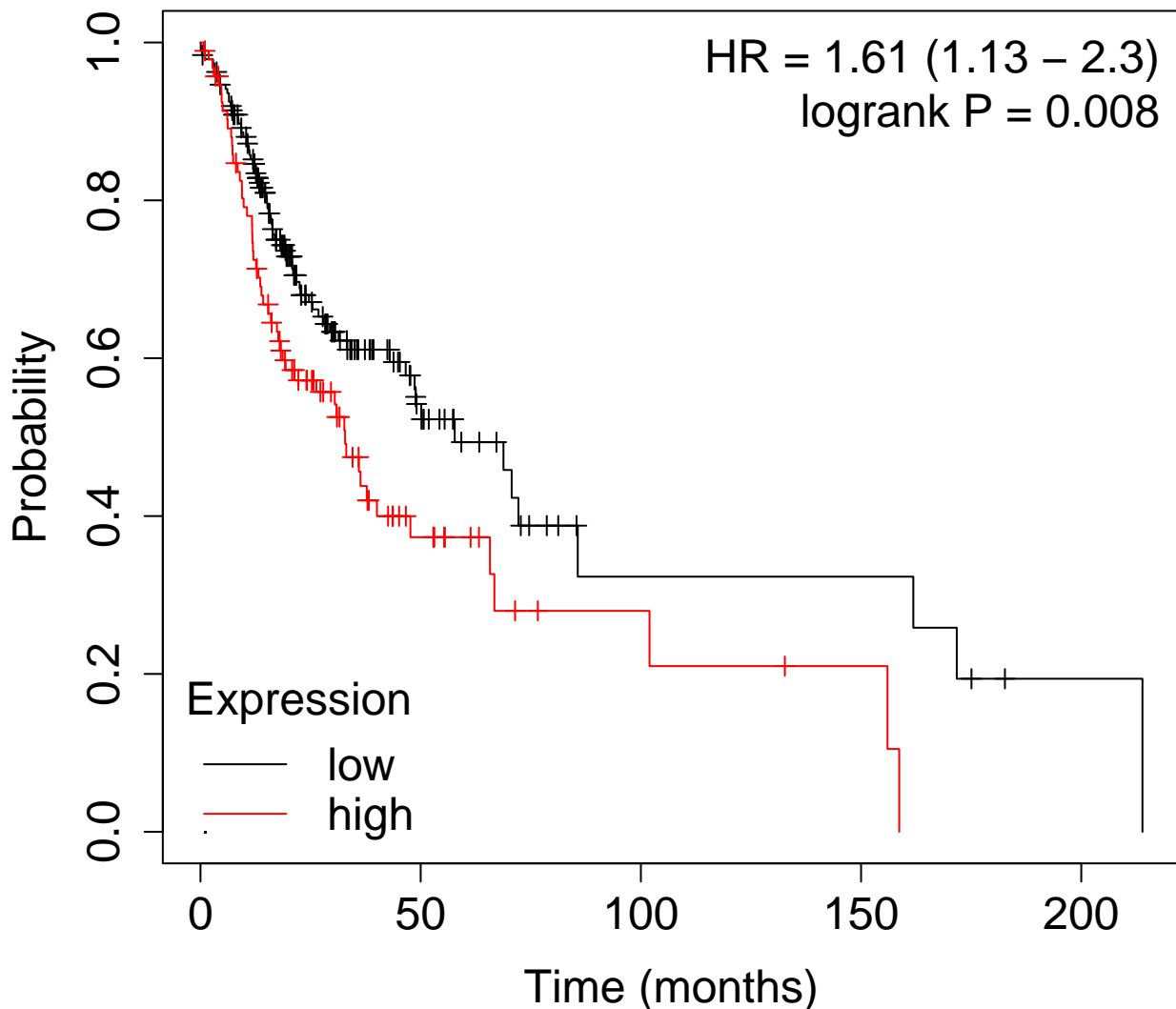

Number at risk

low  
high

188  
94

28  
14

5  
4

5  
2

1  
0

Supplement: Supplemental Information 5 [file peerj-14-20970-s005.zip › Figure 5/H-K/F2RL1-HNSE-Eosinophils-decreased.pdf]

## F2RL1-HNSE-Eosinophils-enriched

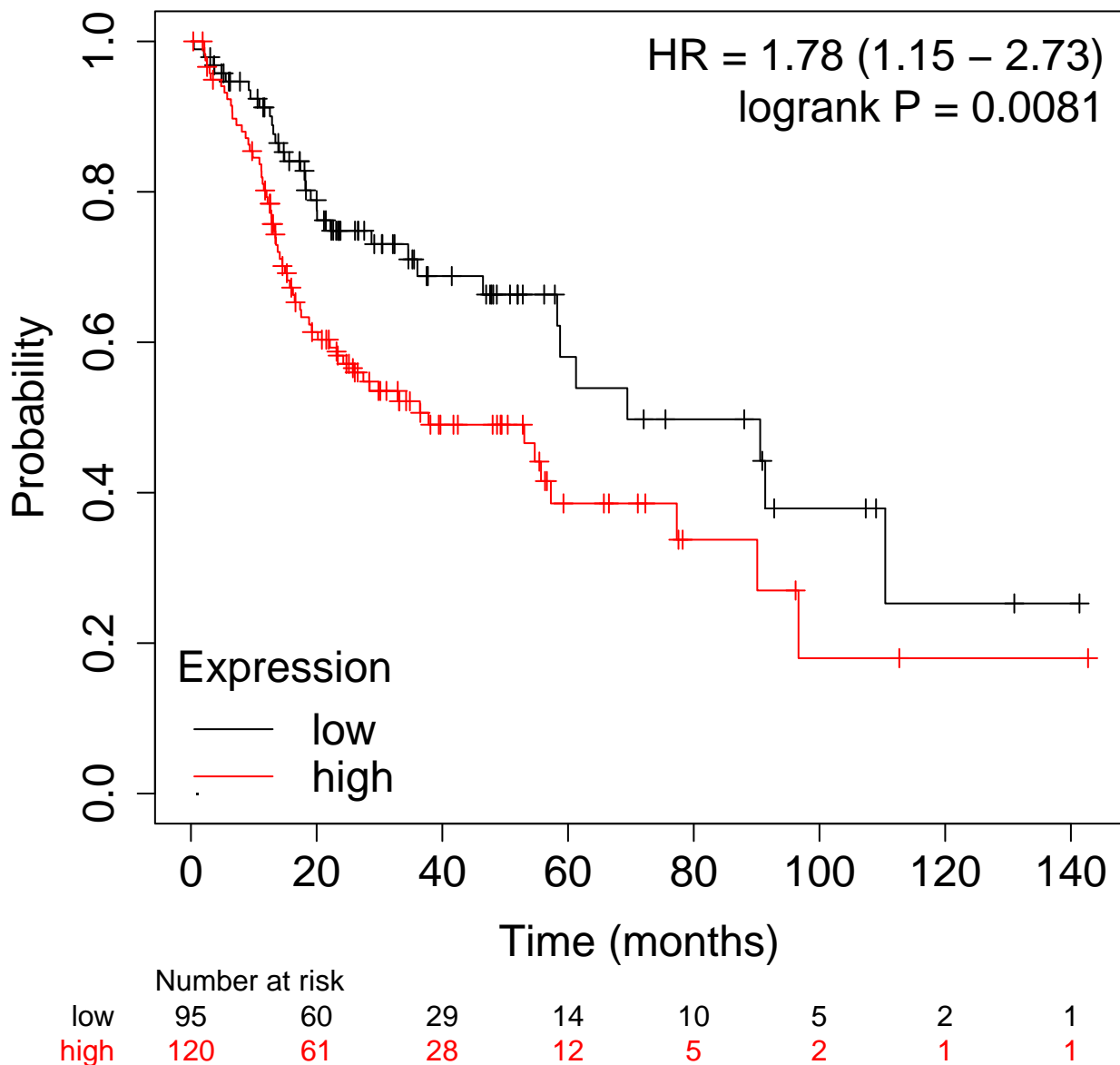

Supplement: Supplemental Information 5 [file peerj-14-20970-s005.zip › Figure 5/H-K/F2RL1-HNSE-Eosinophils-enriched.pdf]

# F2RL1-HNSE-Natural killer T-cells-decreased

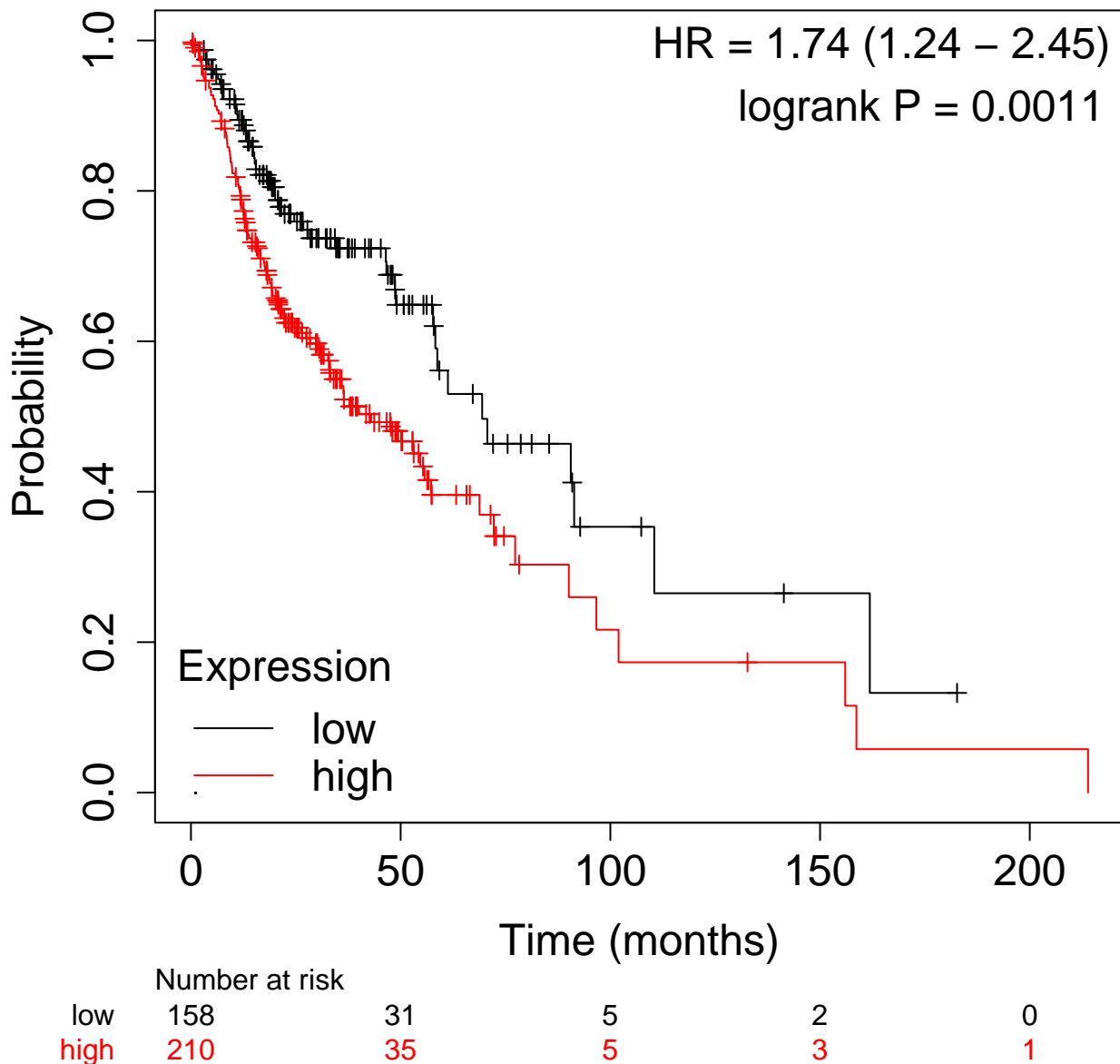

Supplement: Supplemental Information 5 [file peerj-14-20970-s005.zip › Figure 5/H-K/F2RL1-HNSE-Natural killer T-cells-decreased.pdf]

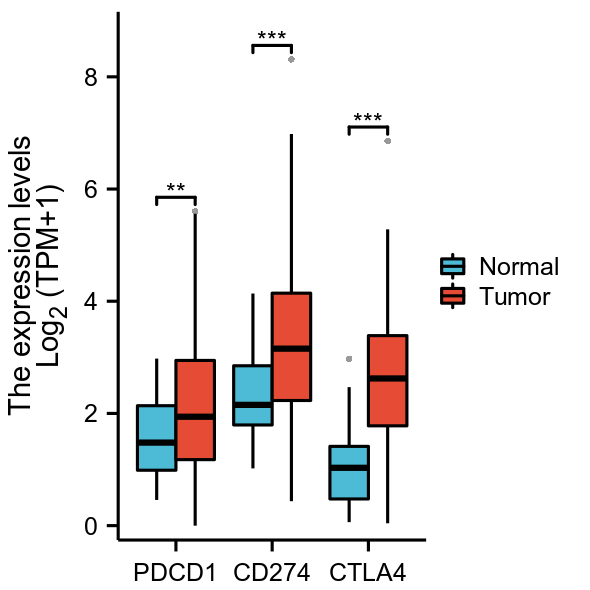

Supplement: Supplemental Information 5 [file peerj-14-20970-s005.zip › Figure 5/L/output/Unpaired sample.png]

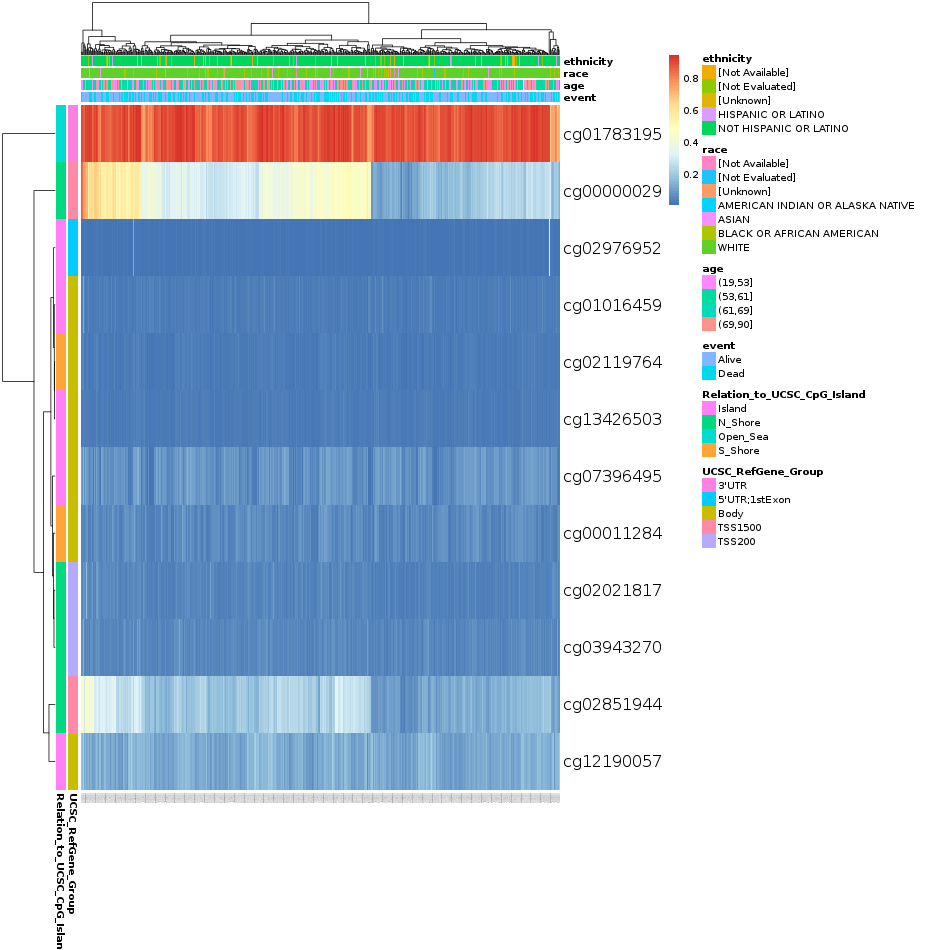

Supplement: Supplemental Information 5 [file peerj-14-20970-s005.zip › Figure 6/A/heatmap.png]

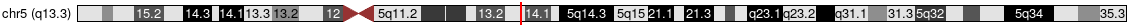

Supplement: Supplemental Information 5 [file peerj-14-20970-s005.zip › Figure 6/B/E.png]

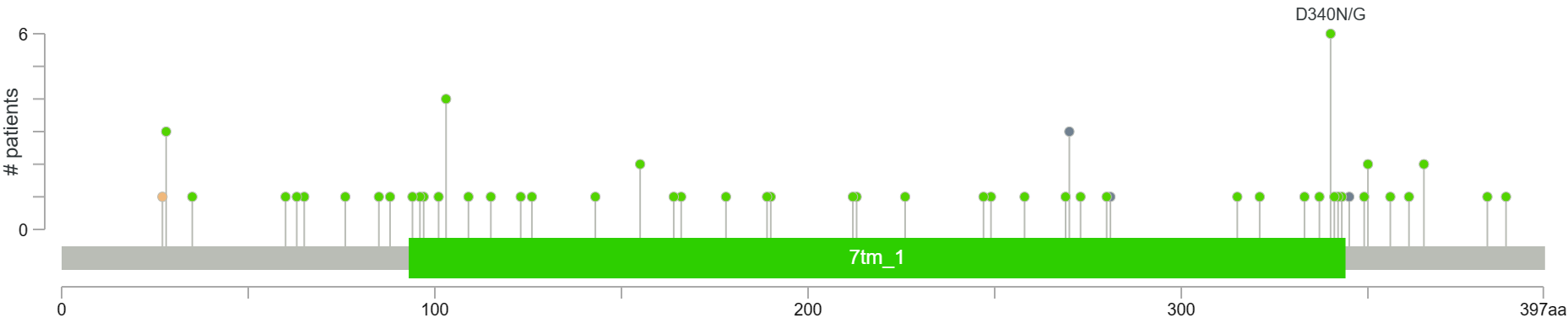

Supplement: Supplemental Information 5 [file peerj-14-20970-s005.zip › Figure 6/C/F2RL1-Mutation.png]

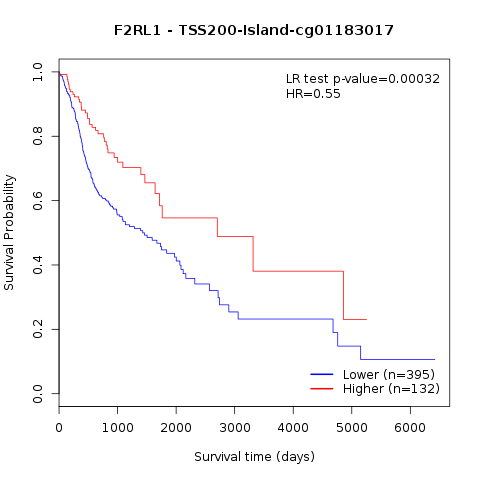

Supplement: Supplemental Information 5 [file peerj-14-20970-s005.zip › Figure 6/D-L/others/survplot (3).png]

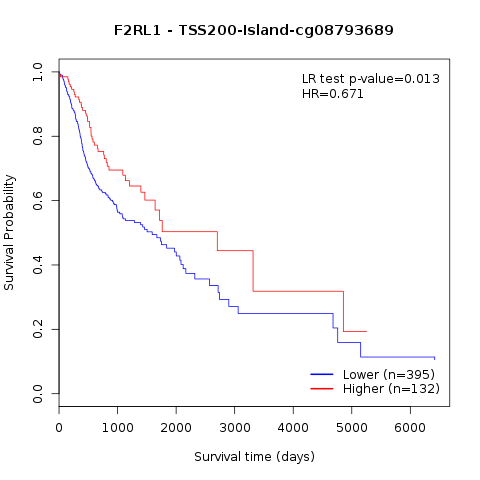

Supplement: Supplemental Information 5 [file peerj-14-20970-s005.zip › Figure 6/D-L/others/survplot (4).png]

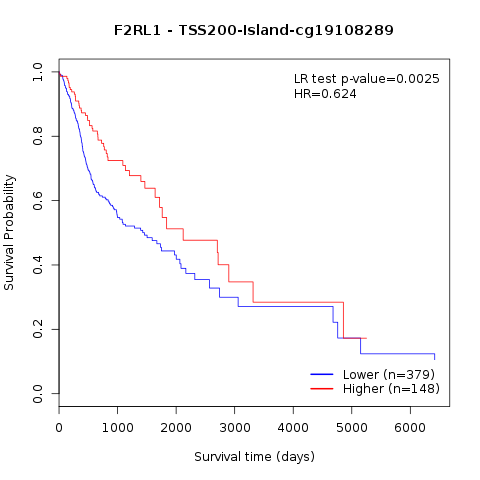

Supplement: Supplemental Information 5 [file peerj-14-20970-s005.zip › Figure 6/D-L/others/survplot (6).png]

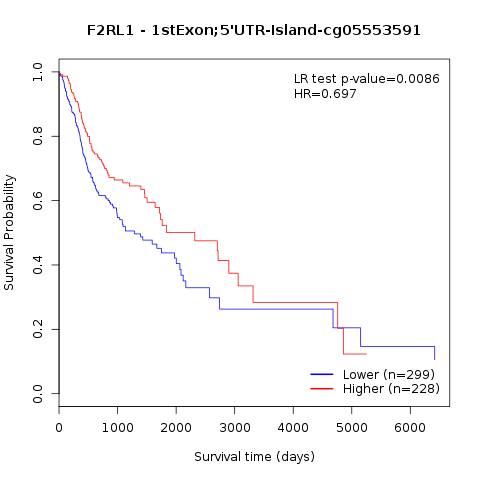

Supplement: Supplemental Information 5 [file peerj-14-20970-s005.zip › Figure 6/D-L/others/survplot (7).png]

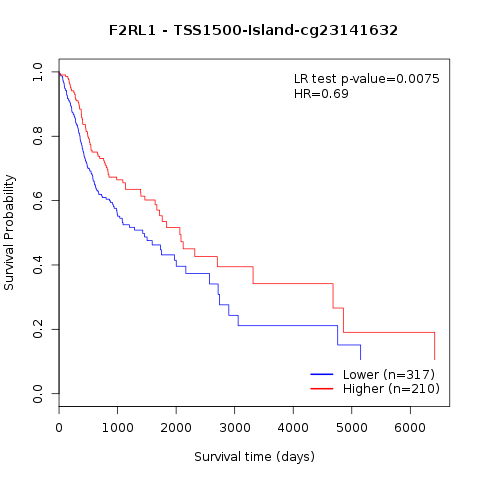

Supplement: Supplemental Information 5 [file peerj-14-20970-s005.zip › Figure 6/D-L/others/survplot (8).png]

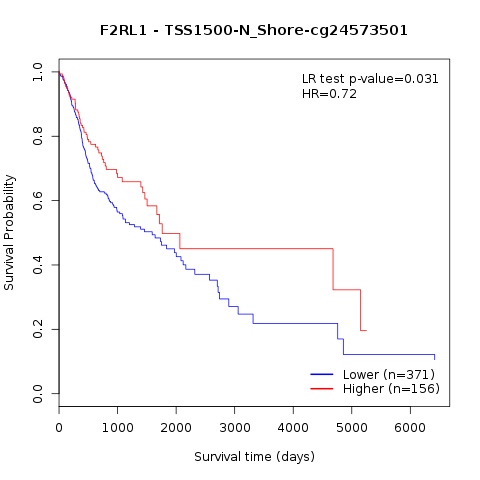

Supplement: Supplemental Information 5 [file peerj-14-20970-s005.zip › Figure 6/D-L/survplot (1).png]

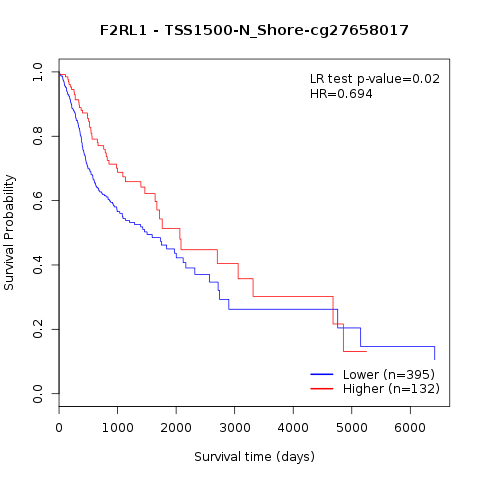

Supplement: Supplemental Information 5 [file peerj-14-20970-s005.zip › Figure 6/D-L/survplot (2).png]

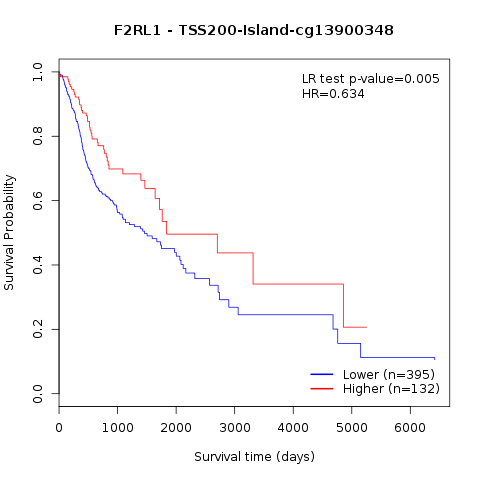

Supplement: Supplemental Information 5 [file peerj-14-20970-s005.zip › Figure 6/D-L/survplot (5).png]

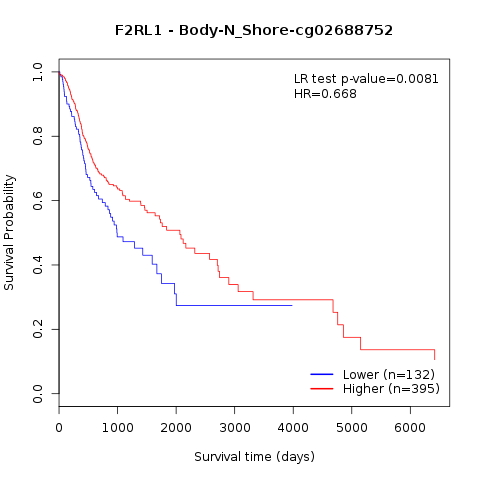

Supplement: Supplemental Information 5 [file peerj-14-20970-s005.zip › Figure 6/D-L/survplot.png]

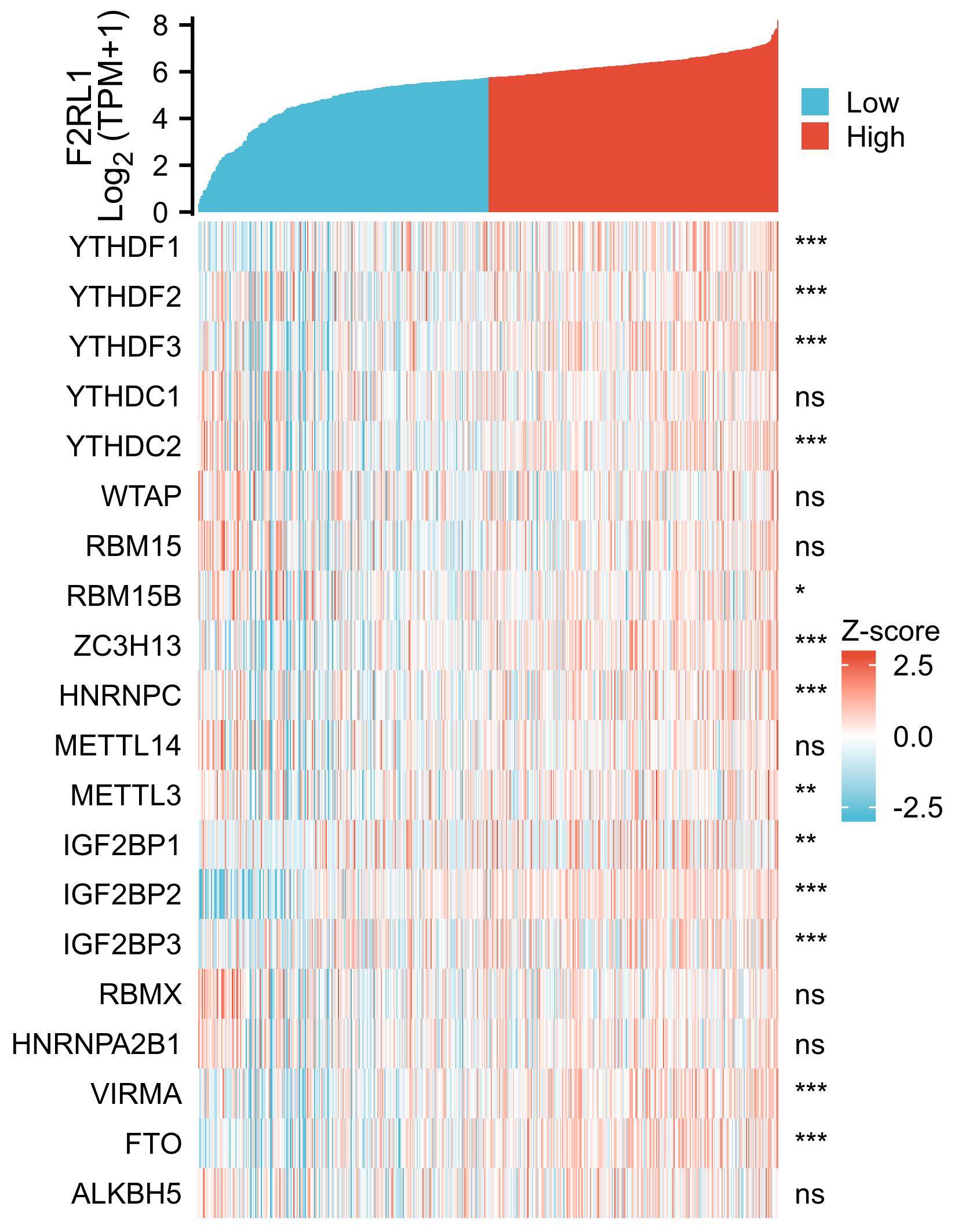

Supplement: Supplemental Information 5 [file peerj-14-20970-s005.zip › Figure 7/A/m6a.tiff]

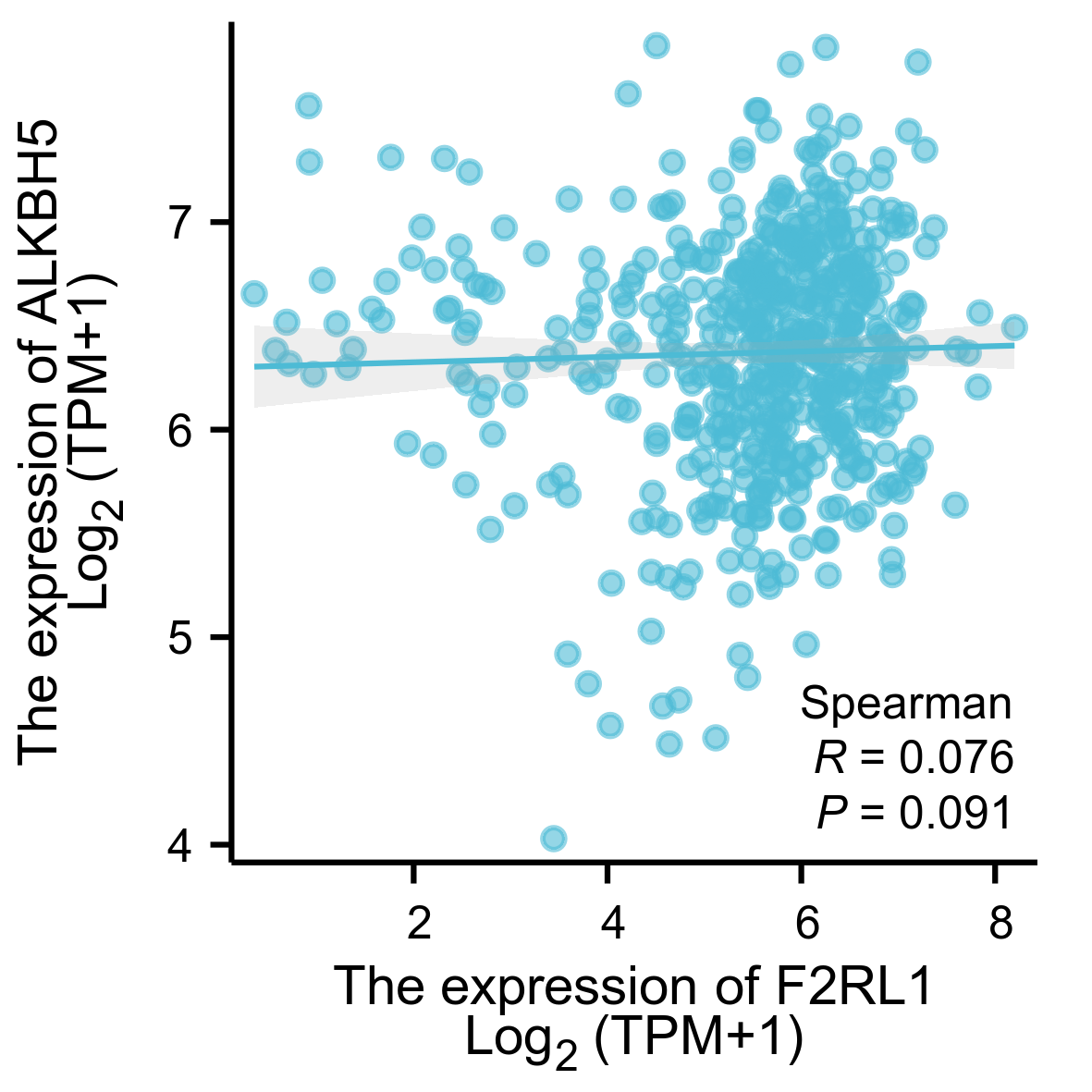

Supplement: Supplemental Information 5 [file peerj-14-20970-s005.zip › Figure 7/B-M/no-Significance/ALKBH5.tiff]

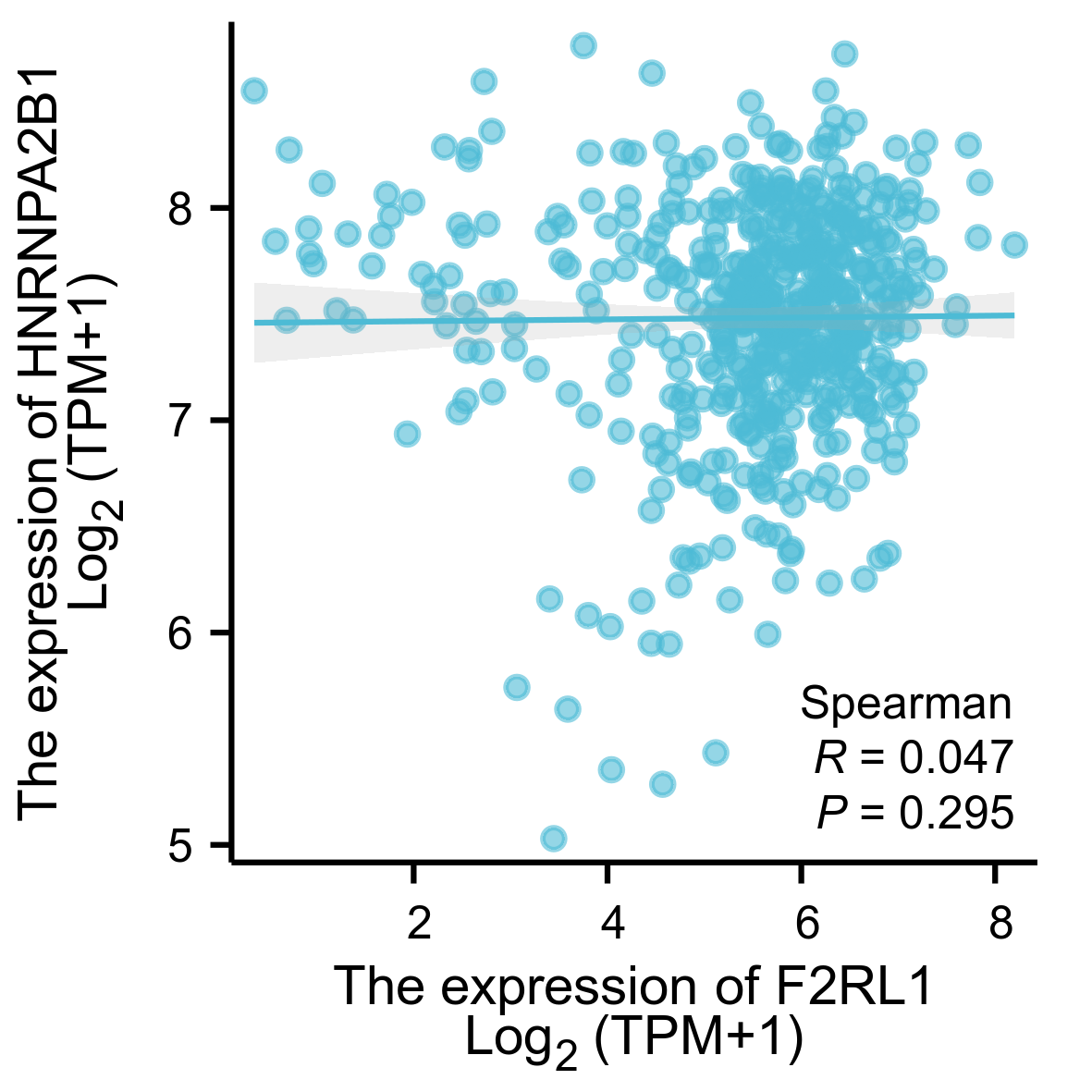

Supplement: Supplemental Information 5 [file peerj-14-20970-s005.zip › Figure 7/B-M/no-Significance/HNRNPA2B1.tiff]

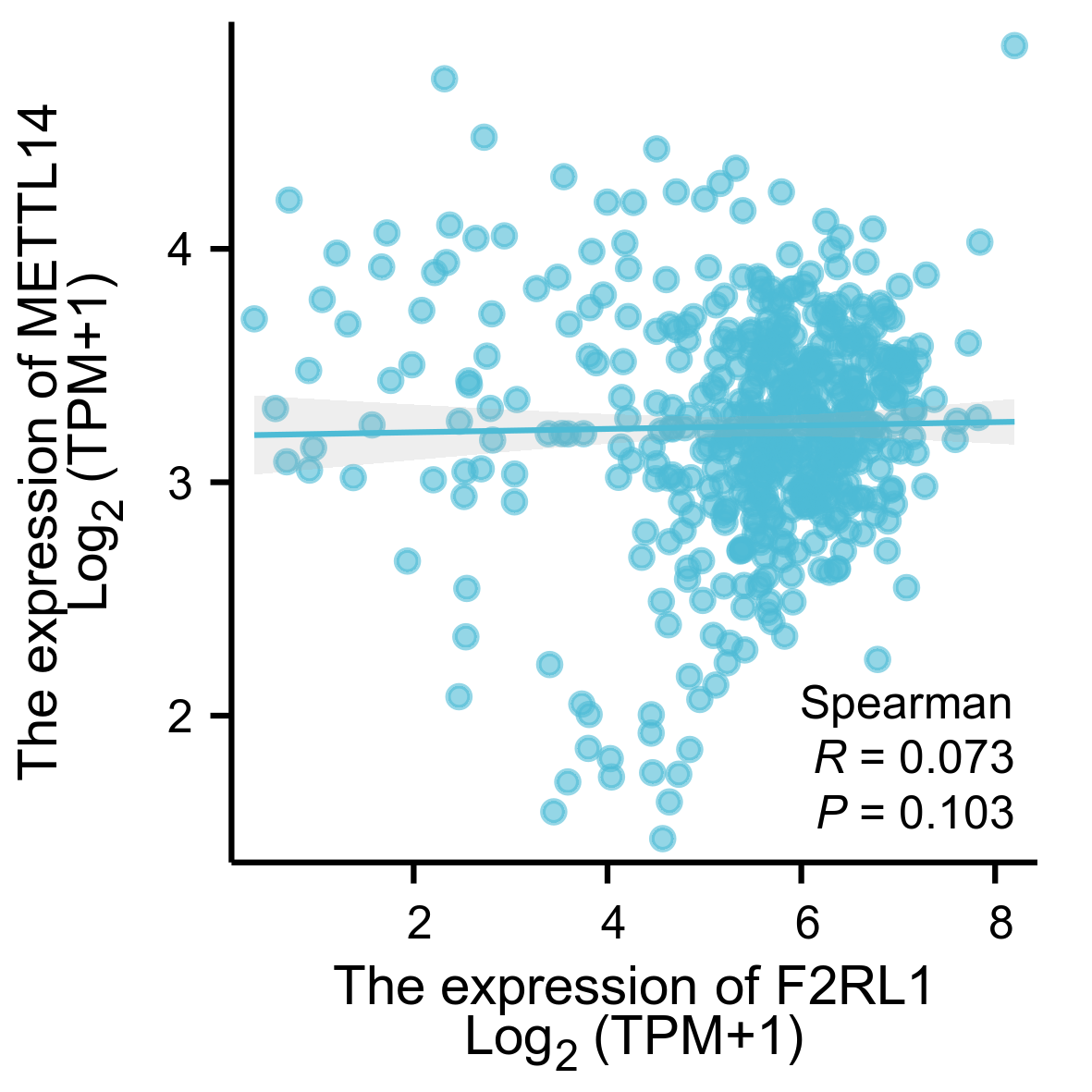

Supplement: Supplemental Information 5 [file peerj-14-20970-s005.zip › Figure 7/B-M/no-Significance/METTL14.tiff]

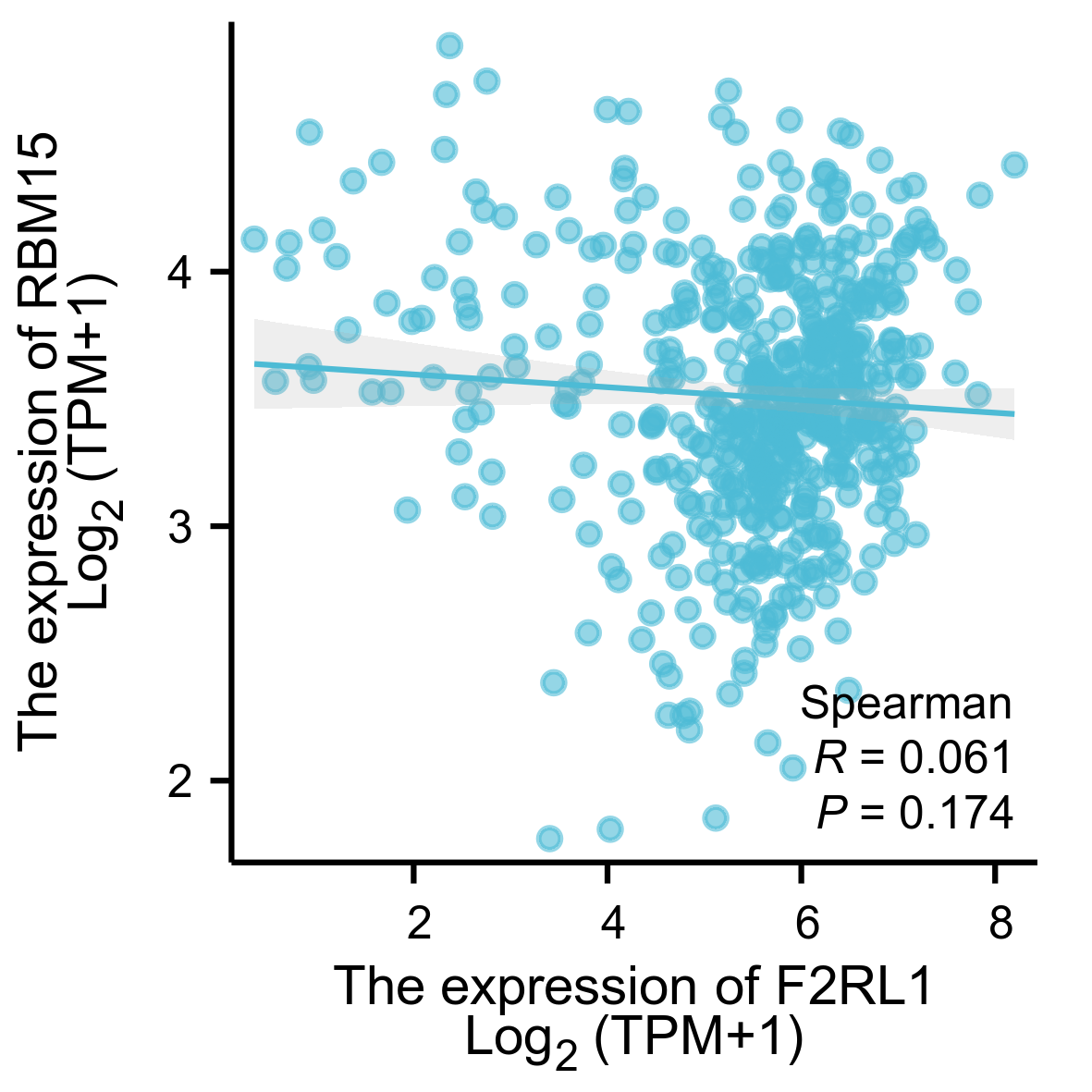

Supplement: Supplemental Information 5 [file peerj-14-20970-s005.zip › Figure 7/B-M/no-Significance/RBM15.tiff]

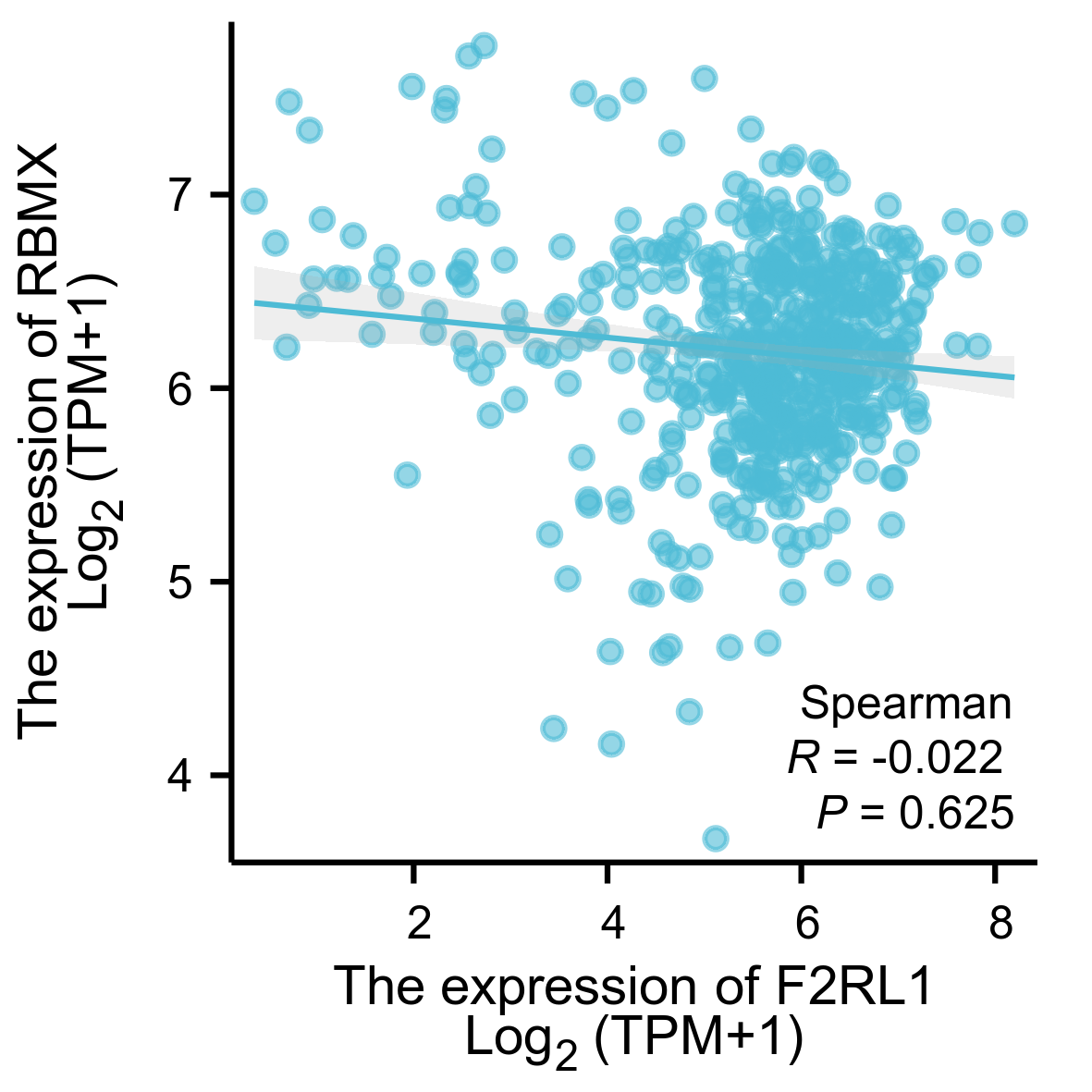

Supplement: Supplemental Information 5 [file peerj-14-20970-s005.zip › Figure 7/B-M/no-Significance/RBMX.tiff]

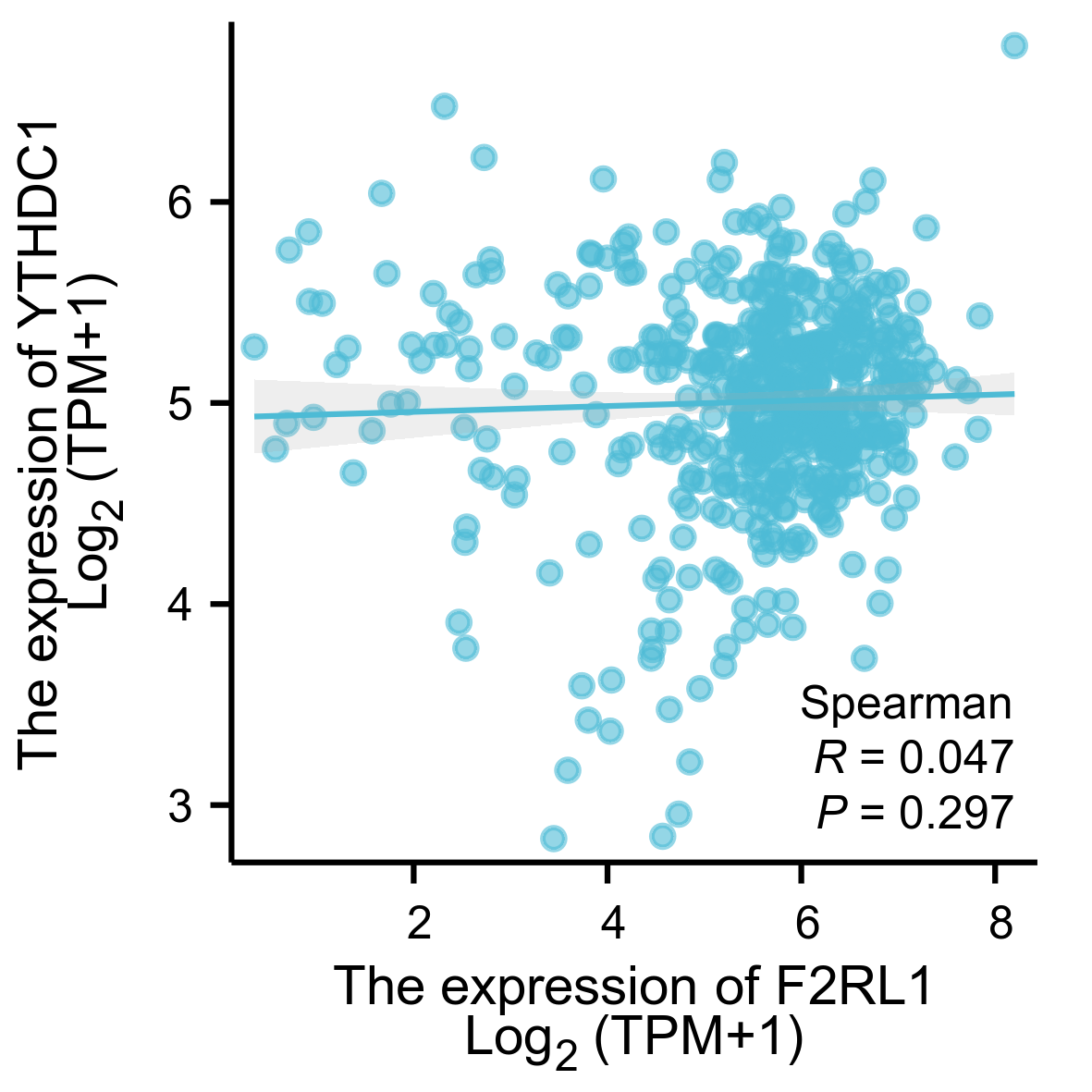

Supplement: Supplemental Information 5 [file peerj-14-20970-s005.zip › Figure 7/B-M/no-Significance/YTHDC1.tiff]

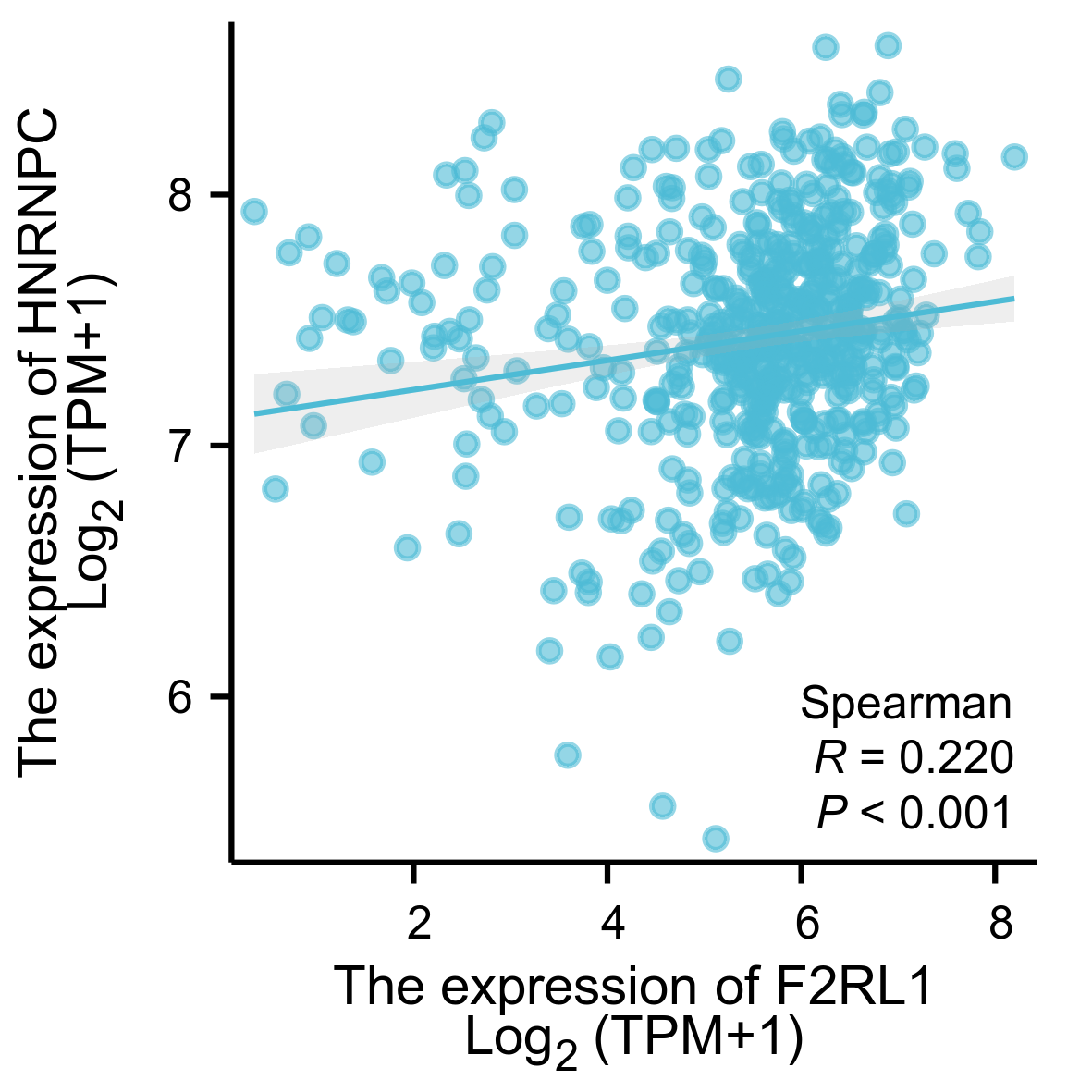

Supplement: Supplemental Information 5 [file peerj-14-20970-s005.zip › Figure 7/B-M/Significance/HNRNPC.tiff]

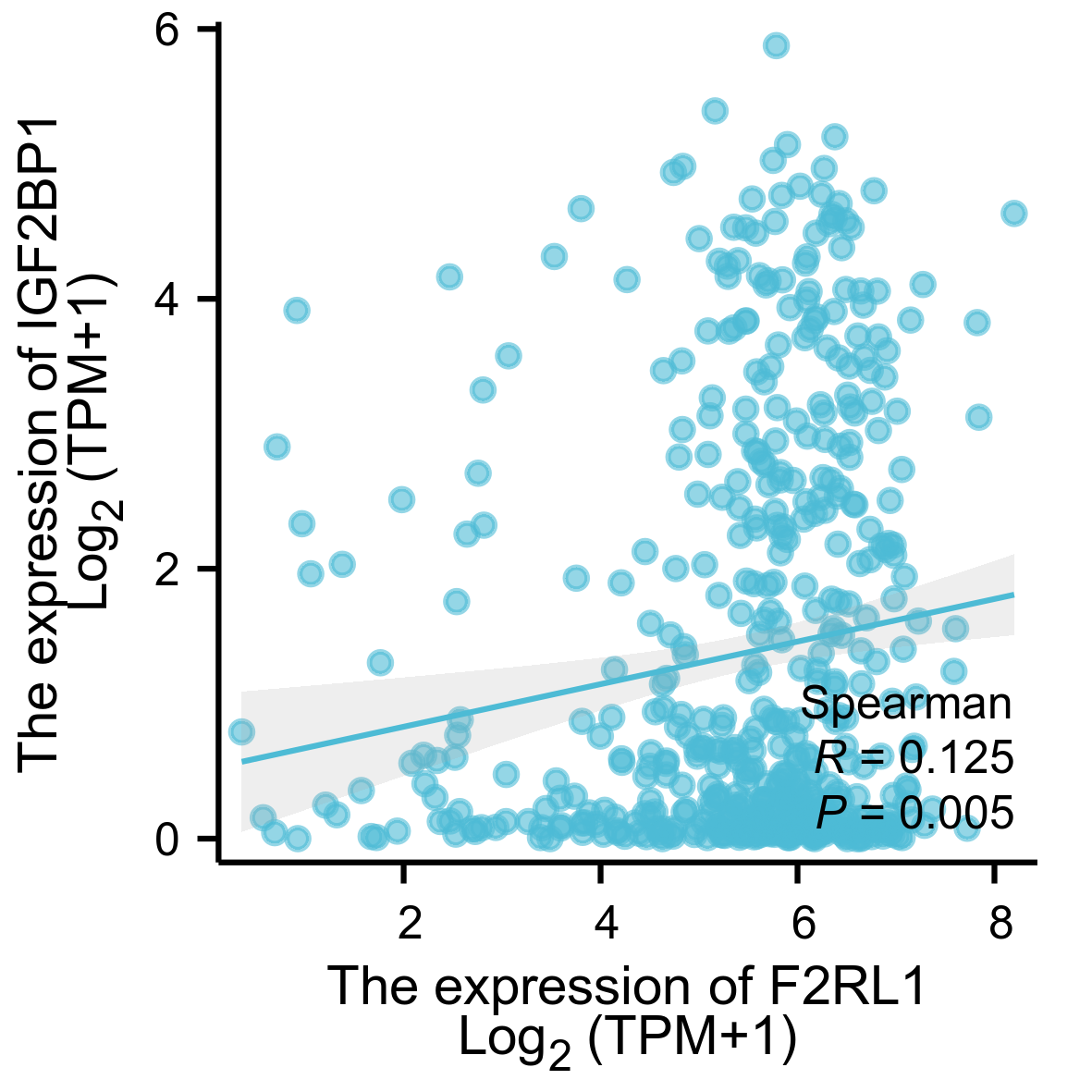

Supplement: Supplemental Information 5 [file peerj-14-20970-s005.zip › Figure 7/B-M/Significance/IGF2BP1.tiff]

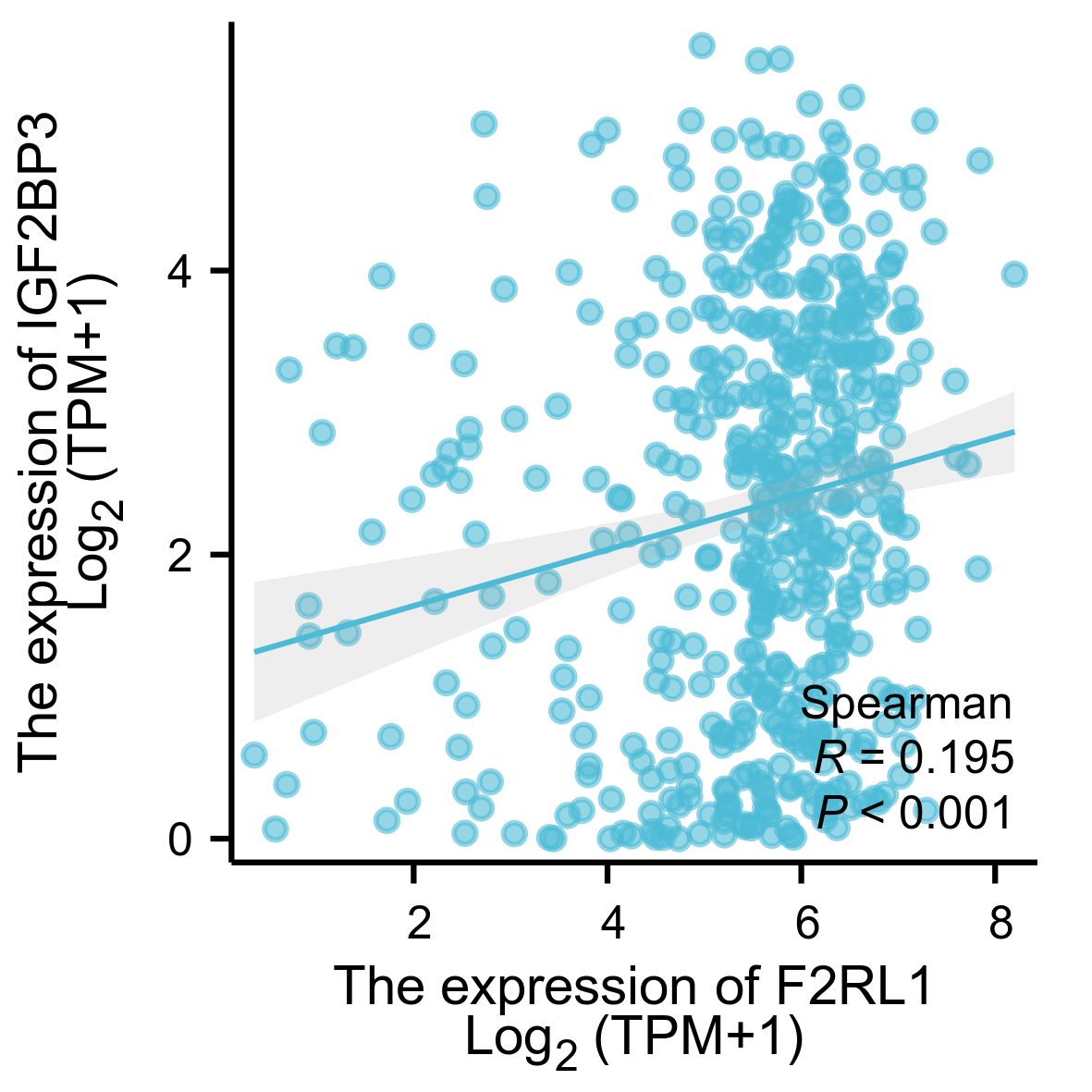

Supplement: Supplemental Information 5 [file peerj-14-20970-s005.zip › Figure 7/B-M/Significance/IGF2BP3.tiff]

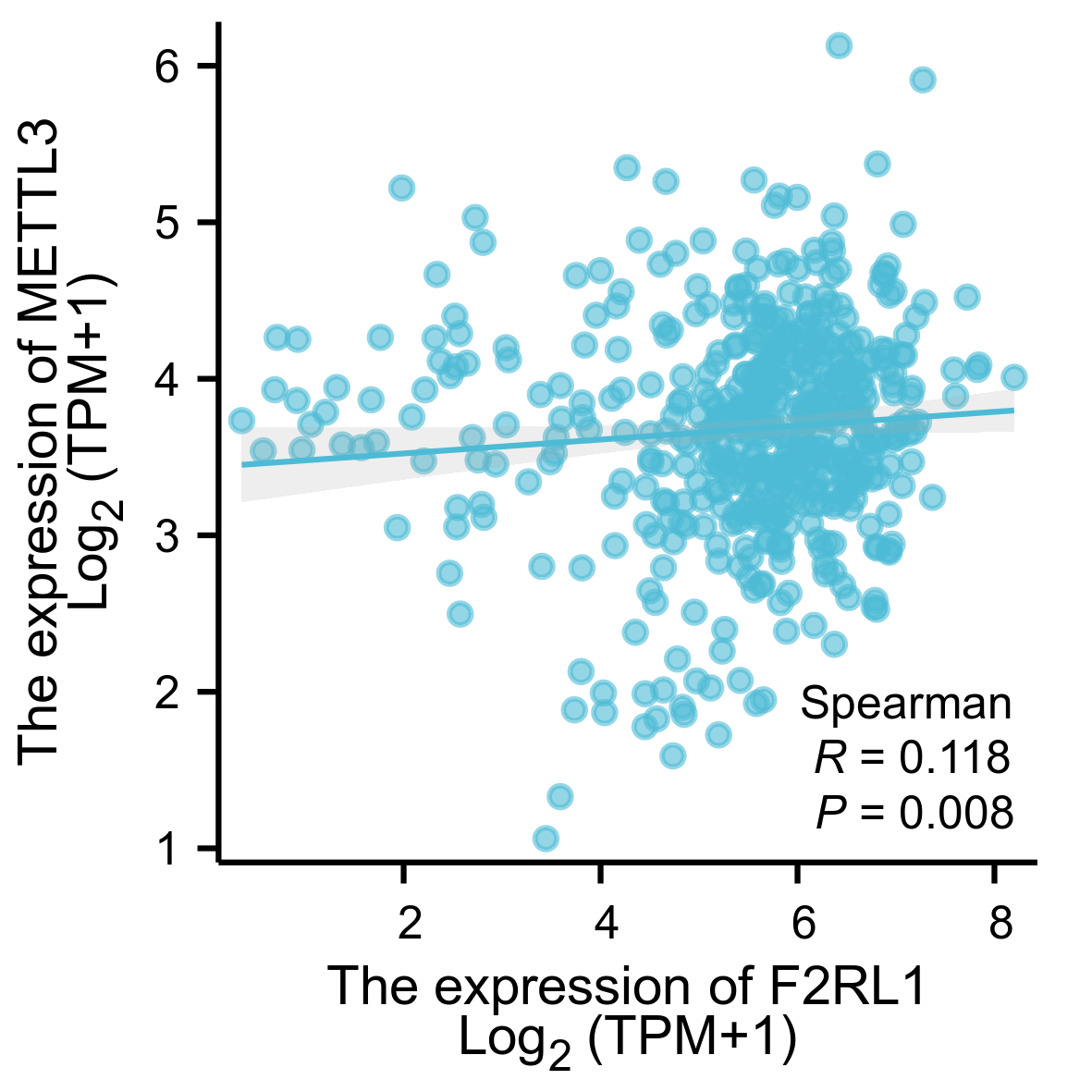

Supplement: Supplemental Information 5 [file peerj-14-20970-s005.zip › Figure 7/B-M/Significance/METTL3.tiff]

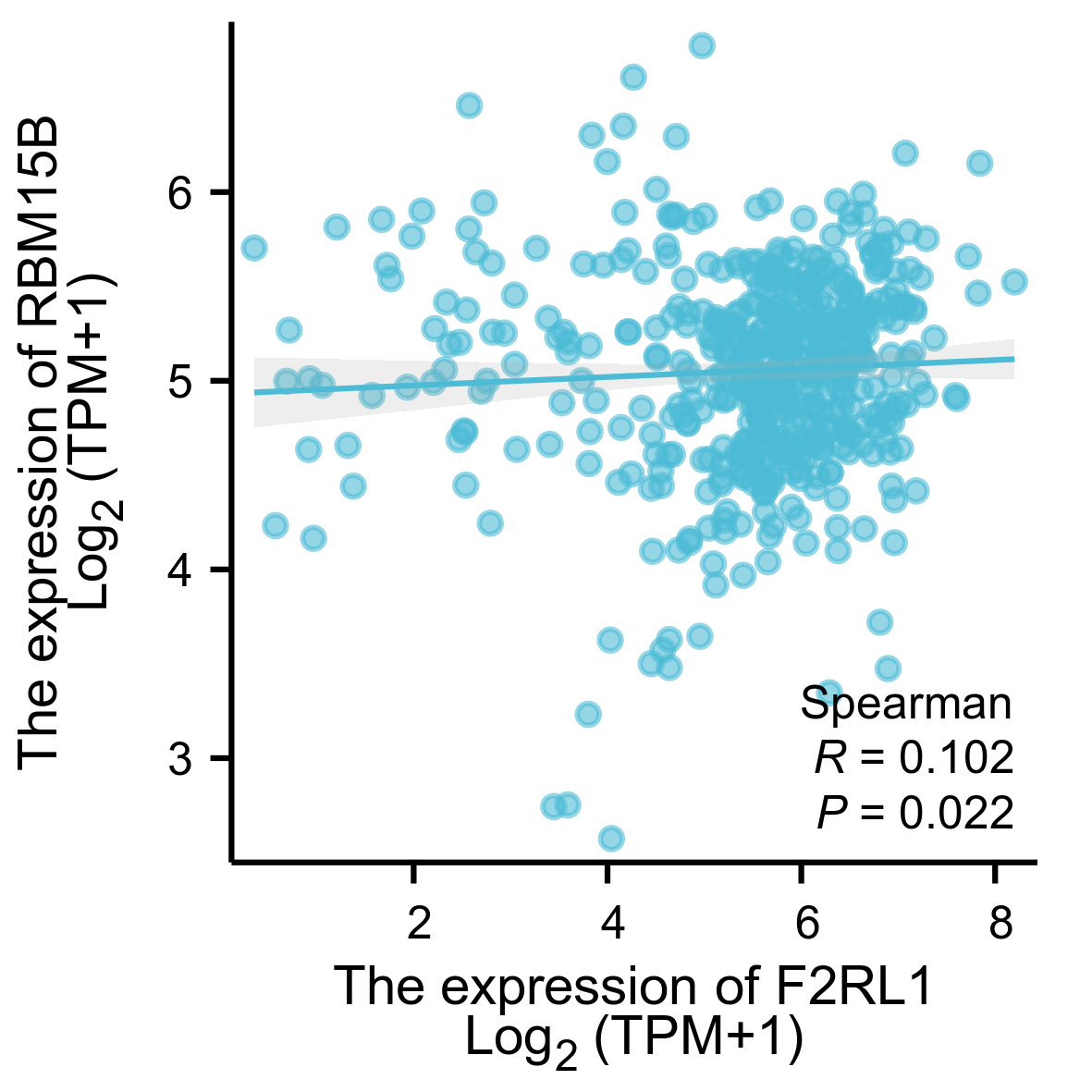

Supplement: Supplemental Information 5 [file peerj-14-20970-s005.zip › Figure 7/B-M/Significance/RBM15B.tiff]

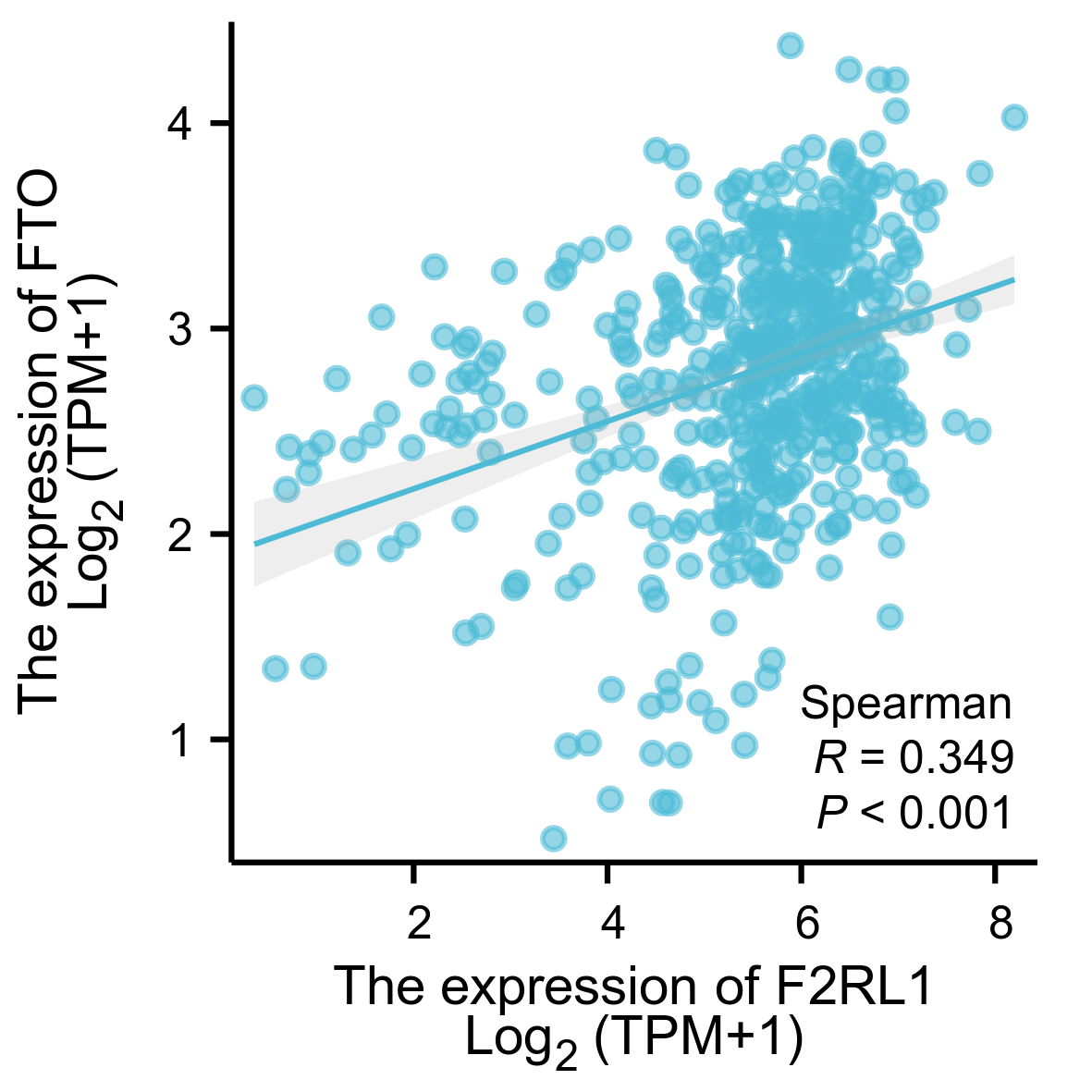

Supplement: Supplemental Information 5 [file peerj-14-20970-s005.zip › Figure 7/B-M/Significance/R》0.3/FTO.tiff]

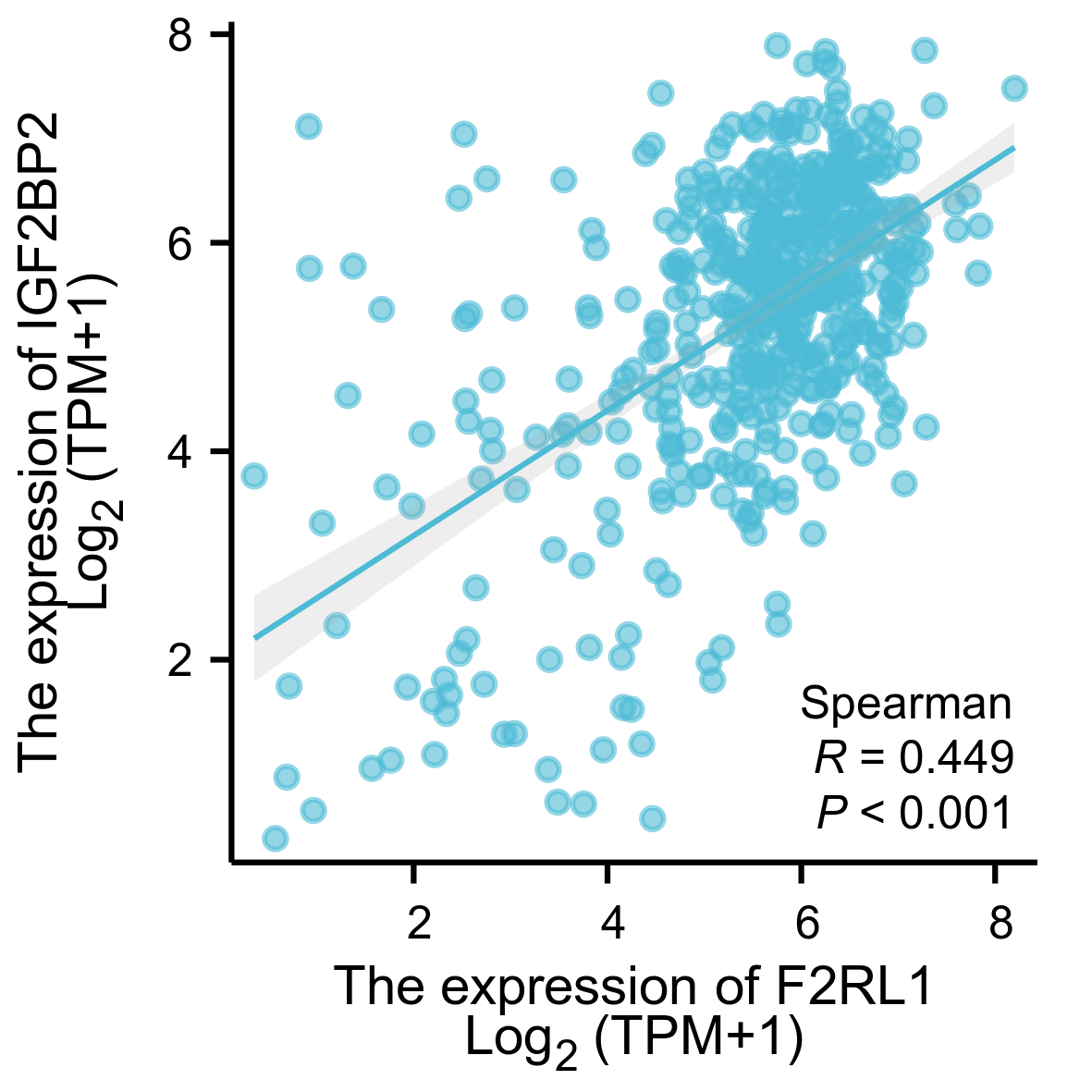

Supplement: Supplemental Information 5 [file peerj-14-20970-s005.zip › Figure 7/B-M/Significance/R》0.3/IGF2BP2.tiff]

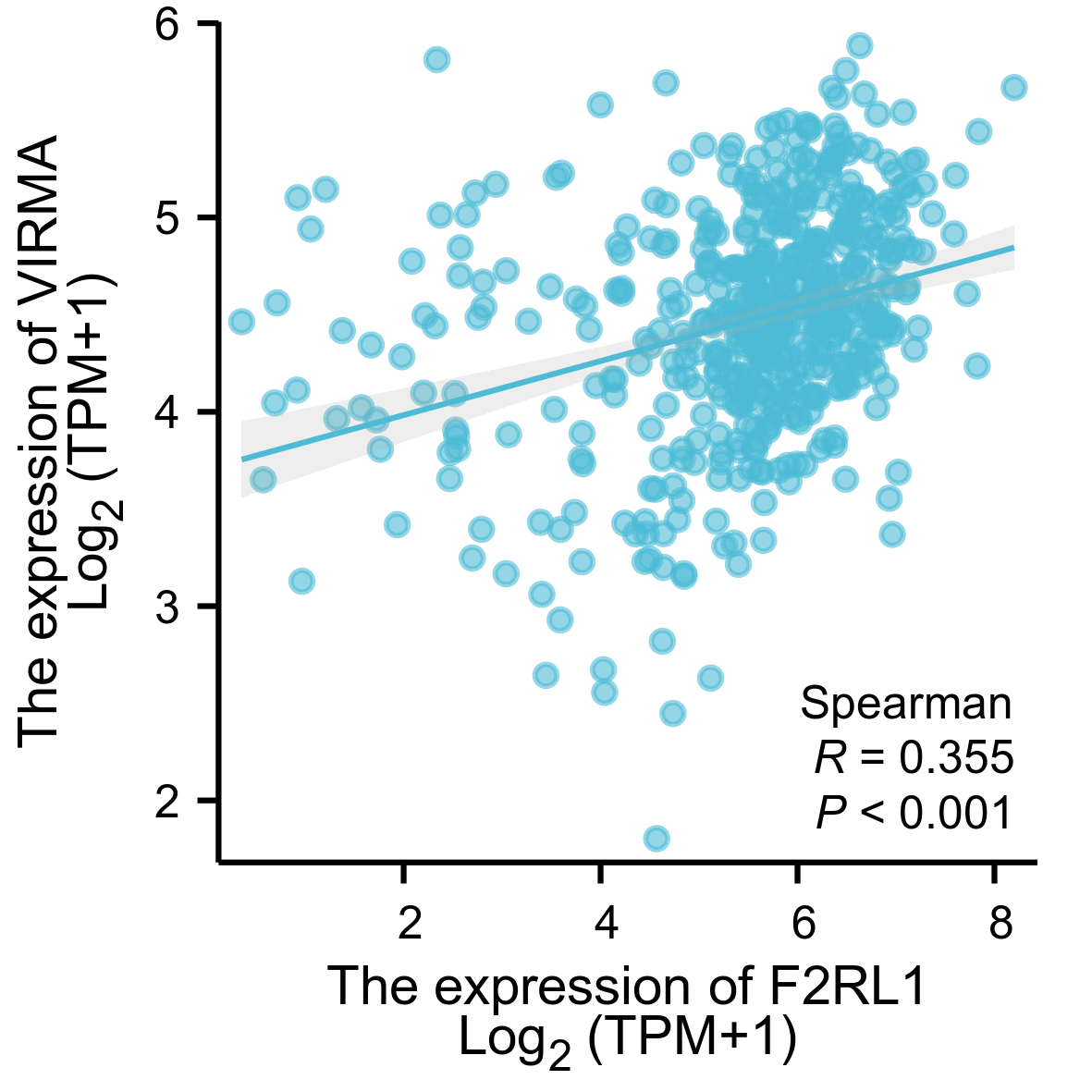

Supplement: Supplemental Information 5 [file peerj-14-20970-s005.zip › Figure 7/B-M/Significance/R》0.3/VIRMA.tiff]

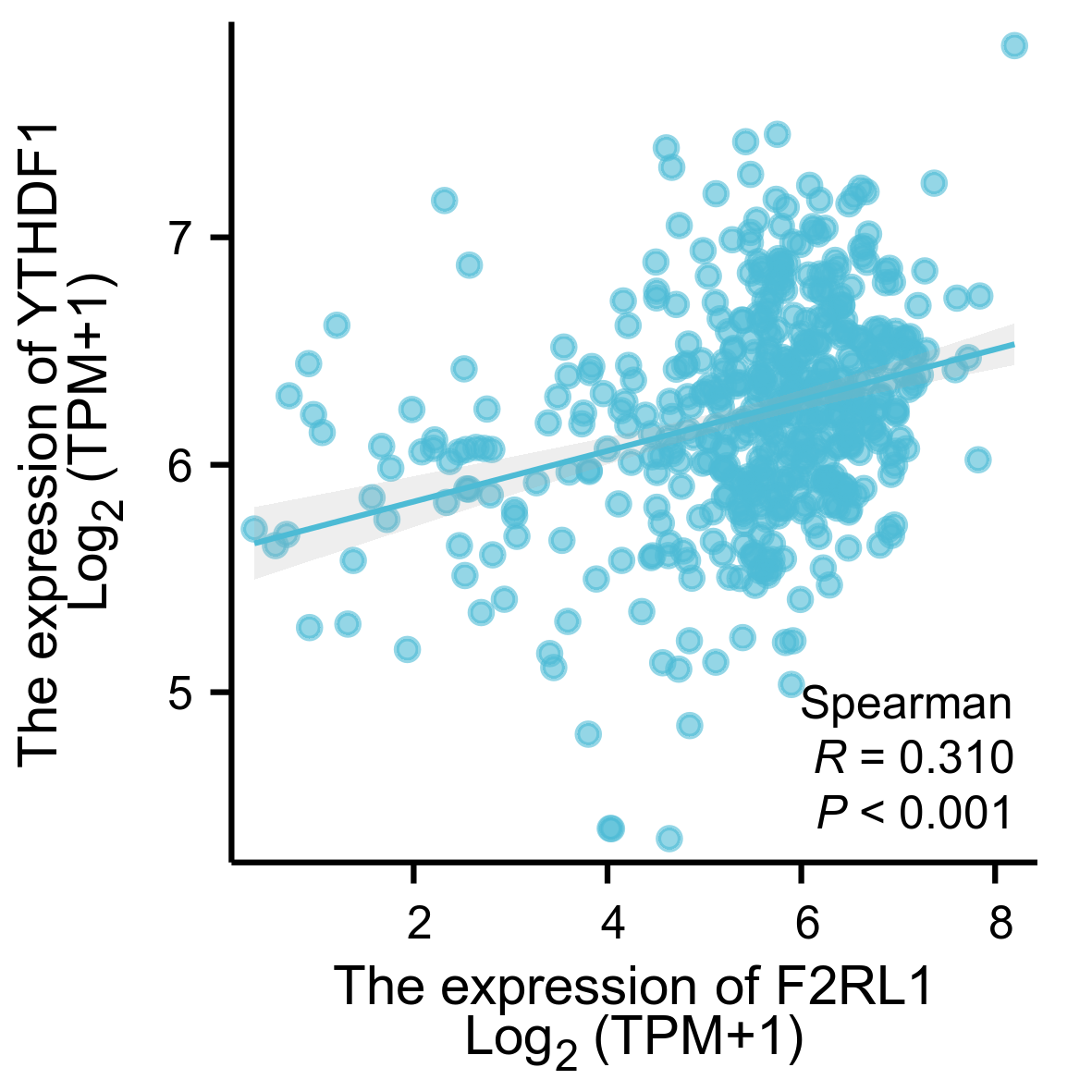

Supplement: Supplemental Information 5 [file peerj-14-20970-s005.zip › Figure 7/B-M/Significance/R》0.3/YTHDF1.tiff]

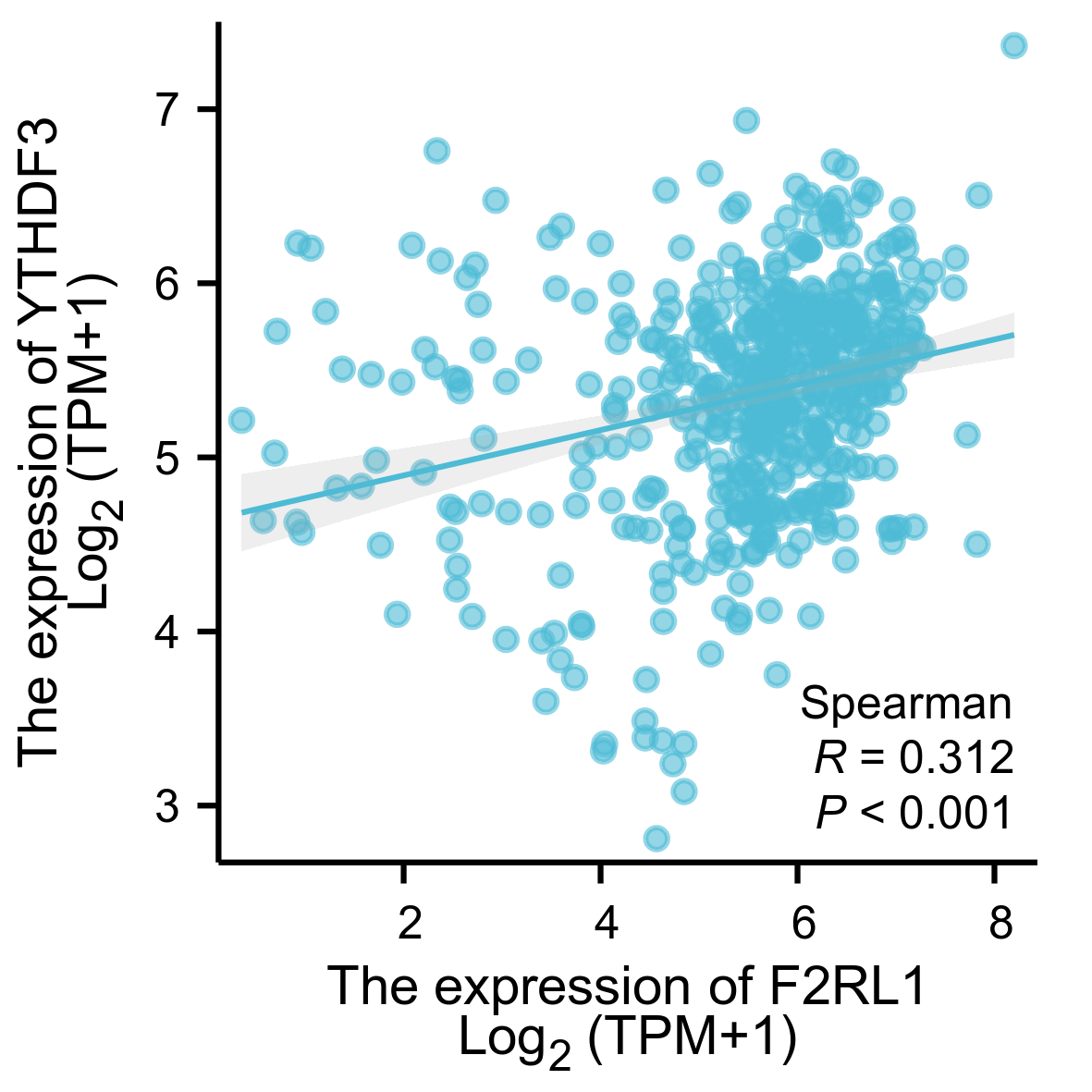

Supplement: Supplemental Information 5 [file peerj-14-20970-s005.zip › Figure 7/B-M/Significance/R》0.3/YTHDF3.tiff]

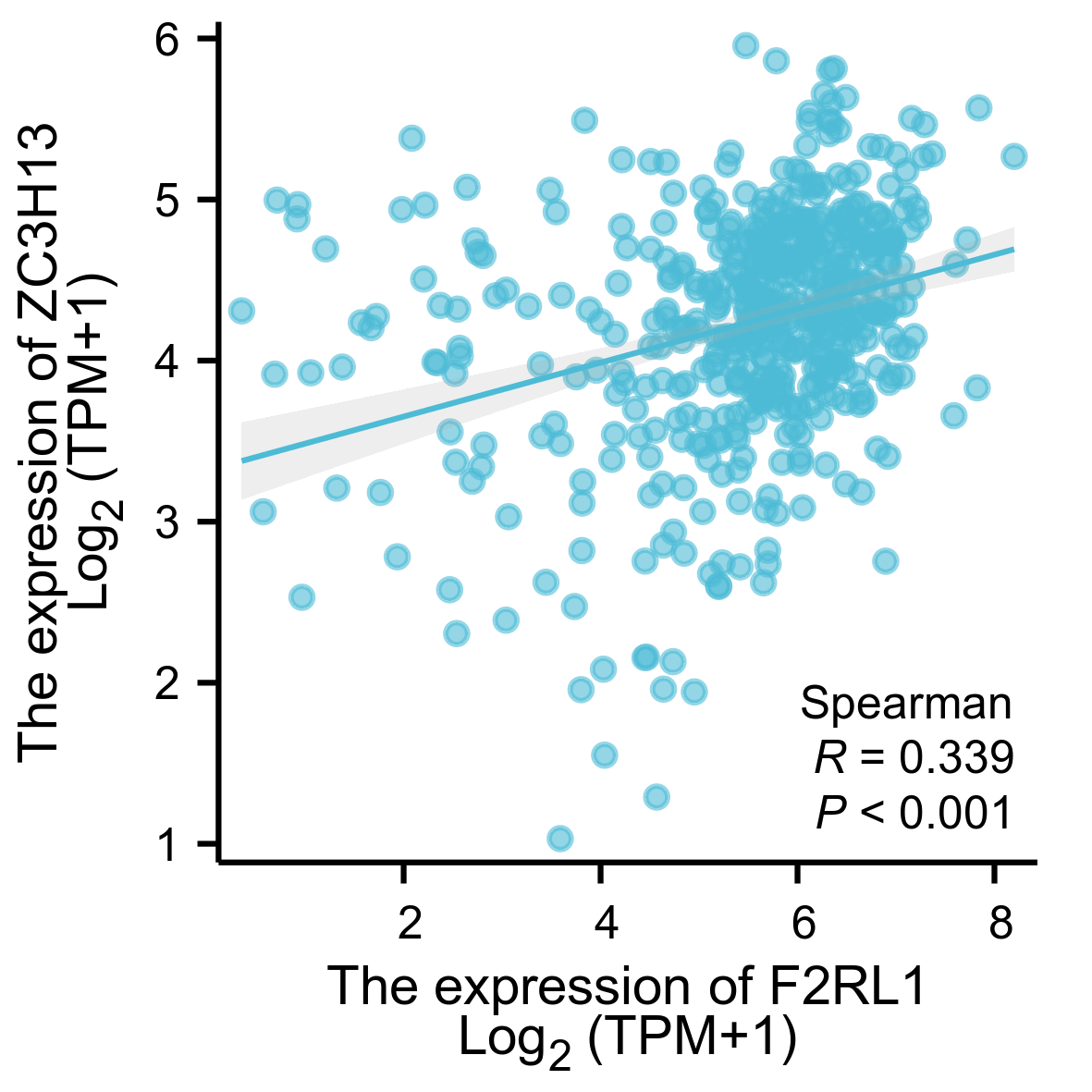

Supplement: Supplemental Information 5 [file peerj-14-20970-s005.zip › Figure 7/B-M/Significance/R》0.3/ZC3H13.tiff]

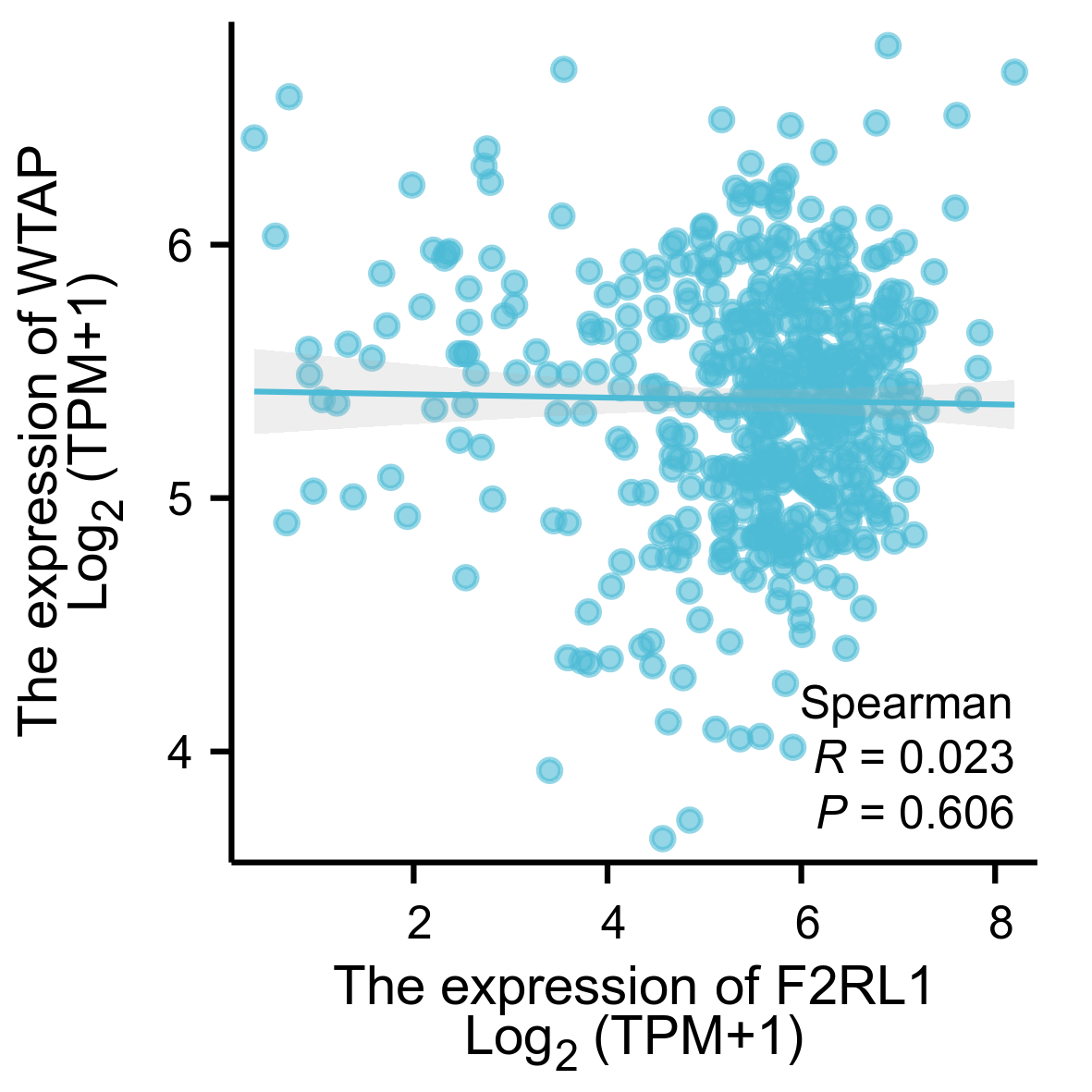

Supplement: Supplemental Information 5 [file peerj-14-20970-s005.zip › Figure 7/B-M/Significance/WTAP.tiff]

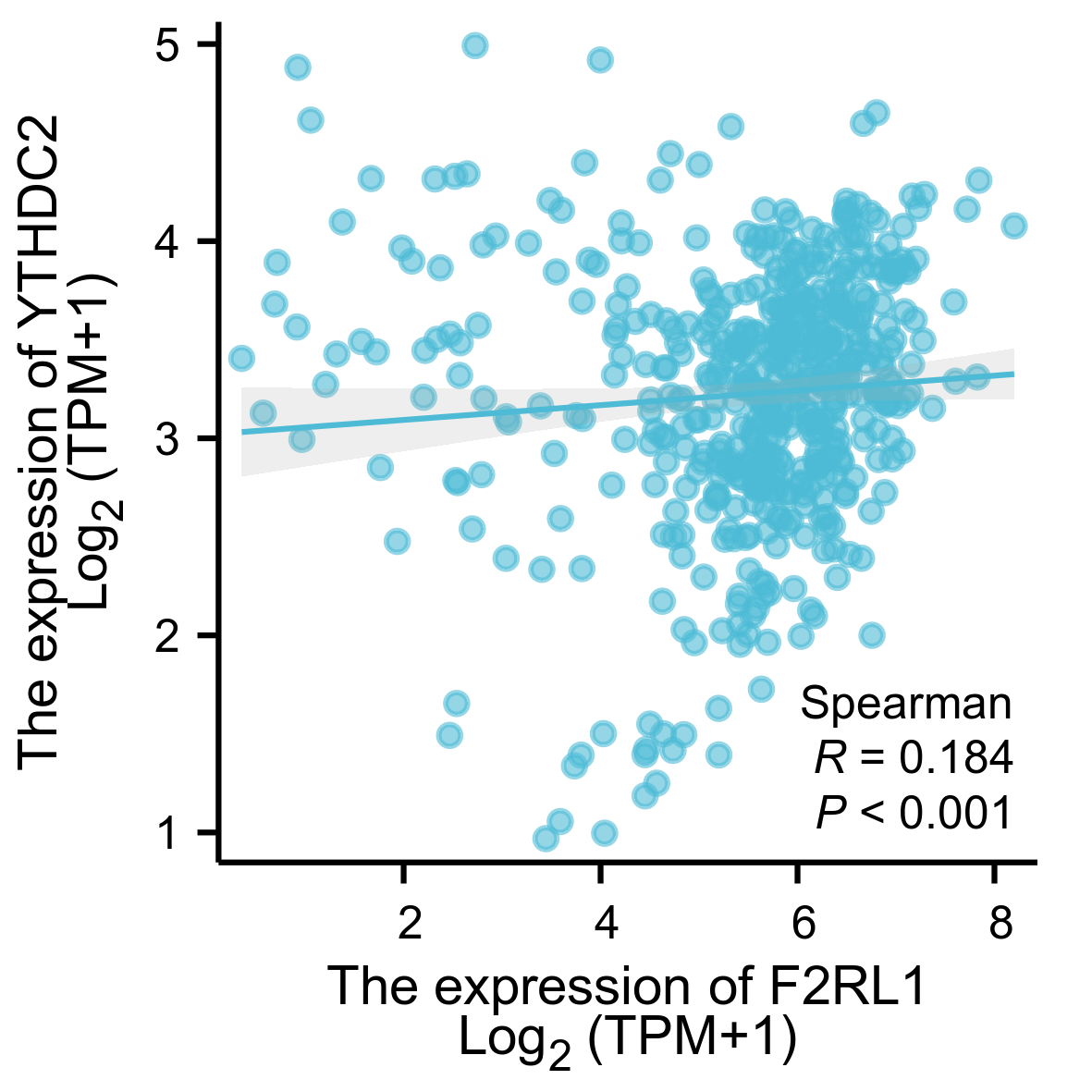

Supplement: Supplemental Information 5 [file peerj-14-20970-s005.zip › Figure 7/B-M/Significance/YTHDC2.tiff]

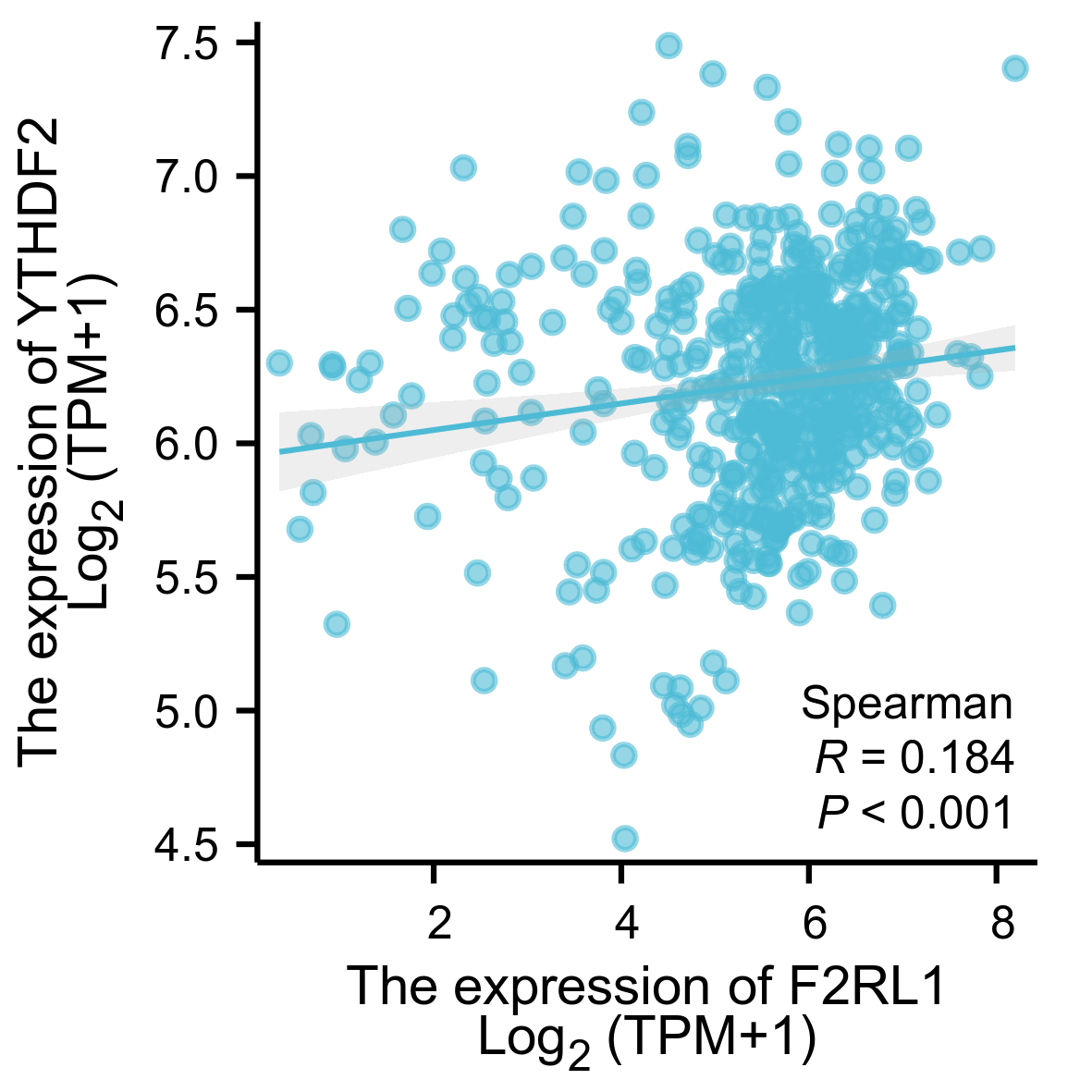

Supplement: Supplemental Information 5 [file peerj-14-20970-s005.zip › Figure 7/B-M/Significance/YTHDF2.tiff]

# FTO

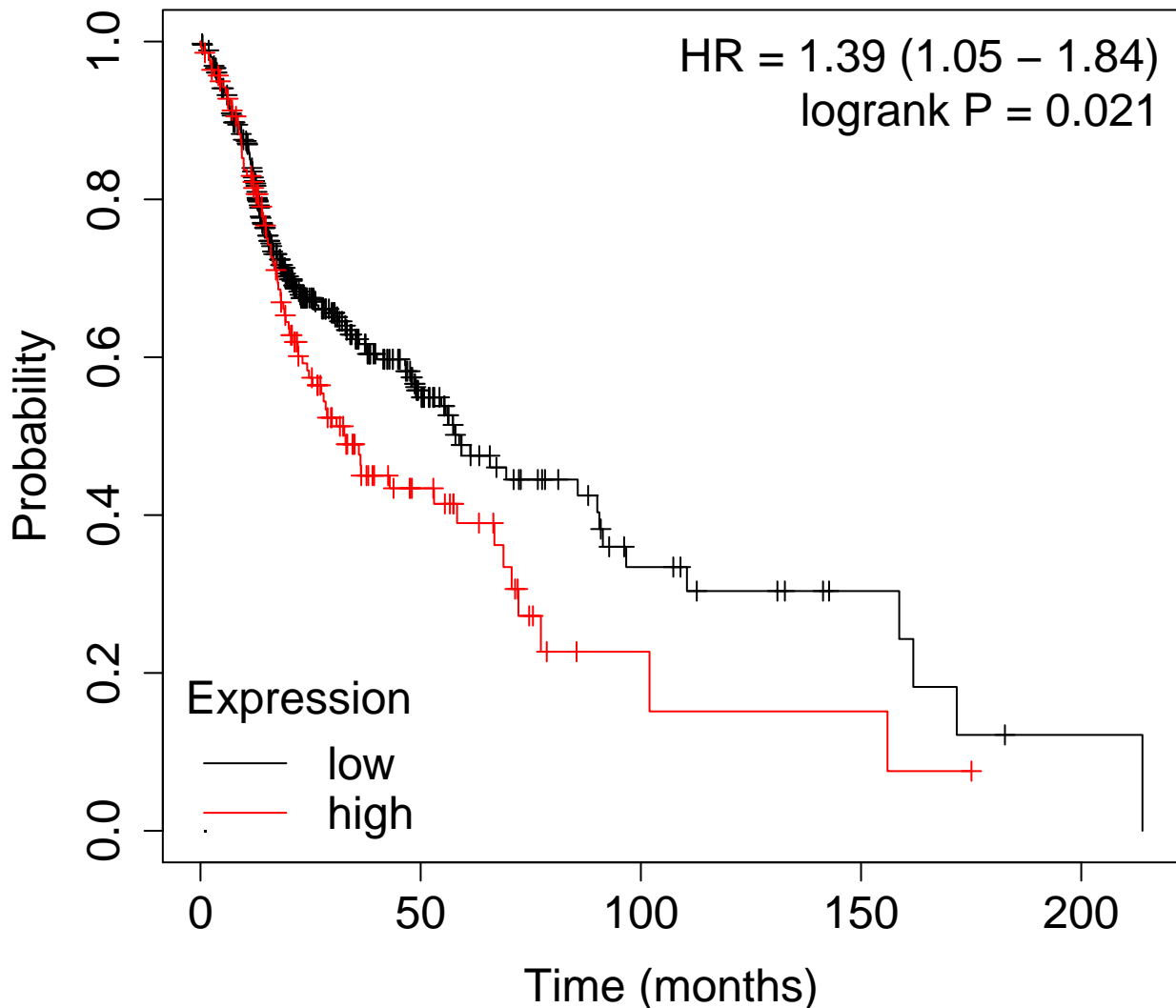

Number at risk

|      |     |    |    |   |   |
|------|-----|----|----|---|---|
| low  | 359 | 63 | 13 | 5 | 1 |
| high | 140 | 23 | 3  | 2 | 0 |

Supplement: Supplemental Information 5 [file peerj-14-20970-s005.zip › Figure 7/N-S/032005_480800_664186e575611_FTO.pdf]

# HNRNPC

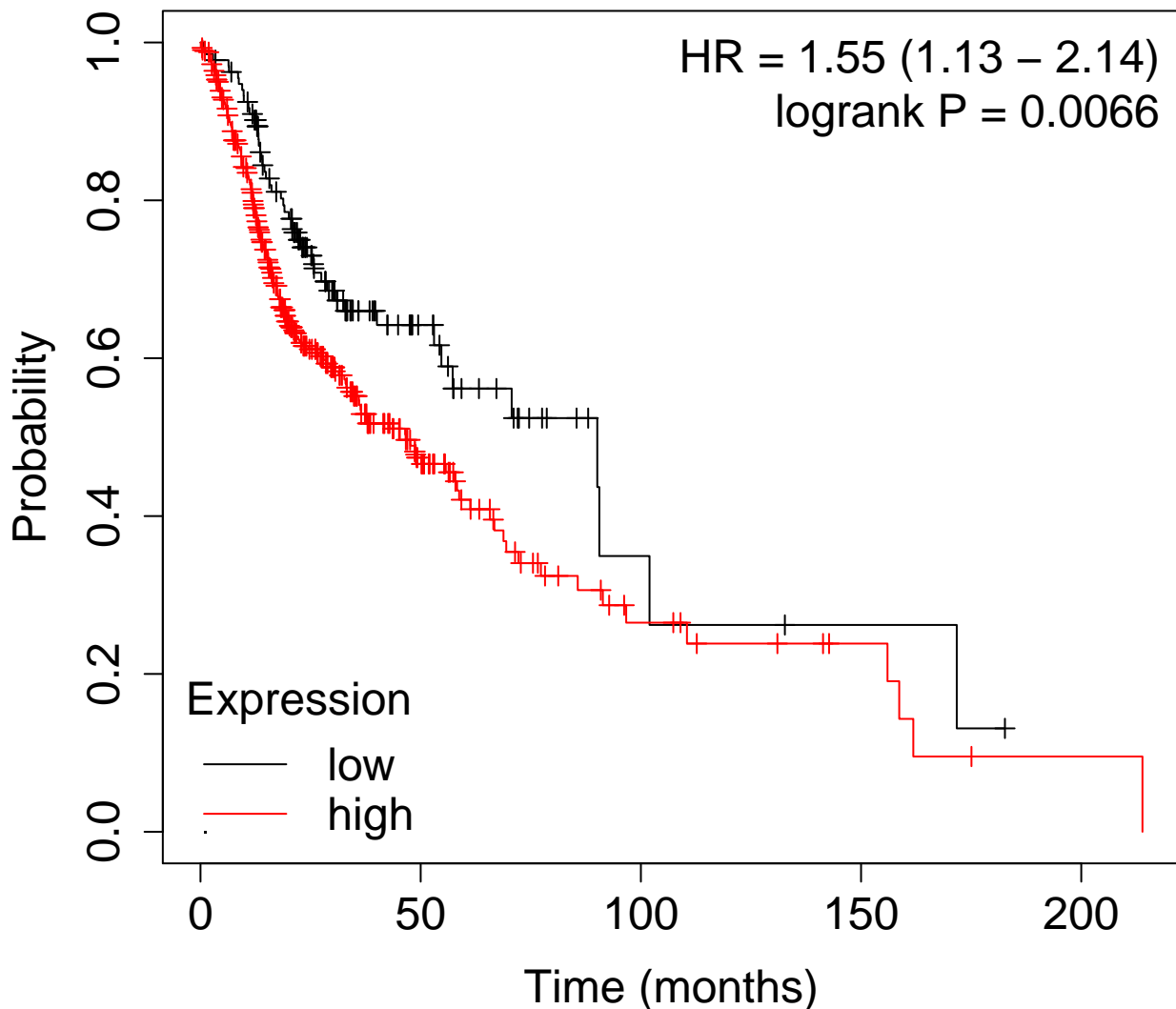

Number at risk

|      |     |    |    |   |   |
|------|-----|----|----|---|---|
| low  | 134 | 27 | 4  | 2 | 0 |
| high | 365 | 59 | 12 | 5 | 1 |

Supplement: Supplemental Information 5 [file peerj-14-20970-s005.zip › Figure 7/N-S/032114_640700_6641872a9c6ef_HNRNPC.pdf]

# IGF2BP1

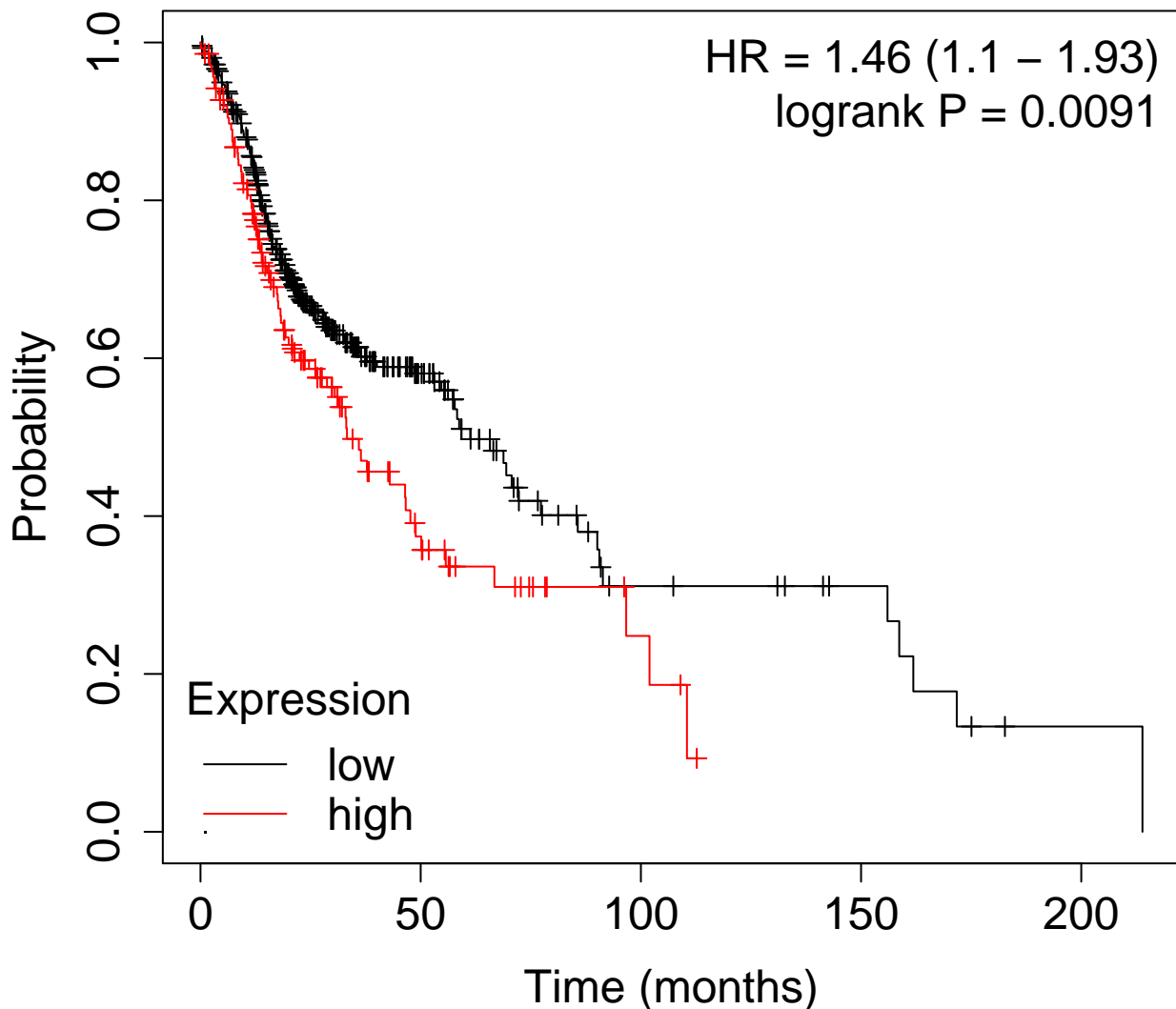

Number at risk

low  
high

360  
139

64  
22

12  
4

7  
0

1  
0

Supplement: Supplemental Information 5 [file peerj-14-20970-s005.zip › Figure 7/N-S/032148_710800_6641874cad8a5_IGF2BP1.pdf]
