# Supplementary material for: Coagulation factor II receptor-like 1 as a prognostic and immuno-modulatory factor in head and neck squamous cell carcinoma
Source: PeerJ. 2026 Mar 18;14:e20970. doi: 10.7717/peerj.20970 (PMC13005615; doi:10.7717/peerj.20970)
Supplement: Supplemental Information 5 [file peerj-14-20970-s005.zip › Figure 5/D-G/B-cell.zip!/reports.html]

仙桃-免疫浸润-散点图-在线分析报告


免疫浸润-散点图-在线分析报告

导出时间: 2024-05-20 21:04:36

目录

- 免疫浸润-散点图

- 统计描述

- 异常值分析

- 正态性检验(Shapiro-Wilk normality test)

- 相关性分析

- 方法学

免疫浸润-散点图

免疫浸润-散点图

**相关性散点图**: 分析1个变量和另外1个或者2个变量之间的相关性

**统计方法**: Spearman

相关系数为正，说明两个变量之间存在正相关关系; 相关系数为负，说明两个变量之间存在负相关关系;

相关系数绝对值代表相关程度，0-0.3代表弱或者不相关; 0.3-0.5代表弱相关; 0.5-0.8代表中等程度相关; 0.8-1代表强相关

相关是否有统计学意义还需要结合p值来查看

统计描述

各个组对应常见「统计描述指标」

| 组别 | 数目 | 最小值 | 最大值 | 中位数(Median) | 四分位距(IQR) | 下四分位 | 上四分位 | 均值(Mean) | 标准差(SD) | 标准误(SE) |
| --- | --- | --- | --- | --- | --- | --- | --- | --- | --- | --- |
| F2RL1 | 502 | 0.35558 | 8.2007 | 5.7588 | 1.2863 | 5.0505 | 6.3369 | 5.4631 | 1.3451 | 0.060034 |
| B cells | 502 | 0.085785 | 0.70678 | 0.30098 | 0.12063 | 0.24058 | 0.36121 | 0.30556 | 0.092595 | 0.0041327 |

异常值分析

离群值 = Q1(下四分位) - 1.5\*IQR(四分位间距) 或者 Q3(上四分位) + 1.5\*IQR(四分位间距)

异常值 = Q1(下四分位) - 3.0\*IQR(四分位间距) 或者 Q3(上四分位) + 3.0\*IQR(四分位间距)

| 组别 | 离群值 | 异常值 |
| --- | --- | --- |
| F2RL1 | 2.81485800155505,... | 0.916170875339791... |
| B cells | 0.608682732824903... |  |

各组离群值和异常值如上所示，如数据确认非人为记录错误，可不进行处理

正态性检验(Shapiro-Wilk normality test)

| 组别 | 自由度(df) | 统计量 | p值 |
| --- | --- | --- | --- |
| F2RL1 | 501 | 0.87977 | 2.69e-19 |
| B cells | 501 | 0.98071 | 3.32e-06 |

正态性检验结果显示，存在有不满足正态分布的变量(P < 0.05)，建议选择用 非参数检验方法(Spearman)

相关性分析

同时提供Pearson和Spearman统计方法，可以根据需要选择标注在图中的方法

| 方法 | 组别I | 组别J | 自由度(df) | 统计量 | 相关系数 | 置信区间(95%CI) | p值 |
| --- | --- | --- | --- | --- | --- | --- | --- |
| Pearson | F2RL1 | B cells | 500 | -7.6022 | -0.32189 | -0.39818 - -0.24116 | 1.45e-13 |
| Spearman | F2RL1 | B cells |  | 2.658e+07 | -0.26086 |  | 3.41e-09 |

相关系数为正，说明两个变量之间存在正相关关系; 相关系数为负，说明两个变量之间存在负相关关系;

相关系数绝对值代表相关程度，0-0.3代表弱或者不相关; 0.3-0.5代表弱相关; 0.5-0.8代表中等程度相关; 0.8-1代表强相关

相关是否有统计学意义还需要结合p值来查看

方法学

**软件**: R (4.2.1)版本

**R包**: ggplot2[3.3.6]

**处理过程:**

· 分析数据变量之间相关性后，用gglot2可视化结果

**补充说明:**

· 统计方法: Spearman

· 主分子: F2RL1[ENSG00000164251.5]

· 免疫浸润算法: 基于R包-GSVA[1.46.0](Hänzelmann et al., 2013)中提供的ssGSEA算法, 利用Immunity文章(Bindea, Gabriela, et al., 2013)提供的24种免疫细胞的markers来计算对应云端数据的免疫浸润情况，具体24种免疫细胞可查看对应参考文献

· 免疫细胞: B cells

**参考文献:**

Hänzelmann, Sonja, Robert Castelo, and Justin Guinney. GSVA: gene set variation analysis for microarray and RNA-seq data. BMC bioinformatics 14.1 (2013): 1-15.文献链接

Bindea, Gabriela, et al. Spatiotemporal dynamics of intratumoral immune cells reveal the immune landscape in human cancer. Immunity 39.4 (2013): 782-795.文献链接

**数据:**

· 数据获取: 从TCGA数据库 ( https://portal.gdc.cancer.gov ) 下载并整理TCGA-HNSC(头颈鳞状细胞癌)项目STAR流程的RNAseq数据并提取TPM格式的数据 以及 临床数据

· 数据过滤策略: 去除正常+去除无临床信息+去除重复

· 数据处理方法: log2(value+1)
